# Tandem Iridium-Catalyzed Decarbonylative C–H Activation of Indole: Sacrificial Electron-Rich Ketone-Assisted Bis-arylsulfenylation

**DOI:** 10.1021/acs.orglett.1c00829

**Published:** 2021-04-28

**Authors:** Subban Kathiravan, Prasad Anaspure, Tianshu Zhang, Ian A. Nicholls

**Affiliations:** Bioorganic & Biophysical Chemistry Laboratory, Linnaeus University Centre for Biomaterials Chemistry, Department of Chemistry & Biomedical Chemical Sciences, Linnaeus University, Kalmar 39182, Sweden

## Abstract

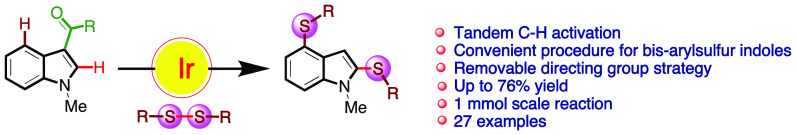

Described herein is a decarbonylative tandem C–H bis-arylsulfenylation of indole at the C2 and C4 C–H bonds through the use of pentamethylcyclopentadienyl iridium dichloride dimer ([Cp*IrCl_2_]_2_) catalyst and disulfides. A new sacrificial electron-rich adamantoyl-directing group facilitates indole C–H bis-functionalization with a traceless in situ removal. Various differently substituted disulfides can be easily accommodated in this reaction by a coordination to Ir(III) through the formation of six- and five-membered iridacycles at the C2 and C4 positions, respectively. Mechanistic studies show that a C–H activation-induced C–C activation is involved in the catalytic cycle.

Indoles are the fourth most-prominent heterocyclic motif present in currently marketed drugs and pharmaceuticals.^1^ The transition-metal-catalyzed C–H functionalization of indole provides access to a broad array of functionalities,^2^ and indole is of strategic importance, as it overcomes limitations associated with classical reactions by circumventing the need for prefunctionalization, and it provides an efficient atom and step economy.^3^

Many of the methods used for the C–H functionalization of indole at the C2 or C4 positions involve nonremovable directing-group assistance.^4^ For example, a Pd-catalyzed C–H alkenylation at the C4 position has been described by Jia using an amino acid as a directing group.^5^ Prabhu and co-workers have demonstrated a Ru-catalyzed C–H alkenylation using the formyl group to control the selectivity at the C4 position.^6^ Later they studied Rh- and Ru-catalyzed C–H alkenylations using complementary acetyl and trifluoromethylacetyl groups for the regioselective C–H activation reaction for controlling selectivity at C2 and C4.^7^ Zhang has shown decarboxylative C2/C4 C–H alkenylation reactions using a Rh catalyst.^8^ An iridium-catalyzed C4 C–H amination has been disclosed independently by Prabhu and You.^9^ The Pd-catalyzed C4 arylation was studied by Zhi, and the acetyl-directing group has been removed in a separate step.^10^ Recently, Miura et al. reported the use of thiomethyl ether as an efficient directing group for C4 C–H functionalization.^11^ In another study, You described a pivaloyl group-assisted C2/C4 heteroarylation of indoles by a controlled metalation tuning.^12^ However, in all the above cases stoichiometric quantities of reagents are required to remove the directing group, which significantly limits the utility of these approaches in a synthesis. Accordingly, the development of clean and user-friendly methods for indole C–H functionalization using readily removable directing groups remains a highly desirable challenge.

Thioethers are frequently found in pharmaceutical agents, polymeric materials, and biologically active natural products.^13^ Over the past few years the use of C–H activation reactions has emerged as a powerful tool for thioether preparation.^14^ Yu and co-workers have reported ligand-promoted Rh(III)-catalyzed aryl thiolation reactions using amide directing groups,^15^ and Daugulis presented an aryl sulfenylation of benzoic acid derivatives using bidendate aminoquinoline directing groups.^16^ Despite these advances the examples of the arylthiolation of indoles remain extremely limited, with the regiocontrolled C2/C4 methyl thiolation of indoles under Rh-catalyzed conditions using oxime as a directing group by Samatha et al. the only example reported.^17^ With the above background, we envisioned that a removable directing group for the arylsulfenylation using an iridium catalyst would provide a new complementary method for indole C–H functionalization. However, there are a number of challenges associated with this proposal, including (1) the deactivation of reactive iridacycles through sulfur ligation, (2) the competition between five- and six-membered iridacycle pathways involving the C2 and C4 positions, respectively, and (3) the possibility of undesired side-product formation.

Thus far, the established indole C–H functionalization directing groups have predominantly afforded six-membered metallacycles rather than their less stable five-membered counterparts.^18^ Inspired by the electron-rich ketone-assisted sp^2^ C–H activation of ortho C–H bonds, we hypothesized that the readily accessible and inexpensive adamantoyl group could be used to generate dual metallacycles to facilitate a C–S bond formation (Scheme 1). This choice was further motivated by its utility as a directing group in C–H activation reactions for iridium-catalyzed amination and palladium-catalyzed amidation, among others.^19^ Its use in conjunction with indole C–H functionalization was yet to be examined. Here, we explored and demonstrated the iridium(III)-catalyzed decarbonylative direct arylsulfenylation of indole at C2/C4 C–H bonds.

**Scheme 1:**
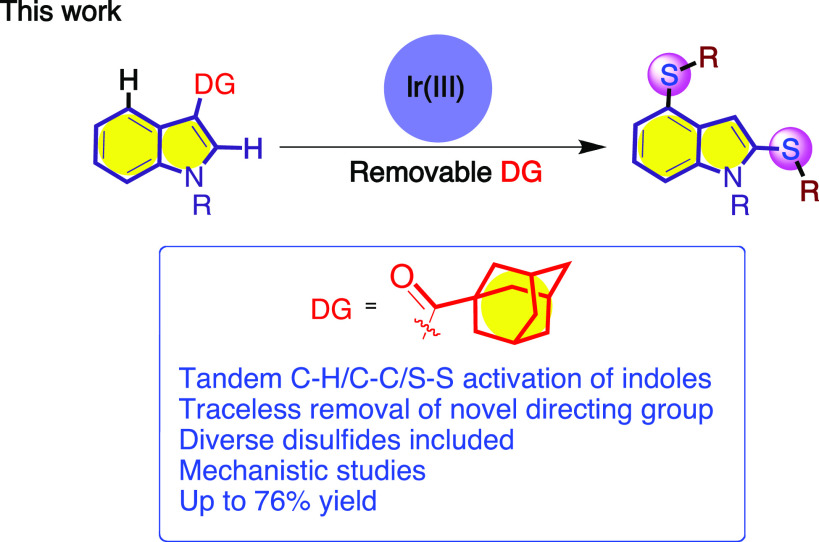
This Work Proposal—Iridium-Catalyzed C–H Activation of Indole

At the outset, to test the hypothesis we chose **1a** as test substrate to probe the reactivity of iridium(III) catalysis (using pentamethylcyclopentadienyl iridium dichloride dimer ([IrCp*Cl_2_]_2_)) in the arylsulfenylation of **1a** with disulifide **2a**, AgNTf_2_ as a silver additive, and silver carbonate as a terminal oxidant. To our surprise, when the initial reactions were performed using anhydrous 1,2-dichloroethane (DCE) as solvent at 120 °C for 22 h, the bis-aryl sulfenylated C–H activation product **3a** was obtained in 68% isolated yield with no evidence (thin-layer chromatography (TLC)) of monosubstituted indole products. The addition of acidic additives, L-MPAA ligands,^20^ which are commonly used in 3d and 4d metal catalysis to promote electrophilic metalation processes, gave lower isolated yields indicating that this reaction does not follow an electrophilic metallation pathway (Table 1, entries 1 & 2). Changing the oxidant from silver carbonate to Ag_2_O or to AgOAc reduced the product formation (Table 1, entries 3 & 4), whereas the use of AgF gave no product (Table 1, entry 5). Silver carbonate could be replaced with Cu(OAc)_2_, albeit with a reduced yield, suggesting that the acetate ligand is not required for the coordination of the metal center (Table 1, entry 7). Changing the solvent from DCE to dichloromethane also resulted in a lower yield (Table 1, entry 8). When the reaction was performed under air, no consumption of starting materials was observed (Table 1, entry 9). A decrease of the reaction temperature to 60 °C lowered the yield to 35% (Table 1, entry 10), and at room temperature no reaction was observed (Table 1, entry 11). Two different catalysts, namely, [RuCl_2_(*p*-cymene)_2_]_2_ and pentamethylcyclopentadienyl rhodium dichloride dimer ([RhCp*Cl_2_]_2_), both known for their capacity to catalyze C–H transformations, were investigated, but neither gave the product in a better yield (Table 1, entries 12 & 13).

**Table 1:**
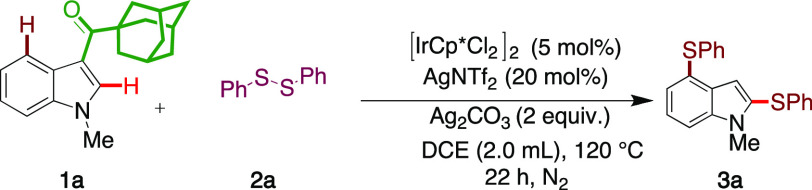
Optimization of Reaction Conditions and Control Experiments^a^

entry	deviation from above	yield^b^ (%)
1	PivOH	17
2	1-AdCOOH	35
3	Ag_2_O	15
4	AgOAc	28
5	AgF	NR
6	Cu(OAc)_2_	55
7	DCM as solvent	16
8	under air	NR
9	at 60 °C	35
10	at RT	NR
11	[RuCl_2_(*p*-cymene)_2_]_2_	NR
12	[RhCp*Cl_2_]_2_	50

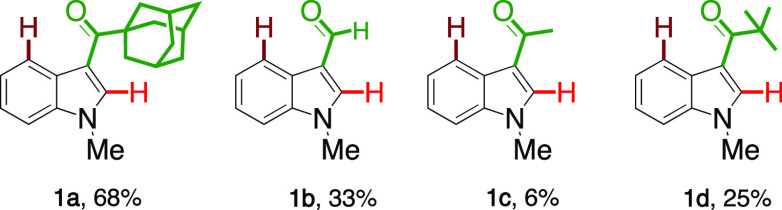

a Reaction conditions: **1a** (0.17 mmol), **2a** (0.25 mmol), [IrCp*Cl_2_]_2_ (5 mol %), AgNTf_2_ (30 mol %), Ag_2_CO_3_ (2.0 equiv), 1,2-DCE (2 mL), 120 °C, 22 h.

b Isolated yield; NR = no reaction.

Having established optimal conditions for this iridium(III)-catalyzed C–H activation, we focused our attention on exploring the scope of the directing ketones on the reaction by using a series of carbonyl substituents. To our delight, the more electron-rich adamantoyl derivative gave the product in the highest yield, 68%. The acetyl and pivaloyl groups gave the expected products in 6 and 25% yields, respectively, thus illustrating the importance of having an electron-rich ketone for generating five- and six-membered metallacycles. Interestingly, the formyl group also delivered the decarbonylative aryl sulfenated product in 33% yield.

With the optimized reaction conditions in hand, we first examined the effect of substitution of the disulfide on reaction with the model substrate **1a** (Table 2). Both electron-donating and electron-withdrawing groups were tolerated under the reaction conditions, and the reaction efficiency was found to be significantly affected by disulfide aromatic ring substituents. The 2-Me (**3b**) group substitutions gave the product in 34% yield, but the less sterically hindered 3-Me (**3c**) and 4-Me (**3d**) gave 50% and 55% yields. We then explored the influence of halogenated (Cl, Br, & F) disulfides and found that, irrespective of their position (ortho, meta, or para), all gave the products (**3e**–**3l**) in moderate to good yields (38–70%), the products being amenable for further transformations. The reaction proceeded smoothly with trifluoromethyl (**3m**, 43%) and *tert*-butyl (**3n**, 37%) substituted disulfides. Furthermore, the use of disubstituted bulky disulfides provided the products (**3o**–**3s**) in moderate yields (48–60%). To our surprise, we used various aliphatic disulfides and 2-pyridine disulfide in this study but without success (see the Supporting Information).

**Table 2:**
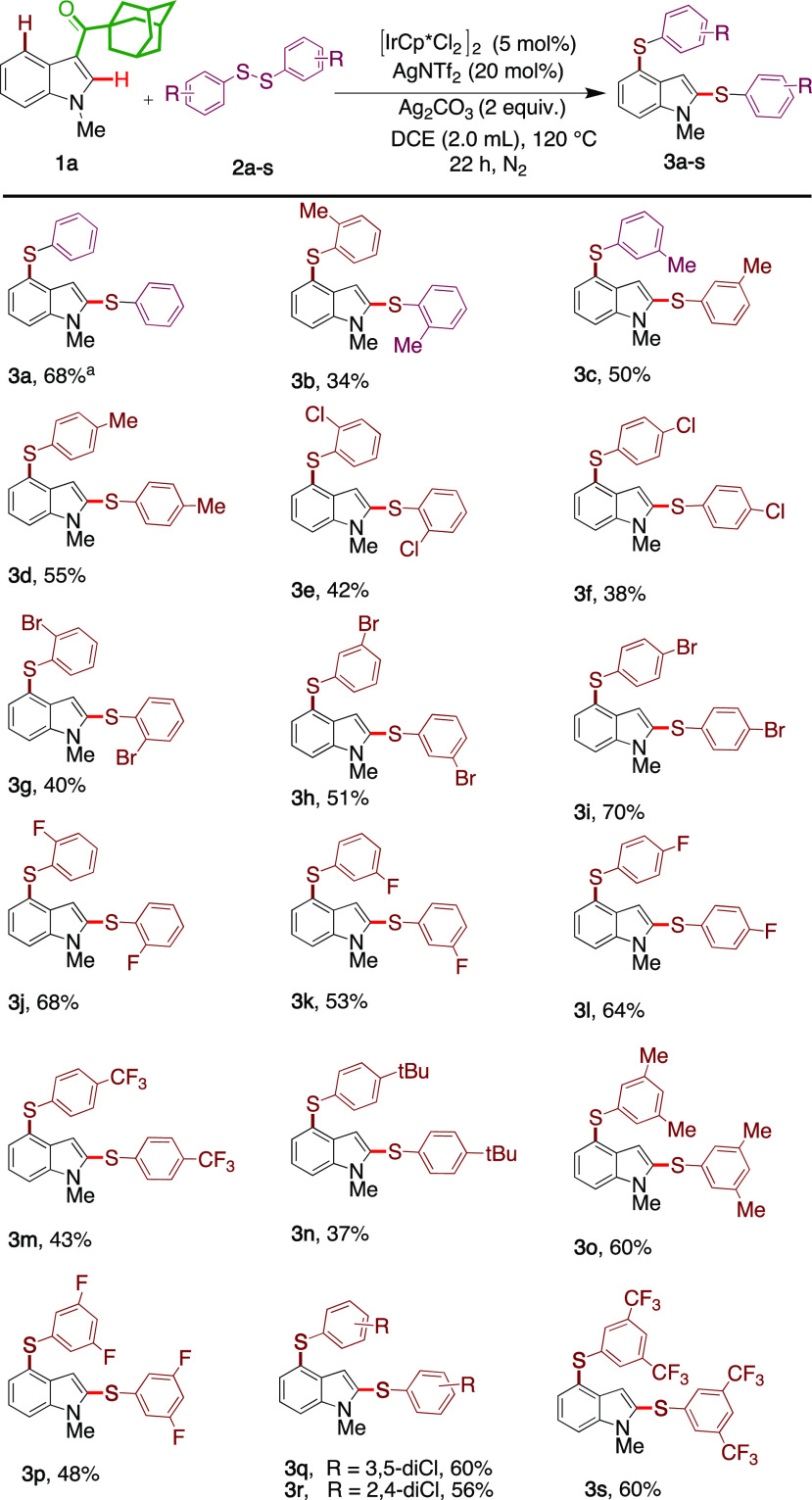
Scope of Disulfides^a^^,^^b^

a Reaction conditions: substrate **1** (0.17 mmol, 1 equiv), disulfide **2** (0.25 mmol, 2 equiv), [IrCp*Cl_2_]_2_ (5 mol %), AgNTf_2_ (30 mol %), Ag_2_CO_3_ (0.65 mmol, 2 equiv), 1,2-DCE (2 mL), 120 °C, 22 h.

b Isolated yield.

Encouraged by the broad scope of disulfides accepted by the model substrate **1a**, we proceeded to explore the reaction tolerance to an indole substitution (Table 3). To our delight, indoles bearing Cl (**4b**), Br (**4c**), I (**4d**), F (**4e**), CN (**4f**), and COOMe (**4g**) at the C5 position were all tolerated. C6 substituted indoles (**1h**–**1j**) with electron-donating and electron-withdrawing groups were all compatible and provided the products (**4h**–**4j**) in 70–71% yields. The C7 methyl-substituted indole also gave the product (**4k**) in 32% yield.

**Table 3:**
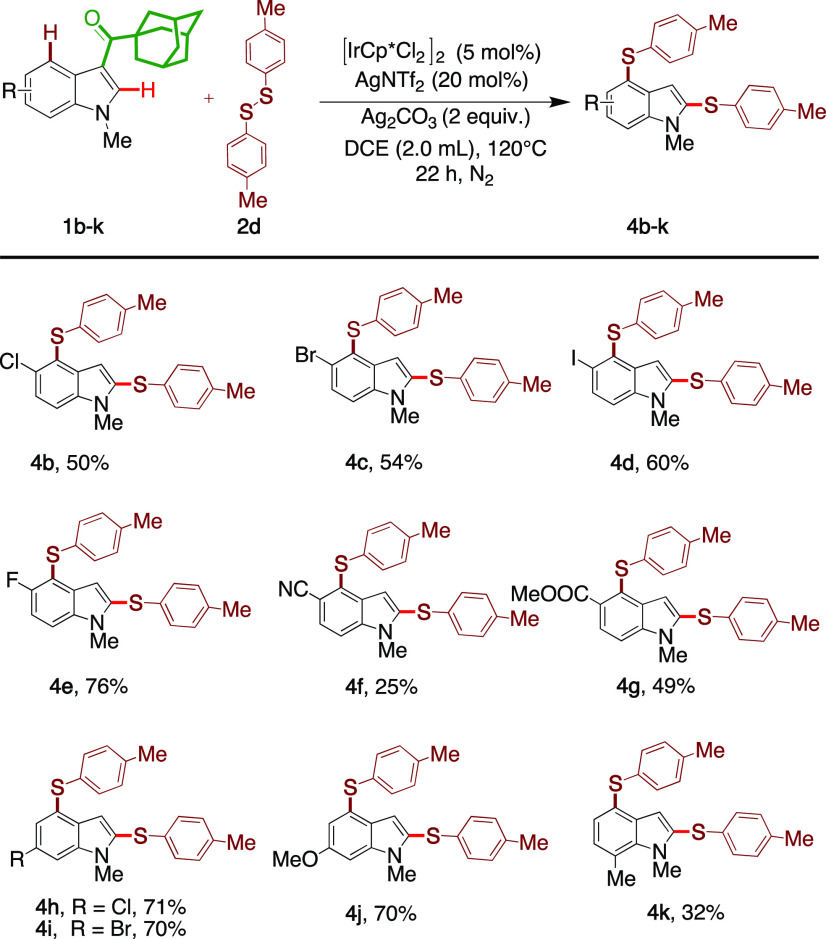
Scope of Indoles^a^^,^^b^

a Reaction conditions: substrate **1** (0.17 mmol, 1 equiv), disulfide **2** (0.25 mmol, 2 equiv), [IrCp*Cl_2_]_2_ (5 mol %), AgNTf_2_ (30 mol %), Ag_2_CO_3_ (0.65 mmol, 2 equiv), 1,2-DCE (2 mL), 120 °C, 22 h.

b Isolated yield.

Given the synthetic applicability of the decarbonylative iridium(III)-catalyzed C–H activation, we devised a series of control experiments in order to delineate the mechanism underlying this reaction (Scheme 2). To this end, an intermolecular competition experiment between electron-rich and electron-deficient substituted disulfides (**2d** & **2m**) with **1a** showed that an inherently higher reactivity was observed with the electron-deficient disulfide. Additionally, we did not observe the cross-sulfenylation product. This indicates that the reaction does not proceed via a concerted metallation/deprotonation (CMD) (Scheme 2a). We also found that the use of thiol (**5**) under these oxidative conditions generated the disulfide (**6**) as the only major product, and starting material (**1a**) was recovered, which further indicates that the disulfides are oxidatively added to the metal center during the C–H activation (Scheme 2b). Finally, reactions in deuterated solvents were performed (Scheme 2c). We observed 10% deuteration at C2 and 15% deuteration at C4 in the absence of silver carbonate, which suggests that the silver carbonate functions as both a promoter and an oxidant (Scheme 2c-1). Interestingly, when we studied the reaction in the presence of Ag_2_CO_3_, 82% of the D/H exchange was observed at the C4/C2 positions. This is indicative of the efficient formation of six- and five-membered iridacycles (Scheme 2c-2). This further emphasizes the role of silver carbonate in the mechanism, as this reaction needed a second silver additive in stoichiometric quantities to initiate the cyclometalation. The extent of the D/H exchange observed when using the electron-deficient ketone *N*-methyl-3-acetyl indole was lower at C2 (16%) than at C4 (51%). This suggests that the acetyl directing group preferentially forms the more stable six-membered iridacycle (Scheme 2c-3). We also extended this study to include the pivaloyl indole derivative, in which case a C4/C2 D/H exchange comparable to that of the more electron-rich adamantyl derivative was observed (Scheme 2c-4). We made several attempts to obtain the crystal structure of an iridacycle, to characterize the intermediate, but without success. The robustness of the iridium(III) catalysis was demonstrated by its use in a gram-scale (1 mmol) reaction, where no significant loss of catalytic activity was observed and with the product isolated in 52% (0.21 g) yield (Scheme 2d).

**Scheme 2:**
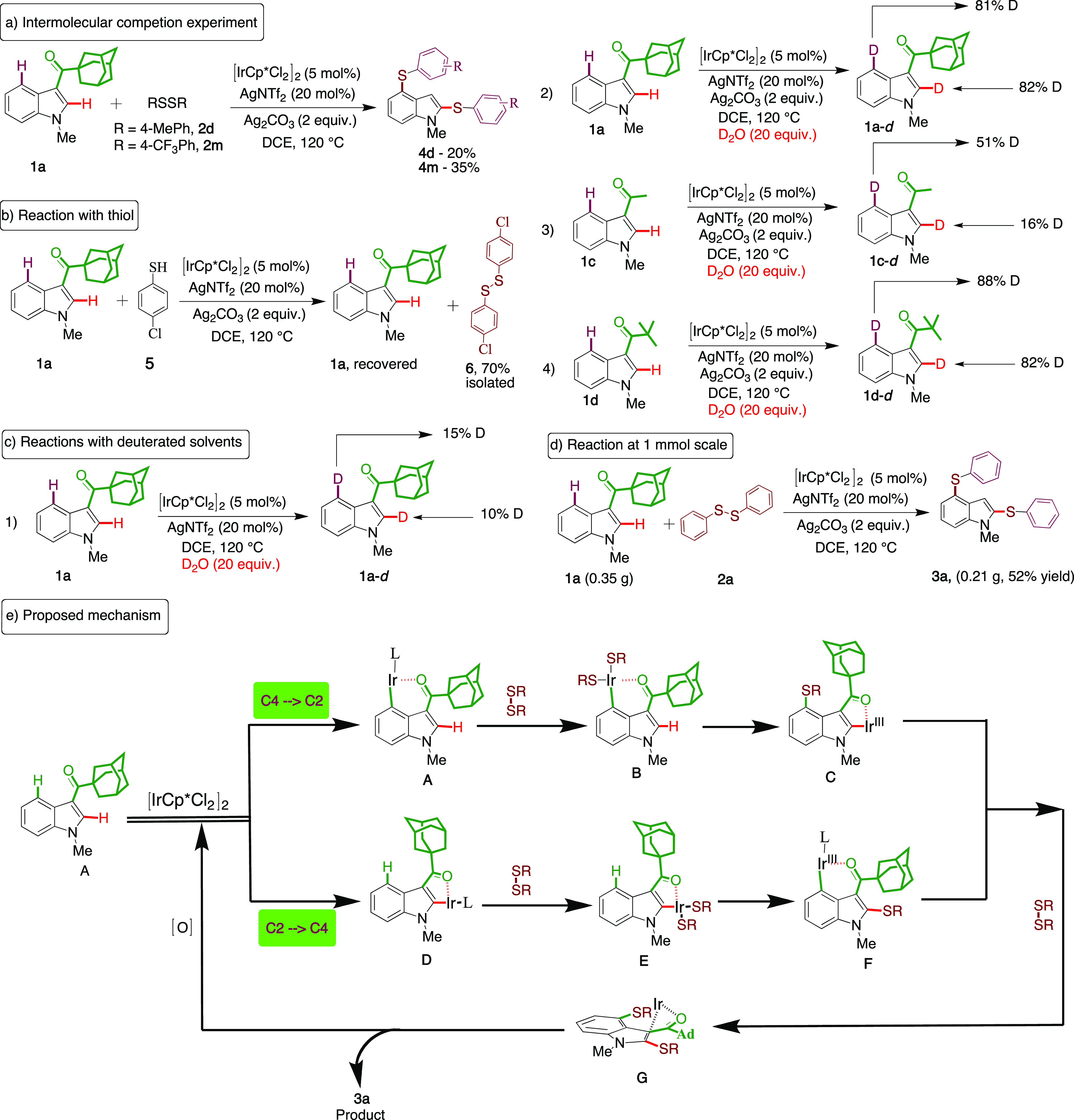
Mechanistic Studies and Proposed Mechanism

On the basis of the evidence from these mechanistic studies and in combination with reported data,^21^ we propose that the mechanism proceeds through a tandem pathway. Initially, the catalyst coordinates with either the C4 or C2 C–H bond and the electron-rich ketone to form either the corresponding six-membered (**A**, preferred) or five-membered (**D**) metallacycle, respectively. The oxidative addition of disulfide **2a** affords the complex **B** or **E**, which upon reductive elimination generates either complex **C** or **F**. After the reaction by both pathways, the iridacycle **G** is obtained following a carbonyl group dislocation. The high temperature can be anticipated to play a crucial role in decarbonylation.^12^ In the final step reductive elimination provides the product **3a**, and the reoxidation by silver regenerates the active catalyst (Scheme 2e).

## Conclusions

In summary, we have reported the first tandem decarbonylative arylsulfenylation of indoles at the C2/C4 positions through an iridium(III) catalyst. The electron-rich adamantyl ketone directing group presented here facilitates the formation of both six- and five-membered iridacycles and is amenable to reaction with a broad scope of disulfides with good yields. Further efforts to develop decarbonylative transition-metal-catalyzed regioselective C–H functionalizations are currently underway in our laboratory.

## Supporting Information Available

Details of synthetic procedures, NMR & HRMS spectra (PDF)
