# Supplementary material for: Tandem Iridium-Catalyzed Decarbonylative C–H Activation of Indole: Sacrificial Electron-Rich Ketone-Assisted Bis-arylsulfenylation
Source: Org Lett. 2021 Apr 28;23(9):3331–6. doi: 10.1021/acs.orglett.1c00829 (PMC8289288; doi:10.1021/acs.orglett.1c00829)
Supplement: Supplementary file 1 — ol1c00829_si_001.pdf [file ol1c00829_si_001.pdf]

Supporting Information

Tandem Iridium-Catalyzed Decarbonylative C–H Activation of Indole: Sacrificial Electron-Rich Ketone-Assisted Bis-arylsulfenylation

Subban Kathiravan*[a], Prasad Anaspure [a], Tianshu Zhang [a] and Ian A. Nicholls *[a]

Bioorganic & Biophysical Chemistry Laboratory, Linnaeus University Centre for Biomaterials Chemistry, Department of Chemistry & Biomedical Sciences, Linnaeus University, Kalmar SE-39182, Sweden
ian.nicholls@lnu.se
suppan.kathiravan@lnu.se

TABLE OF CONTENTS

1. Table of contents ……………………………………………………………………………………1

2. General information……………………………………………………………………………6

3. Synthesis of starting materials…………………………………………………………………6

4. Mechanistic studies ……………………………………………………………………………9

5. Characterization Data…………………………………………………………………………13

6. NMR spectra of isolated compounds…………………………………………………………..24

^{1}H NMR spectrum of **3a**……………………………………………………………………24

$^{13}C\{^{1}H\}$ NMR spectrum of **3a**…………………………………………………………24

HRMS spectrum of **3a**……………………………………………………………………………25

^{1}H NMR spectrum of **3b**……………………………………………………………………26

$^{13}C\{^{1}H\}$ NMR spectrum of **3b**…………………………………………………………26

HRMS spectrum of **3b**……………………………………………………………………………27

^{1}H NMR spectrum of **3c**……………………………………………………………………..28

$^{13}C\{^{1}H\}$ NMR spectrum of **3c**…………………………………………………………28

HRMS spectrum of **3c**……………………………………………………………………………29

^{1}H NMR spectrum of **3d**……………………………………………………………………..30

$^{13}C\{^{1}H\}$ NMR spectrum of **3d**…………………………………………………………30

HRMS spectrum of **3d**……………………………………………………………………………31

^{1}H NMR spectrum of **3e**……………………………………………………………………..32

$^{13}C\{^{1}H\}$ NMR spectrum of **3e**…………………………………………………………32

HRMS spectrum of **3e**……………………………………………………………………………33

^{1}H NMR spectrum of **3f**……………………………………………………………………..34

$^{13}C\{^{1}H\}$ NMR spectrum of **3f**…………………………………………………………..34

^{1}H NMR spectrum of **3g**……………………………………………………………………35

$^{13}C\{^{1}H\}$ NMR spectrum of **3g**…………………………………………………………35

HRMS spectrum of **3g**……………………………………………………………………………36

^{1}H NMR spectrum of **3h**……………………………………………………………………37

$^{13}C\{^{1}H\}$ NMR spectrum of **3h**…………………………………………………………37

HRMS spectrum of **3h**……………………………………………………………………………38

^{1}H NMR spectrum of **3i**……………………………………………………………………..39

^{13}C{^{1}H} NMR spectrum of **3i**……………………………………………………………….39

^{1}H NMR spectrum of **3j**……………………………………………………………………..40

^{13}C{^{1}H} NMR spectrum of **3j**……………………………………………………………….40

^{19}F NMR spectrum of **3j**……………………………………………………………………..41

HRMS spectrum of **3j**……………………………………………………………………………..42

^{1}H NMR spectrum of **3k**……………………………………………………………………..43

^{13}C{^{1}H} NMR spectrum of **3k**……………………………………………………………….43

^{19}F NMR spectrum of **3k**……………………………………………………………………..44

HRMS spectrum of **3k**……………………………………………………………………………..45

^{1}H NMR spectrum of **3l**……………………………………………………………………...46

^{13}C{^{1}H} NMR spectrum of **3l**………………………………………………………………..46

^{19}F NMR spectrum of **3l**……………………………………………………………………..47

HRMS spectrum of **3l**……………………………………………………………………………..48

^{1}H NMR spectrum of **3m**……………………………………………………………………..49

^{19}F NMR spectrum of **3m** ……………………………………………………………………..49

^{13}C{^{1}H} NMR spectrum of **3m** ……………………………………………………………...50

HRMS spectrum of **3m**……………………………………………………………………………..51

^{1}H NMR spectrum of **3n**……………………………………………………………………..52

^{13}C{^{1}H} NMR spectrum of **3n**……………………………………………………………….52

HRMS spectrum of **3n**……………………………………………………………………………..53

^{1}H NMR spectrum of **3o**……………………………………………………………………..54

^{13}C{^{1}H} NMR spectrum of **3o**……………………………………………………………….54

HRMS spectrum of **3o**……………………………………………………………………………..55

^{1}H NMR spectrum of **3p**……………………………………………………………………..56

^{13}C{^{1}H} NMR spectrum of **3p**……………………………………………………………….56

^{19}F NMR spectrum of **3p**……………………………………………………………………..57

HRMS spectrum of **3p**………………………………………………………………………..58

^{1}H NMR spectrum of **3q**……………………………………………………………………..59

$^{13}C\{^{1}H\}$ NMR spectrum of **3q**…………………………………………………………….59

HRMS spectrum of **3q**………………………………………………………………………….60

^{1}H NMR spectrum of **3r**……………………………………………………………………….61

$^{13}C\{^{1}H\}$ NMR spectrum of **3r**……………………………………………………………..61

HRMS spectrum of **3r**…………………………………………………………………………..62

^{1}H NMR spectrum of **3s**……………………………………………………………………….63

$^{13}C\{^{1}H\}$ NMR spectrum of **3s**……………………………………………………………...63

^{19}F NMR spectrum of **3s**……………………………………………………………………….64

HRMS spectrum of **3s**…………………………………………………………………………....65

^{1}H NMR spectrum of **4b**……………………………………………………………………….66

$^{13}C\{^{1}H\}$ NMR spectrum of **4b** ……………………………………………………………..66

HRMS spectrum of **4b** …………………………………………………………………………..67

^{1}H NMR spectrum of **4c**……………………………………………………………………….68

$^{13}C\{^{1}H\}$ NMR spectrum of **4c** ……………………………………………………………...68

HRMS spectrum of **4c** …………………………………………………………………………….69

^{1}H NMR spectrum of **4d**………………………………………………………………………….70

$^{13}C\{^{1}H\}$ NMR spectrum of **4d** ………………………………………………………………….70

HRMS spectrum of **4d** ………………………………………………………………………………71

^{1}H NMR spectrum of **4e**……………………………………………………………………………..72

^{19}F NMR spectrum of **4e**…………………………………………………………………………….72

$^{13}C\{^{1}H\}$ NMR spectrum of **4e** ……………………………………………………………………73

HRMS spectrum of **4e** ………………………………………………………………………………….74

^{1}H NMR spectrum of **4f**……………………………………………………………………………..75

$^{13}C\{^{1}H\}$ NMR spectrum of **4f** ……………………………………………………………………..75

HRMS spectrum of **4f** ……………………………………………………………………………..76

^{1}H NMR spectrum of **4g**……………………………………………………………………………77

^{13}C{^{1}H} NMR spectrum of **4g** ………………………………………………………………..77

HRMS spectrum of **4g** ……………………………………………………………………………..78

^{1}H NMR spectrum of **4h**……………………………………………………………………………79

^{13}C{^{1}H} NMR spectrum of **4h** ………………………………………………………………..79

HRMS spectrum of **4h** ……………………………………………………………………………..80

^{1}H NMR spectrum of **4i**……………………………………………………………………………81

^{13}C{^{1}H} NMR spectrum of **4i** ………………………………………………………………...81

HRMS spectrum of **4i** ……………………………………………………………………………...82

^{1}H NMR spectrum of **4j**……………………………………………………………………………83

^{13}C{^{1}H} NMR spectrum of **4j** ………………………………………………………………..83

^{1}H NMR spectrum of **4k**…………………………………………………………………………...84

^{13}C{^{1}H} NMR spectrum of **4k** …………………………………………………………………84

HRMS spectrum of **4k** ……………………………………………………………………………..85

7. References ……………………………………………………………………………………….86

1. General information

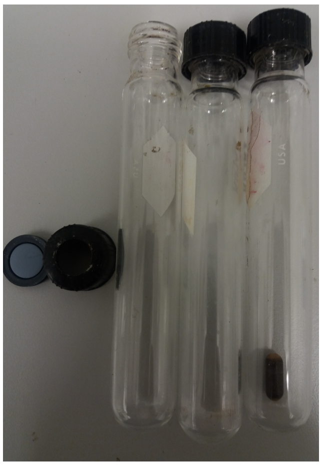

All the catalytic reactions were conducted under nitrogen atmosphere by using standard Schlenk technique. The solvents and chemicals were purchased from Aldrich and Chemtronica in Sweden. 1,2-dichloroethane and dichloromethane was distilled over calcium hydride under nitrogen atmosphere. All glassware's dried overnight at 120°C and if needed flame dried further. Column chromatography was performed on silica gel (60Å). Thin layer chromatography was performed on a silica gel precoated on aluminum foils with fluorescence indicator (254 nm). Yields refer to isolated compounds and 1H NMR determined their purity. The starting materials were purified by column chromatography on Silica gel (Carlo Erba, 60A) and using preparative TLC (Analtech, UV_{254} 20×20 cm, 500 micron) the products were purified. Nuclear magnetic resonance (NMR) spectroscopy was performed at 400 MHz (^{1}H NMR), 101 MHz (^{13}C NMR), and 376 MHz (^{19}F NMR) on Bruker Ascend 400 instrument. Chemical shifts (δ) are provided in ppm and spectra refered to non-deuterated solvent signal. Mass spectra (HRMS) were obtained from Lund University Kemi Centrum Mass Spectrometry facility. Instrument: Waters XEVO-G2 QTOF. ESI+: Capillary voltage3 kV, Cone voltage 35V, Ext 4, Source Temp 120, Des Temp 300, Cone gas 50, Des gas 400. Continuum resolution mode, m/z 100-1200, manual lock mass correction by Leucine Enkephalin (m/z 556.2771). Melting points were determined on a Stuart scientific micro melting point apparatus. Starting materials **1b**, **1c** and **2a** are commercially available and purchased from Aldrich.

2. Synthesis of starting materials

2.2. Synthesis of *N*-methyl indole

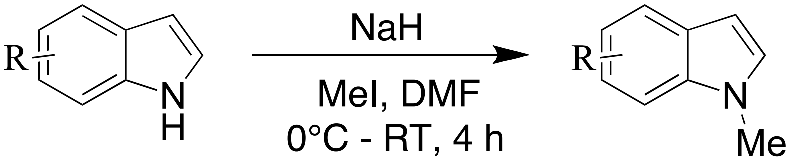

Using reported procedure modified slightly according to our purpose has done the N-methylation of indoles.[1]

2.3. 2,2-Dimethyl-1-(1-methyl-1H-indol-3-yl)propan-1-one

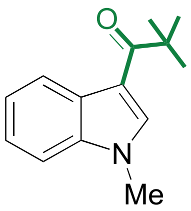

1d

This starting material was prepared according to the previously reported procedure.[2]

2.4. 1-Methyl-3-yl(tricyclo[3.3.1.13,7]dec-1-yl)methanone

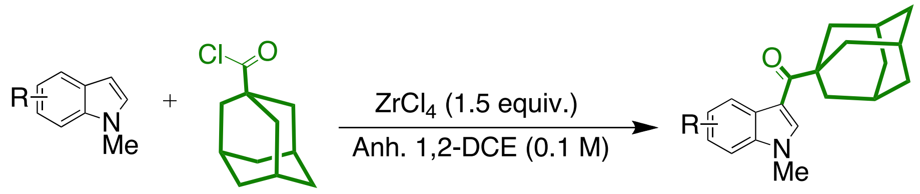

1.3 equiv. 1 equiv. 49-73 % yield

The starting materials were prepared according to the previously reported procedure.[2] Starting material **1a** was also prepared using this procedure

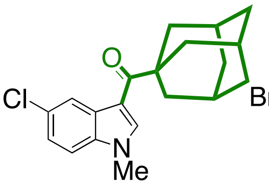

1e, 69%

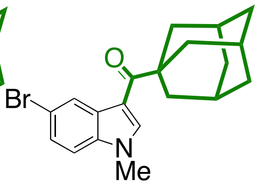

1f, 75%

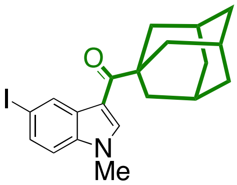

1g, 62%

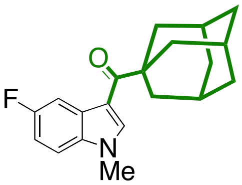

1h, 57%

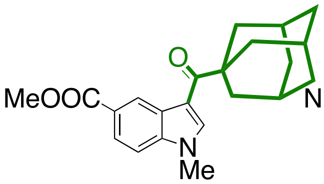

1i, 60%

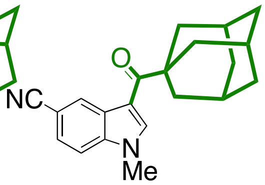

1j, 73%

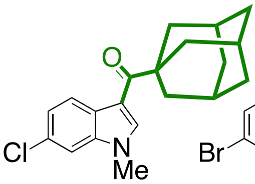

1k, 65%

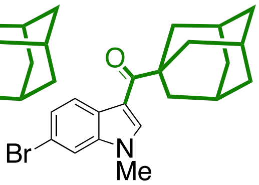

1l, 60%

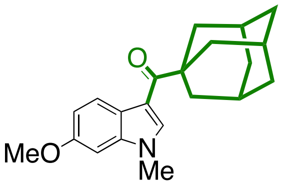

1m, 55%

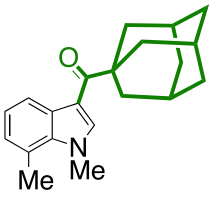

1n, 64%

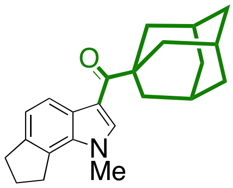

1o, 49%

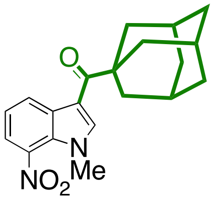

1p, 60%

2.5. Synthesis of disulfides

2.6. Table –S1: List of disulfides synthesized according to previously reported procedure[3]

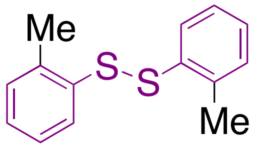
2b

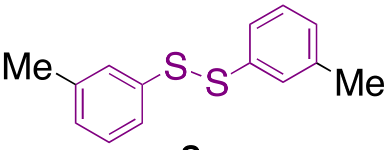
2c

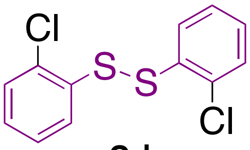
2d

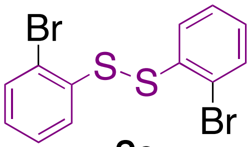
2e

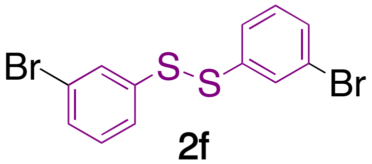
2f

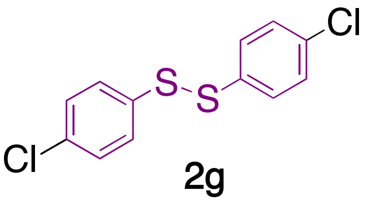
2g

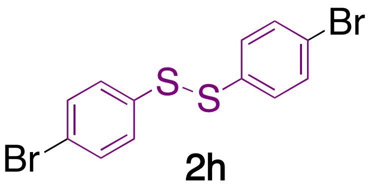
2h

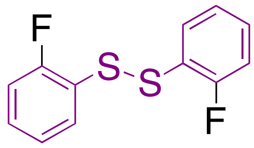
2i

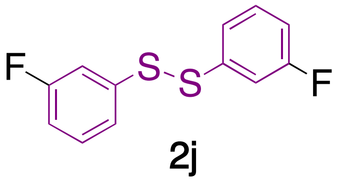
2j

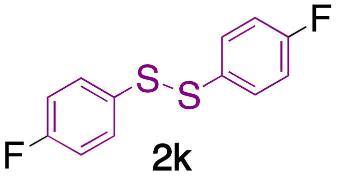
2k

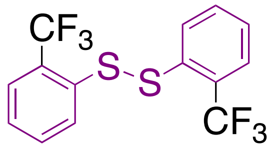
2l

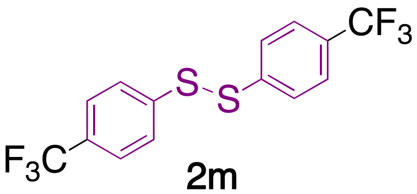
2m

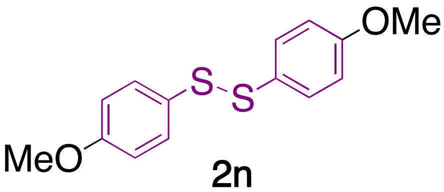
2n

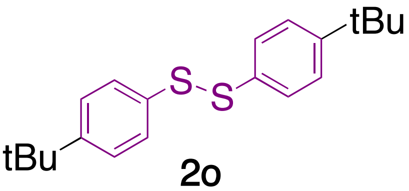
2o

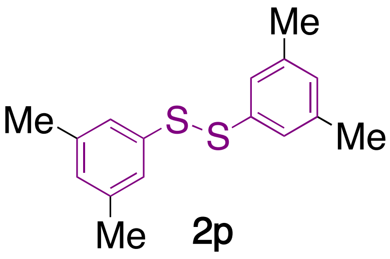
2p

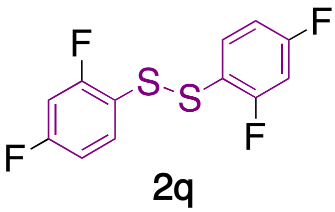
2q

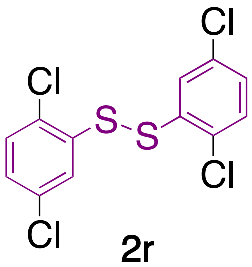
2r

2.7. Table -S2: List of commercially available alkyl disulfides used in this study

No product formation has been observed with the following alkyl/heteroaryl disulfies

Disulfide	CAS No.	Product description
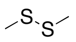	11258-26-4	TCI
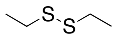	110-88-6	Aldrich
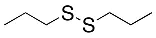	629-19-6	Aldrich
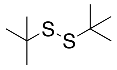	110-06-5	TCI
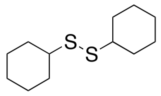	2550-40-5	TCI
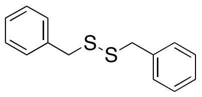	150-60-7	Alfa-Aesar
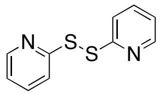	2127-03-9	Aldrich

3. Mechanistic studies

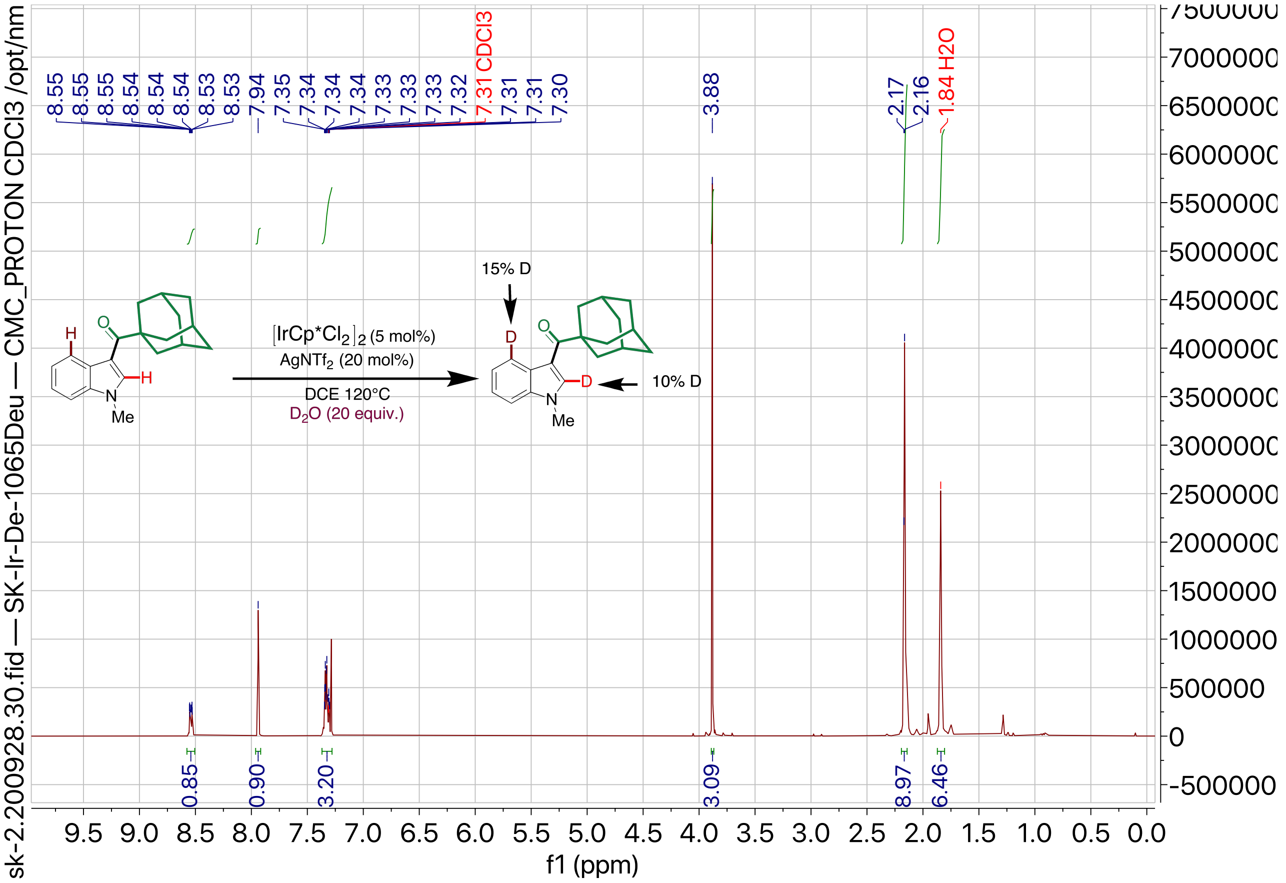

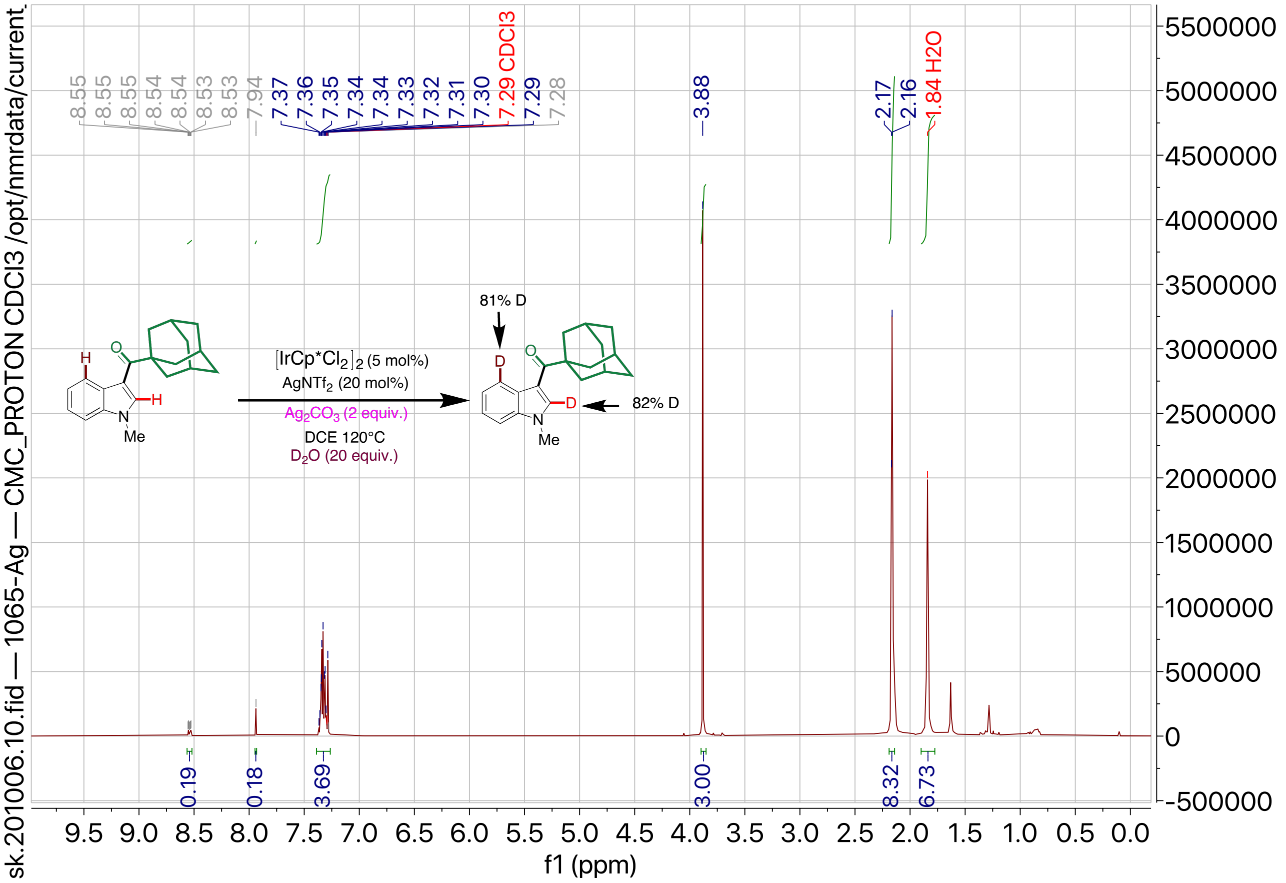
sk.201006.10.fid — 1065-Ag — CMC_PROTON CDCl3 /opt/nmrdata/current
[IrCp*Cl2]2 (5 mol%)
AgNTf2 (20 mol%)
Ag2CO3 (2 equiv.)
DCE 120°C
D2O (20 equiv.)
81% D
82% D
7.29 CDCl3
1.84 H2O
3.88
2.17
2.16
0.19
0.18
3.69
3.00
8.32
6.73
f1 (ppm)

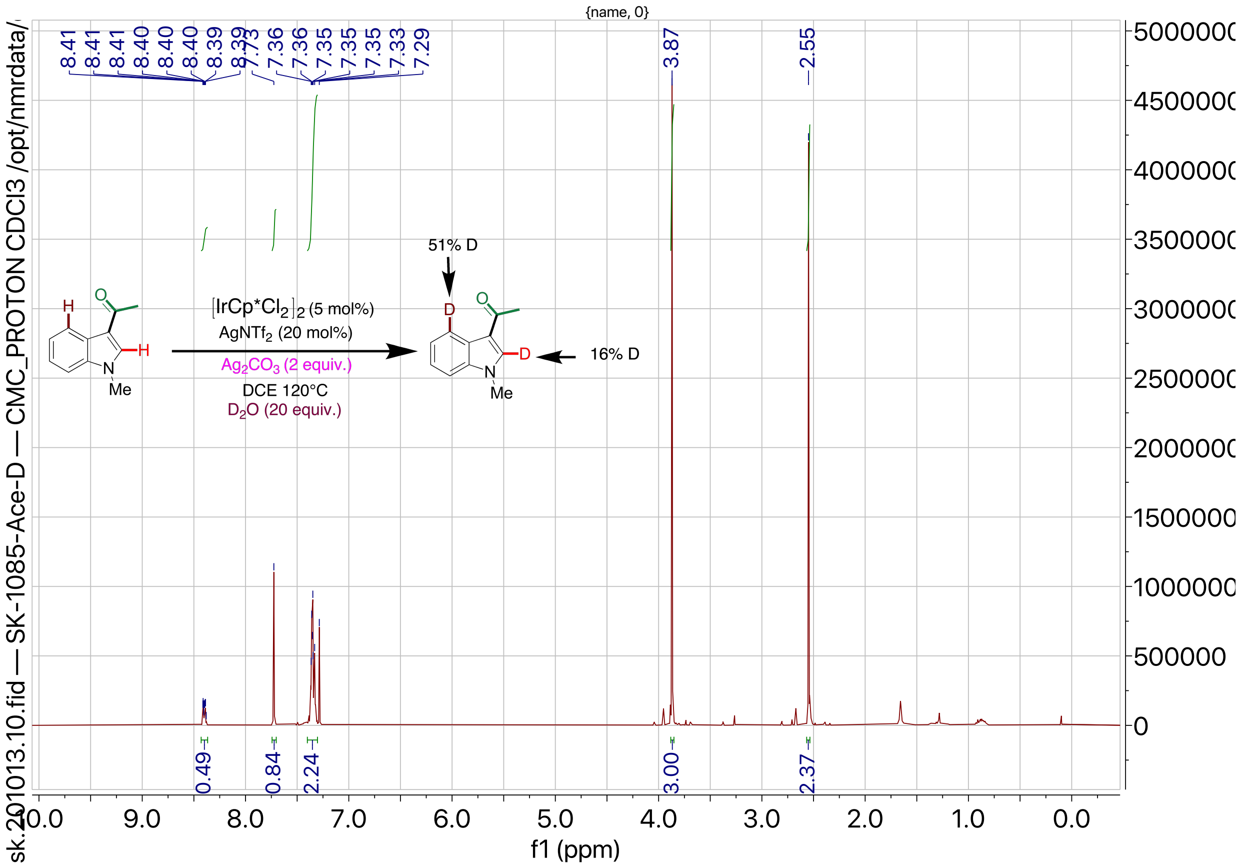
sk.201013.10.fid — SK-1085-Ace-D — CMC_PROTON CDCl3 /opt/nmrdata/
{name, 0}
[IrCp*Cl2]2 (5 mol%)
AgNTf2 (20 mol%)
Ag2CO3 (2 equiv.)
DCE 120°C
D2O (20 equiv.)
51% D
16% D
3.87
2.55
0.49
0.84
2.24
3.00
2.37
f1 (ppm)

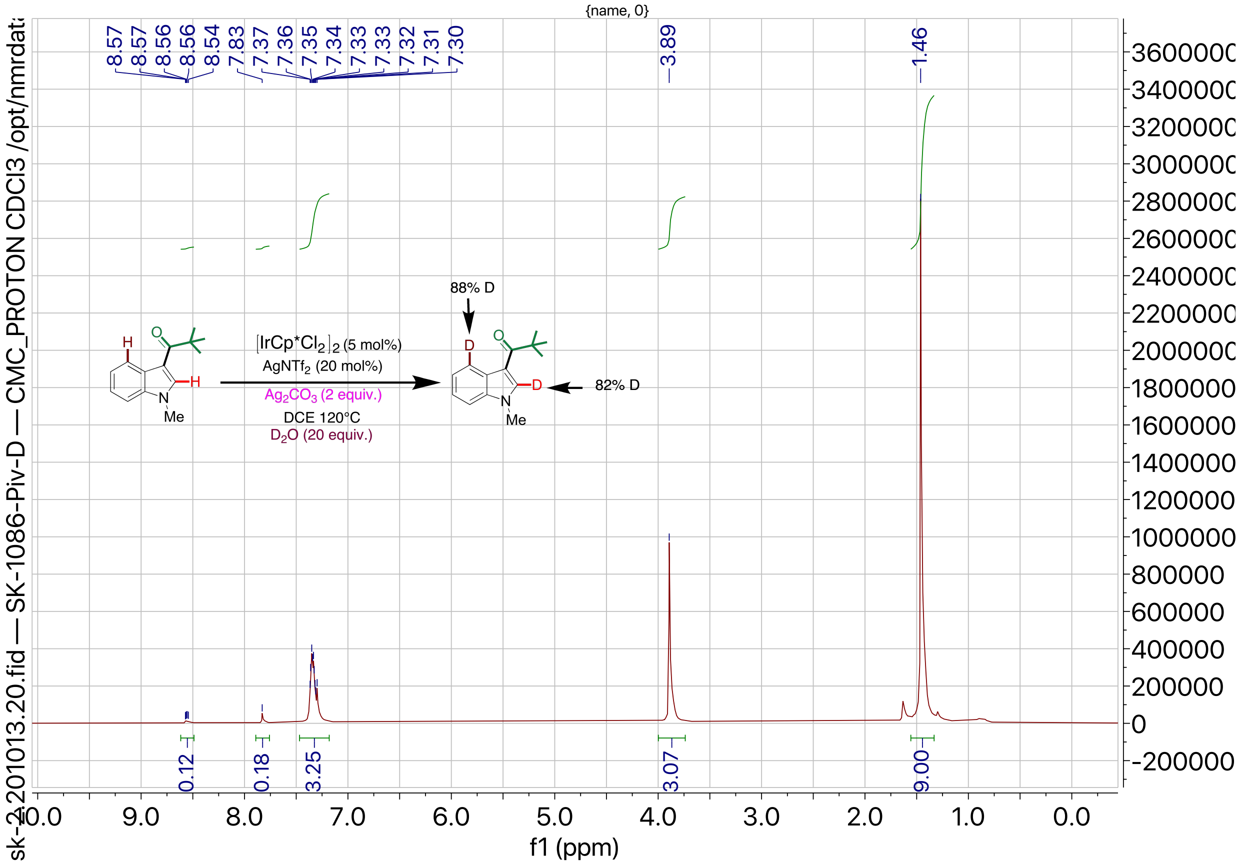

General procedure for the decarbonylative arylsulfenylation of indoles

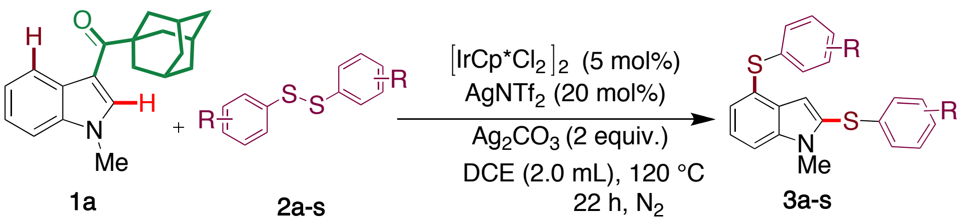

N-Methyl-3-adamantyl indole (**1a**) (50 mg, 0.17 mmol, 1 equiv.), aryl disulfide (**2a-s**) (0.25 mmol), $[IrCp^*Cl_2]_2$ (0.0045 mmol), $AgNTf_2$ (0.014 mmol), Ag_2CO_3 (0.65 mmol) were added to a reaction tube and the tube was fitted with Teflon capped screw cap. The tube was evacuated and filled with nitrogen for four times. Then 1,2-dichloroethane (2 mL) was added via syringe and the cap was sealed with thick layer of paraflim. The tube was transferred into a preheated aluminum block and continued until consumption of starting materials as shown by thin layer chromatography. The crude reaction mixture was allowed to reach room temperature followed by filtration through celite. Additionally the crude mixture was washed with acetone and methanol. The solvent was removed under vacuum and purified by preparative TLC. (Eleuent (v/v) – Petroelumether (100 mL)/acetone (4 mL). For final purification to get pure product we used Petroelumether (95 mL)/acetone (5 mL).

General procedure for the decarbonylative arylsulfenylation of indoles (4)

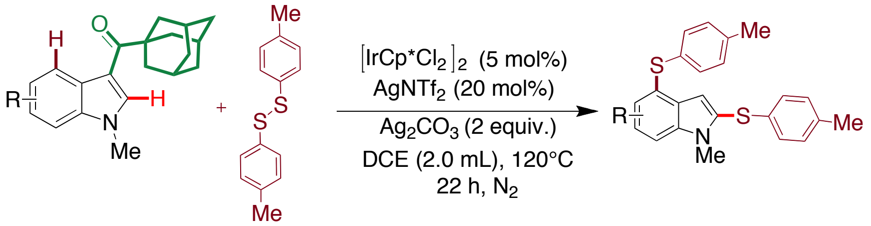

N-Methyl-3-adamantyl indole (**1b-k**) (50 mg, 0.17 mmol, 1 equiv.), aryl disulfide (**2d**) (0.25 mmol), $[IrCp*Cl_2]_2$ (0.0045 mmol), $AgNTf_2$ (0.014 mmol), Ag_2CO_3 (0.65 mmol) were added to a reaction tube and the tube was fitted with Teflon capped screw cap. The tube was evacuated and filled with nitrogen for four times. Then 1,2-dichloroethane (2 mL) was added via syringe and the cap was sealed with thick layer of paraflim. The tube was transferred into a preheated aluminum block and continued until consumption of starting materials as shown by thin layer chromatography. The crude reaction mixture was allowed to reach room temperature followed by filtration through celite. Additionally the crude mixture was washed with acetone and methanol. The solvent was removed under vacuum and purified by preparative TLC (Eleuent (v/v) – Petroelumether/acetone (100 mL/4 mL). For final purification to get pure product we used Petroelumether (95 mL)/acetone (5mL).

General procedure for the decarbonylative arylsulfenylation of indole at 1mmol scale

N-Methyl-3-adamantyl indole (**1a**) (0.35g, 1,19 mmol, 1 equiv.), aryl disulfide (**2a**) (2.38 mmol), $[IrCp*Cl_2]_2$ (0.059 mmol), $AgNTf_2$ (0.36 mmol), Ag_2CO_3 (2.38 mmol) were added to a reaction tube and the tube was fitted with Teflon capped screw cap. The tube was evacuated and filled with nitrogen for four times. Then 1,2-dichloroethane (5 mL) was added via syringe and the cap was sealed with thick layer of paraflim. The tube was transferred into a preheated aluminum block and continued until consumption of starting materials as shown by thin layer chromatography. The crude reaction mixture was allowed to reach room temperature followed by filtration through celite. Additionally the crude mixture was washed with acetone and methanol. The solvent was removed under vacuum and purified by column chromatography (Eleuent (v/v) – Petroelumether(100 mL) /acetone (2 mL).

5. Characterization Data

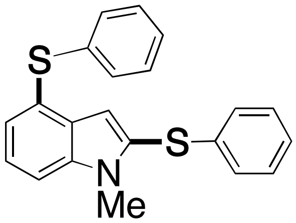

3a

Yellow Solid, m.pt: 90-92 °C. 40 mg, 68%. ^{1}H NMR (400 MHz, Chloroform-*d*) δ 7.59 (d, *J* = 8.0 Hz, 1H), 7.34 – 7.23 (m, 2H), 7.14 – 7.06 (m, 4H), 7.06 – 7.00 (m, 5H), 6.99 – 6.93 (m, 3H), 3.72 (s, 3H). ^{13}C NMR (101 MHz, $CDCl_3$) δ 137.4, 134.8, 133.2, 128.1, 127.6, 126.3, 125.5, 125.1, 123.9, 122.9, 120.0, 119.3, 110.0, 109.15, 30.0.

HRMS (ESI) *m/z*: $[M+Na]^+$calcd for $C_{21}H_{17}NS_2Na$ 370.0695; Found: 370.0700

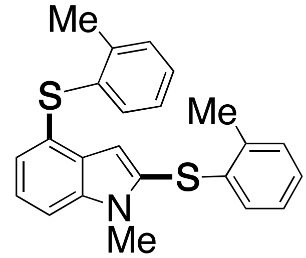

3b

Yellow Solid, m.pt: 145-147 °C. 22 mg, 34%. ^{1}H NMR (400 MHz, Chloroform-*d*) δ 7.53 (d, *J* = 8.0 Hz, 1H), 7.34 (d, *J* = 8.3 Hz, 1H), 7.31 – 7.23 (m, 1H), 7.10 (td, *J* = 7.4, 6.9, 1.1 Hz, 1H), 7.03 (dd, *J* = 13.3, 7.3 Hz, 3H), 6.96 – 6.90 (m, 1H), 6.90 – 6.83 (m, 2H), 6.79 (qd, *J* = 7.8, 1.3 Hz, 2H), 6.62 (dd, *J* = 7.9, 1.4 Hz, 1H), 6.47 (dd, *J* = 7.9, 1.3 Hz, 1H), 3.68 (s, 3H), 2.40 (s, 4H), 2.38 (s, 3H). ^{13}C NMR (101 MHz, $CDCl_3$) δ 137.7, 136.5, 134.2, 134.2, 133.7, 129.2, 128.8, 128.2, 125.7, 125.4, 125.1, 124.8, 124.7, 123.5, 122.8, 119.9, 119.3, 109.1, 30.0, 28.6, 28.0. HRMS (ESI) *m/z*: $[M+Na]^+$ calcd for $C_{23}H_{21}NS_2Na$ 398.1015; Found: 398.1013

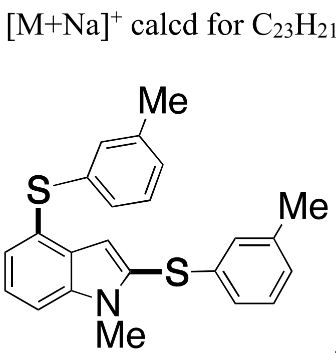

3c

Yellow Solid, m.pt: 101-103 °C. 32 mg, 50%. ^{1}H NMR (400 MHz, Chloroform-*d*) δ 7.61 (d, *J* = 8.0 Hz, 1H), 7.34 – 7.23 (m, 2H), 7.11 (ddd, *J* = 8.0, 6.9, 1.2 Hz, 1H), 6.98 (t, *J* = 7.7 Hz, 1H), 6.96 – 6.87 (m, 3H), 6.87 – 6.74 (m, 6H), 3.73 (s, 3H), 2.11 (s, 6H). ^{13}C NMR (101 MHz, $CDCl_3$) δ 138.0, 137.4, 137.3, 137.2, 134.5,

133.5, 128.2, 127.9, 127.4, 127.1, 126.2, 126.1, 124.9, 123.6, 122.8, 122.7, 119.9, 119.3, 110.0, 109.1, 30.1, 20.3, 20.2. HRMS (ESI) *m/z*: $[M+Na]^+$ calcd for $C_{23}H_{21}NS_2Na$ 398.1013; Found: 398.1013

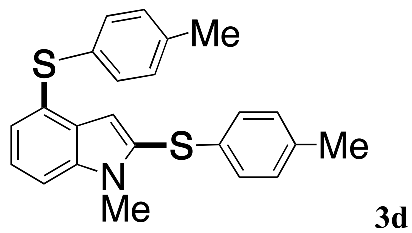

3d

Orange Solid, m.pt: 50-52 °C. 32 mg, 55%. ^1H NMR (400 MHz, Methanol-d_4) δ 7.43 (d, J = 8.0 Hz, 1H), 7.34 (d, J = 8.3 Hz, 1H), 7.18 (ddd, J = 8.3, 7.1, 1.2 Hz, 1H), 7.04 – 6.98 (m, 1H), 6.88 (d, J = 8.1 Hz, 2H), 6.81 (d, J = 7.5 Hz, 6H), 3.66 (s, 3H), 2.12 (s, 3H), 2.10 (s, 3H). ^{13}C NMR (101 MHz, Chloroform) δ 138.4, 136.1, 134.9, 134.6, 134.5, 132.1, 129.5, 129.0, 128.9, 127.5, 126.6, 123.5, 120.5, 119.4, 110.9, 110.0, 36.5, 29.9, 19.5, 19.4. HRMS (ESI) *m/z*: $[M+Na]^+$ calcd for $C_{23}H_{21}NS_2Na$ 398.1011; Found: 398.1013

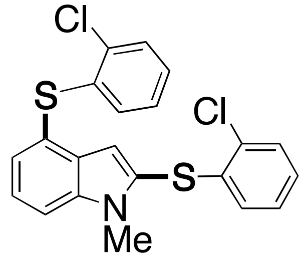

3e

Yellow Solid, m.pt: 115-117 °C. 30 mg, 42%. ^1H NMR (400 MHz, Chloroform-d) δ 7.67 (d, J = 8.0 Hz, 1H), 7.49 (dt, J = 8.4, 1.0 Hz, 1H), 7.42 (ddd, J = 8.3, 6.9, 1.2 Hz, 1H), 7.37 – 7.23 (m, 4H), 7.06 (td, J = 7.7, 1.6 Hz, 1H), 6.99 (tdd, J = 7.8, 3.6, 1.6 Hz, 2H), 6.92 (td, J = 7.6, 1.5 Hz, 1H), 6.61 (ddd, J = 22.6, 7.9, 1.6 Hz, 2H), 3.86 (s, 3H). ^{13}C NMR (101 MHz, $CDCl_3$) δ 138.8, 137.5, 135.1, 133.4, 131.3, 130.5, 129.7, 129.3, 129.1, 127.7, 127.4, 126.9, 126.8, 126.6, 125.6, 124.4, 121.4, 120.3, 110.4, 110.1, 77.3, 77.0, 76.7, 31.1. HRMS (ESI) *m/z*: $[M+Na]^+$ calcd for $C_{23}H_{15}Cl_2NS_2Na$ 437.9920; Found: 437.9920

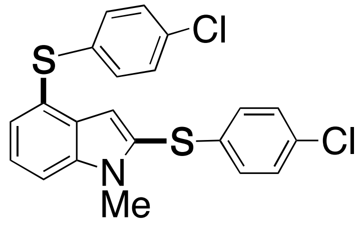

3f

Yellow Solid, m.pt: 146-148 °C. 27 mg, 38%. ^1H NMR (400 MHz, Chloroform-d) δ 7.67 (d, J = 8.0 Hz, 1H), 7.47 – 7.37 (m, 2H), 7.26 (dd, J = 15.3, 7.2 Hz, 1H), 7.17 (d, J = 8.6 Hz, 2H), 7.11 (d, J = 8.6 Hz, 2H), 7.00

(dd, J = 15.5, 8.6 Hz, 4H), 3.84 (s, 3H). ^{13}C NMR (101 MHz, $CDCl_3$) δ 138.5, 136.9, 134.2, 133.8, 132.4, 130.9, 129.3, 129.0, 128.8, 128.7, 127.8, 124.3, 121.4, 120.2, 110.9, 110.4, 31.1. HRMS (ESI) *m/z*: $[M+Na]^+$ calcd for $C_{21}H_{15}Cl_2NS_2Na$ 437.9921; found 437.9920.

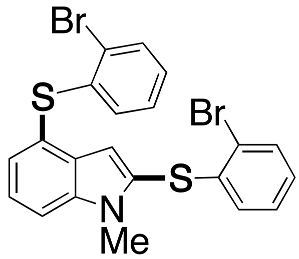

3g

Yellow sticky solid, 34 mg, 40%. ^{1}H NMR (400 MHz, Chloroform-*d*) δ 7.67 (d, J = 8.0 Hz, 1H), 7.54 – 7.46 (m, 3H), 7.46 – 7.38 (m, 1H), 7.25 (ddd, J = 8.0, 6.9, 1.1 Hz, 1H), 7.05 (td, J = 7.6, 1.5 Hz, 1H), 7.02 – 6.94 (m, 2H), 6.91 (td, J = 7.5, 1.7 Hz, 1H), 6.61 (dd, J = 7.8, 1.7 Hz, 1H), 6.54 (dd, J = 7.8, 1.7 Hz, 1H), 3.85 (s, 3H). ^{13}C NMR (101 MHz, $CDCl_3$) δ 139.5, 138.8, 137.2, 133.9, 133.0, 132.6, 129.0, 128.0, 127.6, 127.4, 127.1, 126.6, 125.8, 124.4, 121.4, 120.8, 120.4, 120.1, 110.7, 110.4, 31.2. HRMS (ESI) *m/z*: $[M+Na]^+$ calcd for $C_{21}H_{15}Br_2NS_2Na$ 525.8903; Found: 525.8910.

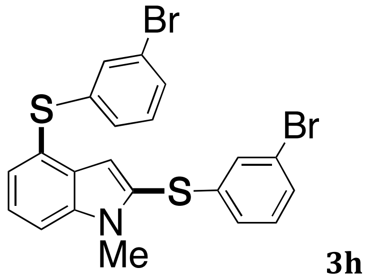

3h

Yellow sticky solid, 44 mg, 51%. ^{1}H NMR (400 MHz, Chloroform-*d*) δ 7.70 (d, J = 8.0 Hz, 1H), 7.48 – 7.39 (m, 2H), 7.30 – 7.23 (m, 2H), 7.22 – 7.16 (m, 3H), 7.06 (t, J = 7.9 Hz, 1H), 7.01 (d, J = 4.9 Hz, 2H), 6.95 (ddd, J = 7.9, 1.9, 1.0 Hz, 1H), 3.87 (s, 3H). ^{13}C NMR (101 MHz, $CDCl_3$) δ 140.8, 138.5, 137.9, 133.5, 130.5, 130.0, 129.8, 129.51, 129.1, 128.9, 128.2, 125.8, 125.0, 124.4, 123.2, 122.8, 121.5, 120.2, 110.5, 110.5, 31.2. HRMS (ESI) *m/z*: $[M+Na]^+$ calcd for $C_{21}H_{15}Br_2NS_2Na$ 527.8890; Found: 527.8888.

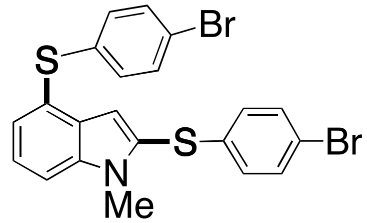

3i

Yellow Solid, m.pt: 96-98 °C, 65 mg, 70%. ^{1}H NMR (400 MHz, Chloroform-*d*) δ 7.56 (d, *J* = 7.9 Hz, 1H), 7.37 – 7.26 (m, 2H), 7.23 – 7.12 (m, 6H), 6.82 (dd, *J* = 16.4, 8.6 Hz, 4H), 3.74 (s, 3H). ^{13}C NMR (101 MHz, $CDCl_3$) δ 137.4, 136.6, 133.8, 132.6, 131.2, 130.6, 127.9, 127.8, 127.0, 123.3, 120.4, 119.2, 117.7, 109.7, 109.3, 76.3, 75.9, 75.6, 30.1, 28.6. HRMS (ESI) *m/z*: $[M+Na]^+$ calcd for $C_{21}H_{15}Br_2NS_2Na$ 528.2793; Found: 528.1493

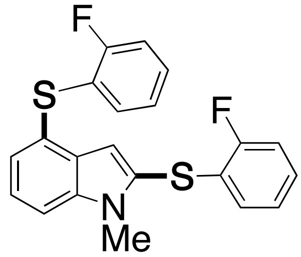

3j

Yellow Solid, m.pt: 80-82 °C, 44 mg, 68%. ^{1}H NMR (400 MHz, Chloroform-*d*) δ 7.58 (d, *J* = 8.0 Hz, 1H), 7.36 – 7.25 (m, 2H), 7.12 (ddd, *J* = 8.0, 6.9, 1.1 Hz, 1H), 7.01 (dddd, *J* = 8.9, 7.2, 5.1, 1.8 Hz, 1H), 6.95 – 6.85 (m, 3H), 6.80 – 6.65 (m, 3H), 6.60 (td, *J* = 7.8, 1.7 Hz, 1H), 3.78 (s, 3H). ^{13}C NMR (101 MHz, $CDCl_3$) δ 159.8, 159.4, 157.4, 157.0, 137.4, 129.1, 129.1, 128.2, 127.5, 127.4, 127.2, 127.1, 125.5, 125.4, 123.7, 123.6, 123.1, 123.1, 120.2, 119.1, 114.7, 114.5, 114.2, 114.0, 109.3, 30.1. ^{19}F NMR (376 MHz, $CDCl_3$) δ -111.44, -111.45, -111.46, -111.47, -111.48, -111.48, -111.49, -112.76, -112.78, -112.79, -112.80, -112.81, -112.81, -112.82. HRMS (ESI) *m/z*: $[M+Na]^+$ calcd for $C_{21}H_{15}F_2NS_2Na$ 406.0503; Found: 406.0512.

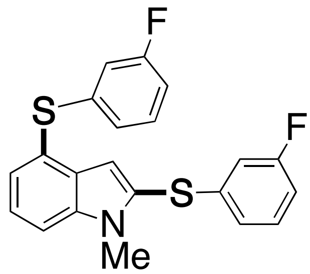

3k

Yellow Solid, m.pt: 65-67 °C, 35 mg, 53%. ^{1}H NMR (400 MHz, Chloroform-*d*) δ 7.59 (d, *J* = 8.0 Hz, 1H), 7.39 – 7.27 (m, 2H), 7.19 – 7.12 (m, 1H), 7.04 (dtd, *J* = 21.9, 8.0, 5.9 Hz, 2H), 6.82 – 6.78 (m, 1H), 6.76 –

6.69 (m, 2H), 6.67 – 6.59 (m, 3H), 3.76 (s, 3H). ^{19}F NMR (376 MHz, $CDCl_3$) δ -111.37, -112.62. ^{13}C NMR (101 MHz, $CDCl_3$) δ 163.2, 163.1, 160.7, 160.7, 140.0, 139.9, 137.5, 137.0, 137.0, 132.4, 129.5, 129.4, 128.9, 128.8, 128.0, 123.4, 121.6, 121.6, 120.8, 120.8, 120.4, 119.2, 113.1, 112.9, 112.3, 112.2, 112.1, 112.0, 111.0, 110.8, 109.5, 109.4, 30.1. HRMS (ESI) *m/z*: $[M+Na]^+$ calcd for $C_{21}H_1F_2NS_2Na$ 406.0512; Found: 406.0509.

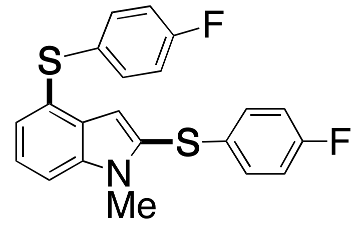

3l

Brown sticky solid, 42 mg, 64%. ^{1}H NMR (400 MHz, Chloroform-*d*) δ 7.69 (d, *J* = 8.0 Hz, 1H), 7.46 – 7.33 (m, 2H), 7.23 (ddd, *J* = 8.0, 6.7, 1.3 Hz, 1H), 7.09 (ddd, *J* = 14.0, 8.9, 5.1 Hz, 4H), 6.95 – 6.82 (m, 4H), 3.84 (s, 3H). ^{19}F NMR (376 MHz, $CDCl_3$) δ -115.34, -117.52. ^{13}C NMR (101 MHz, $CDCl_3$) δ 162.9, 162.3, 160.4, 159.8, 138.3, 134.5, 133.3, 129.9, 129.8, 129.0, 128.9, 128.8, 124.1, 121.2, 120.2, 116.4, 116.2, 115.8, 115.6, 111.5, 110.2, 31.1. HRMS (ESI) *m/z*: $[M+Na]^+$ calcd for $C_{21}H_1F_2NS_2Na$ 406.4671; Found: 406.0501.

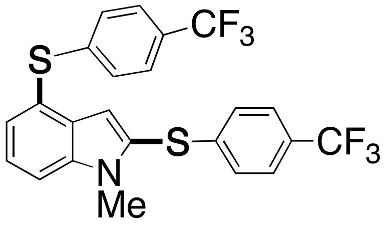

3m

Yellow Solid, m.pt: 56-58 °C, 36 mg, 43%. ^{1}H NMR (400 MHz, Chloroform-*d*) δ 7.57 (d, *J* = 8.0 Hz, 1H), 7.41 – 7.36 (m, 1H), 7.32 (d, *J* = 8.7 Hz, 3H), 7.26 (d, *J* = 8.3 Hz, 2H), 7.20 – 7.14 (m, 1H), 6.99 (dd, *J* = 16.0, 8.2 Hz, 4H), 3.77 (s, 3H). ^{19}F NMR (376 MHz, $CDCl_3$) δ -62.37, -62.59. ^{13}C NMR (101 MHz, $CDCl_3$) δ 142.5, 142.5, 139.8, 139.8, 137.6, 131.8, 127.9, 125.5, 125.0, 125.0, 125.0, 124.9, 124.8, 124.5, 124.5, 124.4, 124.4, 123.6, 120.7, 119.2, 109.5, 109.1, 30.2. HRMS (ESI) *m/z*: $[M+H]^+$ calcd for $C_{23}H_{15}F_6NS_2H$ 484.0630; Found: 484.0628.

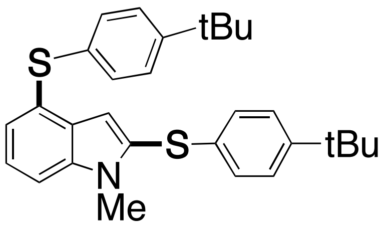

3n

Yellow Solid, m.pt: 54-56 °C, 29 mg, 37%. H NMR (400 MHz, Chloroform-*d*) δ 7.62 (d, *J* = 7.9 Hz, 1H), 7.32 – 7.21 (m, 2H), 7.13 – 7.05 (m, 5H), 6.98 (d, *J* = 8.6 Hz, 2H), 6.92 (d, *J* = 8.5 Hz, 2H), 3.72 (s, 3H), 1.16 (d, *J* = 2.0 Hz, 18H). ^{13}C NMR (101 MHz, $CDCl_3$) δ 148.4, 147.0, 137.3, 134.0, 133.7, 131.2, 128.2, 126.5, 125.6, 125.1, 124.6, 122.7, 119.8, 119.3, 110.3, 109.0, 33.3, 33.2, 30.2, 30.1, 28.6. HRMS (ESI) *m/z*: $[M+Na]^+$ calcd for $C_{29}H_{33}NS_2Na$ 482.1951; Found: 482.1952.

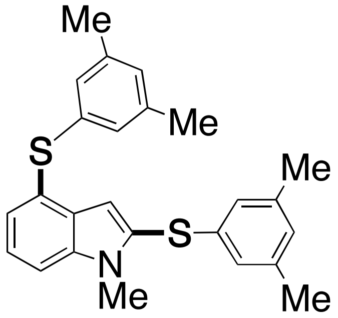

3o

Yellow Sticky solid, 41 mg, 60%. ^1H NMR (400 MHz, Chloroform-*d*) δ 7.62 (d, *J* = 7.9 Hz, 1H), 7.34 – 7.29 (m, 1H), 7.29 – 7.23 (m, 1H), 7.11 (ddd, *J* = 8.0, 6.9, 1.1 Hz, 1H), 6.71 – 6.56 (m, 7H), 3.73 (s, 3H), 2.08 (s, 6H), 2.06 (s, 6H). ^{13}C NMR (101 MHz, $CDCl_3$) δ 137.8, 137.4, 137.1, 137.0, 134.2, 133.6, 128.3, 127.1, 126.0, 124.3, 123.4, 122.6, 119.8, 119.4, 109.0, 30.1, 20.2, 20.1. HRMS (ESI) *m/z*: $[M+Na]^+$ calcd for $C_{25}H_{25}NS_2Na$ 426.1324; Found: 426.1326.

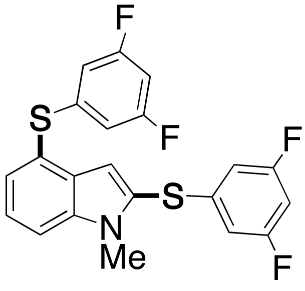

3p

Yellow sticky solid, 34 mg, 48%. ^1H NMR (400 MHz, Chloroform-*d*) δ 7.69 (d, *J* = 8.0 Hz, 1H), 7.49 – 7.38 (m, 2H), 7.30 – 7.24 (m, 1H), 7.06 – 6.92 (m, 2H), 6.90 – 6.78 (m, 4H), 3.88 (s, 3H). ^{13}C NMR (101 MHz, $CDCl_3$) δ 151.9, 151.7, 151.7, 151.5, 150.5, 150.4, 149.7, 149.6, 149.3, 149.2, 149.2, 149.0, 148.0, 147.9, 147.3, 147.2, 138.5, 134.8, 134.7, 134.7, 134.7, 133.7, 131.8, 131.8, 131.7, 128.9, 124.6, 123.8, 123.7, 123.7, 123.7, 122.7, 122.7, 122.6, 122.6, 121.6, 120.1, 118.1, 117.9, 117.5, 117.3, 116.9, 116.7, 115.8, 115.6, 110.9, 110.5, 31.2. ^{19}F NMR (376 MHz, $CDCl_3$) δ -135.34, -136.71, -136.76, -136.79, -139.53, -141.91. HRMS (ESI) *m/z*: $[M+Na]^+$ calcd for $C_{21}H_{13}F_4NS_2Na$ 442.0323; Found: 442.0318.

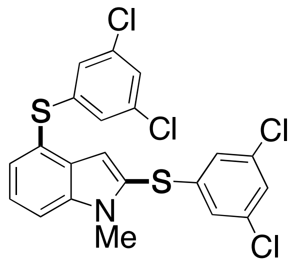
3q

Yellow sticky solid, 50 mg, 60%. ^{1}H NMR (400 MHz, Chloroform-*d*) δ 7.67 (d, *J* = 8.0 Hz, 0H), 7.56 – 7.49 (m, 1H), 7.46 (ddd, *J* = 8.3, 7.0, 1.2 Hz, 1H), 7.34 – 7.19 (m, 3H), 7.01 (dd, *J* = 8.5, 2.4 Hz, 1H), 6.94 (dd, *J* = 8.4, 2.4 Hz, 1H), 6.63 (d, *J* = 2.4 Hz, 1H), 6.50 (d, *J* = 2.4 Hz, 1H), 3.93 (s, 3H). ^{13}C NMR (101 MHz, $CDCl_3$) δ 139.3, 138.8, 136.3, 133.5, 133.0, 132.8, 130.6, 130.2, 130.1, 129.0, 128.8, 127.8, 127.4, 125.9, 125.8, 124.7, 121.8, 120.1, 110.7, 109.1, 31.3. HRMS (ESI) *m/z*: $[M+Na]^+$ calcd for $C_{21}H_{13}Cl_4NS_2Na$ 505.9128; Found: 505.9141.

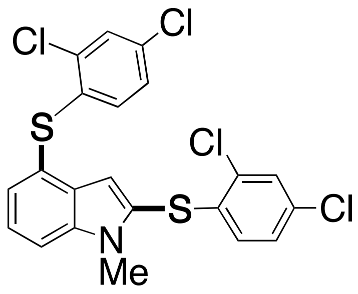
3r

Yellow sticky solid, 46 mg, 56%. ^{1}H NMR (400 MHz, Chloroform-*d*) δ 7.64 (d, *J* = 8.0 Hz, 1H), 7.50 (dt, *J* = 8.4, 1.0 Hz, 1H), 7.43 (ddd, *J* = 8.3, 6.9, 1.2 Hz, 1H), 7.33 (dd, *J* = 4.7, 2.2 Hz, 2H), 7.30 – 7.22 (m, 1H), 6.97 (dd, *J* = 8.6, 2.2 Hz, 1H), 6.87 (dd, *J* = 8.6, 2.2 Hz, 1H), 6.56 (d, *J* = 8.5 Hz, 1H), 6.50 (d, *J* = 8.6 Hz, 1H), 3.88 (s, 3H). ^{13}C NMR (101 MHz, $CDCl_3$) δ 138.7, 136.1, 133.3, 133.1, 132.6, 132.4, 131.1, 130.8, 129.6, 129.1, 129.1, 129.0, 127.6, 127.5, 127.0, 124.6, 121.7, 120.1, 110.6, 109.5, 31.2. HRMS (ESI) *m/z*: $[M+Na]^+$ calcd for $C_{21}H_{13}Cl_4NS_2Na$ 507.9112; Found: 507.9108.

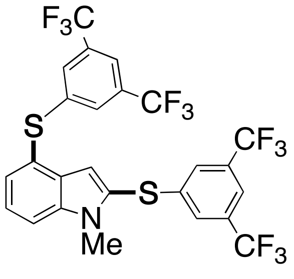
3s

Yellow Solid, m.pt: 112-114 °C, 63 mg, 60%. ^{1}H NMR (400 MHz, Chloroform-*d*) δ 7.67 – 7.63 (m, 2H), 7.57 – 7.54 (m, 1H), 7.53 (t, *J* = 1.0 Hz, 1H), 7.49 (dd, *J* = 6.9, 1.2 Hz, 1H), 7.47 – 7.43 (m, 4H), 7.32 (ddd, *J* = 8.0,

6.9, 1.1 Hz, 1H), 3.94 (s, 3H). ^{13}C NMR (101 MHz, $CDCl_3$) δ 141.7, 139.1, 138.8, 132.9, 132.5, 132.2, 131.9, 131.6, 128.6, 126.7, 125.7, 125.3, 124.2, 124.0, 122.2, 121.5, 121.2, 120.3, 120.0, 119.1, 119.0, 110.9, 109.5, 31.4. ^{19}F NMR (376 MHz, $CDCl_3$) δ -63.04, -63.06, -63.22, -63.23. HRMS (ESI) *m/z*: $[M+H]^+$ calcd for $C_{25}H_{13}F_{12}NS_2H$ 620.0378; Found: 620.0376.

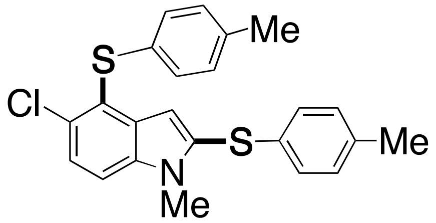

4b

Yellow Solid, m.pt: 156-158 °C, 31 mg, 50%. ^1H NMR (400 MHz, Chloroform-*d*) δ 7.56 (dd, *J* = 1.7, 0.9 Hz, 1H), 7.22 – 7.15 (m, 3H), 6.90 (qd, *J* = 8.2, 5.2 Hz, 8H), 3.68 (s, 3H), 2.18 (s, 3H), 2.17 (s, 3H). ^{13}C NMR (101 MHz, $CDCl_3$) δ 135.6, 135.5, 135.4, 134.0, 133.4, 130.6, 129.2, 128.9, 128.5, 127.1, 126.0, 125.9, 123.1, 118.5, 110.1, 109.9, 30.2, 28.6, 19.9, 19.8. HRMS (ESI) *m/z*: $[M+H]^+$ calcd for $C_{23}H_{20}ClNS_2Na$ 432.0622; Found: 432.0623.

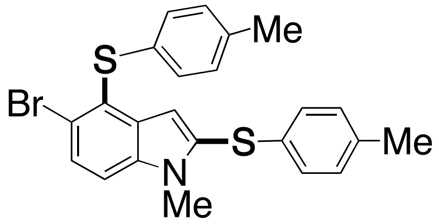

4c

Yellow Solid, m.pt: 80-82 °C, 33 mg, 54%. ^1H NMR (400 MHz, Chloroform-*d*) δ 7.73 (d, *J* = 1.9 Hz, 1H), 7.31 (dd, *J* = 8.7, 1.9 Hz, 1H), 7.19 – 7.12 (m, 2H), 6.95 – 6.85 (m, 9H), 3.67 (s, 3H), 2.18 (s, 3H), 2.17 (s, 3H). ^{13}C NMR (101 MHz, $CDCl_3$) δ 135.9, 135.5, 135.4, 134.1, 133.4, 130.5, 129.8, 128.9, 128.5, 127.1, 126.0, 125.7, 121.6, 113.4, 110.5, 109.8, 30.2, 19.9, 19.8. HRMS (ESI) *m/z*: $[M+Na]^+$ calcd for $C_{23}H_{20}BrNS_2Na$ 476.0113; Found: 476.0118.

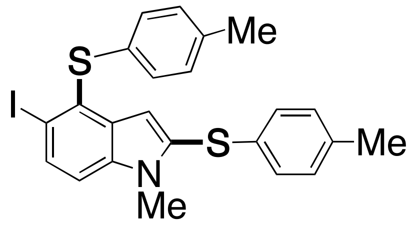

4d

Yellow sticky solid, 35 mg, 60%. ^1H NMR (400 MHz, Chloroform-*d*) δ 7.94 (d, *J* = 1.7 Hz, 1H), 7.48 (dd, *J* = 8.6, 1.7 Hz, 1H), 7.05 (d, *J* = 8.6 Hz, 1H), 6.94 – 6.85 (m, 9H), 3.66 (s, 3H), 2.18 (s, 3H), 2.17 (s, 3H). ^{13}C

NMR (101 MHz, $CDCl_3$) δ 136.3, 135.5, 135.1, 134.0, 133.4, 131.1, 130.5, 130.5, 128.9, 128.5, 127.9, 127.1, 126.0, 110.9, 30.1, 19.9, 19.8. HRMS (ESI) *m/z*: $[M+Na]^+$ calcd for $C_{23}H_{20}INS_2H$ 502.0155; Found: 502.01600.

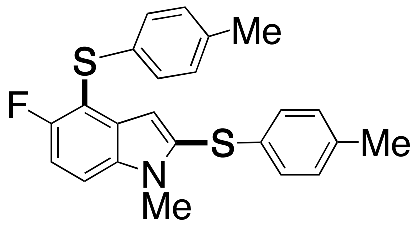

4e

Yellow Solid, m.pt: 107-109 °C, 48 mg, 76%. ^1H NMR (400 MHz, Chloroform-*d*) δ 7.24 – 7.20 (m, 2H), 7.20 – 7.16 (m, 1H), 7.01 – 6.84 (m, 11H), 3.69 (s, 4H), 2.18 (s, 3H), 2.17 (s, 3H). ^{13}C NMR (101 MHz, $CDCl_3$) δ 158.7, 156.3, 135.4, 135.4, 134.0, 133.8, 133.4, 130.7, 128.9, 128.7, 128.6, 128.4, 127.0, 126.1, 111.5, 111.2, 110.2, 110.1, 109.9, 109.9, 104.2, 103.9, 30.3, 28.6, 19.9, 19.8. ^{19}F NMR (376 MHz, $CDCl_3$) δ -122.28, -122.28. HRMS (ESI) *m/z*: $[M+Na]^+$ calcd for $C_{23}H_{20}FNSNa$ 416.0916; Found: 416.0919.

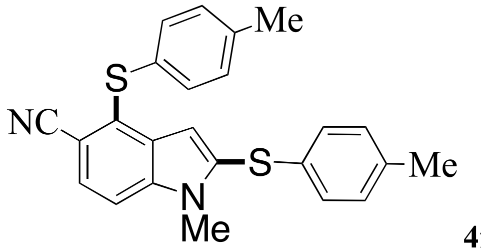

4f

Orange Solid, m.pt: 138-140 °C, 16 mg, 25% ^1H NMR (400 MHz, Chloroform-*d*) δ 7.90 (s, 1H), 7.44 (dd, J = 8.6, 1.6 Hz, 1H), 7.32 (dd, J = 8.6, 0.7 Hz, 1H), 6.98 – 6.92 (m, 7H), 6.90 (d, J = 8.1 Hz, 3H), 3.72 (s, 3H), 2.20 (s, 3H), 2.19 (s, 3H). ^{13}C NMR (101 MHz, $CDCl_3$) δ 138.7, 137.0, 135.9, 134.7, 132.5, 129.9, 129.1, 128.6, 127.8, 127.5, 126.7, 125.4, 124.8, 119.0, 112.2, 109.9, 103.0, 30.3, 28.6, 19.9, 19.9. HRMS (ESI) *m/z*: $[M+Na]^+$ calcd for $C_{24}H_{20}N_2S_2Na$ 423.0963; Found: 423.0966.

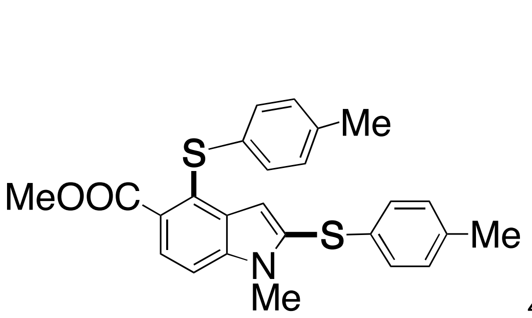

4g

Yellow Solid, m.pt: 165-167 °C, 30 mg, 49%. ^1H NMR (400 MHz, Chloroform-*d*) δ 8.46 (d, J = 1.0 Hz, 1H), 8.04 (dd, J = 8.7, 1.7 Hz, 1H), 7.40 (dd, J = 8.8, 0.7 Hz, 1H), 7.08 – 6.94 (m, 8H), 3.93 (s, 3H), 3.81 (s, 3H),

2.29 (s, 3H), 2.27 (s, 3H). ^{13}C NMR (101 MHz, $CDCl_3$) δ 167.7, 140.6, 137.0, 136.6, 135.1, 134.5, 131.4, 130.0, 129.5, 128.8, 128.2, 127.3, 125.0, 123.0, 123.0, 113.3, 109.8, 51.9, 31.3, 20.9, 20.9. HRMS (ESI) *m/z*: $[M+Na]^+$ calcd for $C_{25}H_{23}NO_2S_2Na$ 456.1068; Found: 456.1063.

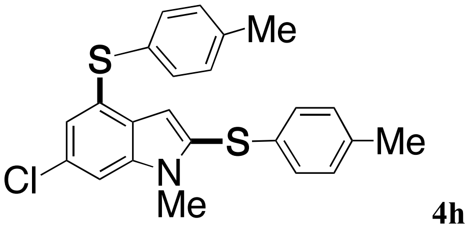

4h

Yellow Solid, m.pt: 82-84 °C, 45 mg, 71%. ^1H NMR (400 MHz, Chloroform-*d*) δ 7.46 (d, *J* = 8.5 Hz, 1H), 7.28 (d, *J* = 1.8 Hz, 1H), 7.04 (dd, *J* = 8.5, 1.8 Hz, 1H), 6.95 – 6.85 (m, 8H), 3.66 (s, 3H), 2.18 (s, 3H), 2.16 (s, 3H). ^{13}C NMR (101 MHz, $CDCl_3$) δ 137.6, 135.4, 134.5, 134.0, 133.3, 130.7, 128.9, 128.9, 128.4, 127.0, 126.5, 126.1, 120.6, 120.2, 110.9, 109.0, 30.1, 28.6, 19.9, 19.8. HRMS (ESI) *m/z*: $[M+Na]^+$ calcd for $C_{23}H_{20}ClNS_2Na$ 432.0621; Found: 432.0623.

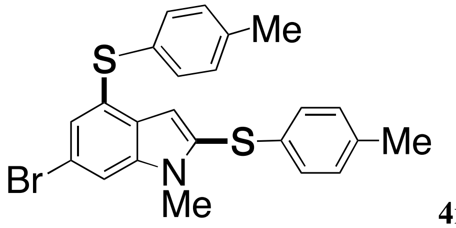

4i

Yellow Solid, m.pt: 81-83 °C, 43 mg, 70%. ^1H NMR (400 MHz, Chloroform-*d*) δ 7.45 (d, *J* = 1.6 Hz, 1H), 7.41 (d, *J* = 8.5 Hz, 1H), 7.20 – 7.15 (m, 1H), 6.93 (d, *J* = 2.4 Hz, 1H), 6.91 (d, *J* = 3.0 Hz, 3H), 6.89 (s, 2H), 6.87 (d, *J* = 2.1 Hz, 2H), 6.86 (s, 1H), 3.66 (s, 3H), 2.18 (s, 3H), 2.17 (s, 3H). ^{13}C NMR (101 MHz, $CDCl_3$) δ 138.0, 135.4, 134.5, 134.0, 133.3, 130.6, 128.9, 128.4, 127.0, 126.9, 126.1, 123.2, 120.5, 116.6, 112.0, 110.9, 30.1, 19.9, 19.8. HRMS (ESI) *m/z*: $[M+Na]^+$ calcd for $C_{23}H_{20}BrNS_2Na$ 476.0113; Found: 476.0118.

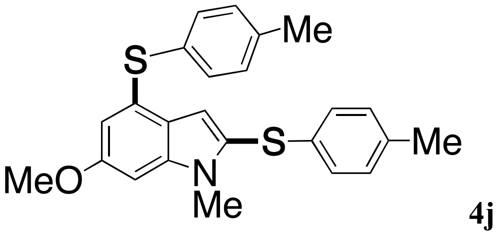

4j

Pink Solid, m.pt: 151-153 °C, 44 mg, 70%. ^1H NMR (400 MHz, Chloroform-*d*) δ 7.51 (dd, *J* = 15.5, 8.6 Hz, 2H), 7.04 (d, *J* = 8.3 Hz, 2H), 7.01 – 6.94 (m, 6H), 6.88 – 6.83 (m, 1H), 6.82 (d, *J* = 2.2 Hz, 1H), 3.91 (s, 3H),

3.76 (s, 3H), 2.28 (s, 3H), 2.26 (s, 3H). ^{13}C NMR (101 MHz, $CDCl_3$) δ 157.9, 139.2, 136.5, 136.0, 134.9, 134.8, 132.7, 130.2, 129.8, 129.4, 129.3, 127.6, 127.5, 127.0, 123.3, 121.1, 111.1, 93.2, 55.7, 31.0, 20.9, 20.9. HRMS (ESI) *m/z*: $[M+Na]^+$ calcd for $C_{24}H_{23}NOS_2Na$ 428.1119; Found: 428.118.

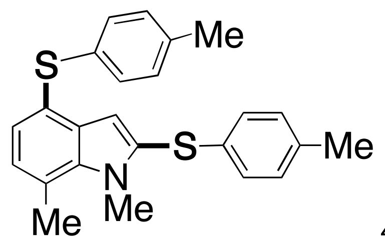

4k

Yellow Solid, m.pt: 131-133 °C, 20 mg, 32%. ^{1}H NMR (400 MHz, Chloroform-*d*) δ 7.44 (dd, *J* = 5.4, 3.9 Hz, 1H), 6.94 (d, *J* = 5.4 Hz, 3H), 6.92 (d, *J* = 2.0 Hz, 2H), 6.89 (d, *J* = 5.0 Hz, 3H), 6.88 – 6.83 (m, 3H), 4.01 (s, 3H), 2.71 (s, 3H), 2.18 (s, 3H), 2.16 (s, 3H). ^{13}C NMR (101 MHz, $CDCl_3$) δ 136.5, 135.0, 134.3, 133.8, 133.6, 131.3, 129.0, 128.8, 128.3, 126.6, 125.8, 125.7, 120.7, 119.8, 117.5, 110.7, 33.2, 19.9, 19.8, 19.3. HRMS (ESI) *m/z*: $[M+Na]^+$ calcd for $C_{24}H_{23}NS_2Na$ 412.1172; Found: 412.1170.

4. NMR spectra of isolated products

^{1}H NMR spectrum of 3a

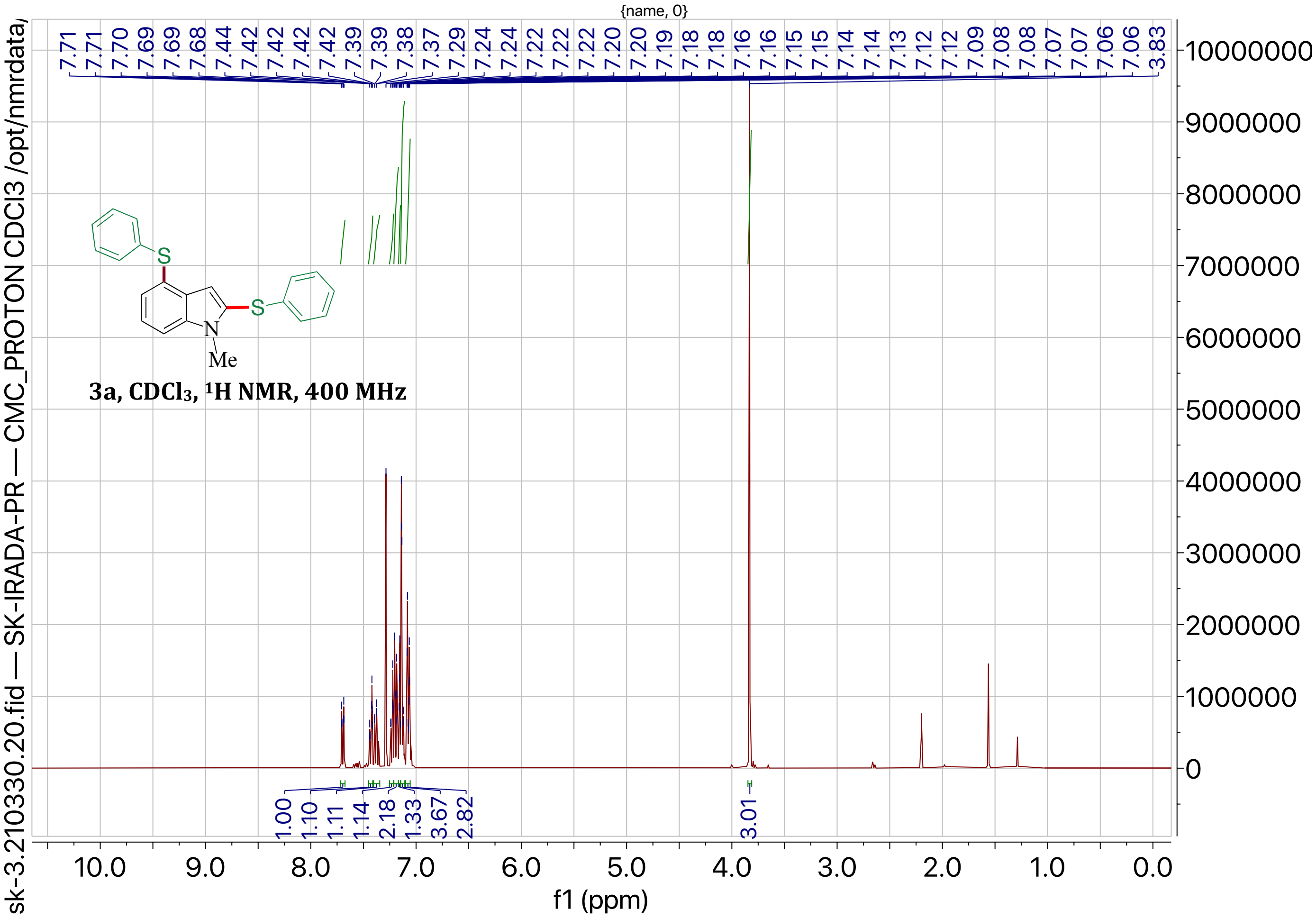

^{13}C{^{1}H} NMR spectrum of 3a

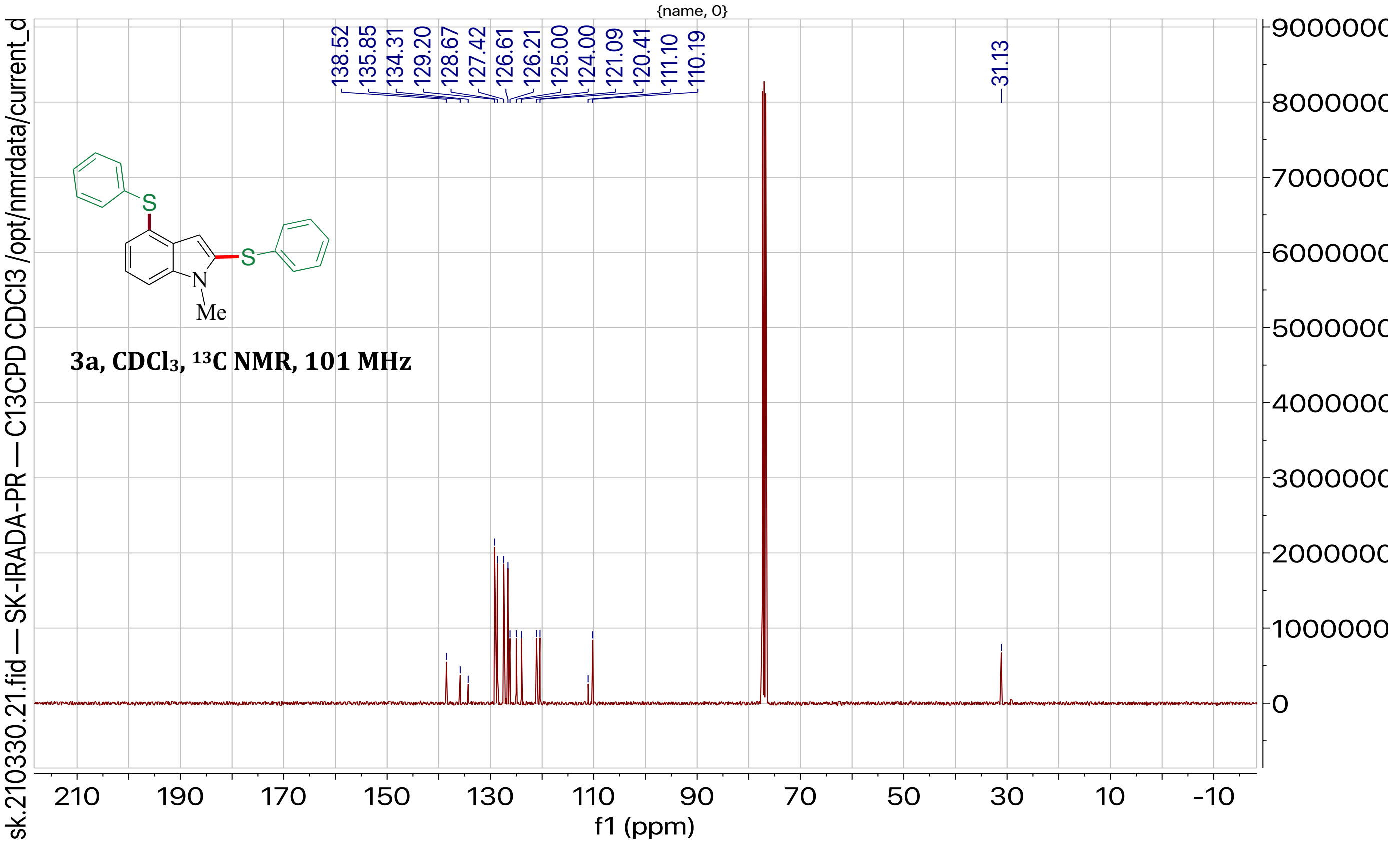

HRMS spectrum of 3a

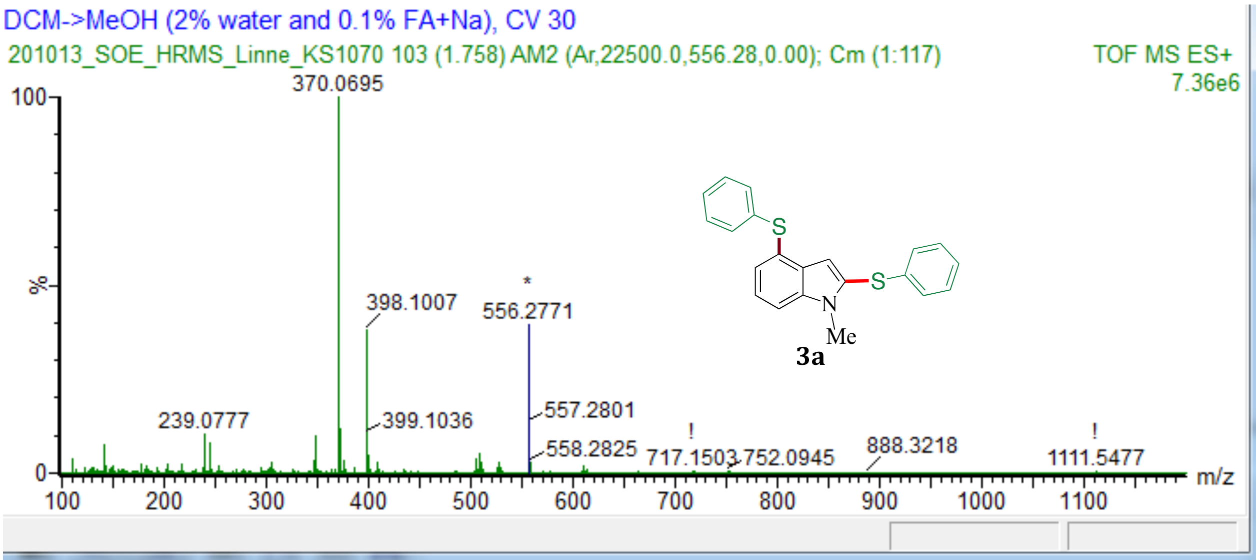

Single Mass Analysis
Tolerance = 2.0 mDa / DBE: min = -0.5, max = 100.0
Element prediction: Off
Number of isotope peaks used for i-FIT = 3
Monoisotopic Mass, Even Electron Ions
154 formula(e) evaluated with 2 results within limits (all results (up to 1000) for each mass)
Elements Used:
C: 0-50 H: 0-50 N: 0-2 O: 0-1 Na: 0-1 S: 0-2

Mass	Calc. Mass	mDa	PPM	DBE	Formula	i...	Fit Conf %	C	H	N	O	Na	S
370.0695	370.0690	0.5	1.4	21.5	C26 H12 N S	6.1...	0.00	26	12	1			1
	370.0700	-0.5	-1.4	13.5	C21 H17 N Na S2	6.0...	100.00	21	17	1		1	2

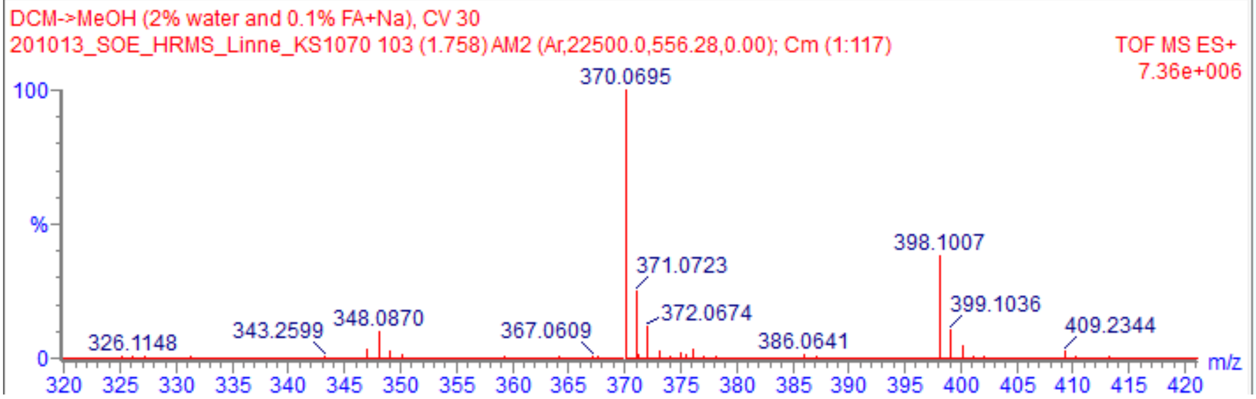

^{1}H NMR spectrum of 3b

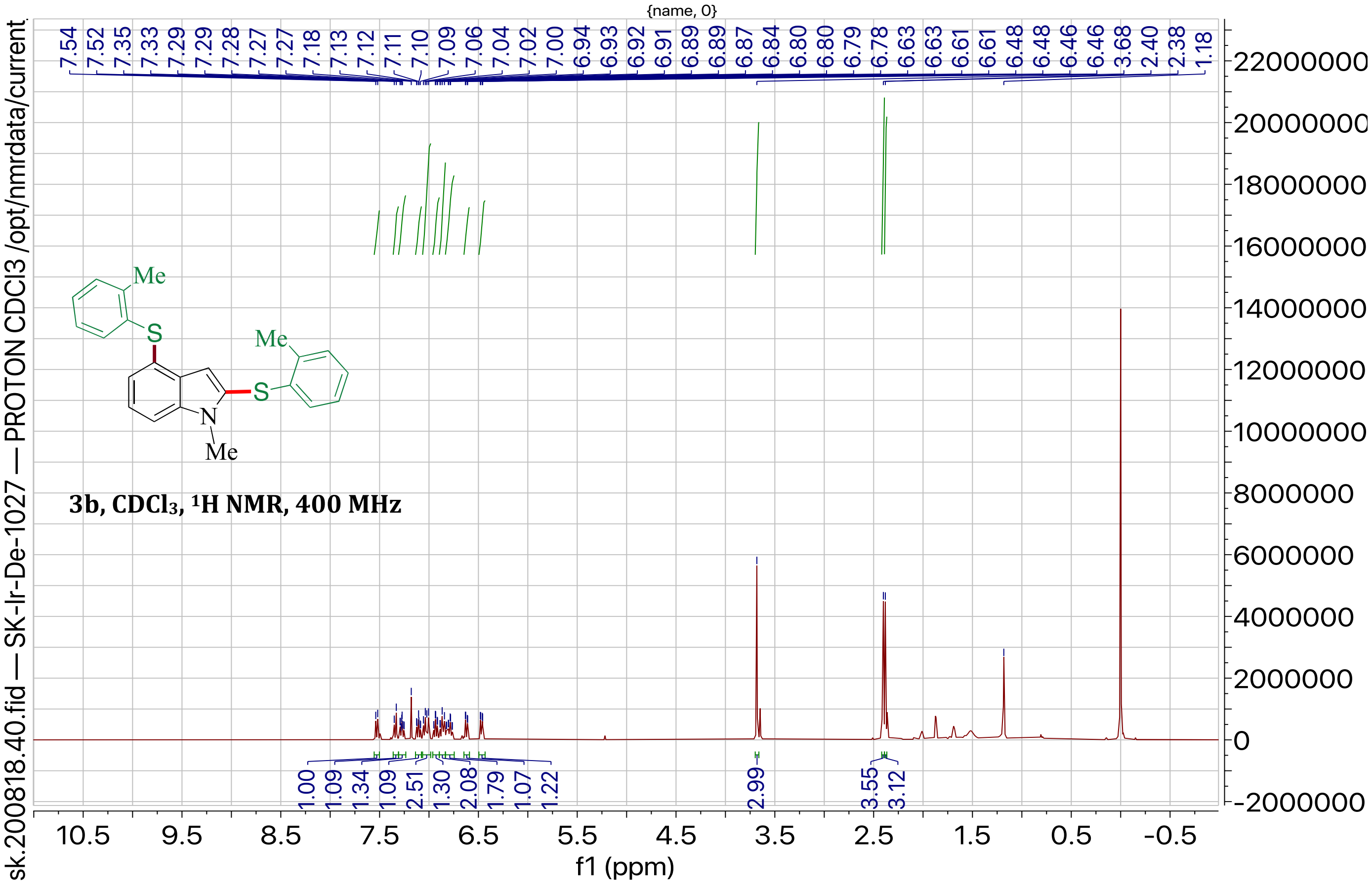

^{13}C{^{1}H} NMR spectrum of 3b

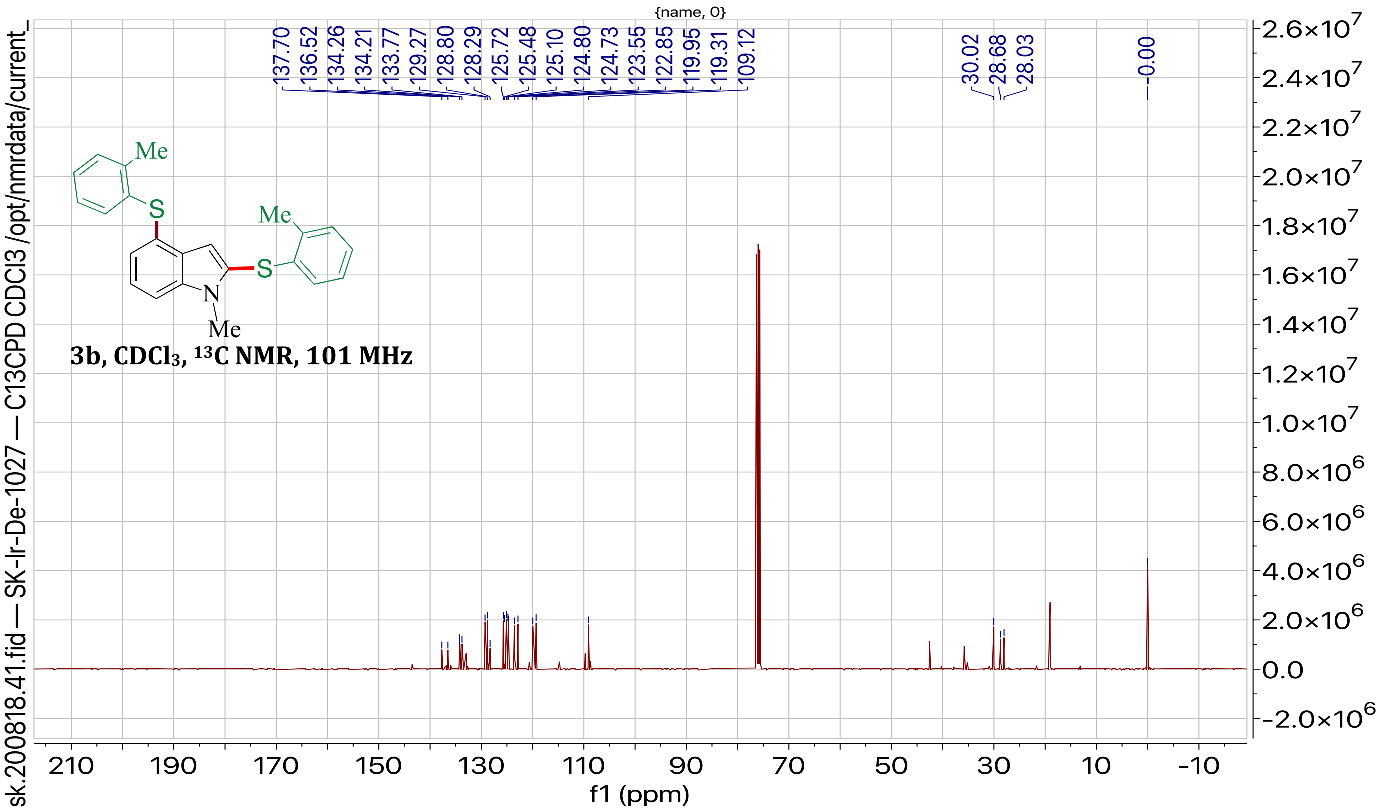

HRMS spectrum of 3b

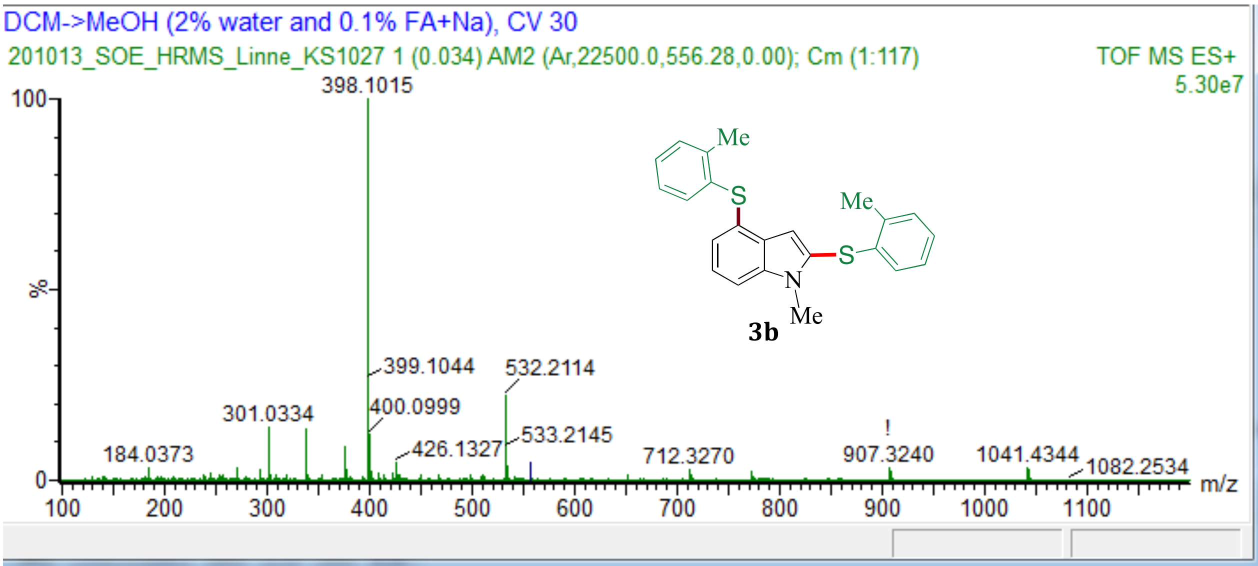

Single Mass Analysis
Tolerance = 2.0 mDa / DBE: min = -0.5, max = 100.0
Element prediction: Off
Number of isotope peaks used for i-FIT = 3
Monoisotopic Mass, Even Electron Ions
308 formula(e) evaluated with 2 results within limits (all results (up to 1000) for each mass)
Elements Used:
C: 0-50 H: 0-50 N: 0-2 O: 0-3 Na: 0-1 S: 0-2

Mass	Calc. Mass	mDa	PPM	DBE	Formula	i...	Fit Conf %	C	H	N	O	Na	S
398.1015	398.1013	0.2	0.5	13.5	C23 H21 N Na S2	0...	100.00	23	21	1		1	2
	398.1003	1.2	3.0	21.5	C28 H16 N S	1...	0.00	28	16	1			1

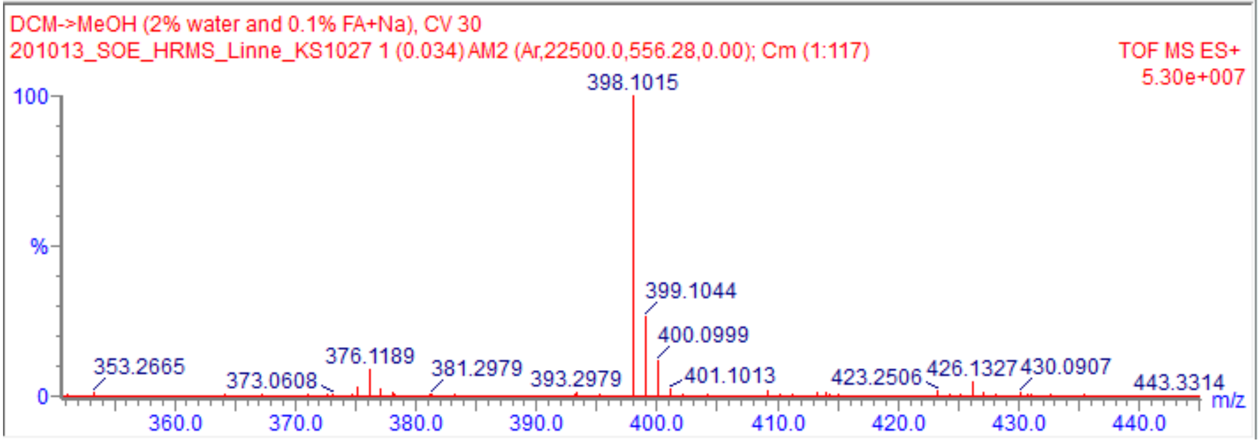

^{1}H NMR spectrum of 3c

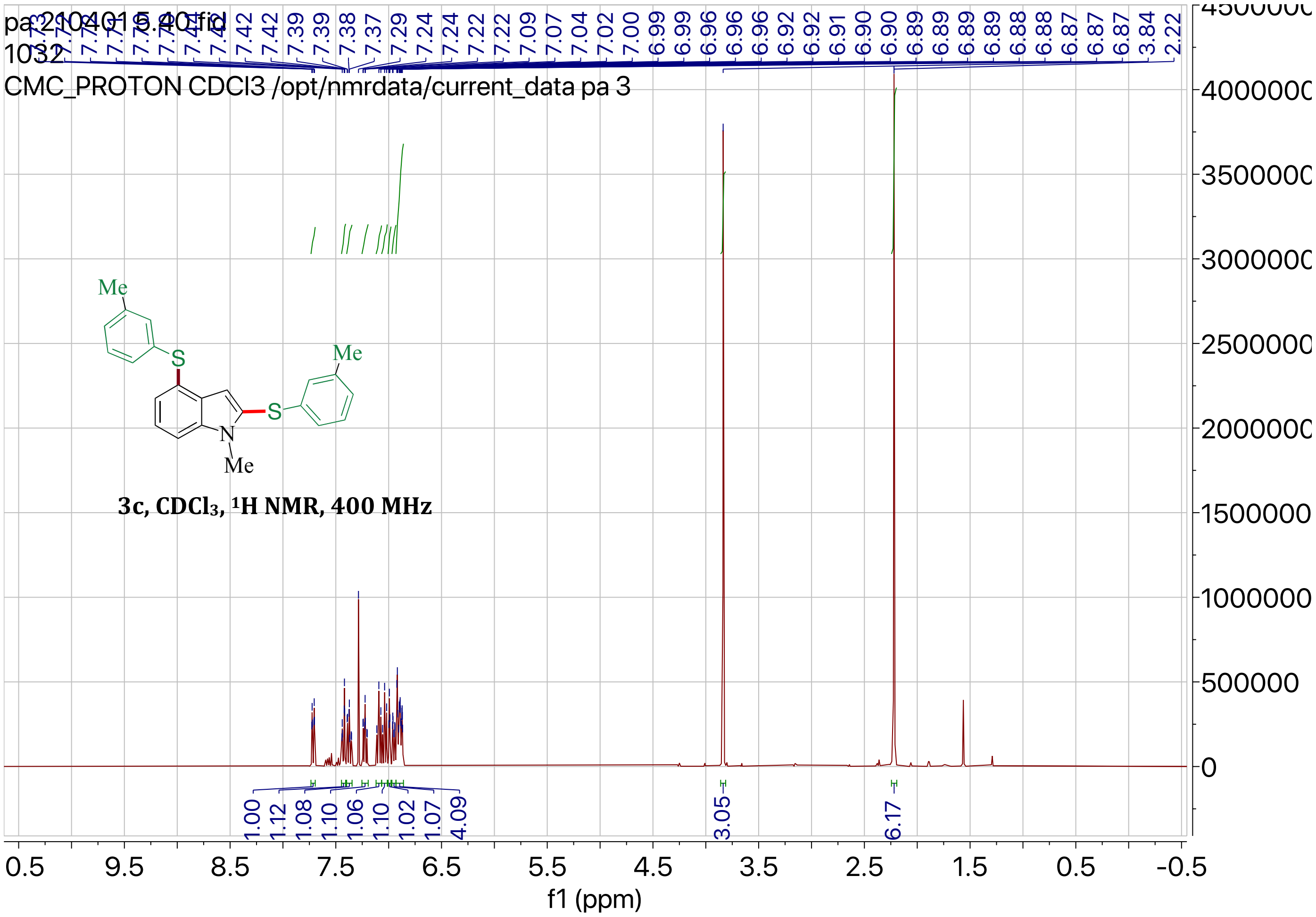

^{13}C{^{1}H} NMR spectrum of 3c

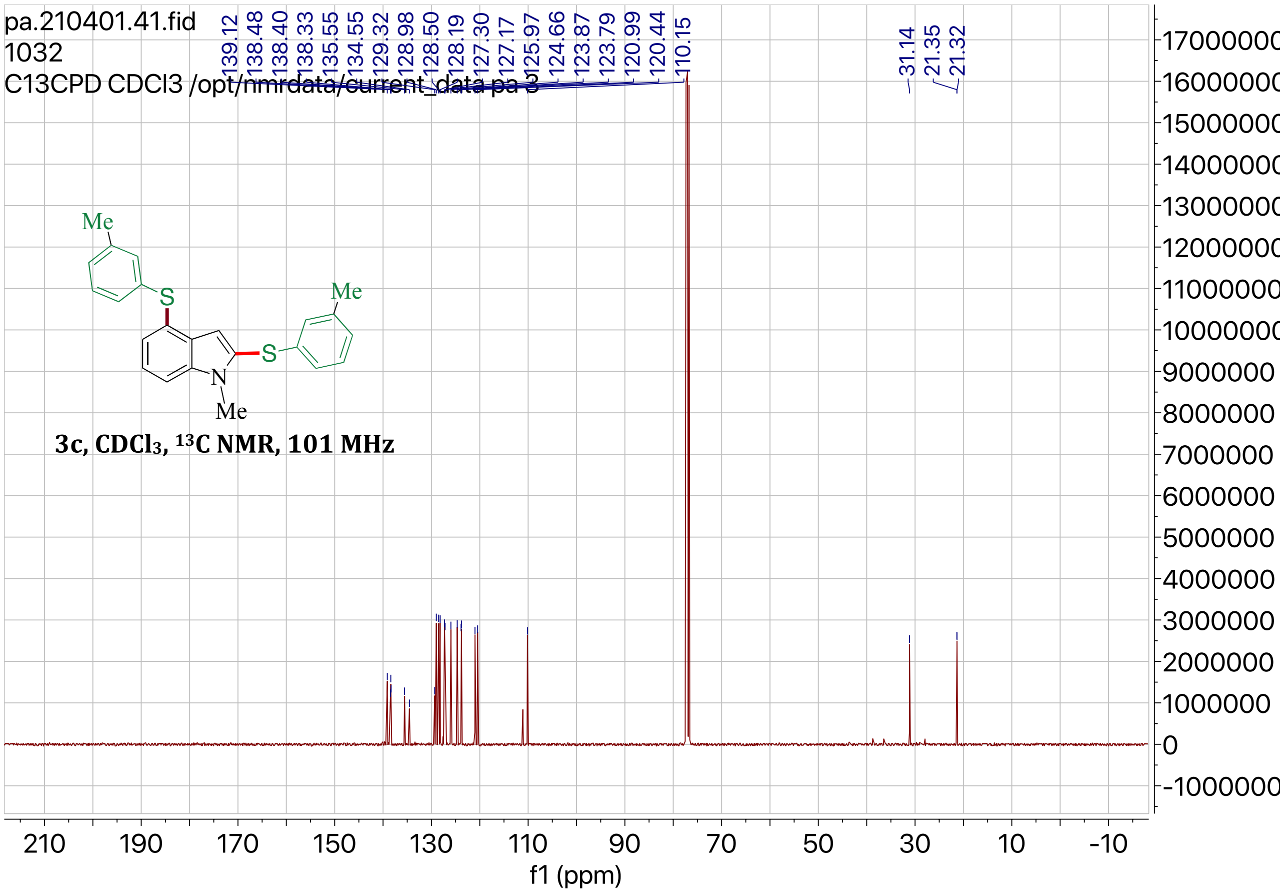

HRMS spectrum of 3c

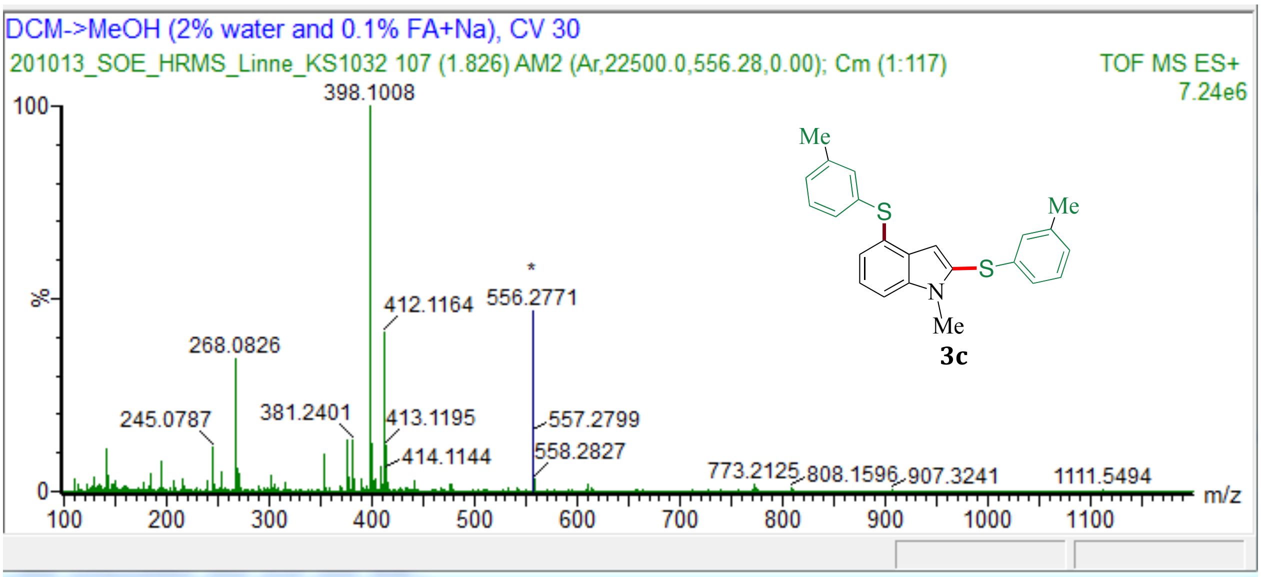

Single Mass Analysis
Tolerance = 2.0 mDa / DBE: min = -0.5, max = 100.0
Element prediction: Off
Number of isotope peaks used for i-FIT = 3
Monoisotopic Mass, Even Electron Ions
308 formula(e) evaluated with 2 results within limits (all results (up to 1000) for each mass)
Elements Used:
C: 0-50 H: 0-50 N: 0-2 O: 0-3 Na: 0-1 S: 0-2

Mass	Calc. Mass	mDa	PPM	DBE	Formula	i...	Fit Conf %	C	H	N	O	Na	S
398.1008	398.1003	0.5	1.3	21.5	C28 H16 N S	61...	0.00	28	16	1			1
	398.1013	-0.5	-1.3	13.5	C23 H21 N Na S2	60...	100.00	23	21	1		1	2

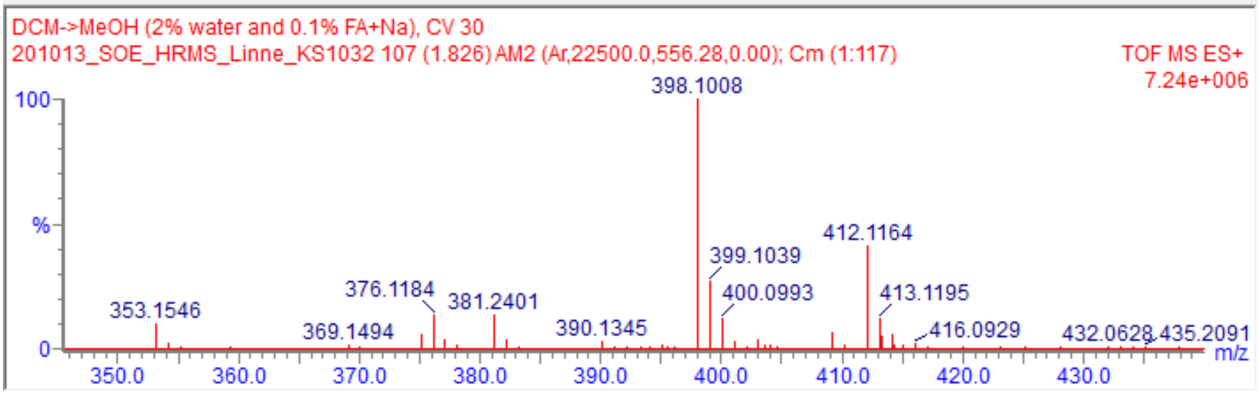

^{1}H NMR spectrum of 3d

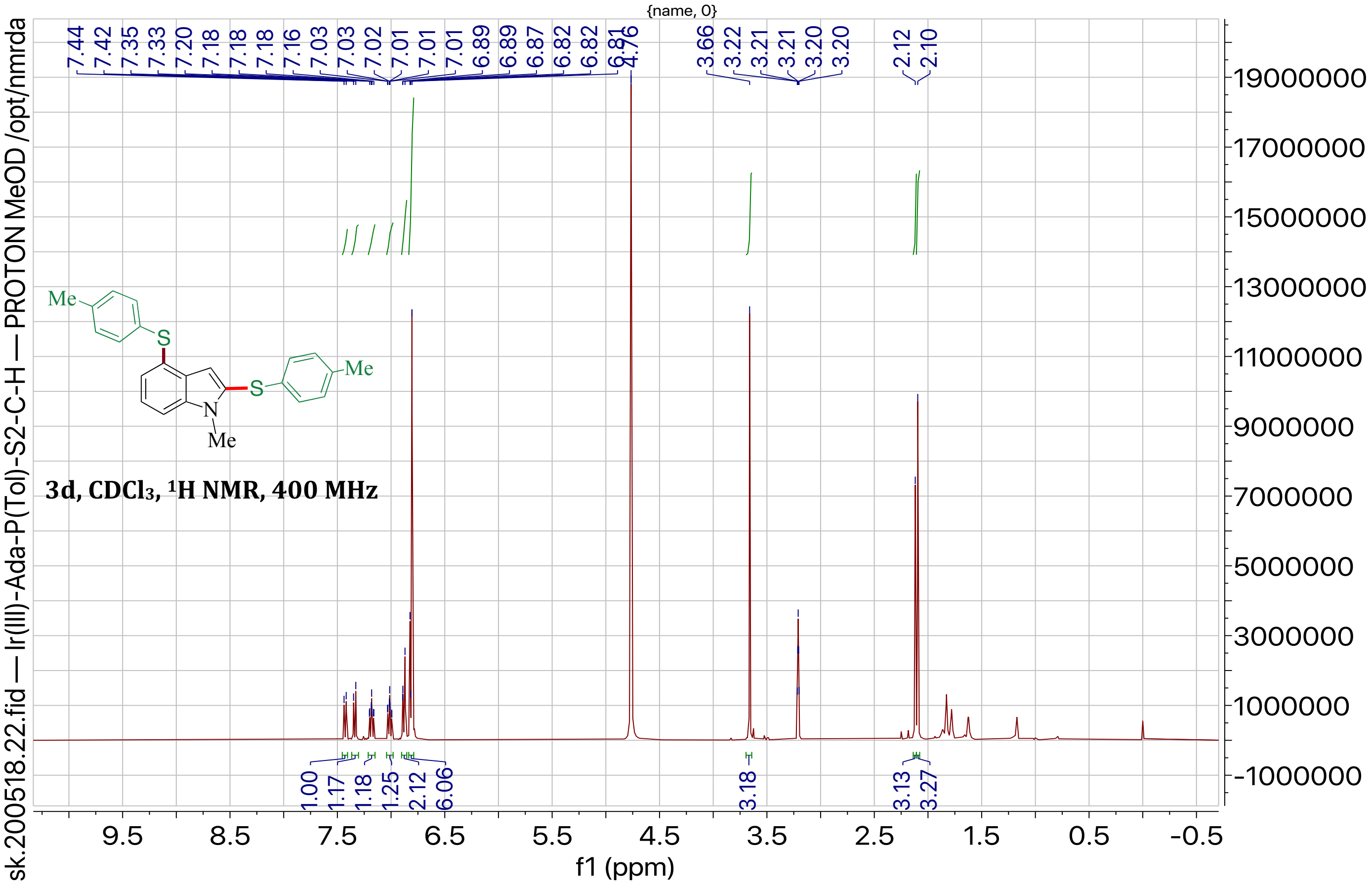

^{13}C{^{1}H} NMR spectrum of 3d

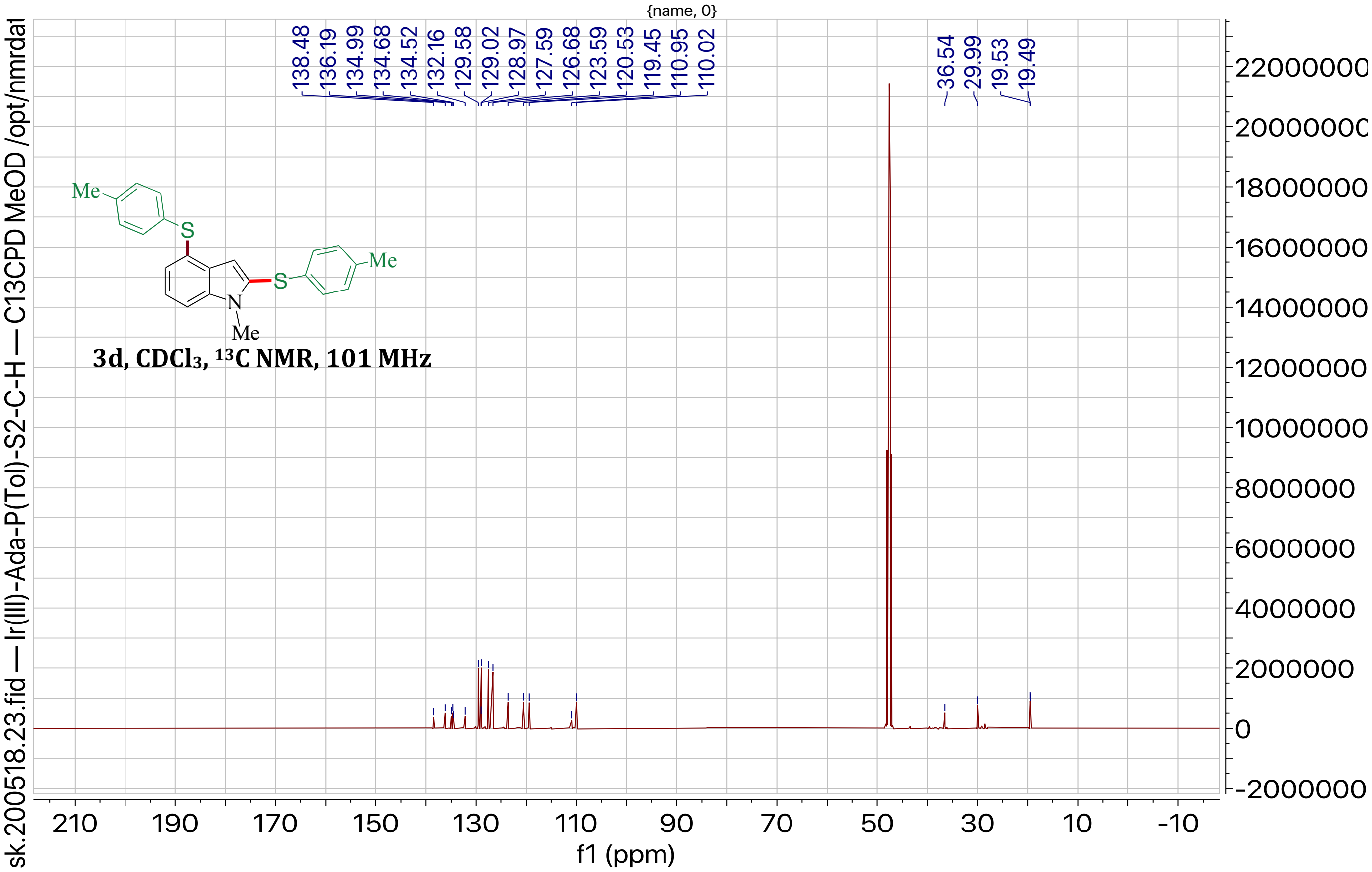

HRMS spectrum of 3d

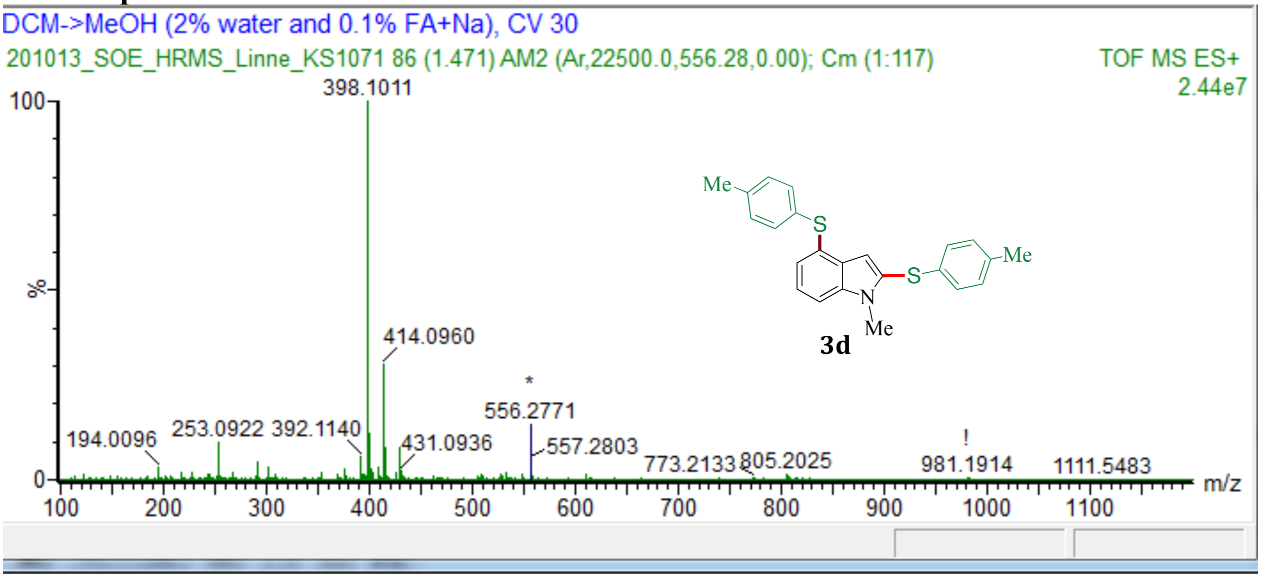

Single Mass Analysis

Tolerance = 2.0 mDa / DBE: min = -0.5, max = 100.0

Element prediction: Off

Number of isotope peaks used for i-FIT = 3

Monoisotopic Mass, Even Electron Ions

156 formula(e) evaluated with 2 results within limits (all results (up to 1000) for each mass)

Elements Used:

C: 0-50 H: 0-50 N: 0-2 O: 0-1 Na: 0-1 S: 0-2

Mass	Calc. Mass	mDa	PPM	DBE	Formula	i.	i. Fit Conf %	C	H	N	O	Na	S
398.1011	398.1013	-0.2	-0.5	13.5	C23 H21 N Na S2	6...	0...100.00	23	21	1		1	2
	398.1003	0.8	2.0	21.5	C28 H16 N S	6...	1...0.00	28	16	1			1

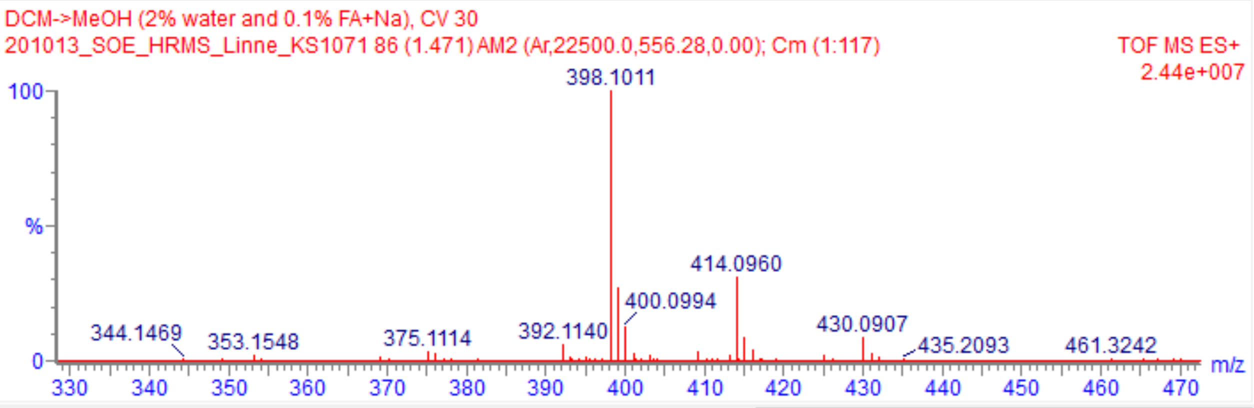

^{1}H NMR spectrum of 3e

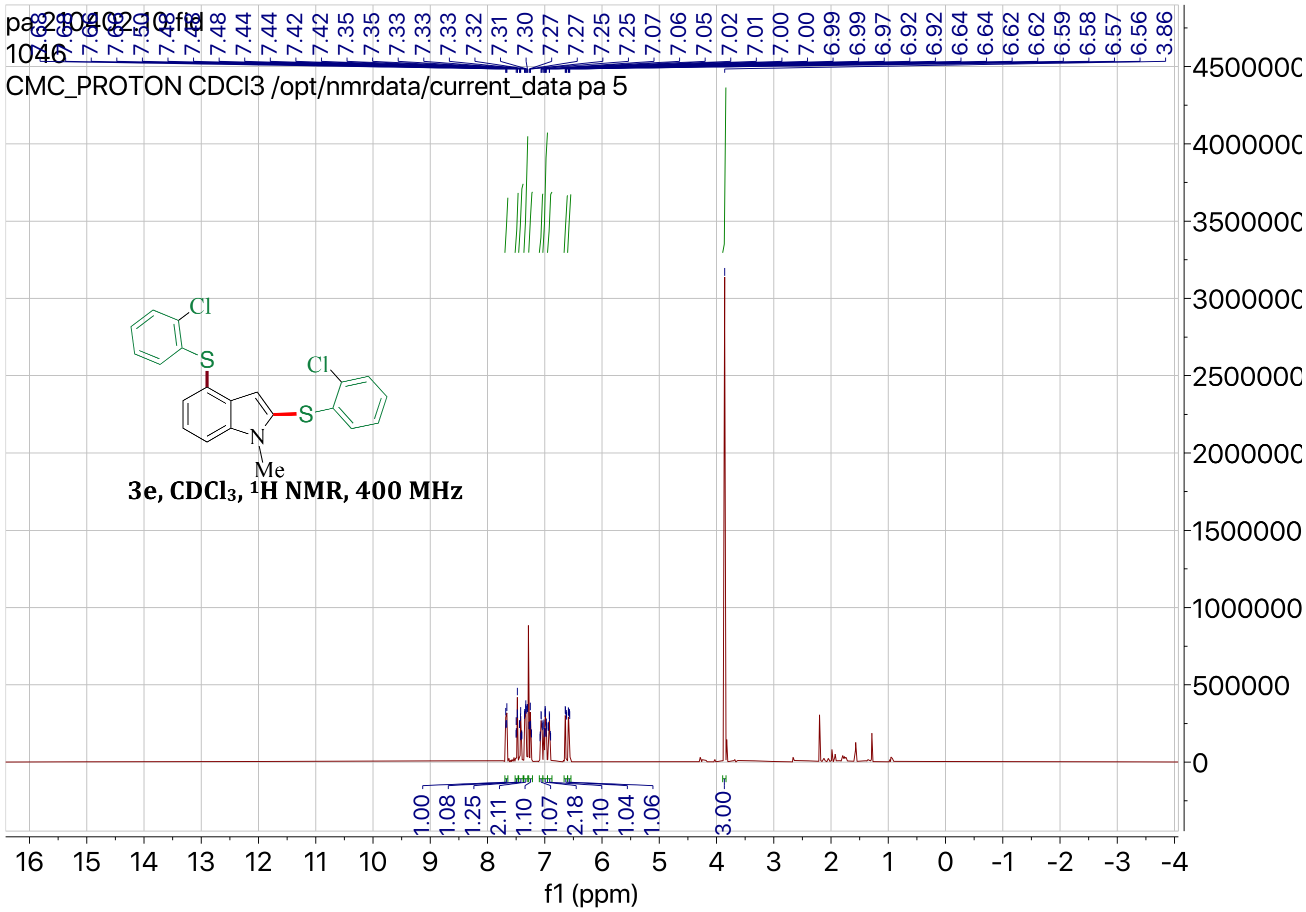

^{13}C{^{1}H} NMR spectrum of 3e

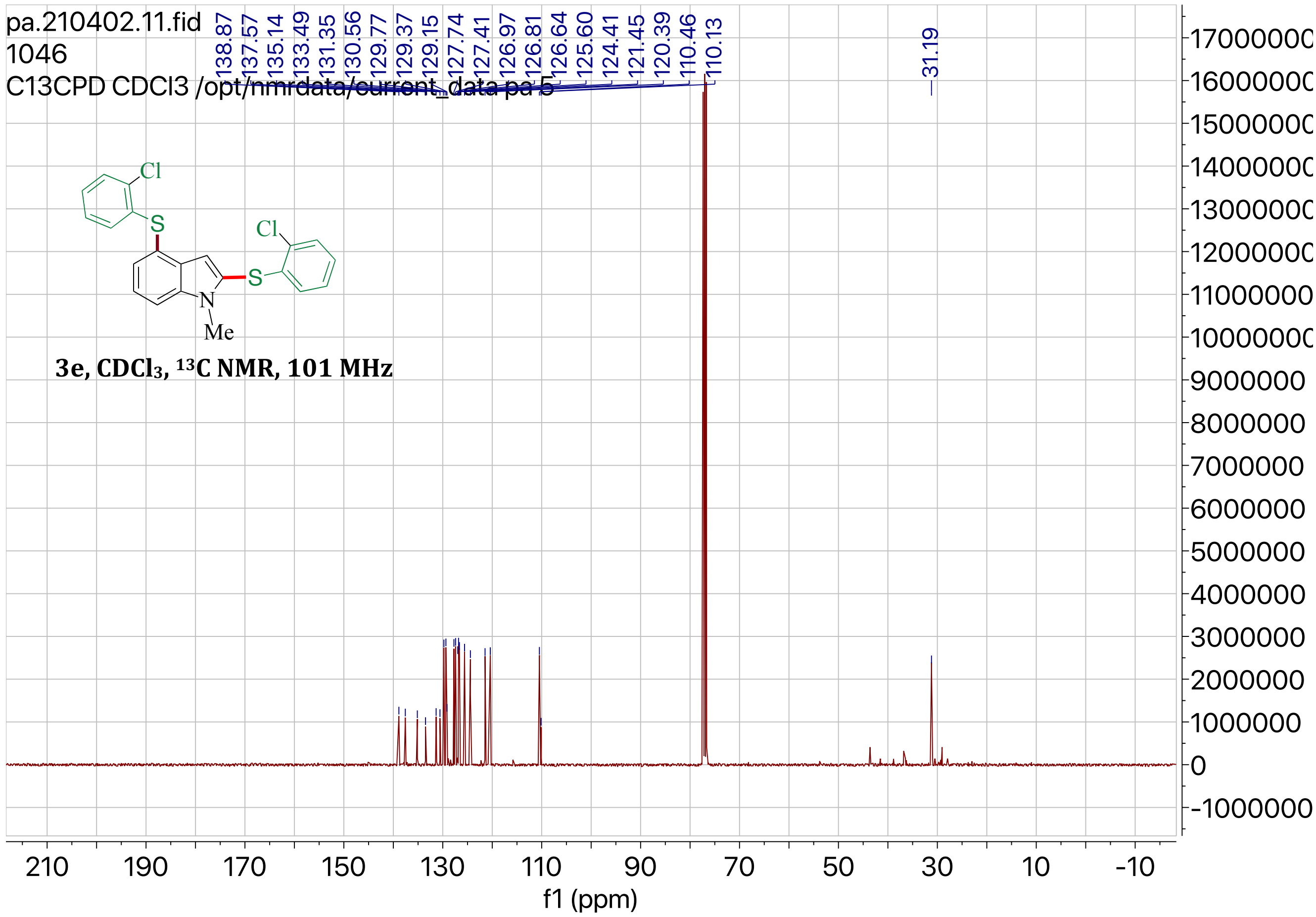

HRMS spectrum of 3e

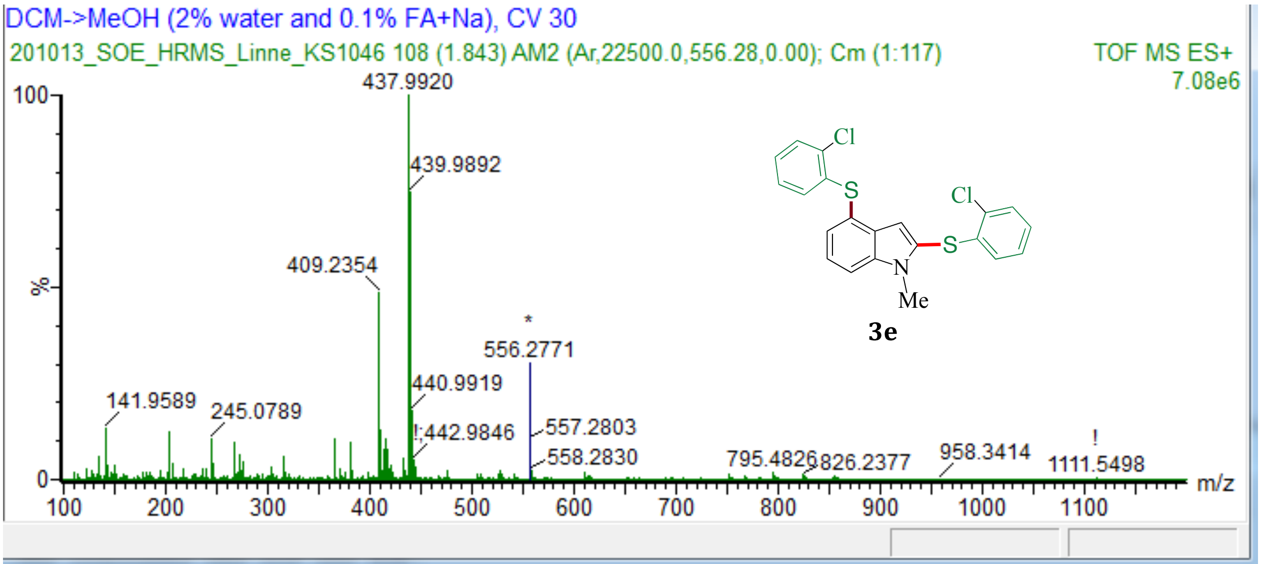

Single Mass Analysis

Tolerance = 2.0 mDa / DBE: min = -0.5, max = 100.0
Element prediction: Off
Number of isotope peaks used for i-FIT = 3

Monoisotopic Mass, Even Electron Ions
603 formula(e) evaluated with 3 results within limits (all results (up to 1000) for each mass)
Elements Used:
C: 0-50 H: 0-50 N: 0-2 O: 0-3 Na: 0-1 S: 0-2
Cl: 1-2

Mass	Calc. Mass	mDa	PPM	DBE	Formula		Fit Conf %	C	H	N	O	Na	S	Cl
437.9920	437.9921	-0.1	-0.2	13.5	C21 H15 N Na S2 Cl2	50.1	100.00	21	15	1		1	2	2
	437.9911	0.9	2.1	21.5	C26 H10 N S Cl2	51.0	0.00	26	10	1			1	2
	437.9934	-1.4	-3.2	23.5	C26 H6 N O3 Na Cl	51.0	0.00	26	6	1	3	1		1

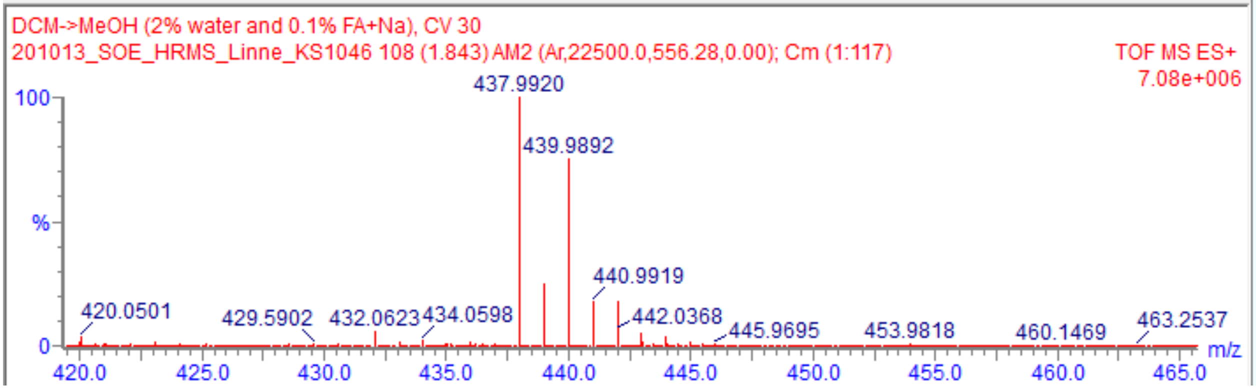

^{1}H NMR spectrum of 3f

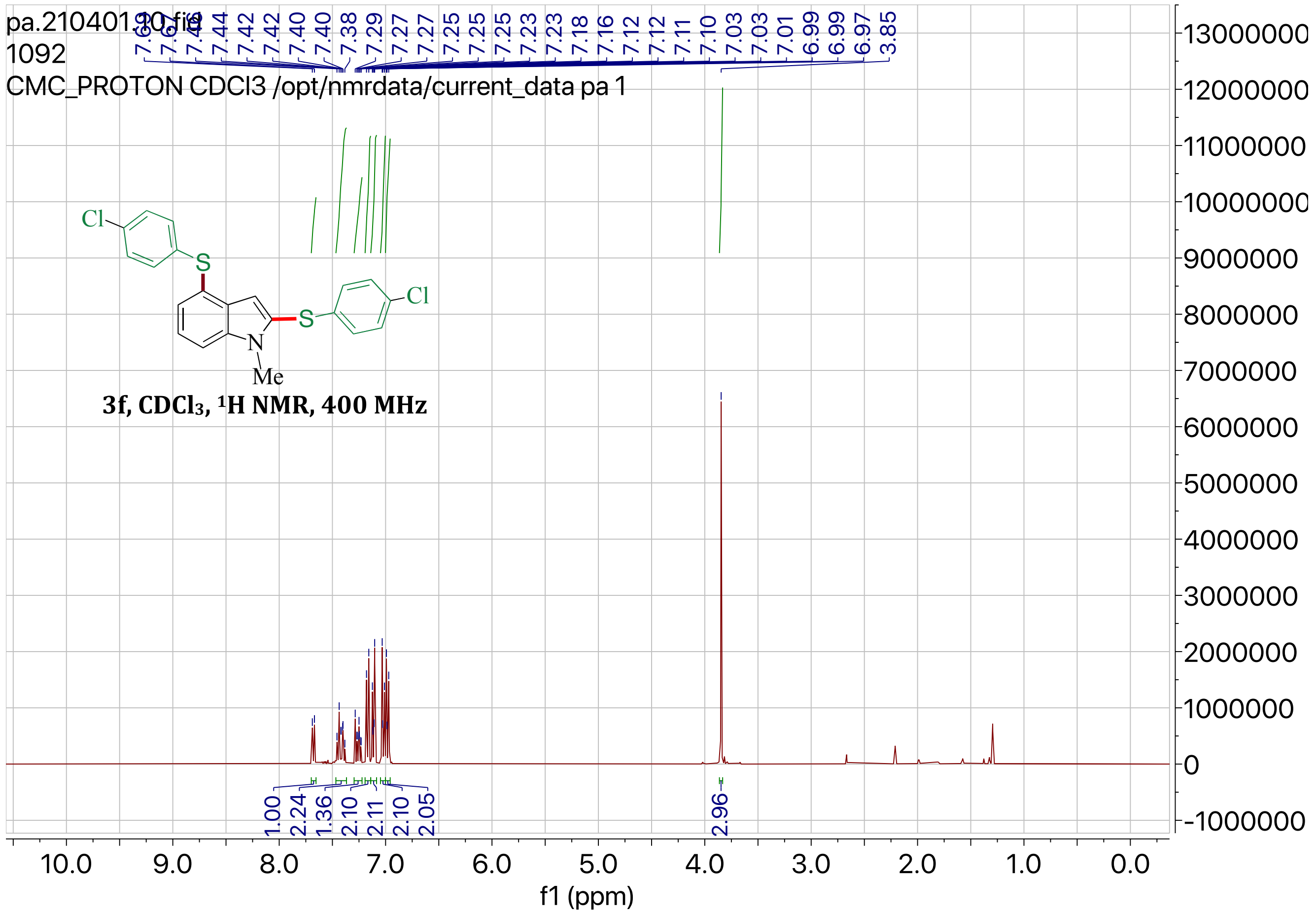

$^{13}C\{^{1}H\}$ NMR spectrum of 3f

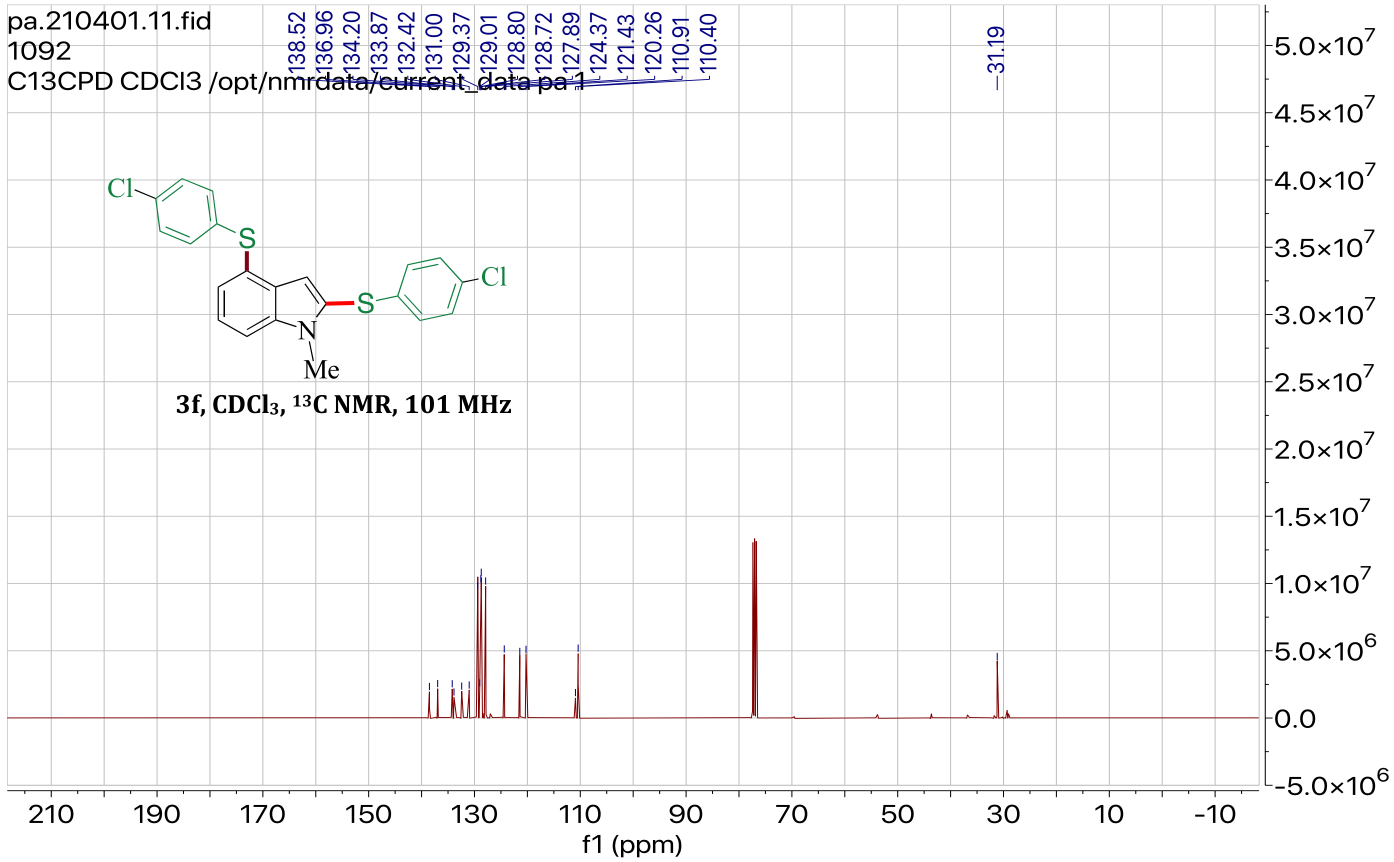

^{1}H NMR spectrum of 3g

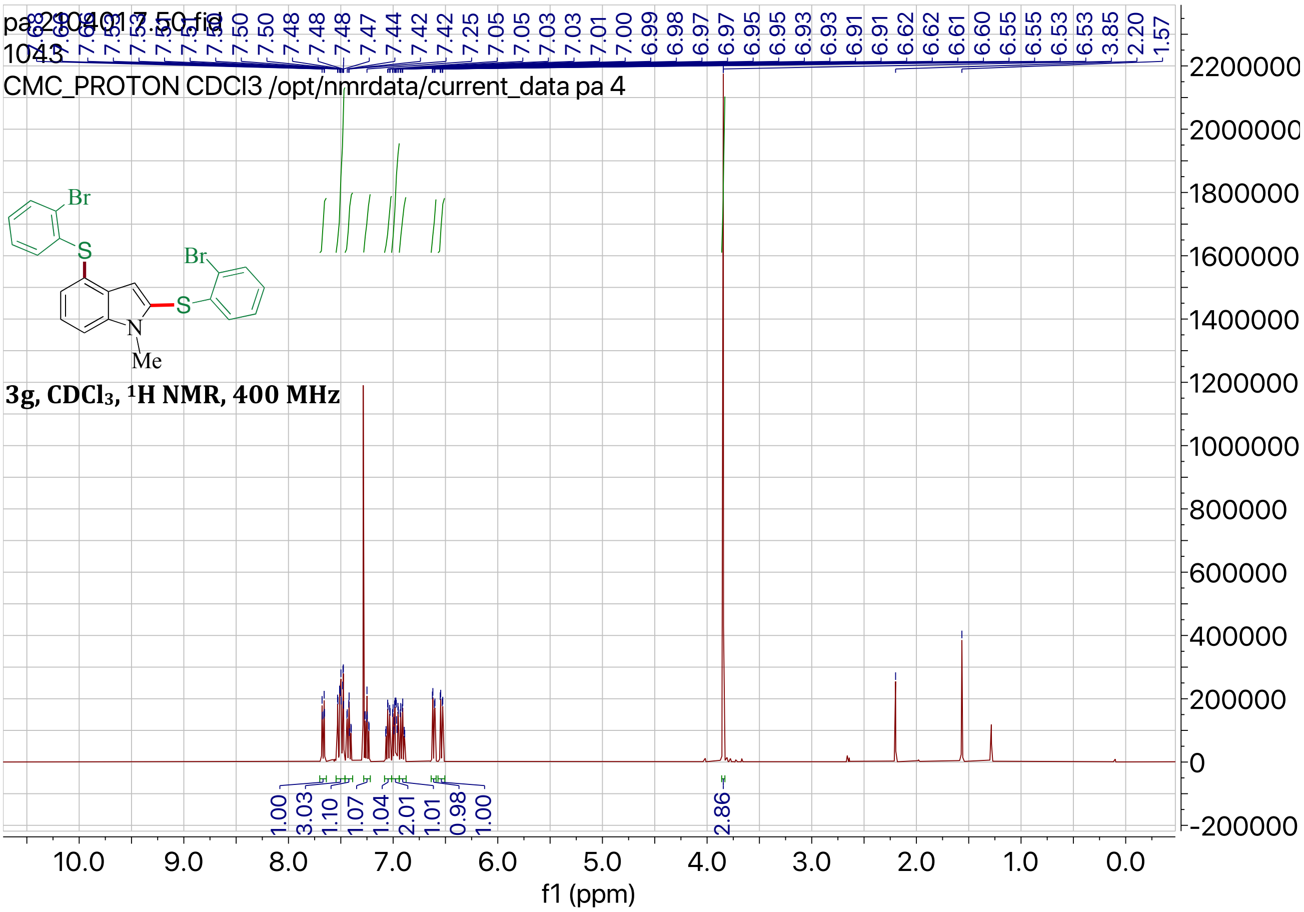

$^{13}C\{^{1}H\}$ NMR spectrum of 3g

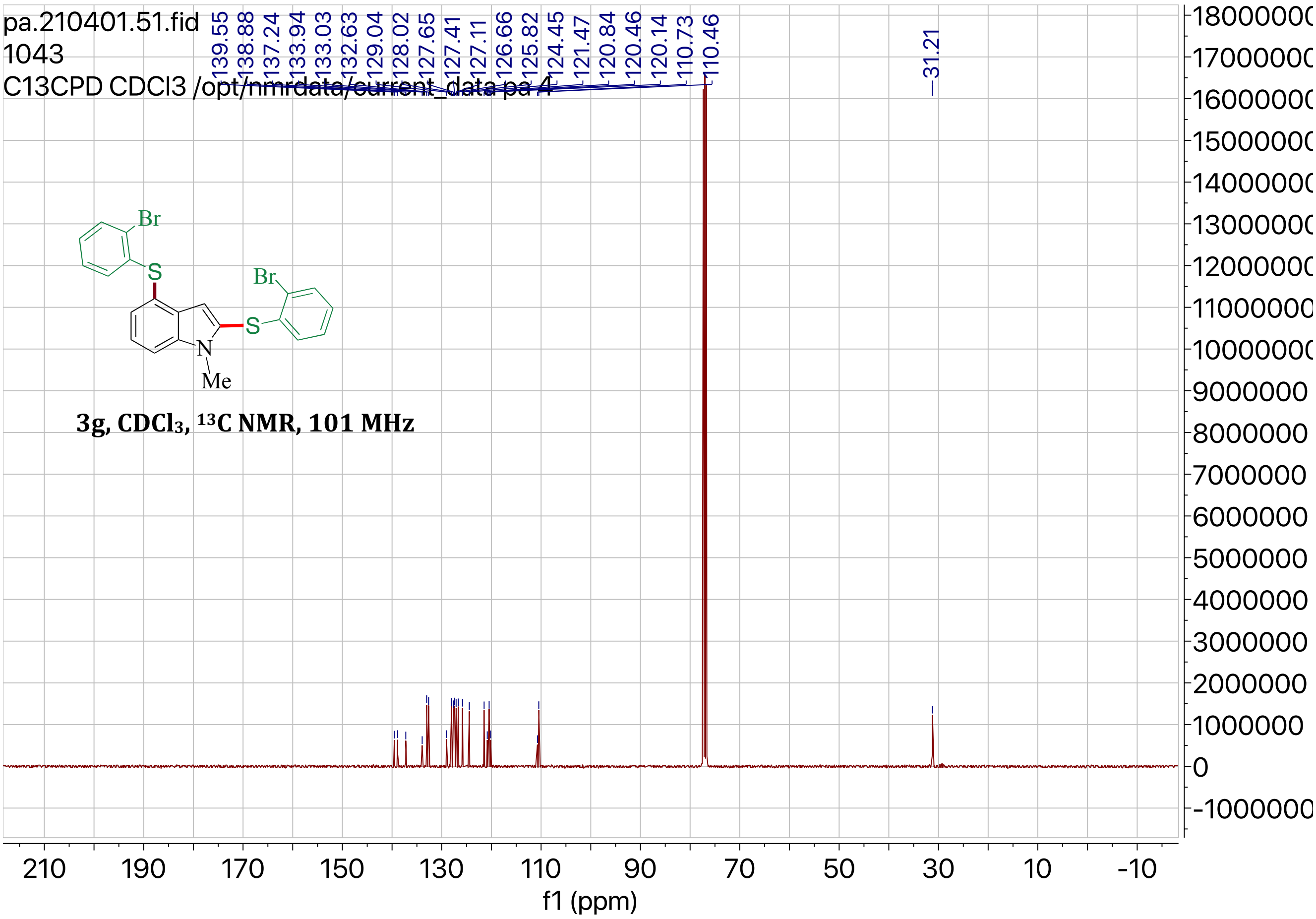

HRMS spectrum of 3g

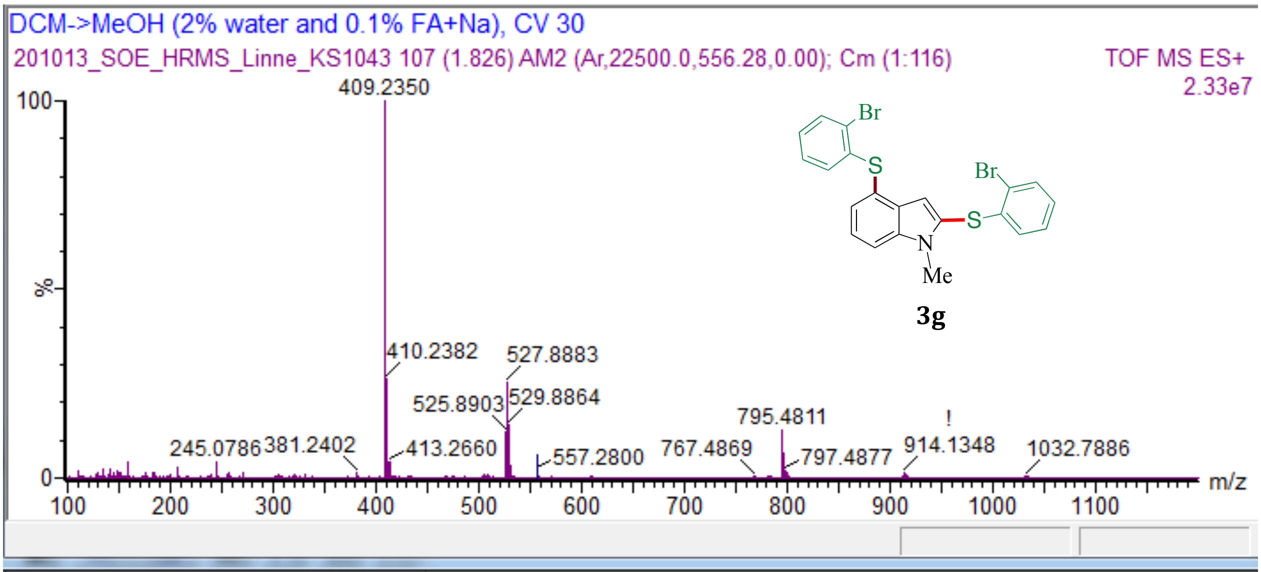

Single Mass Analysis

Tolerance = 2.0 mDa / DBE: min = -0.5, max = 100.0

Element prediction: Off

Number of isotope peaks used for i-FIT = 3

Monoisotopic Mass, Even Electron Ions

308 formula(e) evaluated with 2 results within limits (all results (up to 1000) for each mass)

Elements Used:

C: 0-50 H: 0-50 N: 0-2 O: 0-1 Na: 0-1 S: 1-2

[illegible]

Mass	Calc. Mass	mDa	PPM	DBE	Formula	i...	Fit Conf %	C	H	N	O	Na	S	Br
525.8903	525.8901	0.2	0.4	21.5	C26 H10 N S Br2	51....	0.00	26	10	1			1	2
	525.8910	-0.7	-1.3	13.5	C21 H15 N Na S2 Br2	50....	100.00	21	15	1		1	2	2

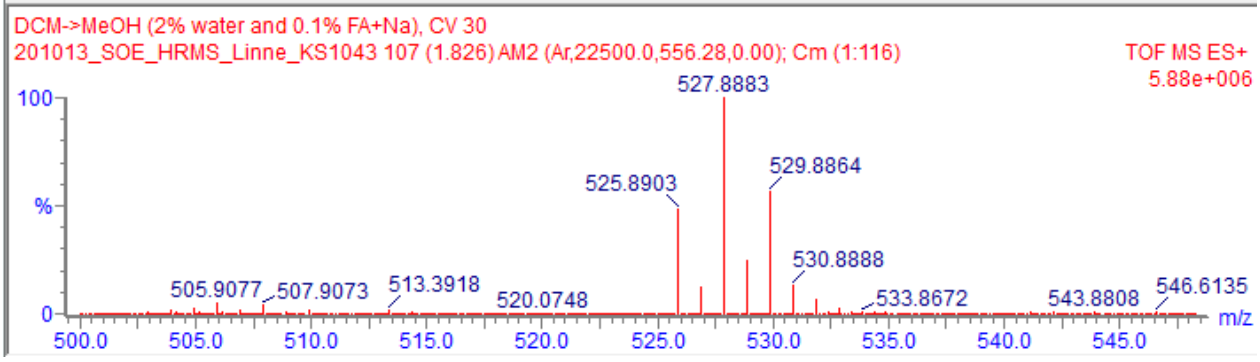

^{1}H NMR spectrum of 3h

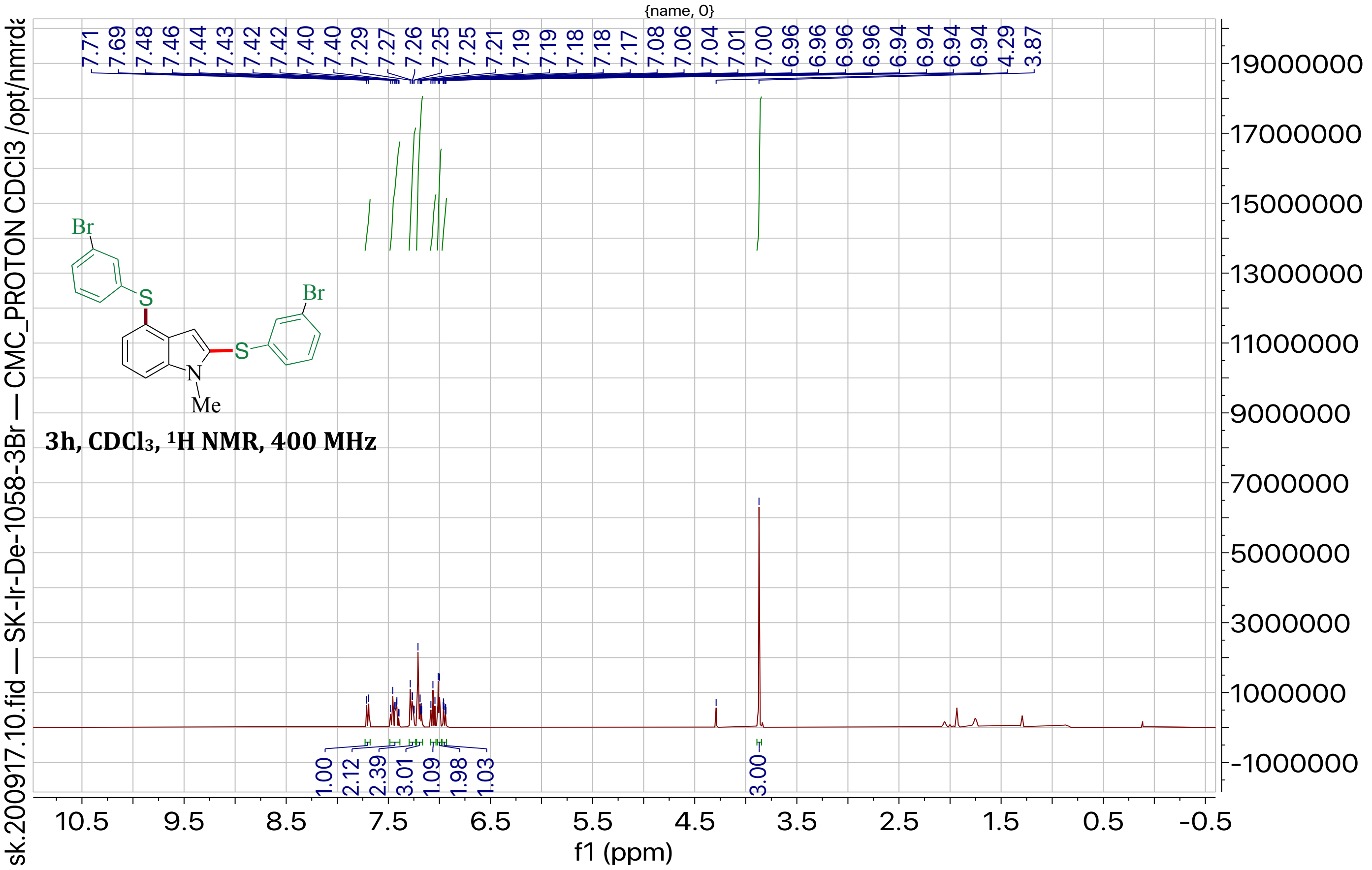

^{13}C{^{1}H} NMR spectrum of 3h

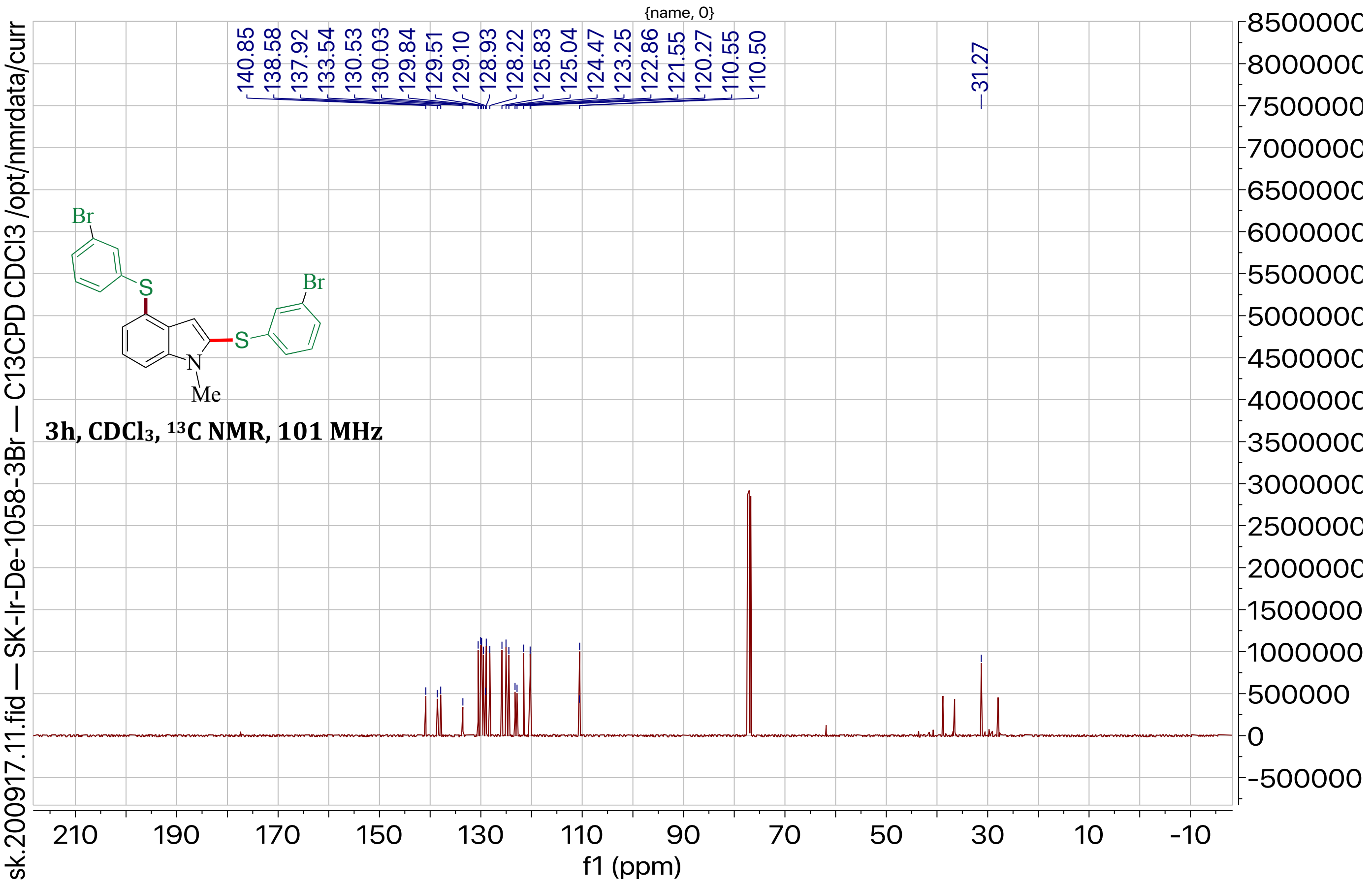

HRMS spectrum of 3h

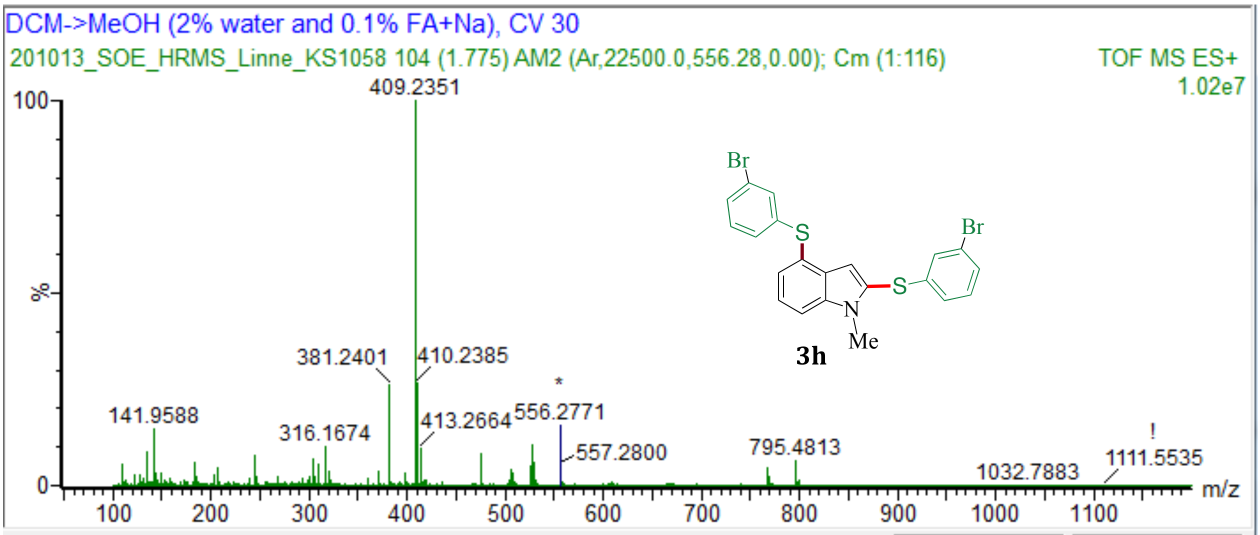

Single Mass Analysis

Tolerance = 2.0 mDa / DBE: min = -0.5, max = 100.0

Element prediction: Off

Number of isotope peaks used for i-FIT = 3

Monoisotopic Mass, Even Electron Ions

918 formula(e) evaluated with 2 results within limits (all results (up to 1000) for each mass)

Elements Used:

C: 0-50 H: 0-50 N: 0-2 O: 0-3 Na: 0-1 S: 0-2

Br: 0-2

Mass	Calc. Mass	mDa	PPM	DBE	Formula	i...	Fit Conf %	C	H	N	O	Na	S	Br
525.8902	525.8901	0.1	0.2	21.5	C26 H10 N S Br2	52...	10.47	26	10	1			1	2
	525.8910	-0.8	-1.5	13.5	C21 H15 N Na S2 Br2	50...	89.53	21	15	1		1	2	2

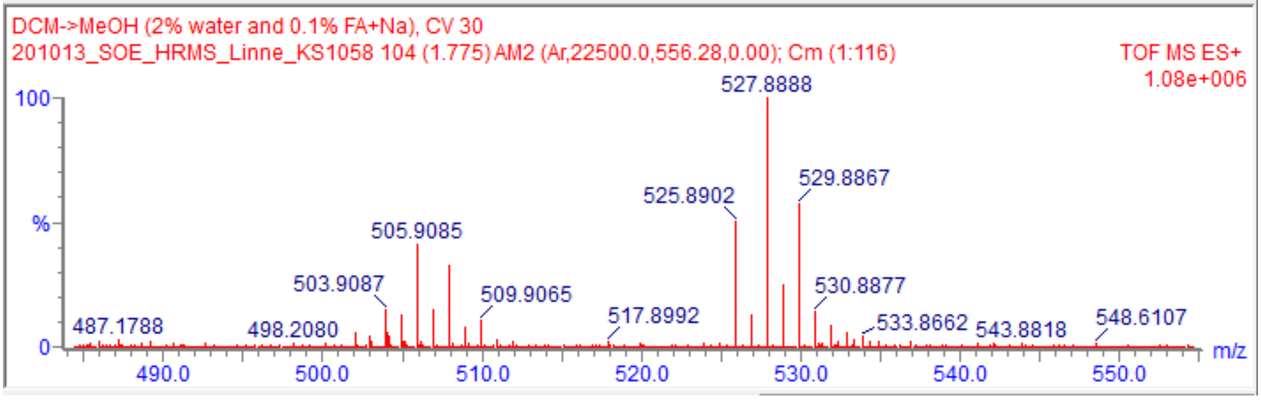

^{1}H NMR spectrum of 3i

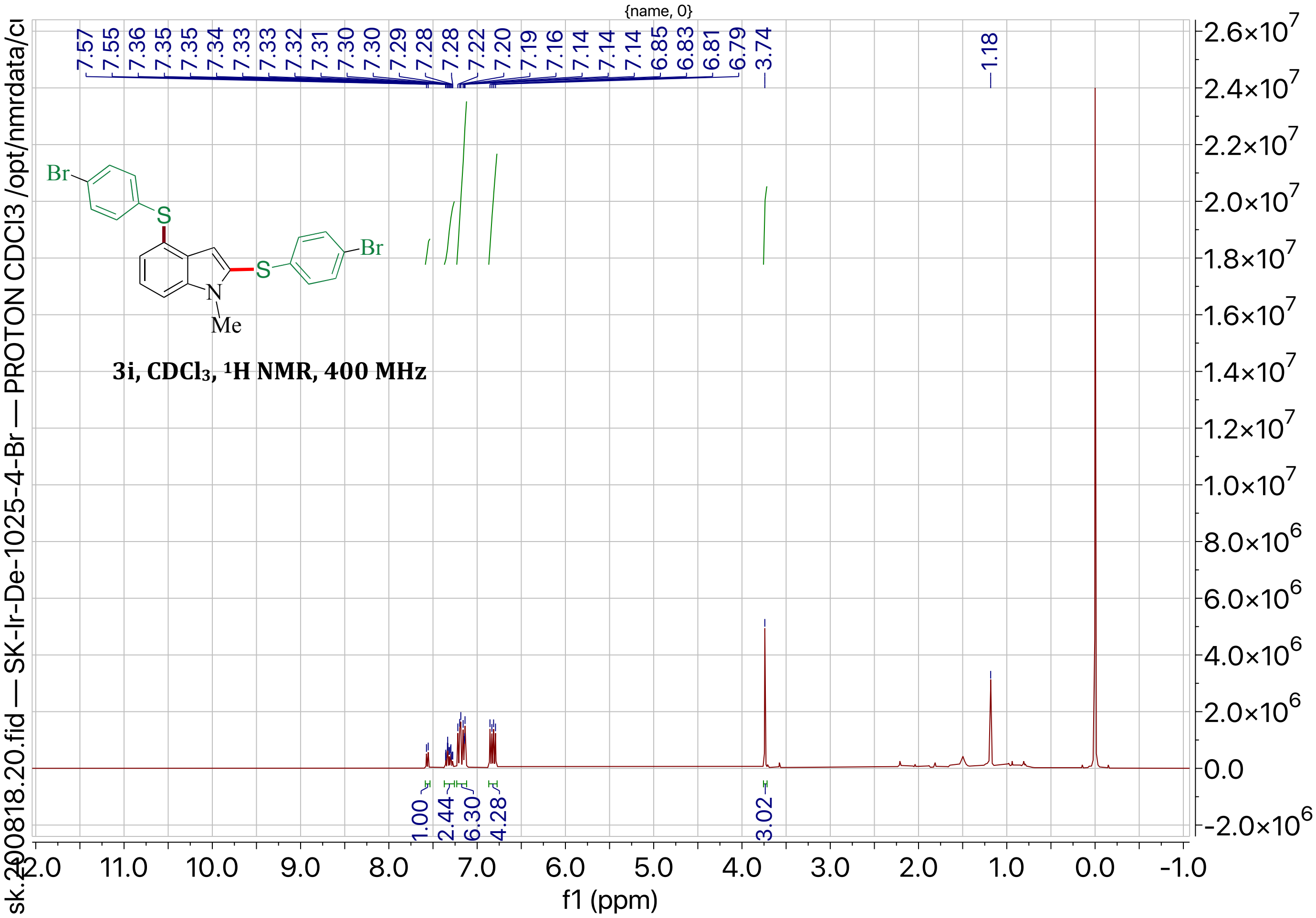

$^{13}C\{^{1}H\}$ NMR spectrum of 3i

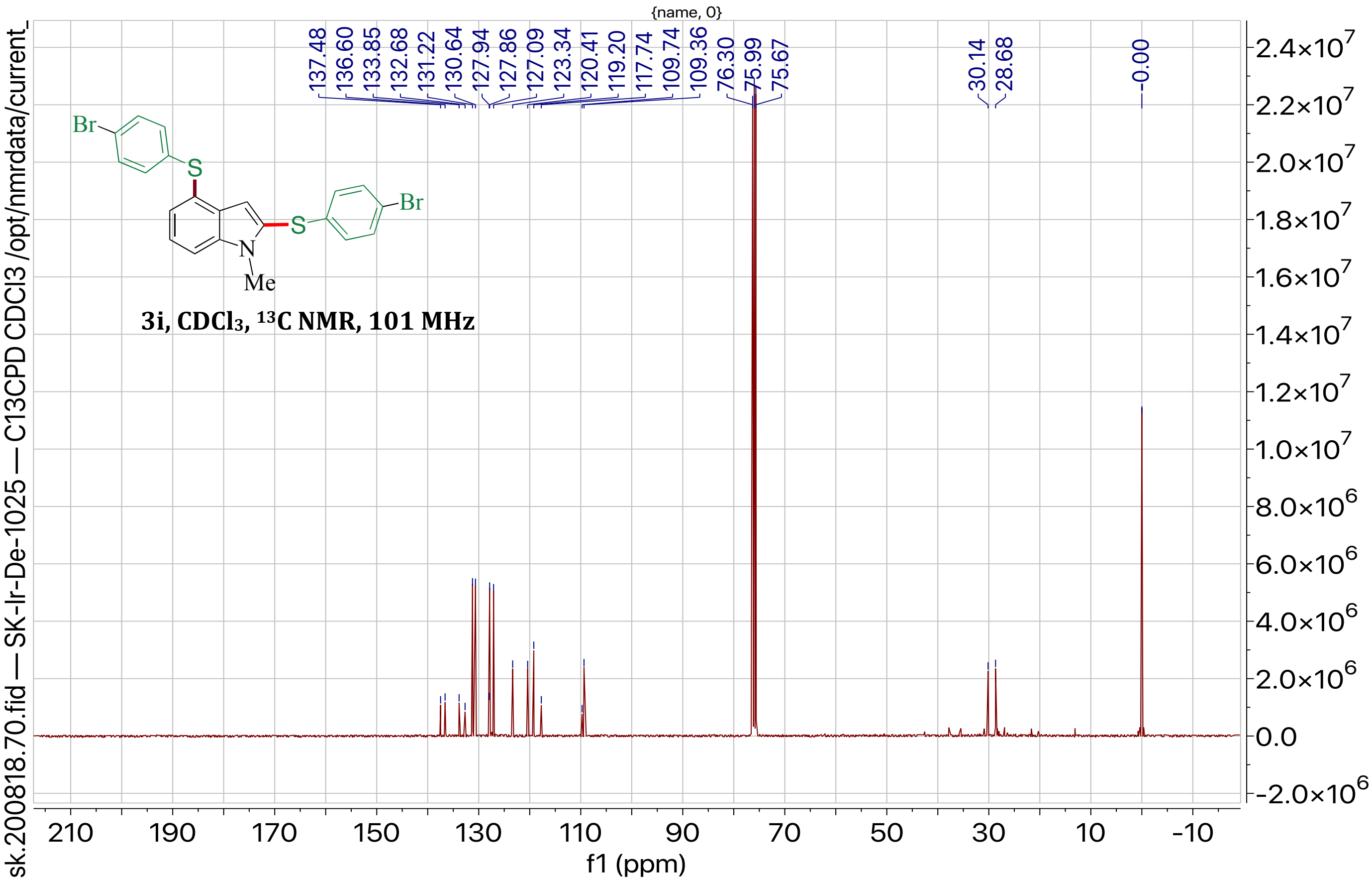

^{1}H NMR spectrum of 3j

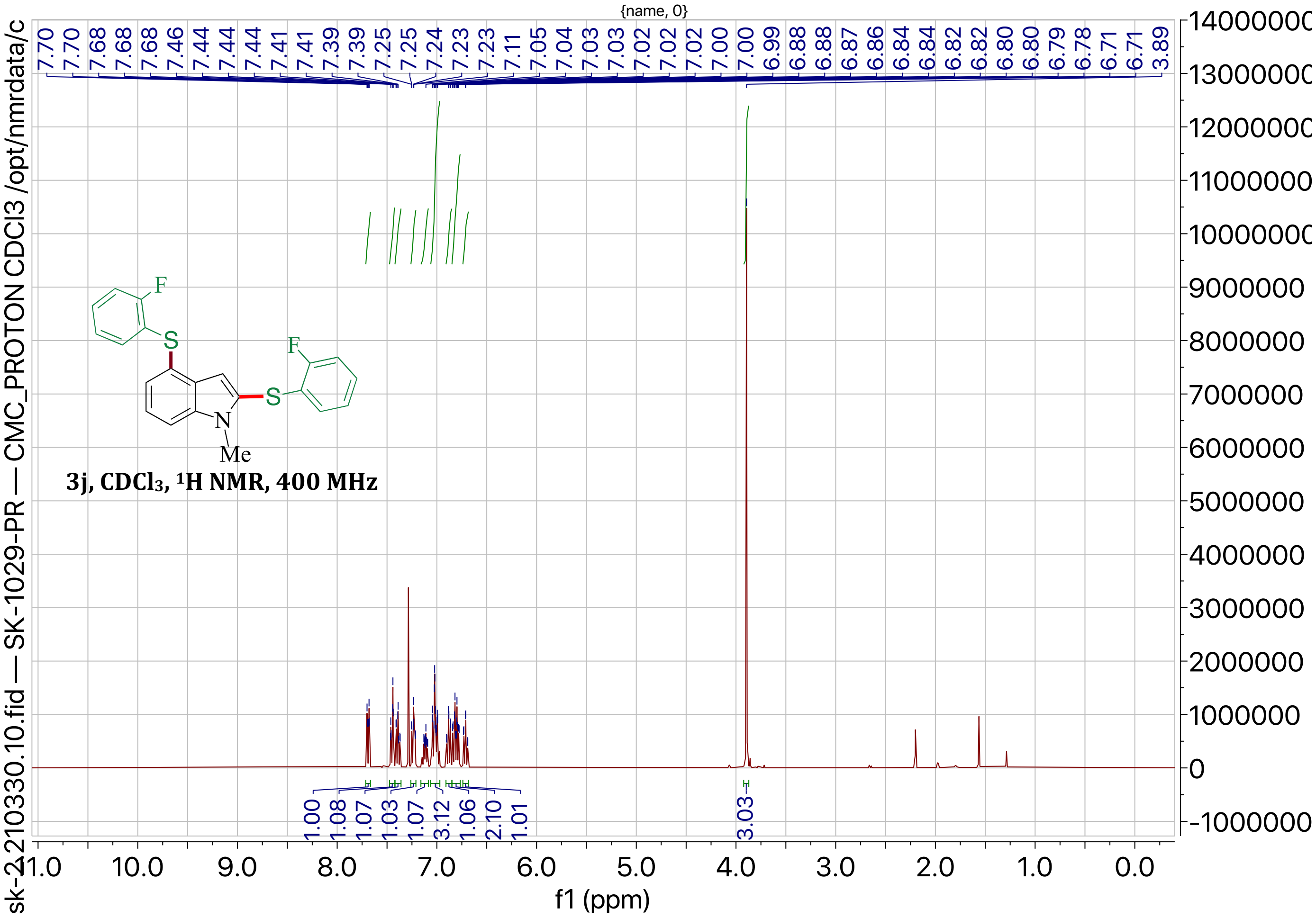

^{13}C{^{1}H} NMR spectrum of 3j

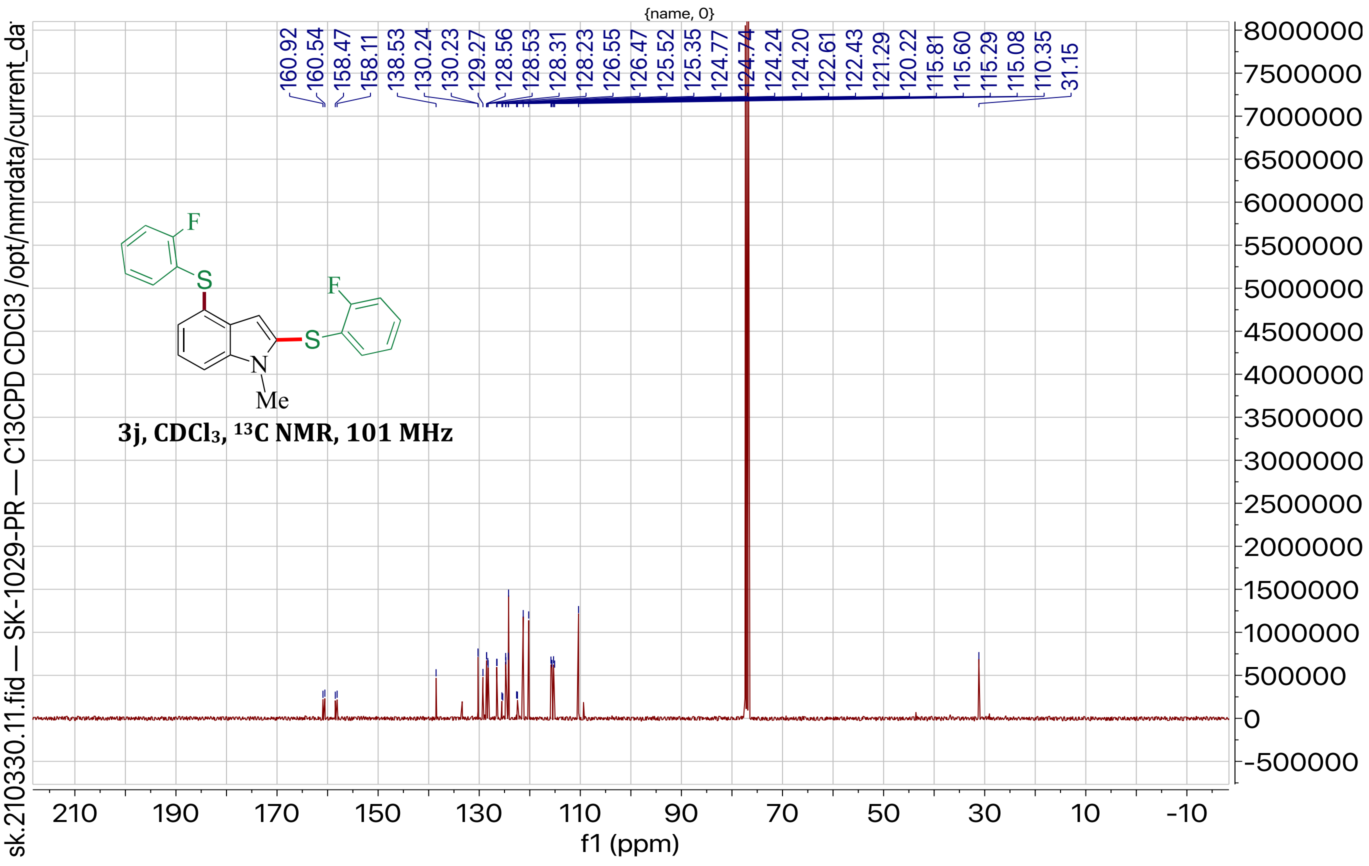

^{19}F NMR spectrum of 3j

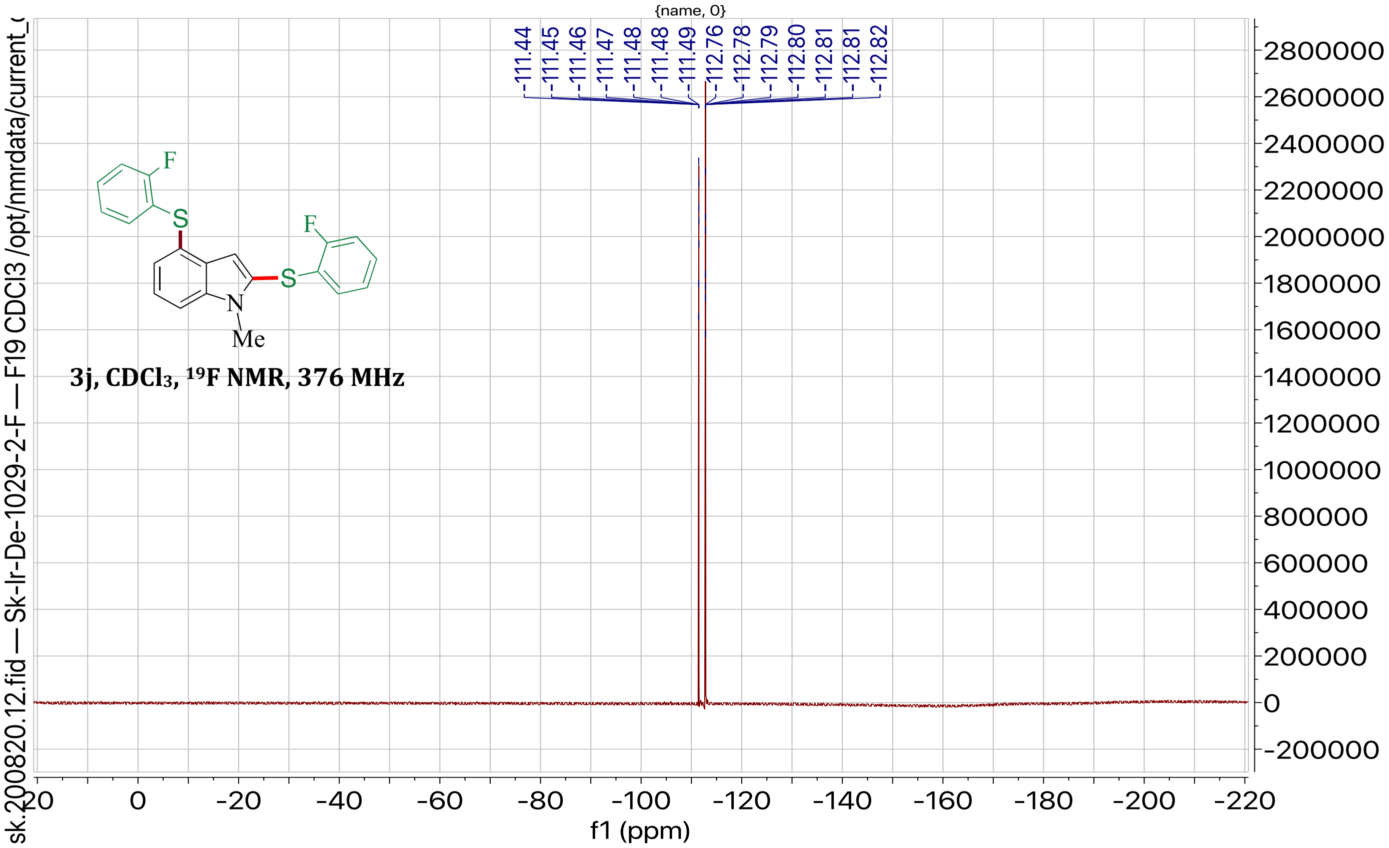

HRMS spectrum of 3j

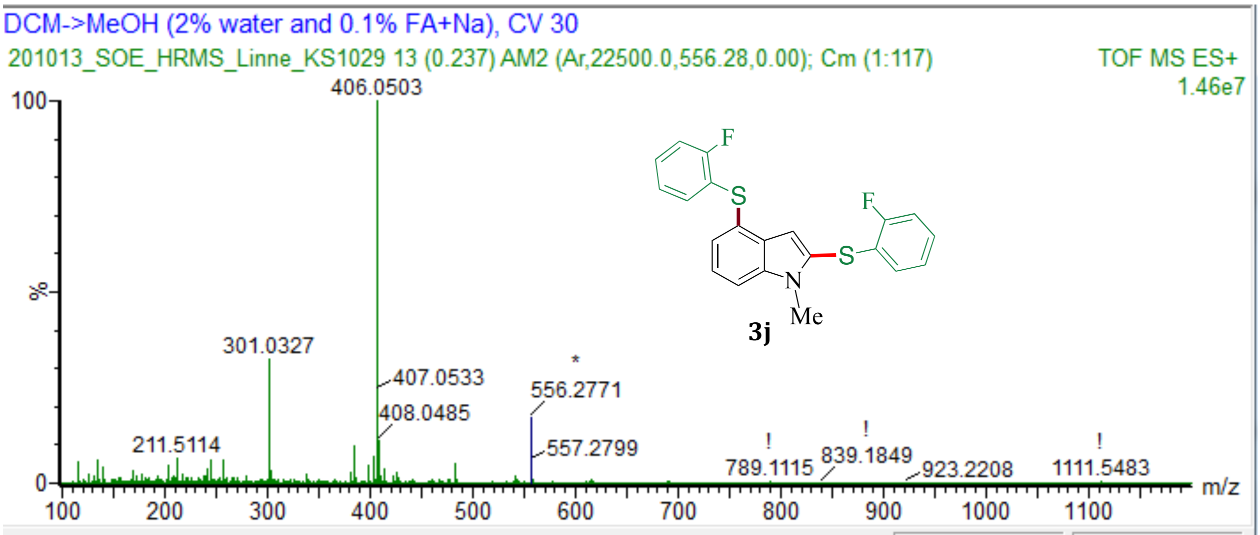

Single Mass Analysis

Tolerance = 2.0 mDa / DBE: min = -0.5, max = 100.0
Element prediction: Off
Number of isotope peaks used for i-FIT = 3

Monoisotopic Mass, Even Electron Ions
294 formula(e) evaluated with 2 results within limits (all results (up to 1000) for each mass)
Elements Used:
C: 0-50 H: 0-50 N: 0-2 O: 0-3 F: 2-2 Na: 0-1
S: 0-2

Mass	Calc. Mass	mDa	PPM	DBE	Formula	i.	Fit Conf %	C	H	N	O.	F	Na	S
406.0503	406.0502	0.1	0.2	21.5	C26 H10 N F2 S	61...0.00		26	10	1		2		1
	406.0512	-0.9	-2.2	13.5	C21 H15 N F2 Na S2	60...100.00		21	15	1		2	1	2

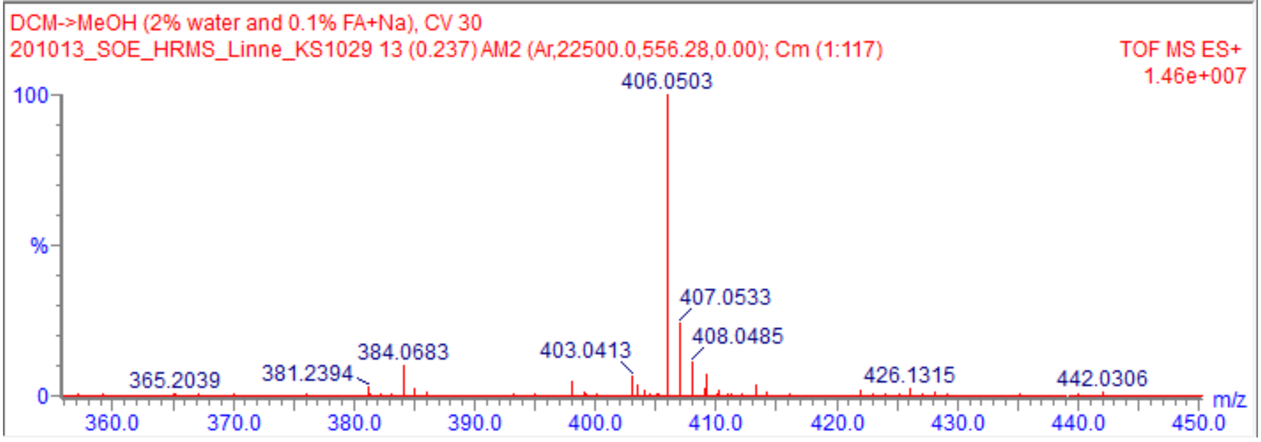

^{1}H NMR spectrum of 3k

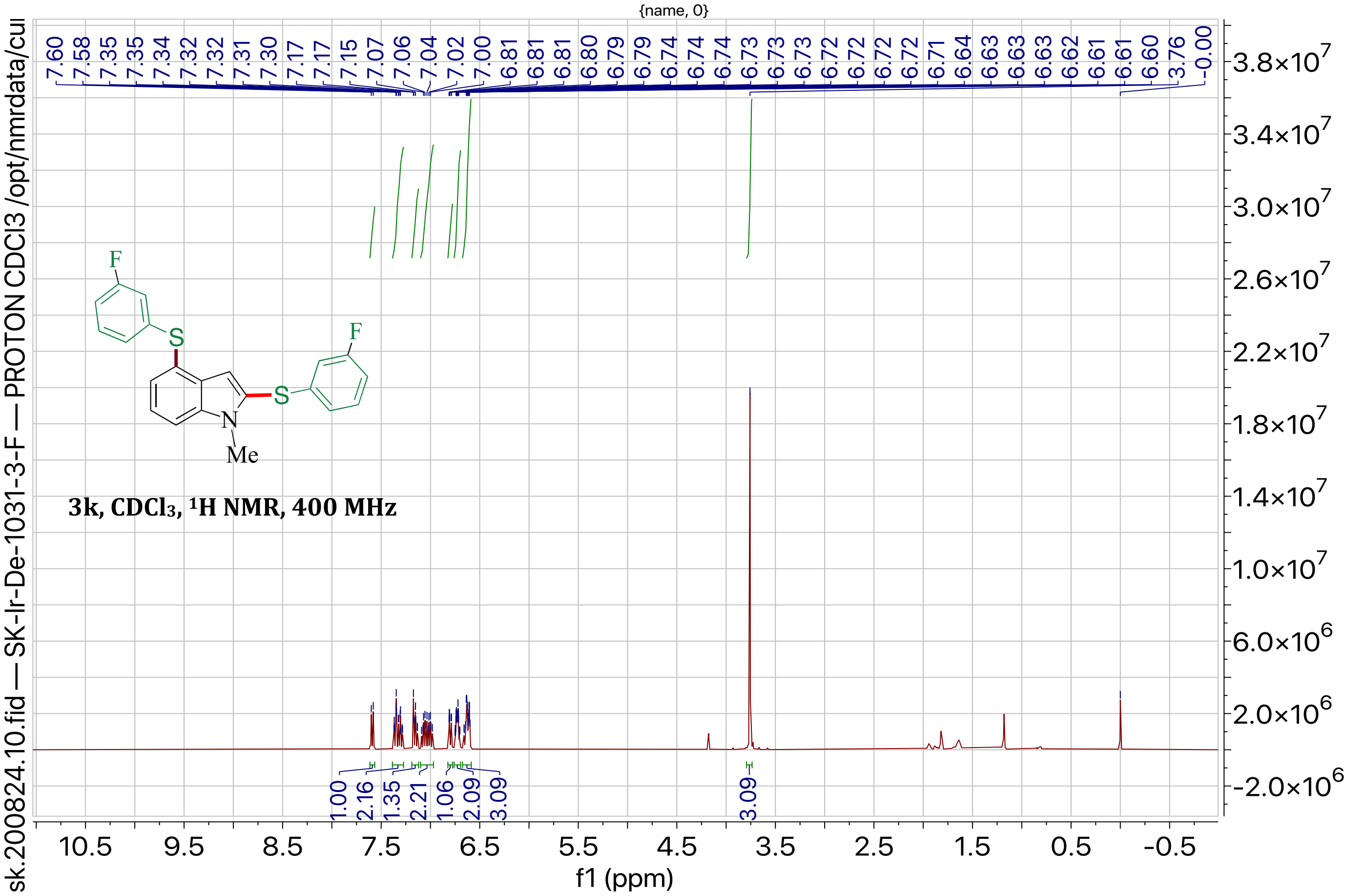

^{13}C{^{1}H} NMR spectrum of 3k

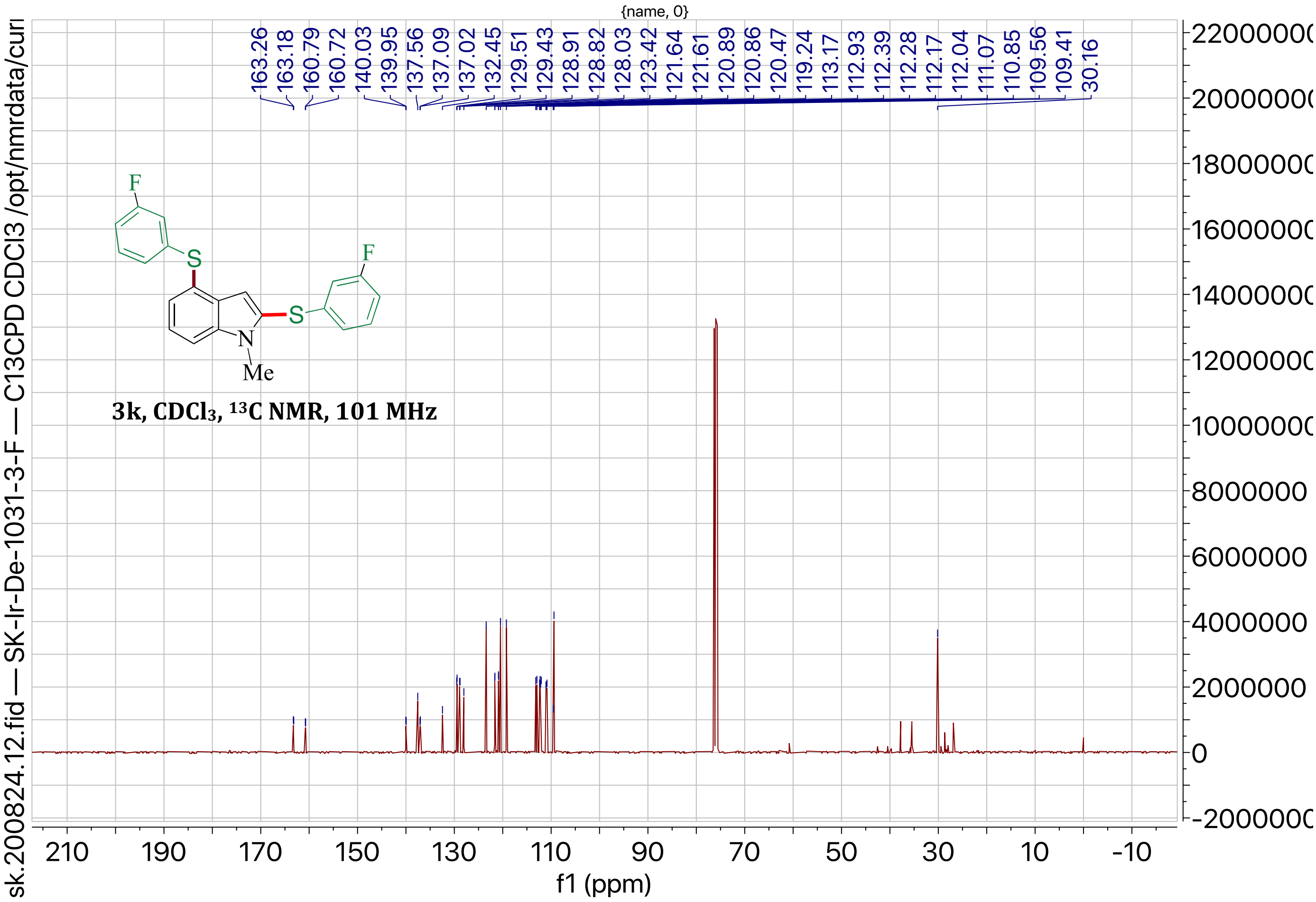

^{19}F NMR spectrum of 3k

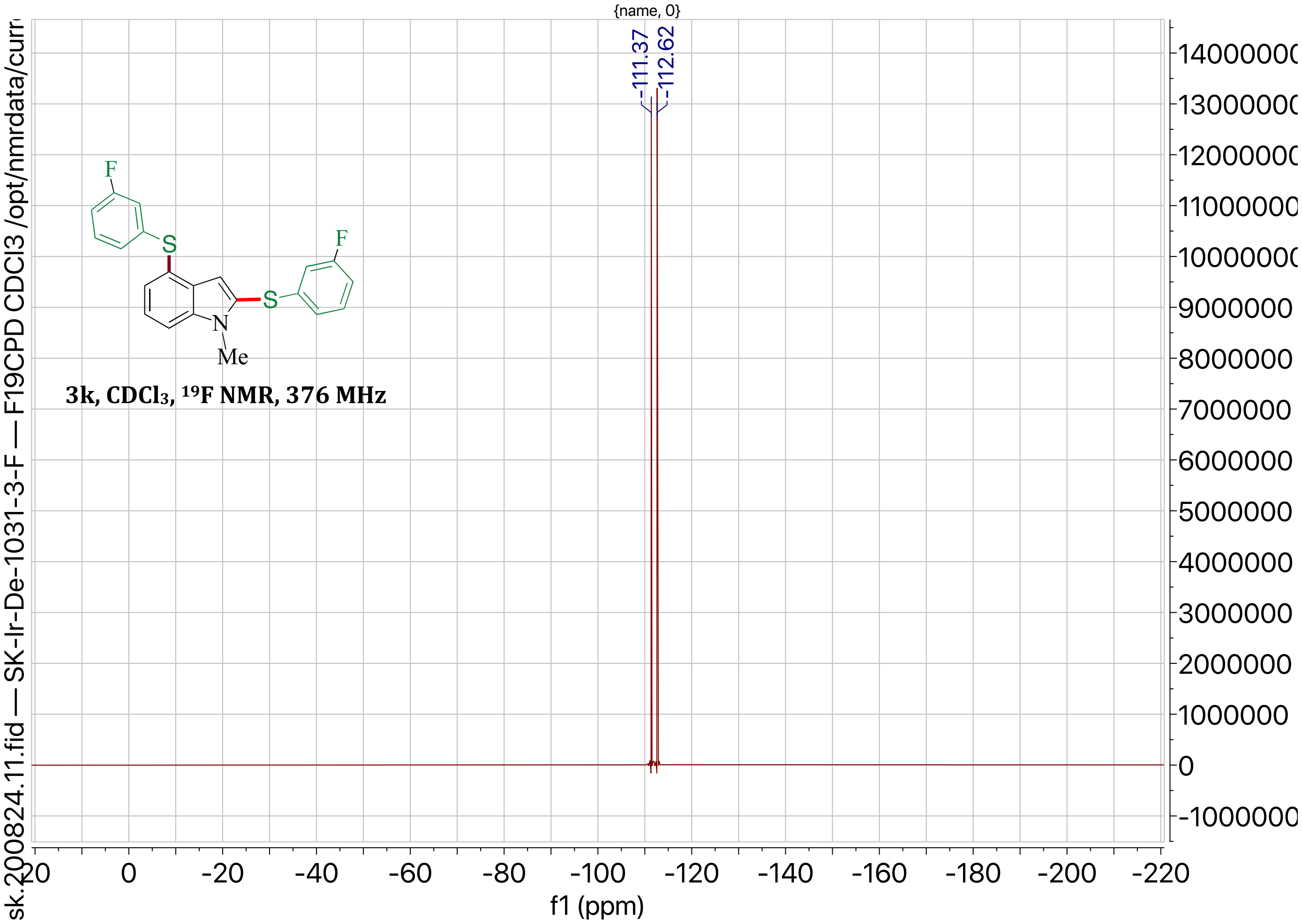

HRMS spectrum of 3k

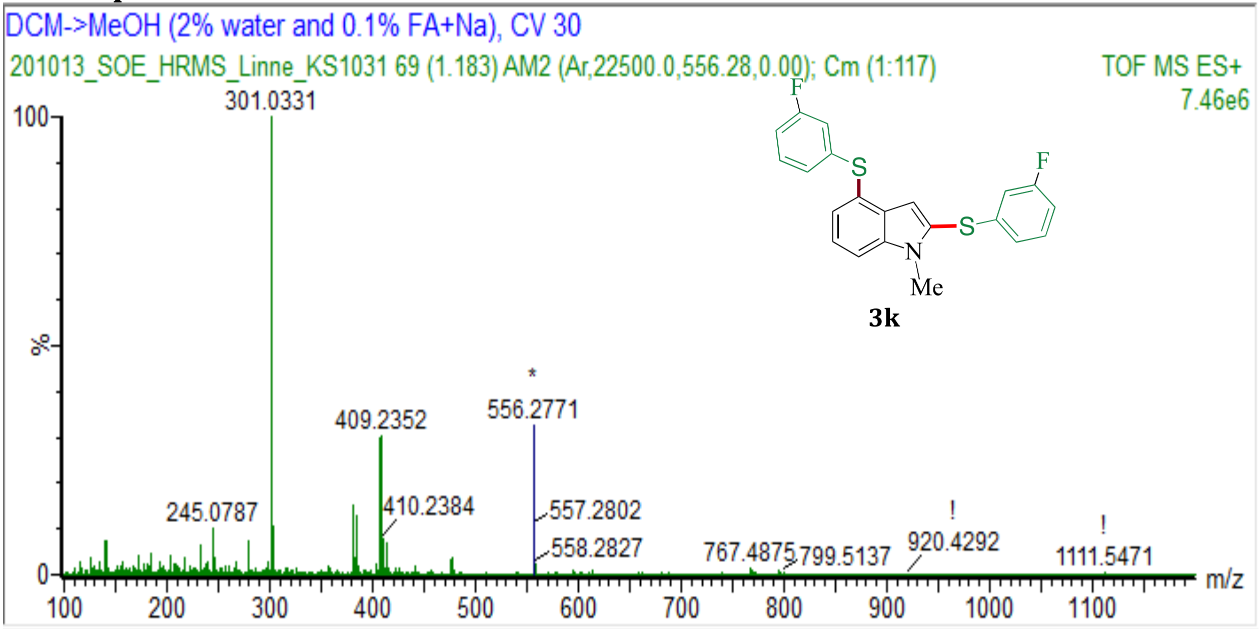

Single Mass Analysis

Tolerance = 2.0 mDa / DBE: min = -0.5, max = 100.0

Element prediction: Off

Number of isotope peaks used for i-FIT = 3

Monoisotopic Mass, Even Electron Ions

280 formula(e) evaluated with 2 results within limits (all results (up to 1000) for each mass)

Elements Used:

C: 0-50 H: 0-50 N: 0-2 O: 0-3 F: 2-2 Na: 0-1

S: 0-2

Mass	Calc. Mass	mDa	PPM	DBE	Formula	i...	Fit Conf %	C	H	N	O.	F	Na	S
384.0685	384.0692	-0.7	-1.8	13.5	C21 H16 N F2 S2	0...	94.58	21	16	1		2		2
	384.0668	1.7	4.4	10.5	C19 H17 N F2 Na S2	2...	5.42	19	17	1		2	1	2

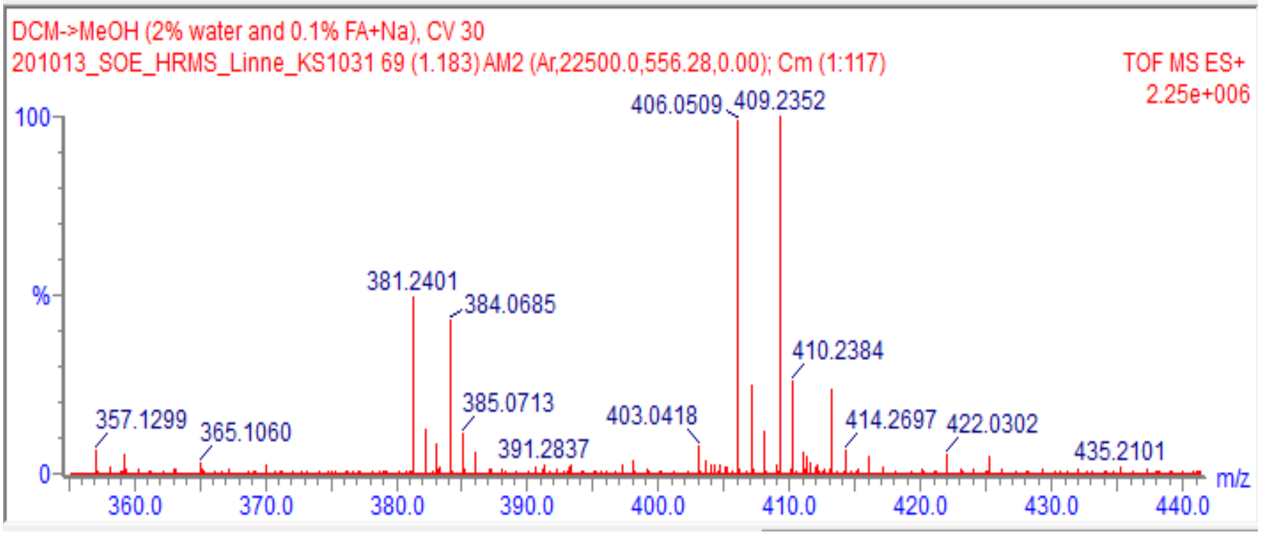

^{1}H NMR spectrum of 3l

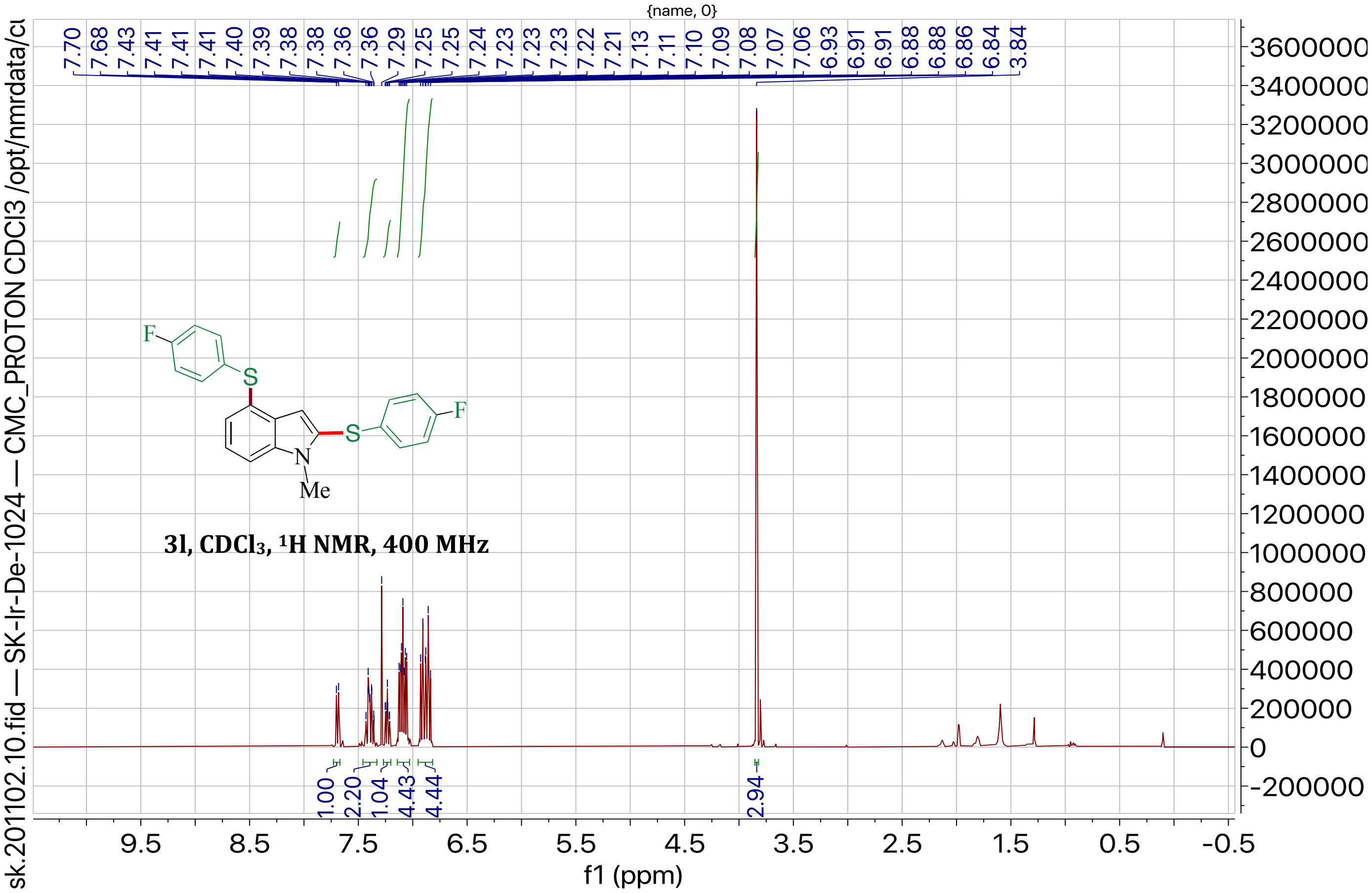

^{13}C{^{1}H} NMR spectrum of 3l

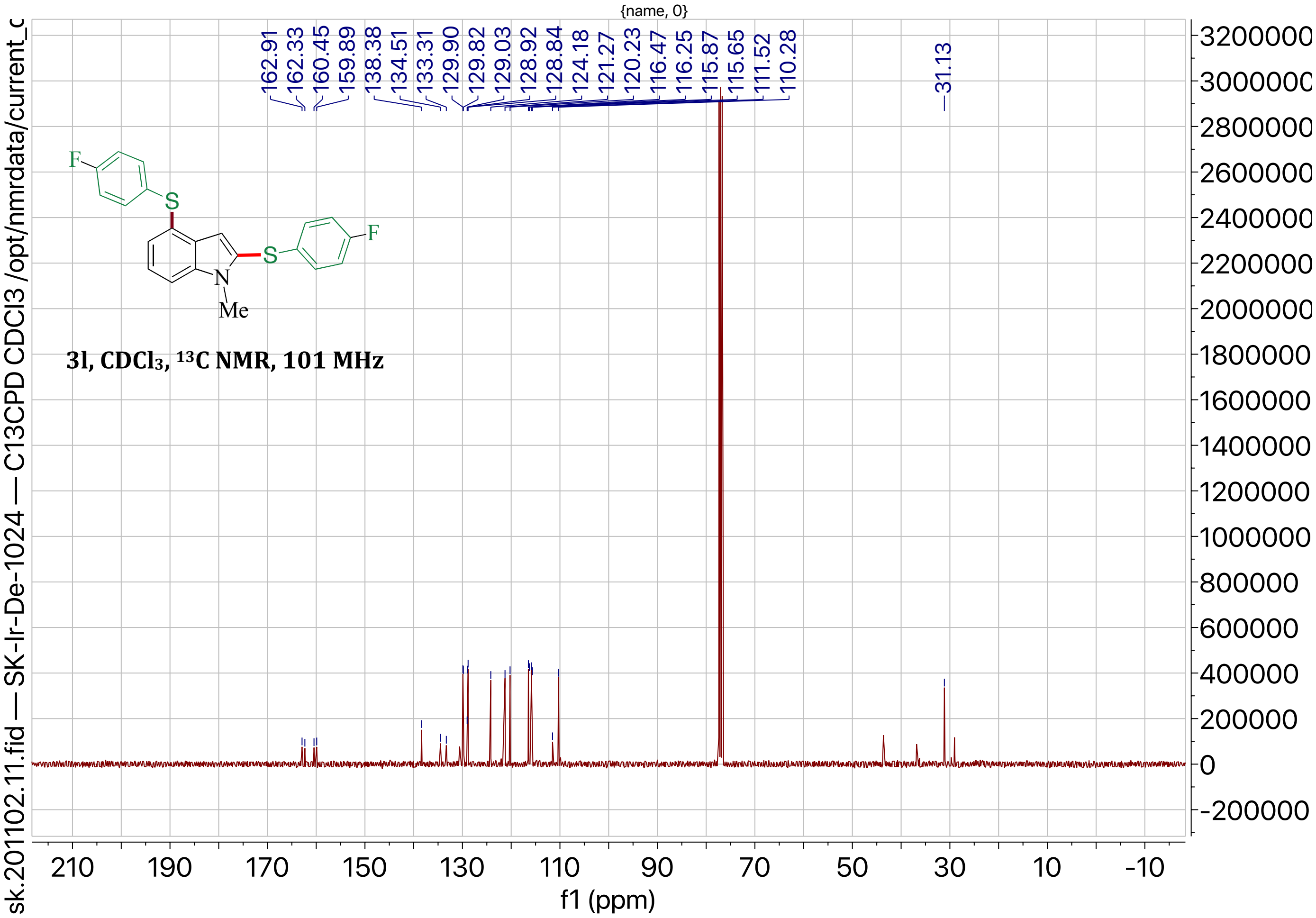

^{19}F NMR spectrum of 3l

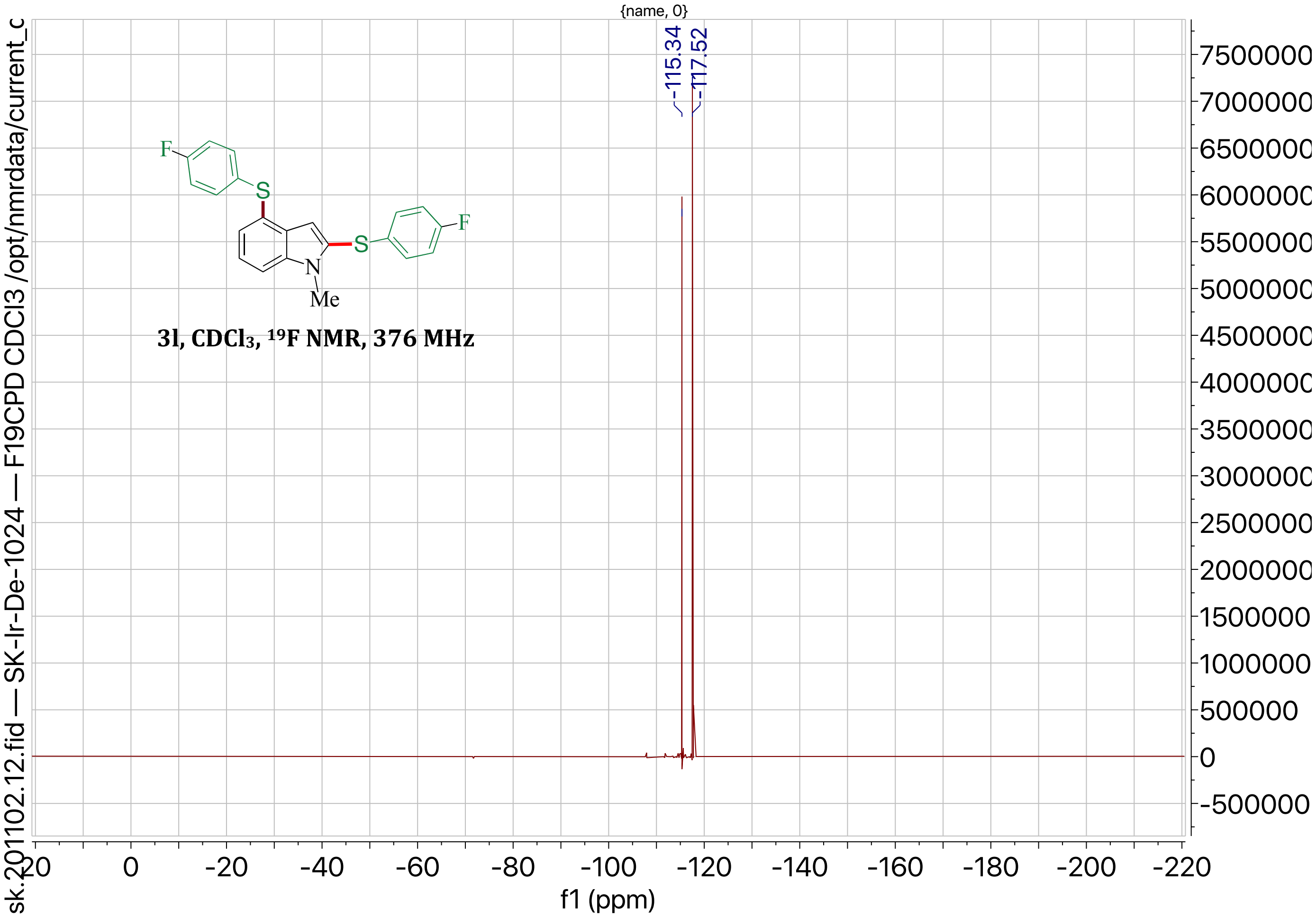

HRMS spectrum of 3l

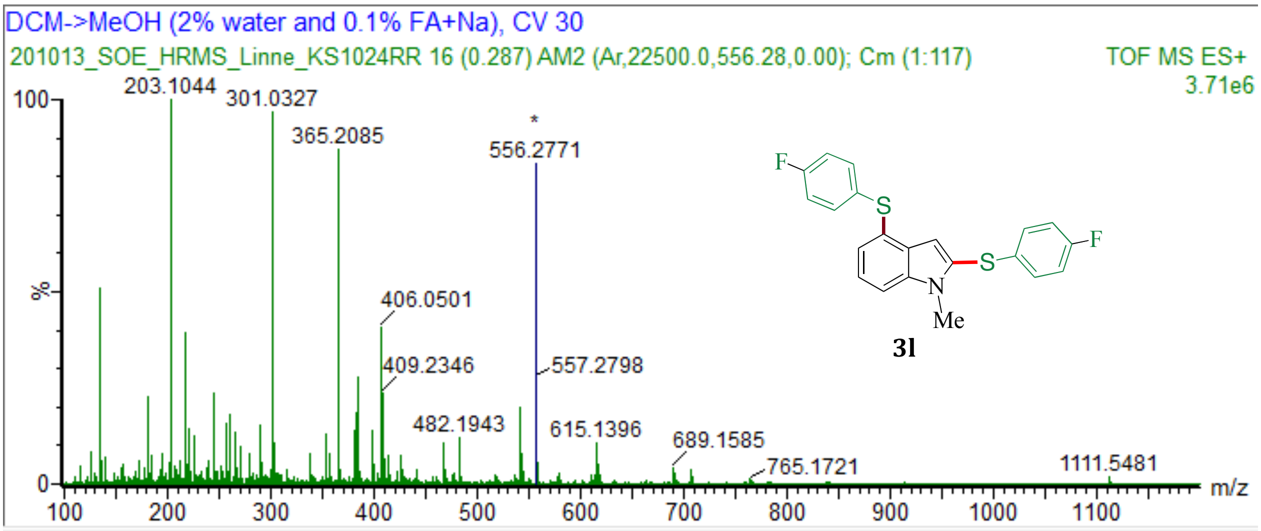

Single Mass Analysis

Tolerance = 2.0 mDa / DBE: min = -0.5, max = 100.0

Element prediction: Off

Number of isotope peaks used for i-FIT = 3

Monoisotopic Mass, Even Electron Ions

294 formula(e) evaluated with 2 results within limits (all results (up to 1000) for each mass)

Elements Used:

C: 0-50 H: 0-50 N: 0-2 O: 0-3 F: 2-2 Na: 0-1

S: 0-2

Mass	Calc. Mass	mDa	PPM	DBE	Formula	i..	Fit Conf %	C	H	N	O.	F	Na	S
406.0501	406.0502	-0.1	-0.2	21.5	C26 H10 N F2 S	[illegible]	0.00	26	10	1		2		1
	406.0512	-1.1	-2.7	13.5	C21 H15 N F2 Na S2	[illegible]	100.00	21	15	1		2	1	2

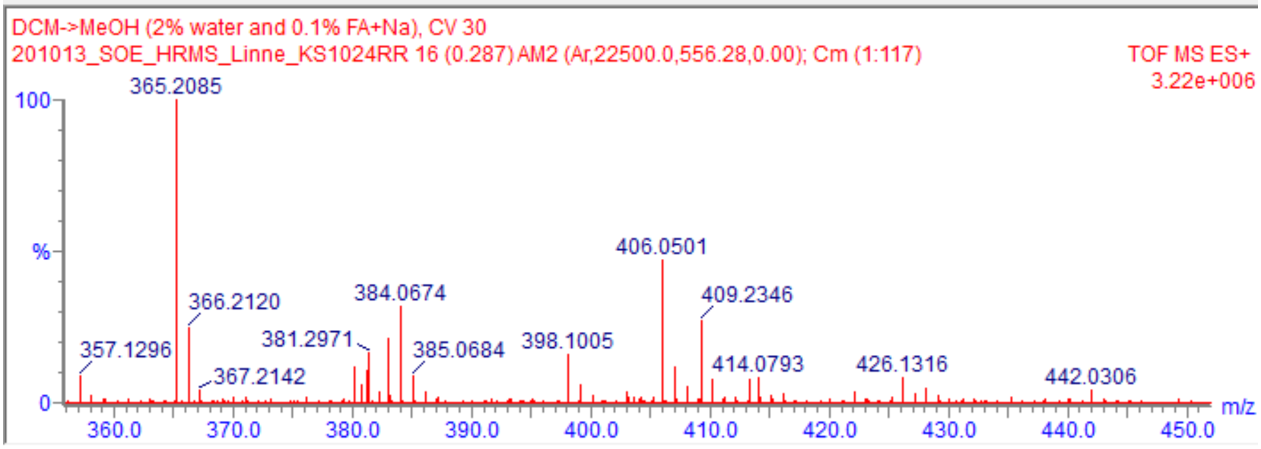

^{1}H NMR spectrum of 3m

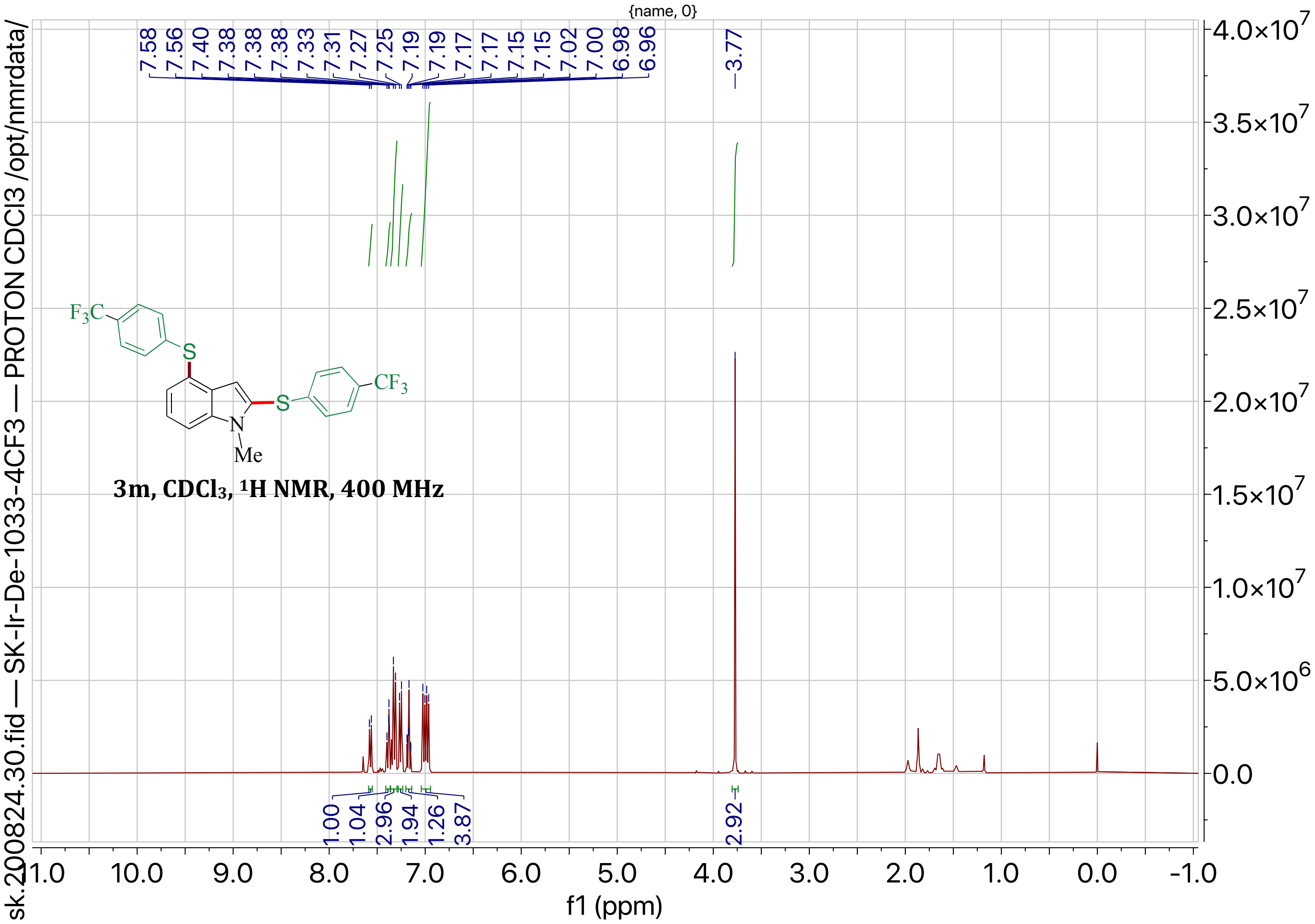

^{19}F NMR spectrum of 3m

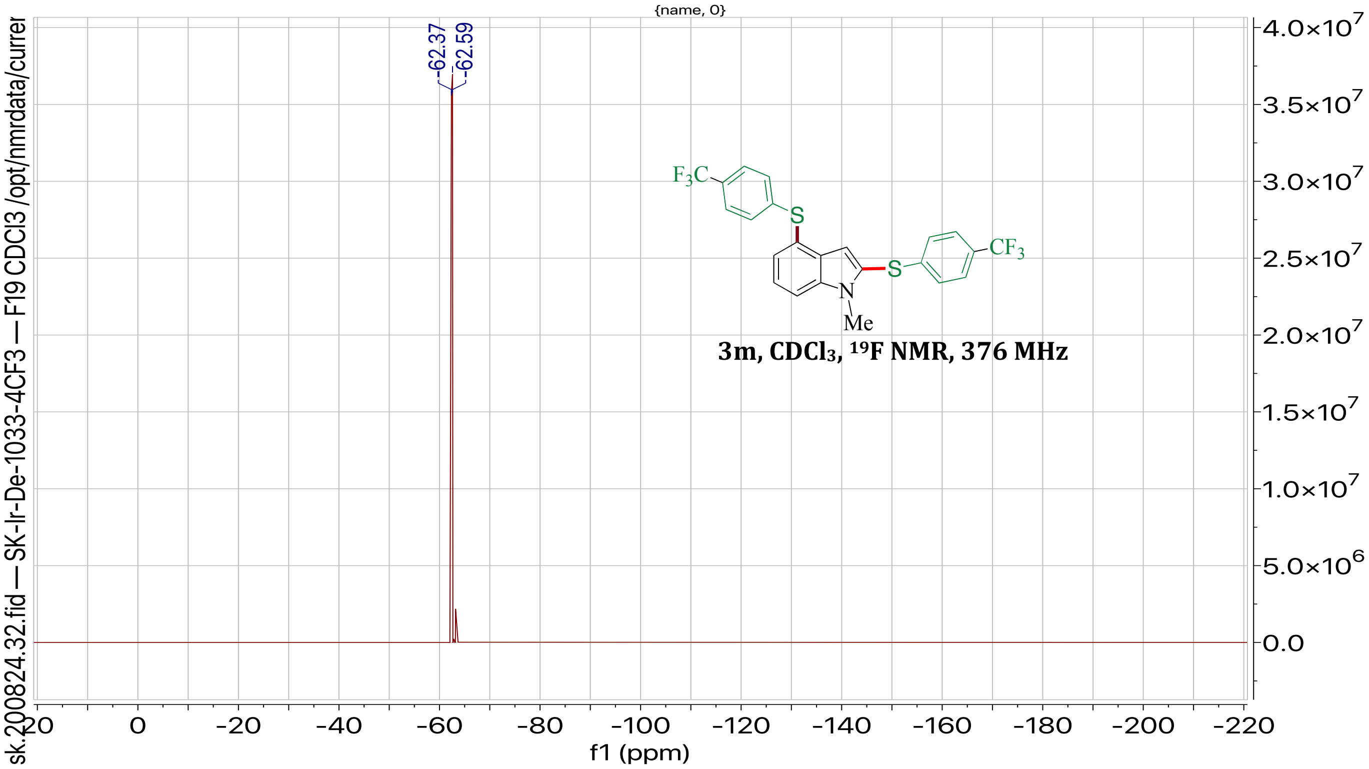

$^{13}C\{^{1}H\}$ NMR spectrum of 3m

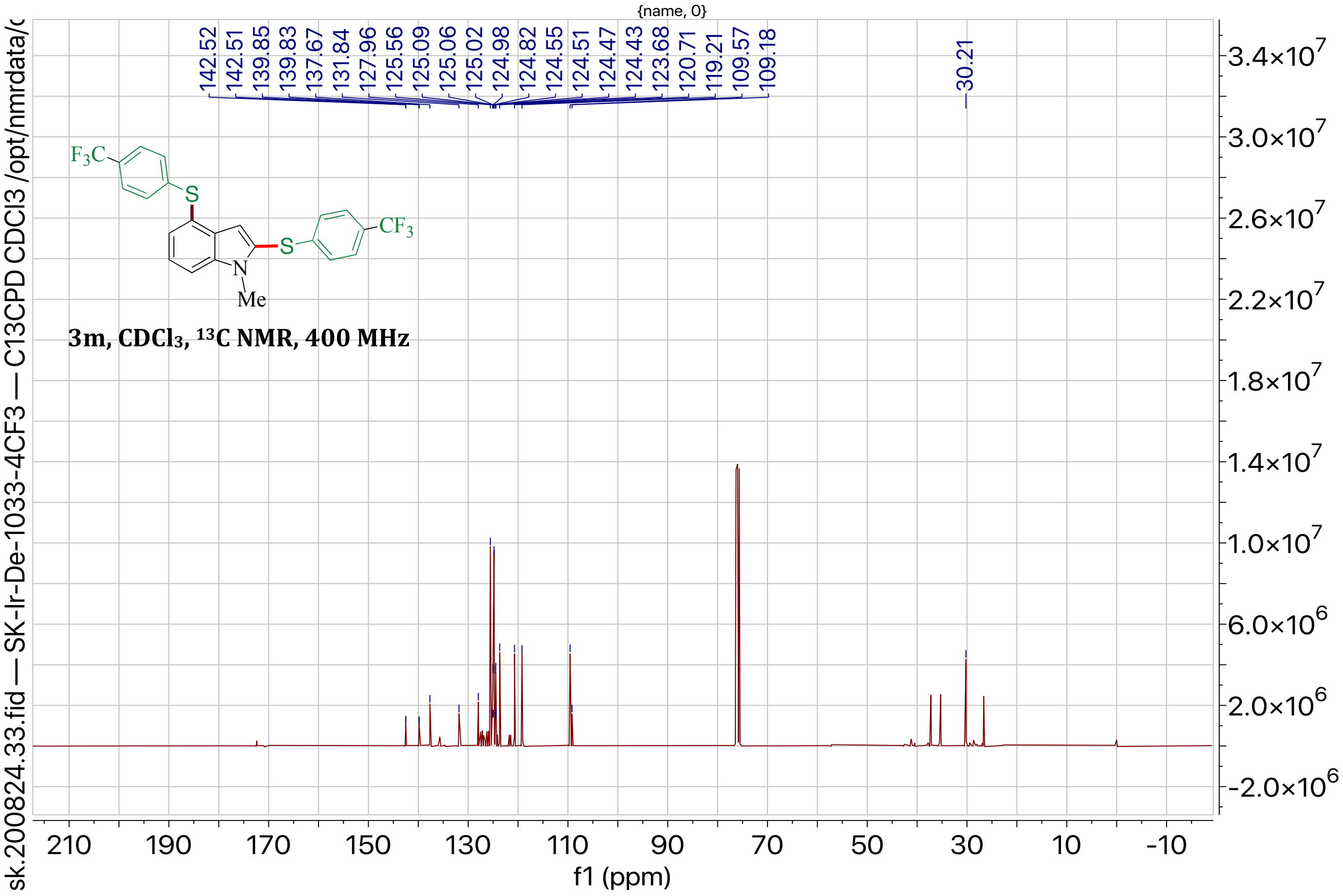

HRMS spectrum of 3m

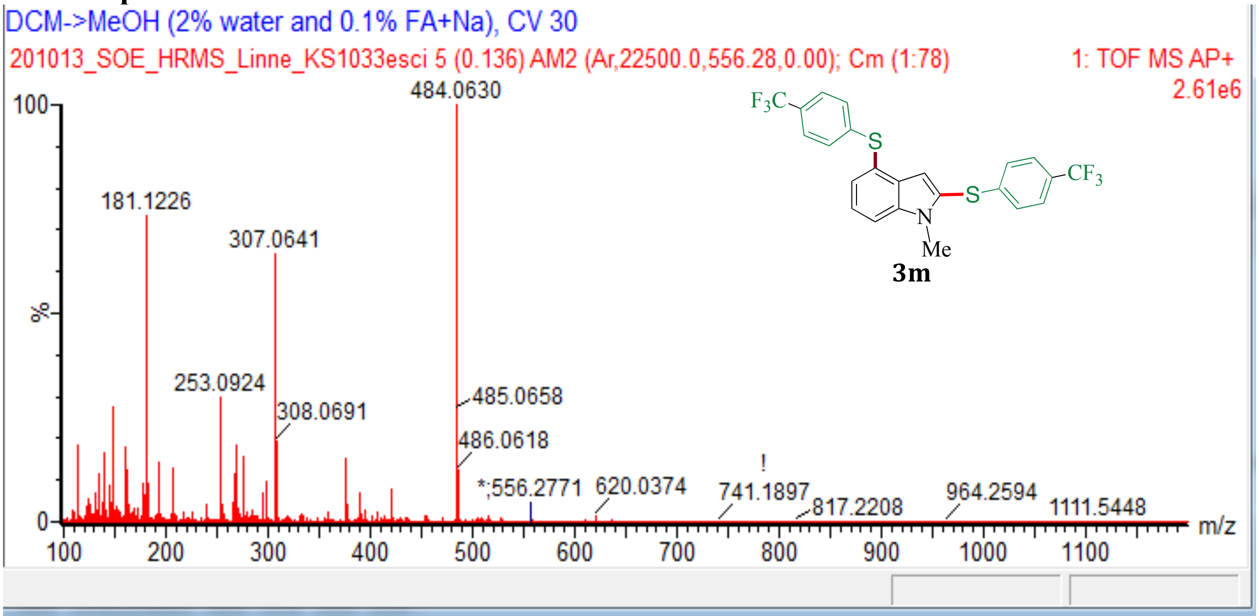

Single Mass Analysis

Tolerance = 2.0 mDa / DBE: min = -0.5, max = 100.0

Element prediction: Off

Number of isotope peaks used for i-FIT = 3

Monoisotopic Mass, Even Electron Ions

290 formula(e) evaluated with 1 results within limits (all results (up to 1000) for each mass)

Elements Used:

C: 0-50 H: 0-50 N: 0-2 O: 0-3 F: 6-6 Na: 0-1

S: 0-2

Mass	Calc. Mass	mDa	PPM	DBE	Formula	i..	Fit Conf %	C	H	N	O.	F	Na	S
484.0630	484.0628	0.2	0.4	13.5	C23 H16 N F6 S2	4n...	n/a	23	16	1		6		2

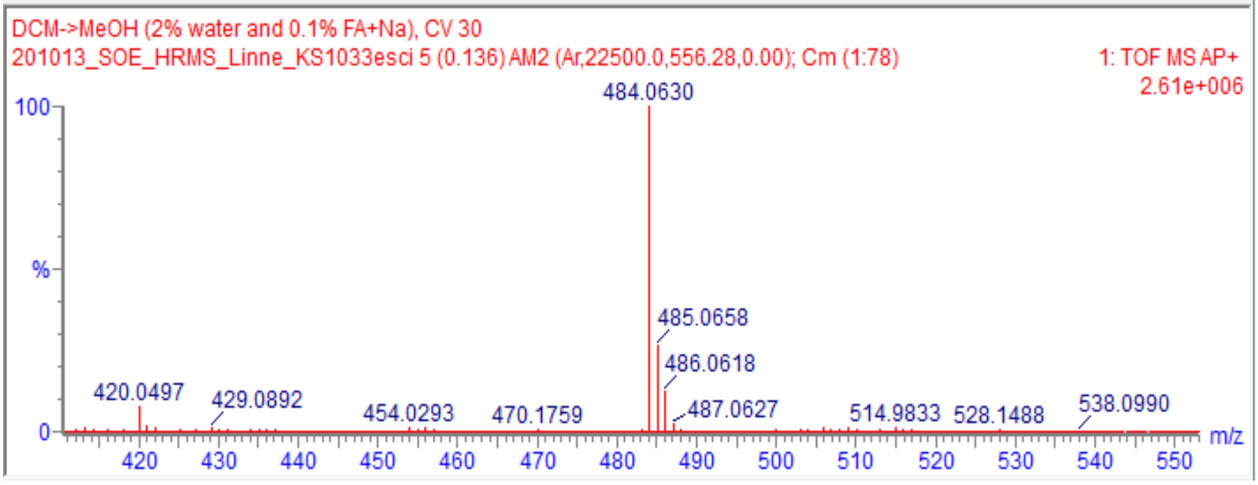

^1H NMR spectrum of 3n

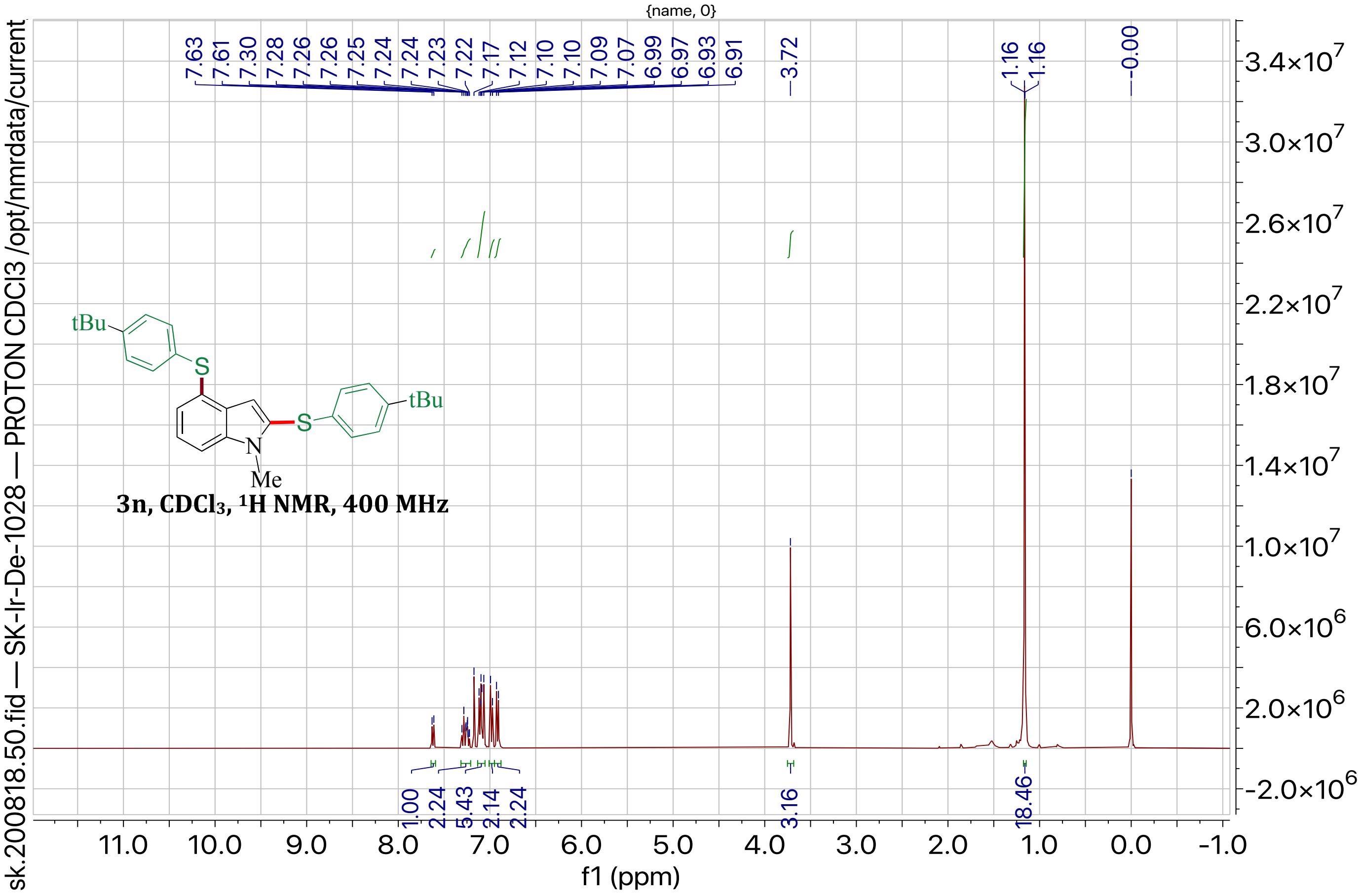

$^{13}C\{^1H\}$ NMR spectrum of 3n

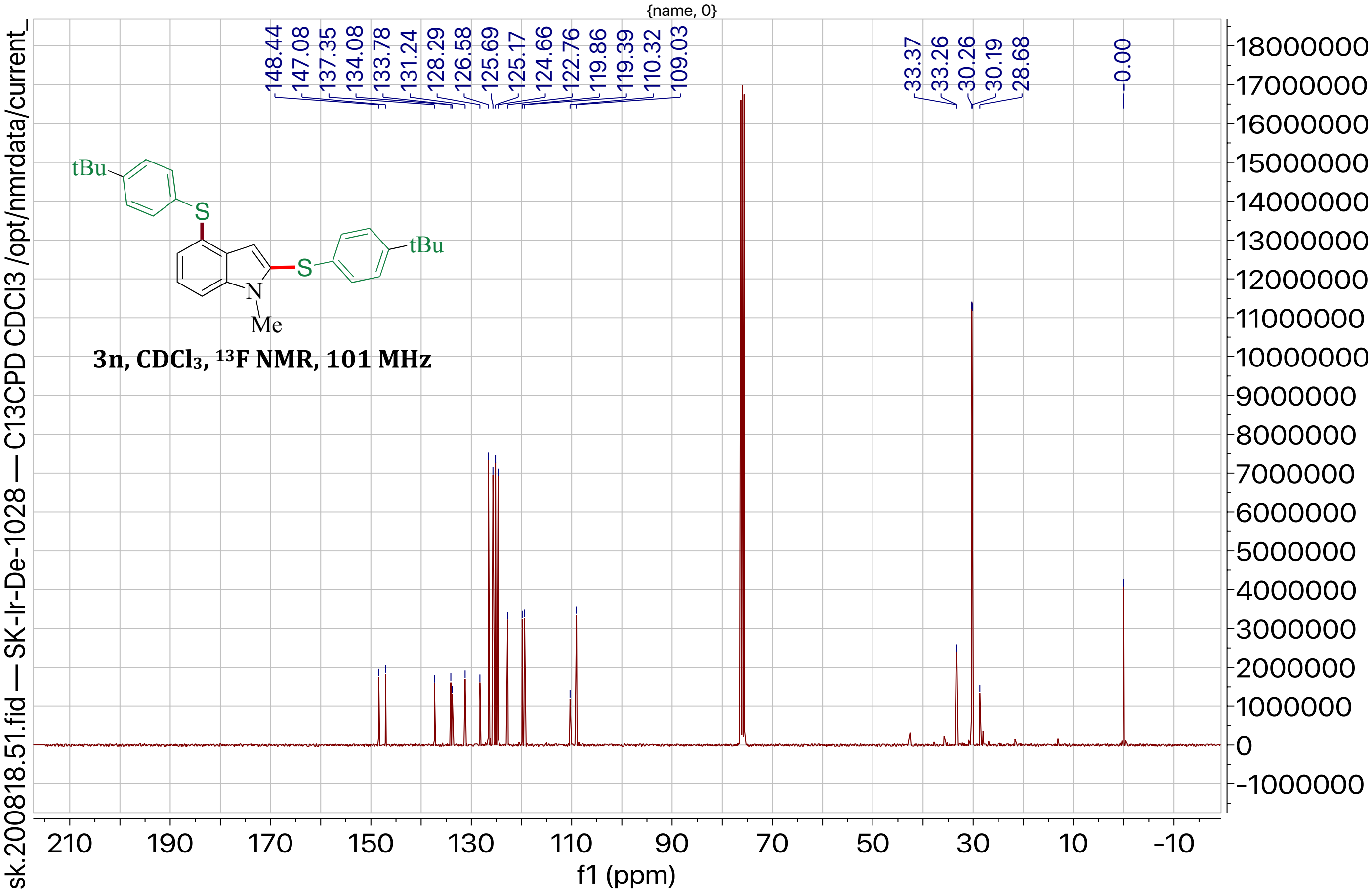

HRMS spectrum of 3n

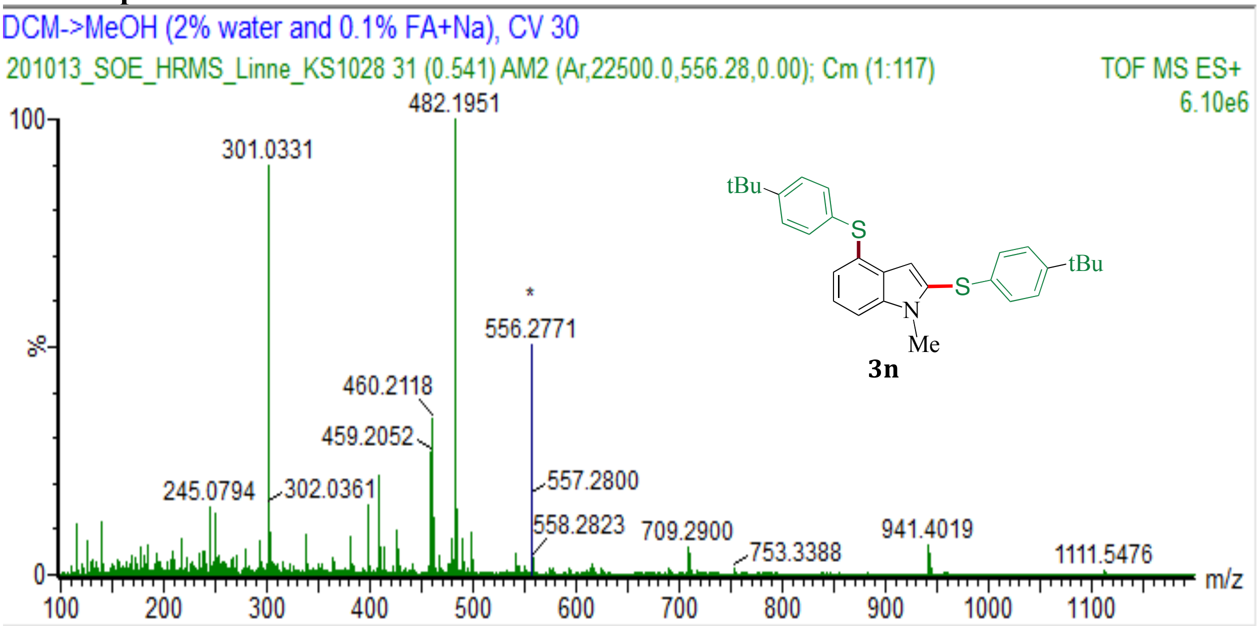

Single Mass Analysis

Tolerance = 2.0 mDa / DBE: min = -0.5, max = 100.0

Element prediction: Off

Number of isotope peaks used for i-FIT = 3

Monoisotopic Mass, Even Electron Ions

312 formula(e) evaluated with 2 results within limits (all results (up to 1000) for each mass)

Elements Used:

C: 0-50 H: 0-50 N: 0-2 O: 0-3 Na: 0-1 S: 0-2

Mass	Calc. Mass	mDa	PPM	DBE	Formula	Fit Conf %	C	H	N	O	Na	S
482.1951	482.1952	-0.1	-0.2	13.5	C29 H33 N Na S2	0.100.00	29	33	1		1	2
	482.1942	0.9	1.9	21.5	C34 H28 N S	1.0.00	34	28	1			1

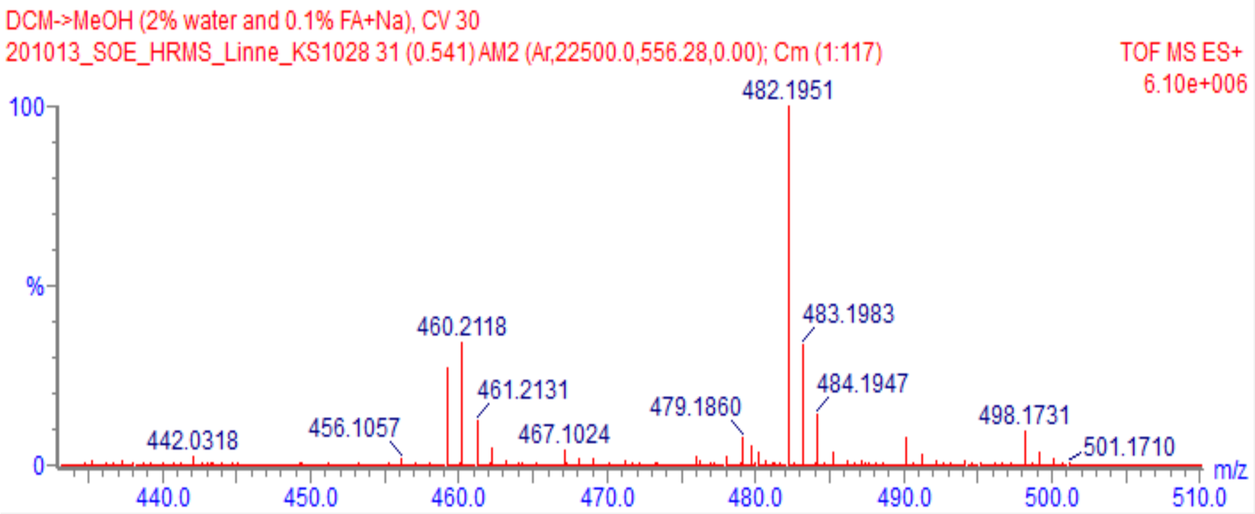

^{1}H NMR spectrum of 3o

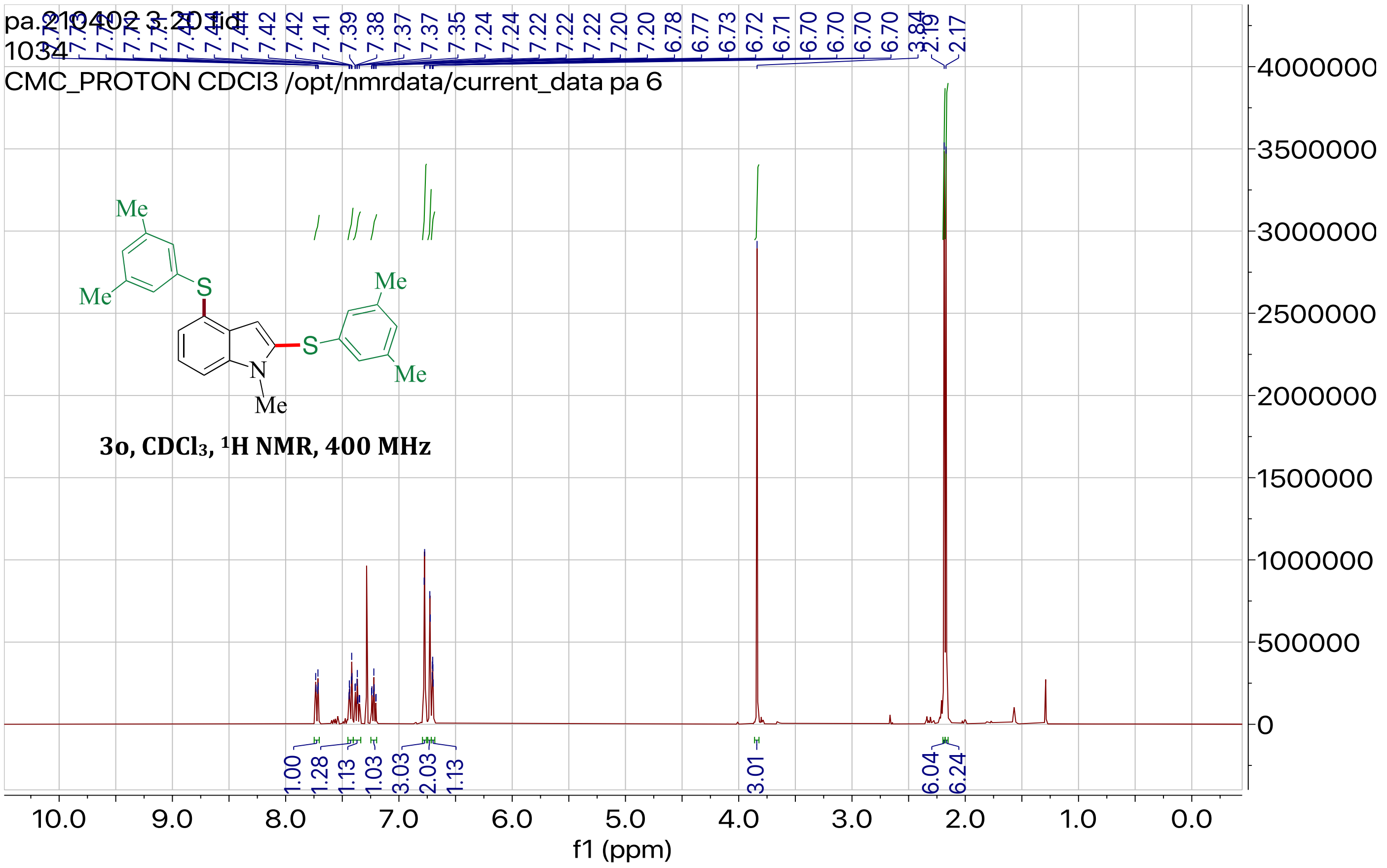

^{13}C{^{1}H} NMR spectrum of 3o

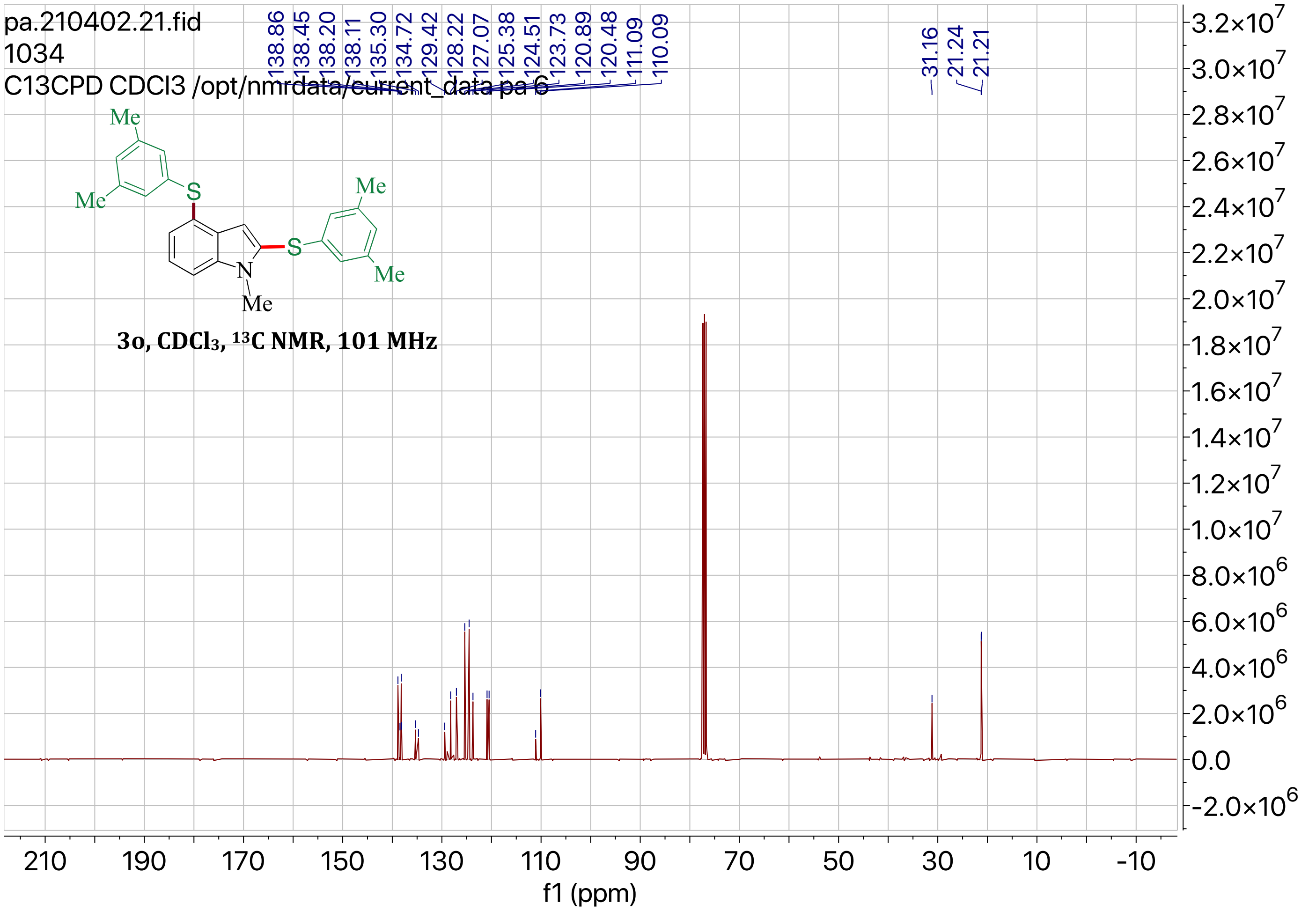

HRMS spectrum of 3o

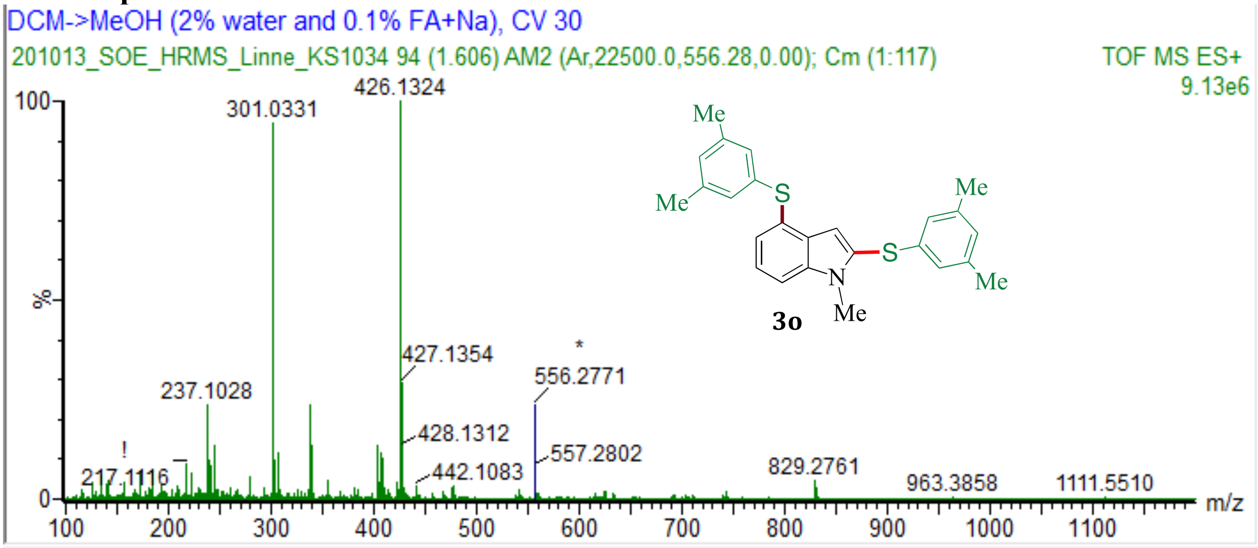

Single Mass Analysis
Tolerance = 2.0 mDa / DBE: min = -0.5, max = 100.0
Element prediction: Off
Number of isotope peaks used for i-FIT = 3
Monoisotopic Mass, Even Electron Ions
311 formula(e) evaluated with 2 results within limits (all results (up to 1000) for each mass)
Elements Used:
C: 0-50 H: 0-50 N: 0-2 O: 0-3 Na: 0-1 S: 0-2

Mass	Calc. Mass	mDa	PPM	DBE	Formula	i..	Fit Conf %	C	H	N	O	Na	S
426.1324	426.1326	-0.2	-0.5	13.5	C25 H25 N Na S2	60...	100.00	25	25	1		1	2
	426.1316	0.8	1.9	21.5	C30 H20 N S	61...	0.00	30	20	1			1

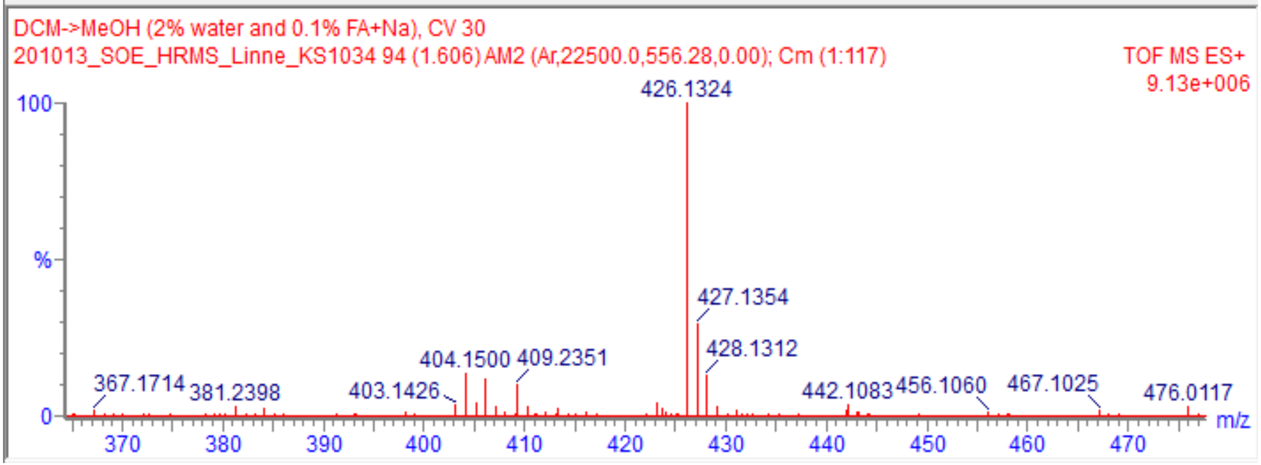

^{1}H NMR spectrum of 3p

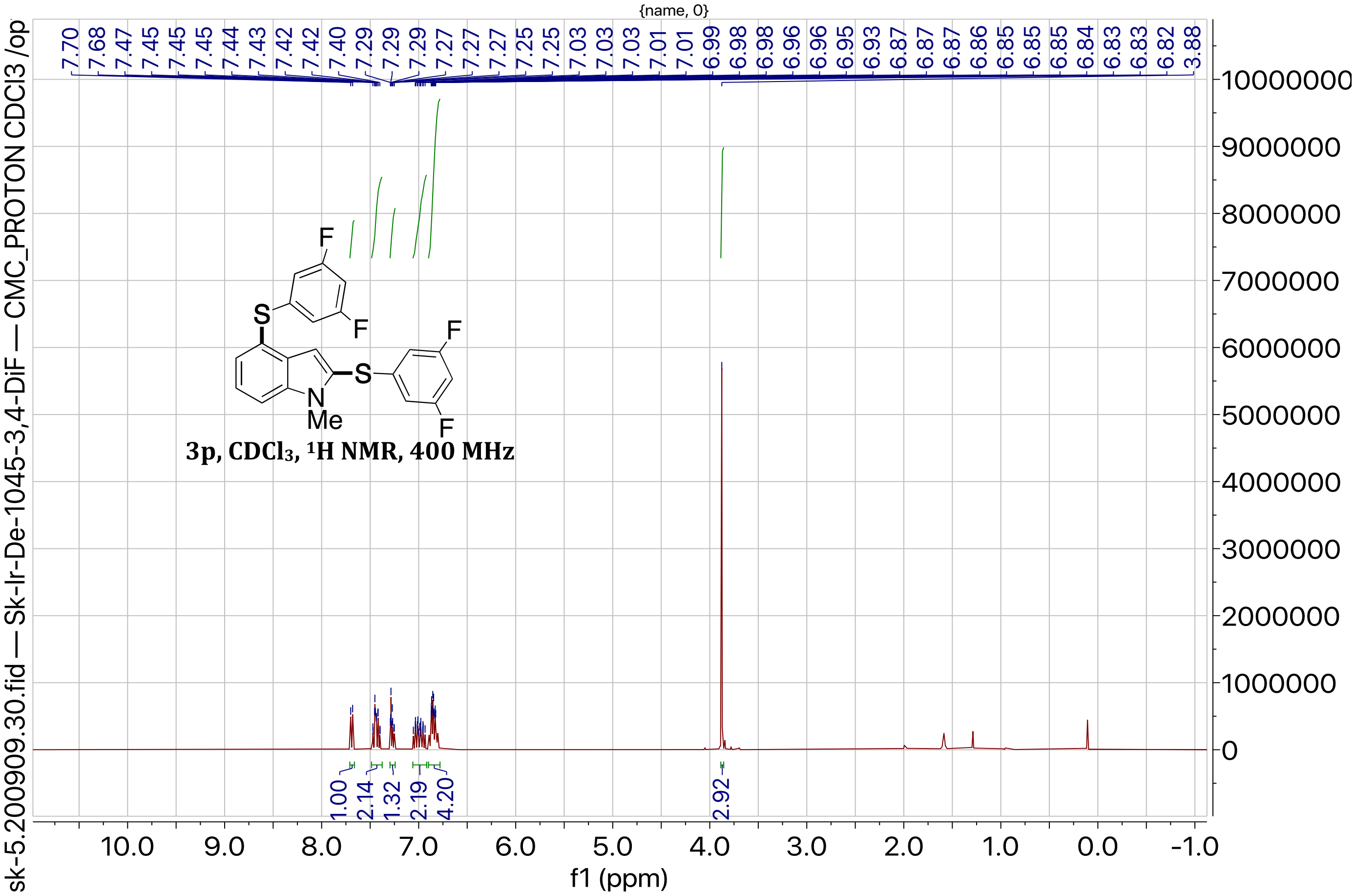

^{13}C{^{1}H} NMR spectrum of 3p

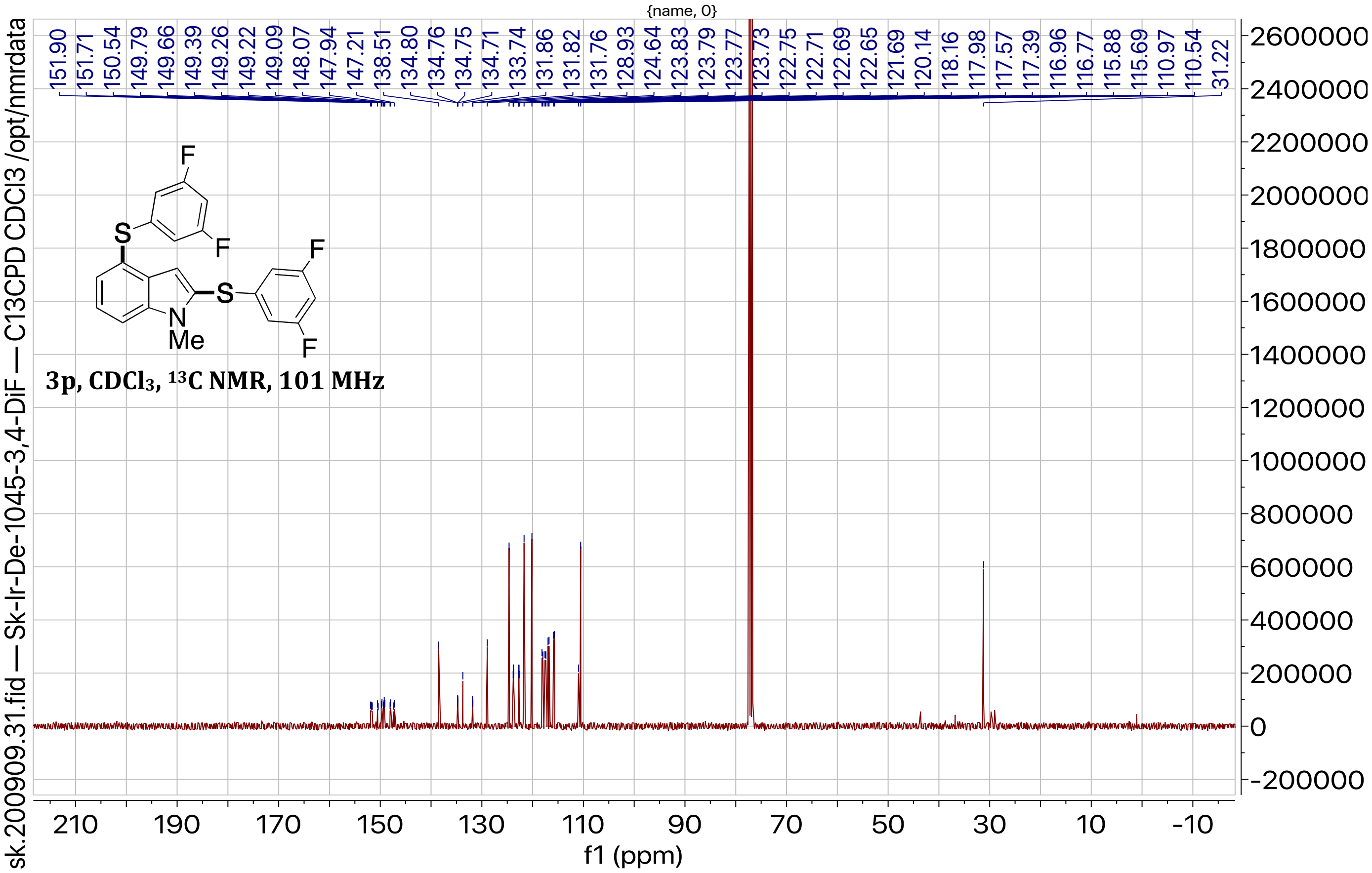

^{19}F NMR spectrum of 3p

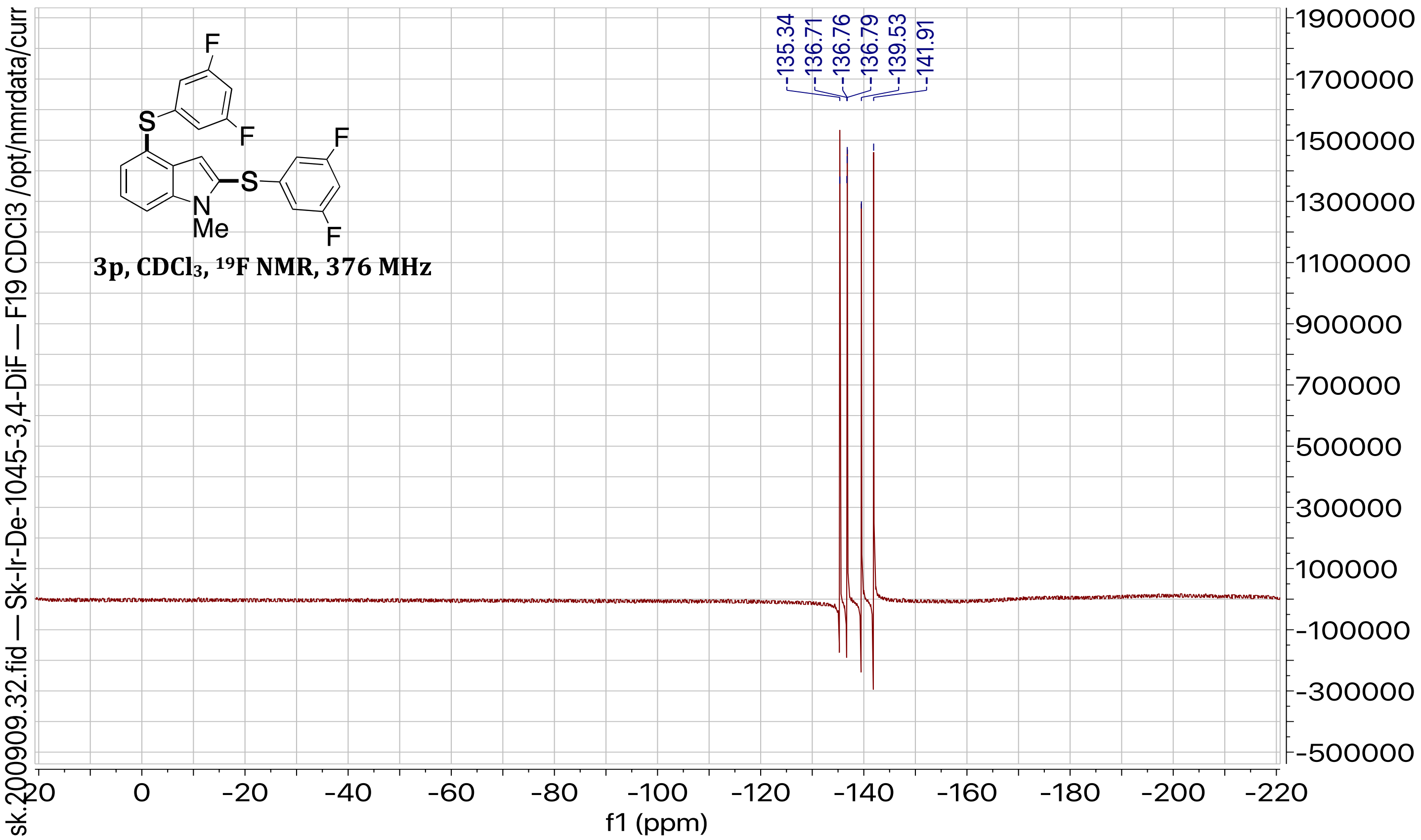

HRMS spectrum of 3p

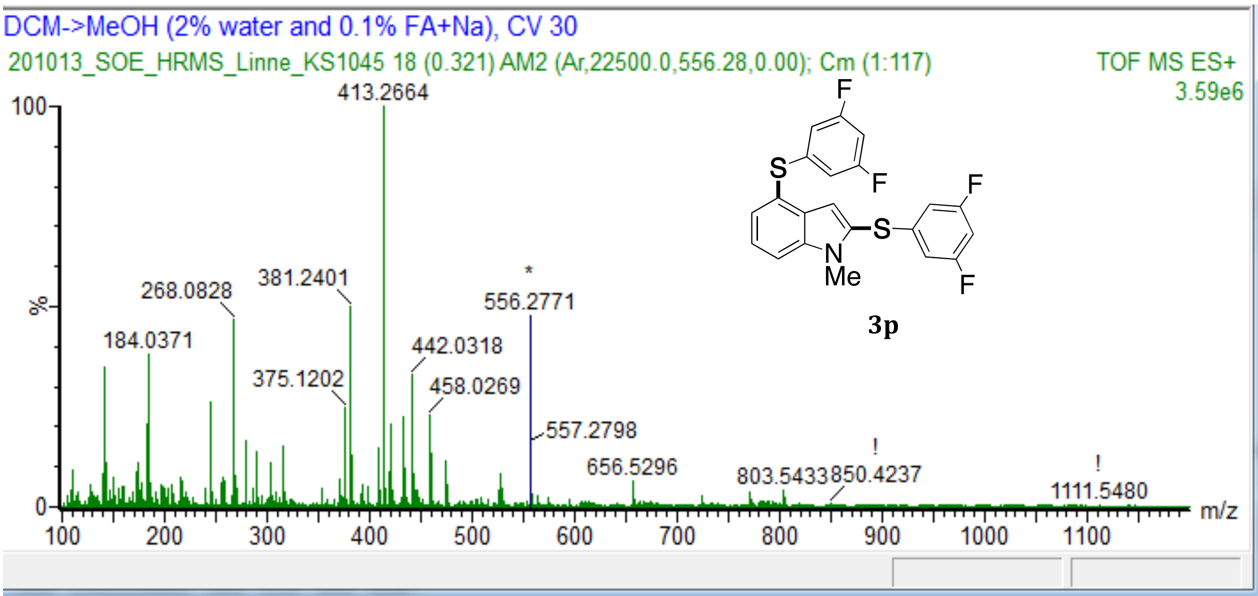

Single Mass Analysis

Tolerance = 2.0 mDa / DBE: min = -0.5, max = 100.0

Element prediction: Off

Number of isotope peaks used for i-FIT = 3

Monoisotopic Mass, Even Electron Ions

587 formula(e) evaluated with 2 results within limits (all results (up to 1000) for each mass)

Elements Used:

C: 0-50 H: 0-50 N: 0-2 O: 0-3 F: 3-4 Na: 0-1

S: 0-2

Mass	Calc. Mass	mDa	PPM	DBE	Formula	i...	Fit Conf %	C	H	N	O	F	Na	S
442.0318	442.0314	0.4	0.9	21.5	C26 H8 N F4 S	51...	0.00	26	8	1		4		1
	442.0323	-0.5	-1.1	13.5	C21 H13 N F4 Na S2	50...	100.00	21	13	1		4	1	2

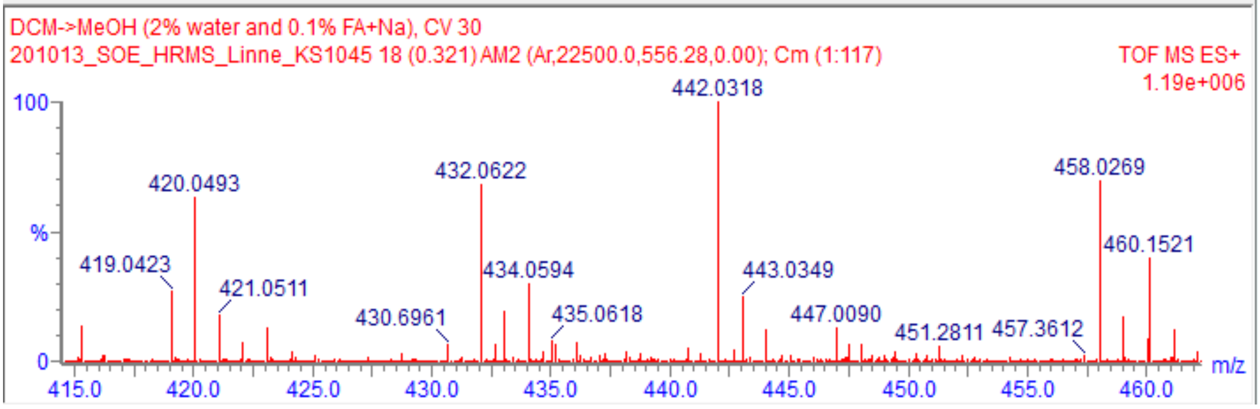

^{1}H NMR spectrum of 3q

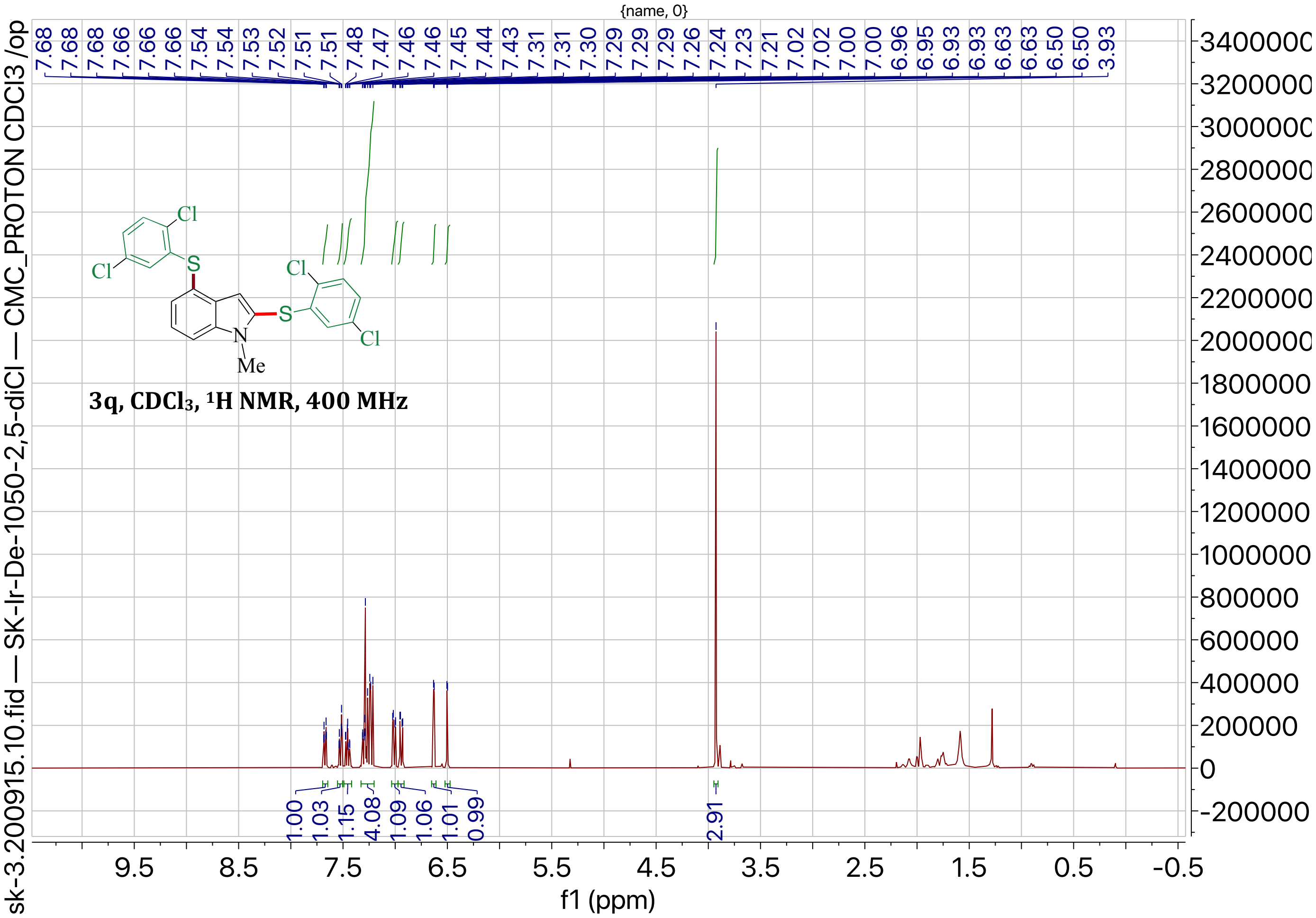

$^{13}C\{^{1}H\}$ NMR spectrum of 3q

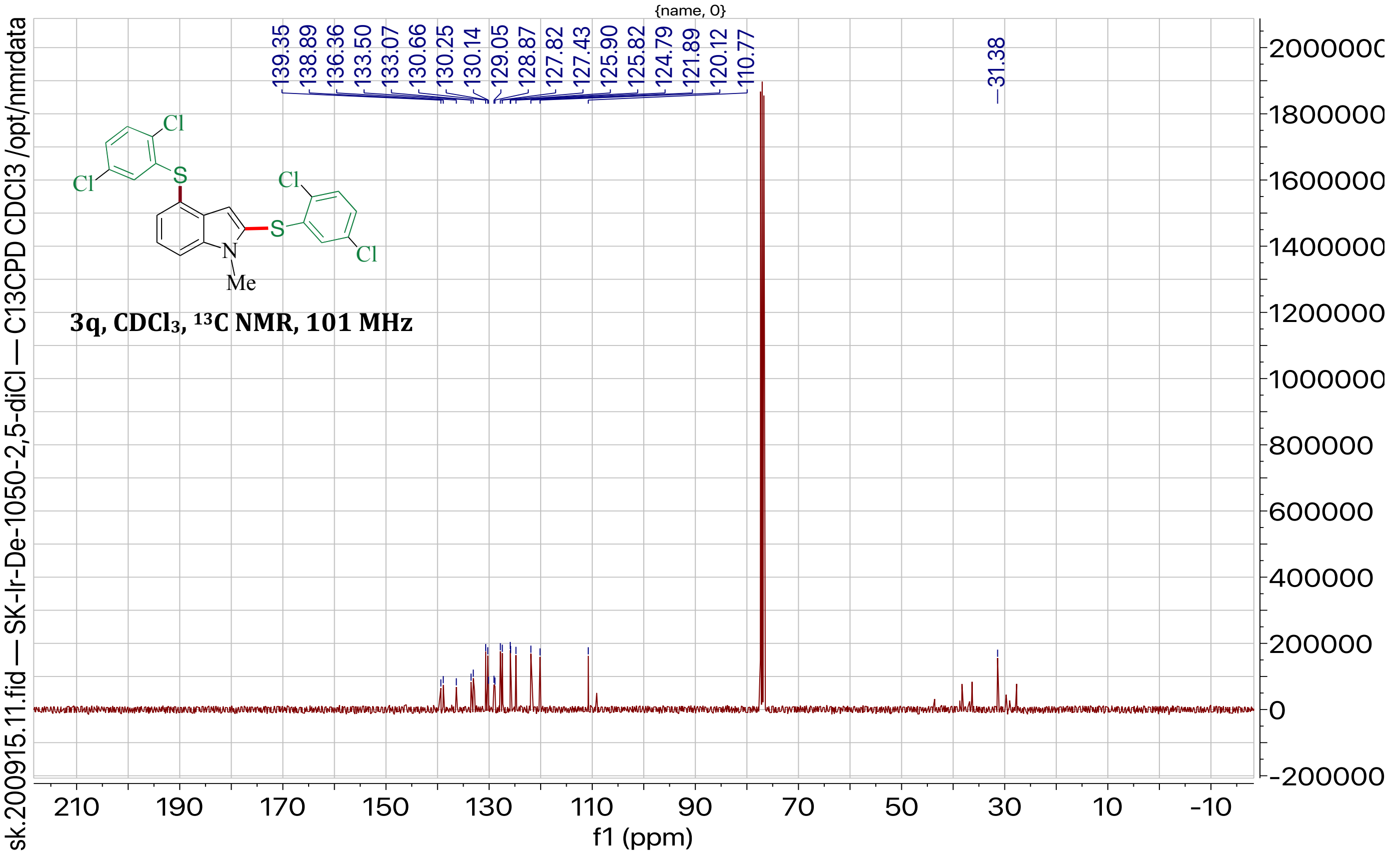

HRMS spectrum of 3q

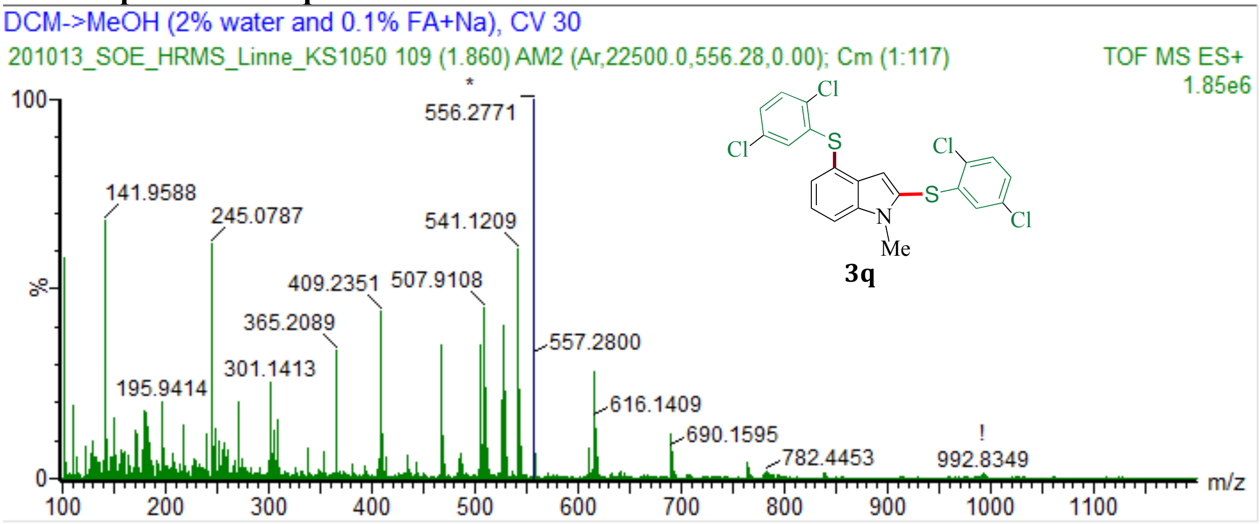

Single Mass Analysis
Tolerance = 2.0 mDa / DBE: min = -0.5, max = 100.0
Element prediction: Off
Number of isotope peaks used for i-FIT = 3
Monoisotopic Mass, Even Electron Ions
148 formula(e) evaluated with 2 results within limits (all results (up to 1000) for each mass)
Elements Used:
C: 0-50 H: 0-50 N: 0-2 O: 0-1 Na: 0-1 S: 0-2
Cl: 4-4

Mass	Calc. Mass	mDa	PPM	DBE	Formula	Fit Conf %	C	H	N	O	Na	S	Cl
505.9128	505.9132	-0.4	-0.8	21.5	C26 H8 N S Cl4	40..70.95	26	8	1			1	4
	505.9141	-1.3	-2.6	13.5	C21 H13 N Na S2 Cl4	41..29.05	21	13	1		1	2	4

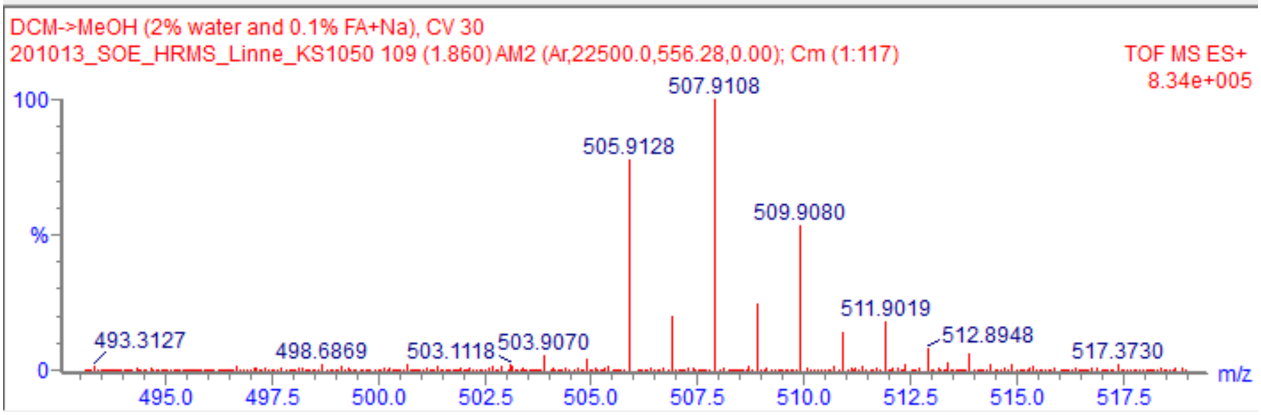

^{1}H NMR spectrum of 3r

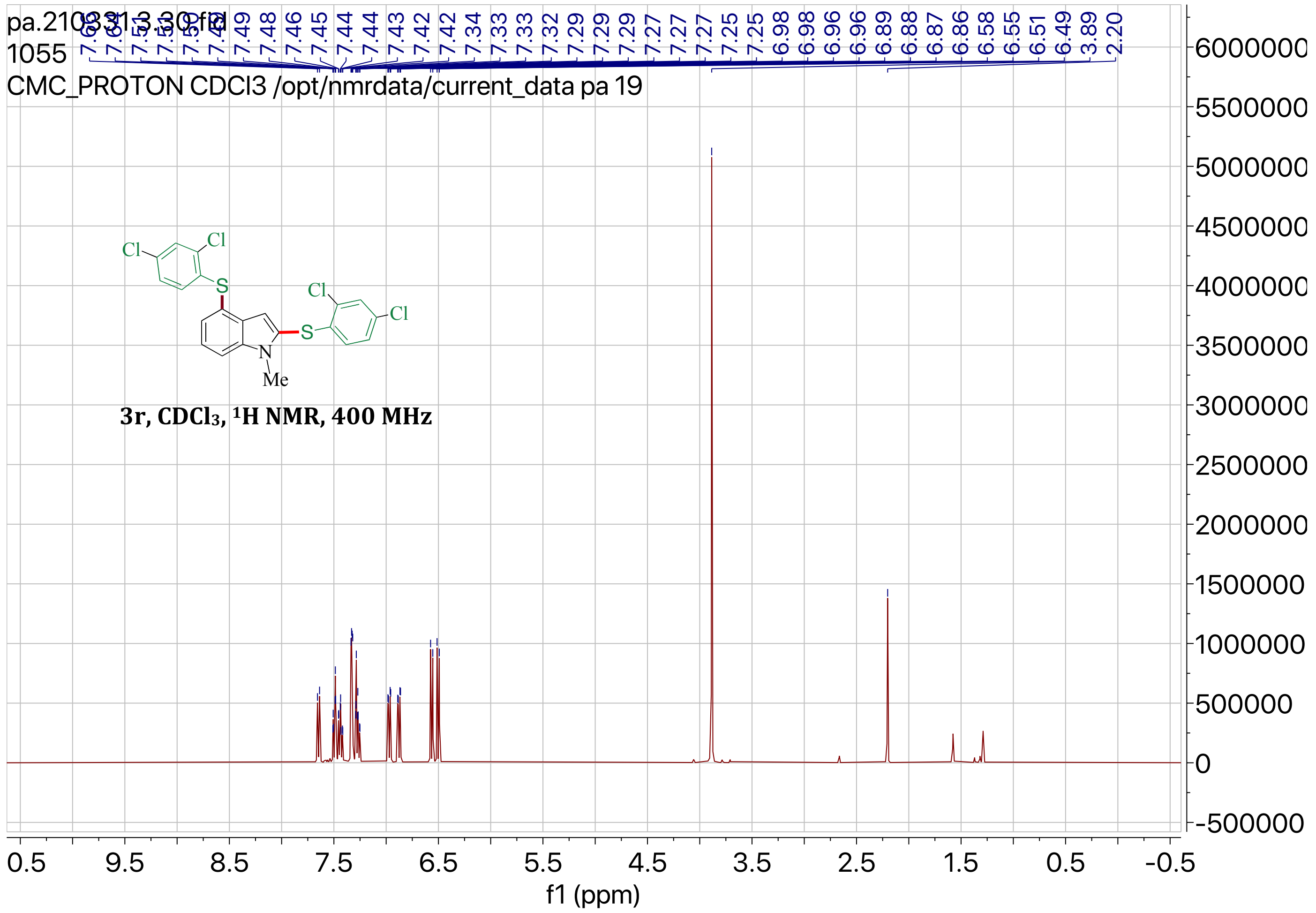

^{13}C{^{1}H} NMR spectrum of 3r

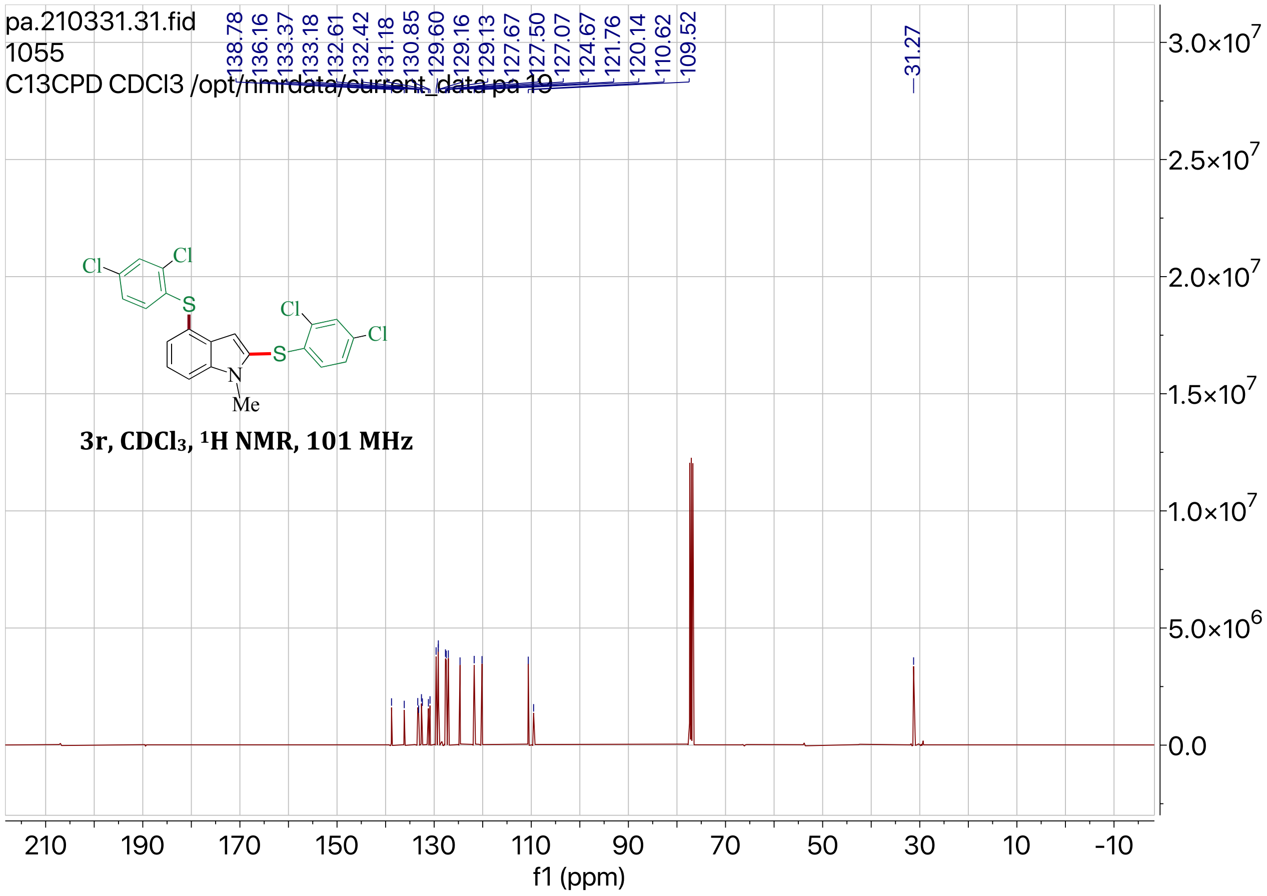

HRMS spectrum of 3r

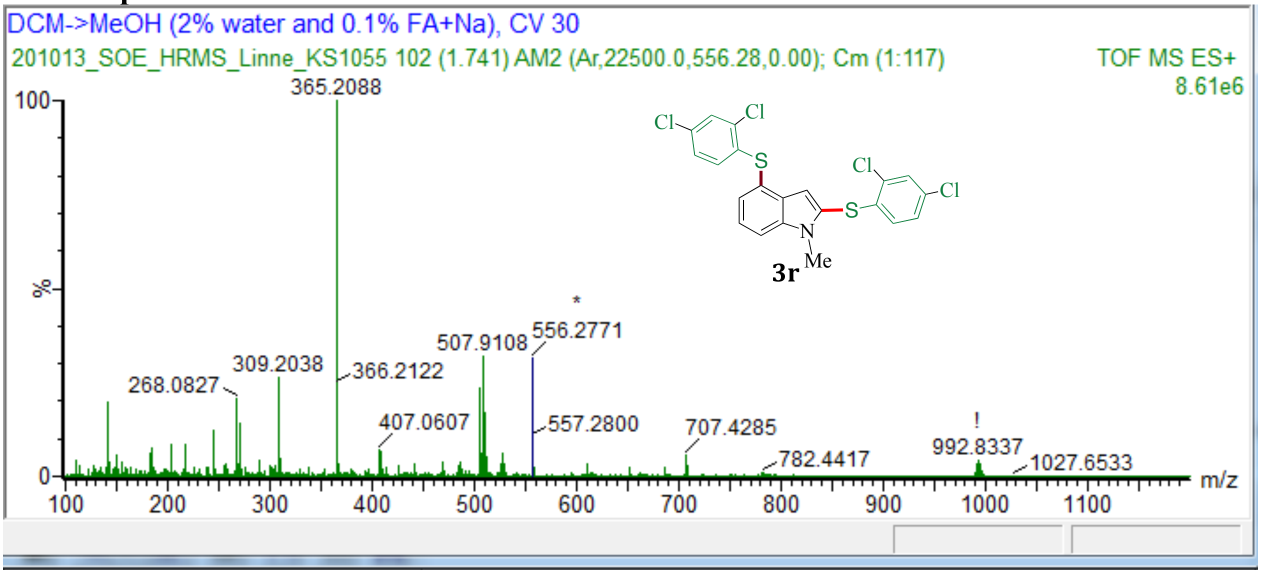

Single Mass Analysis
Tolerance = 2.0 mDa / DBE: min = -0.5, max = 100.0
Element prediction: Off
Number of isotope peaks used for i-FIT = 3
Monoisotopic Mass, Even Electron Ions
148 formula(e) evaluated with 2 results within limits (all results (up to 1000) for each mass)
Elements Used:
C: 0-50 H: 0-50 N: 0-2 O: 0-1 Na: 0-1 S: 0-2
Cl: 4-4

Mass	Calc. Mass	mDa	PPM	DBE	Formula	i...	Fit Conf %	C	H	N	O	Na	S	Cl
505.9135	505.9132	0.3	0.6	21.5	C26 H8 N S Cl4	5...	1.69	26	8	1			1	4
	505.9141	-0.6	-1.2	13.5	C21 H13 N Na S2 Cl4	0...	98.31	21	13	1		1	2	4

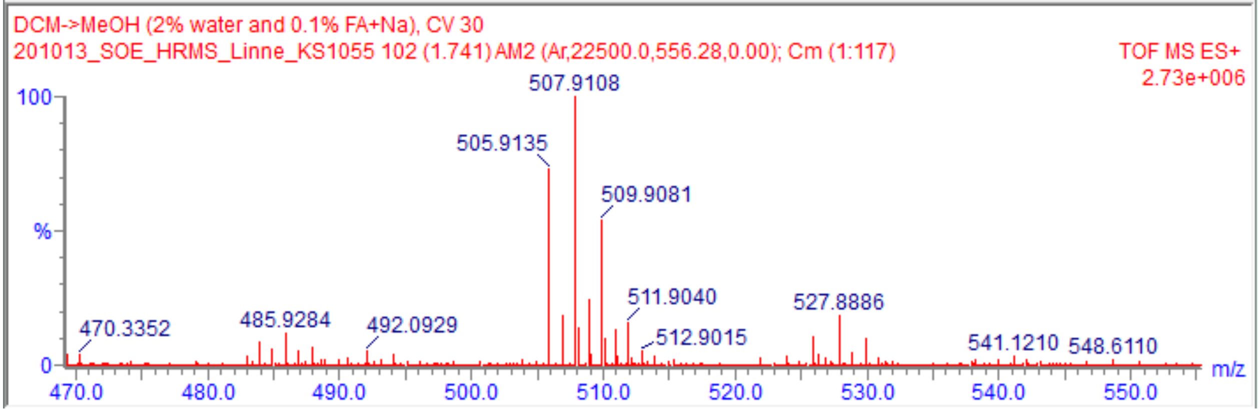

^{1}H NMR spectrum of 3s

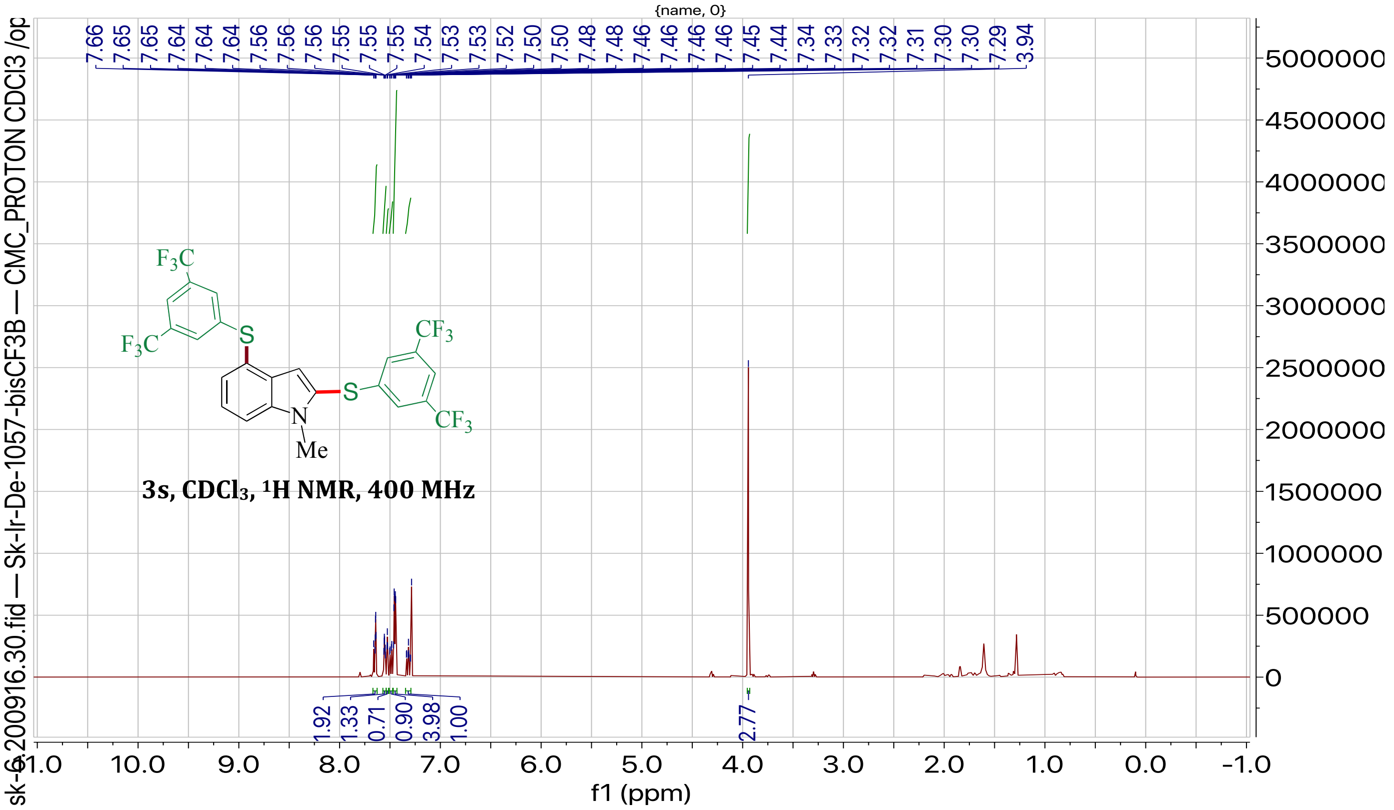

^{13}C{^{1}H} NMR spectrum of 3s

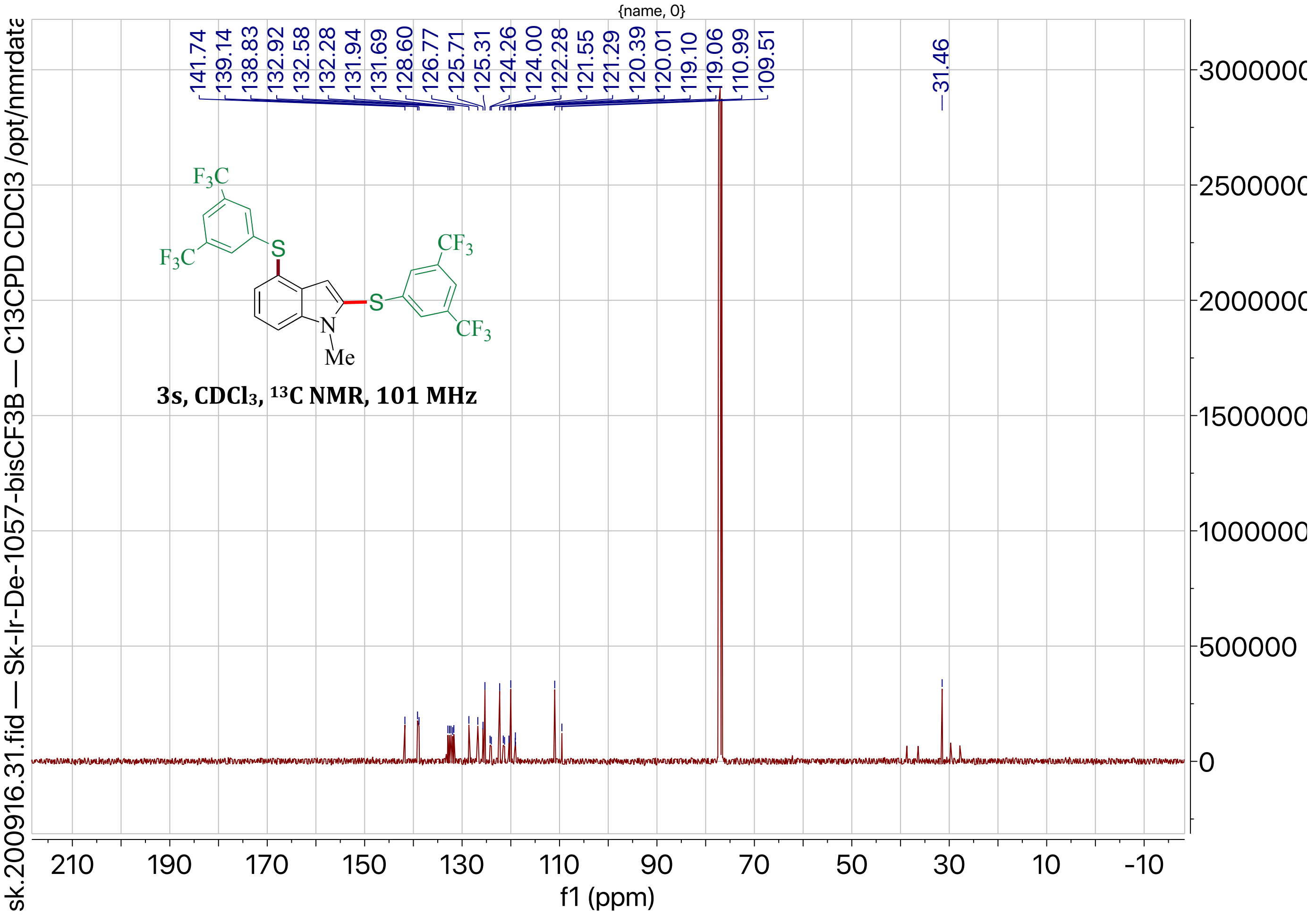

^{19}F NMR spectrum of 3s

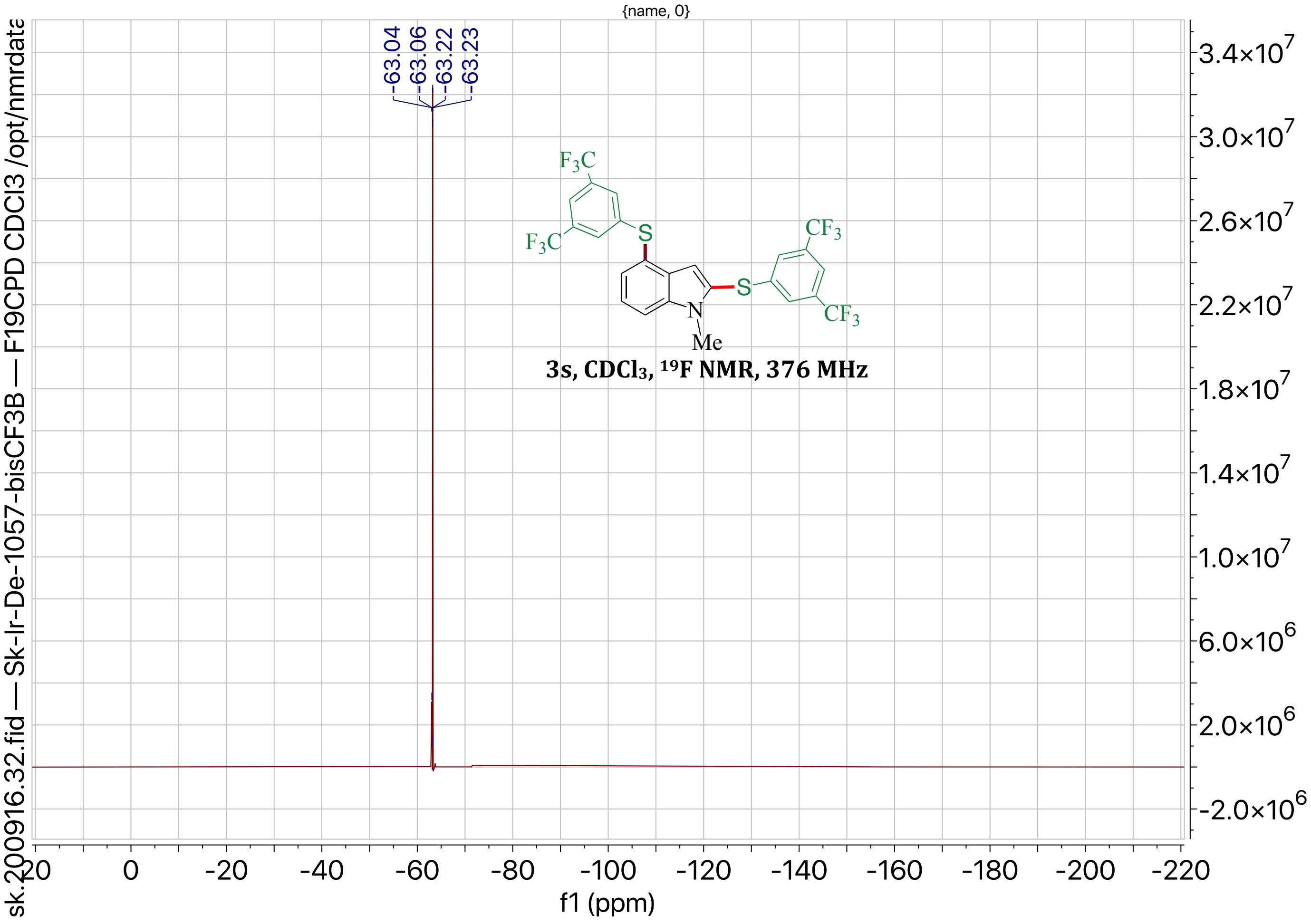

HRMS spectrum of 3s

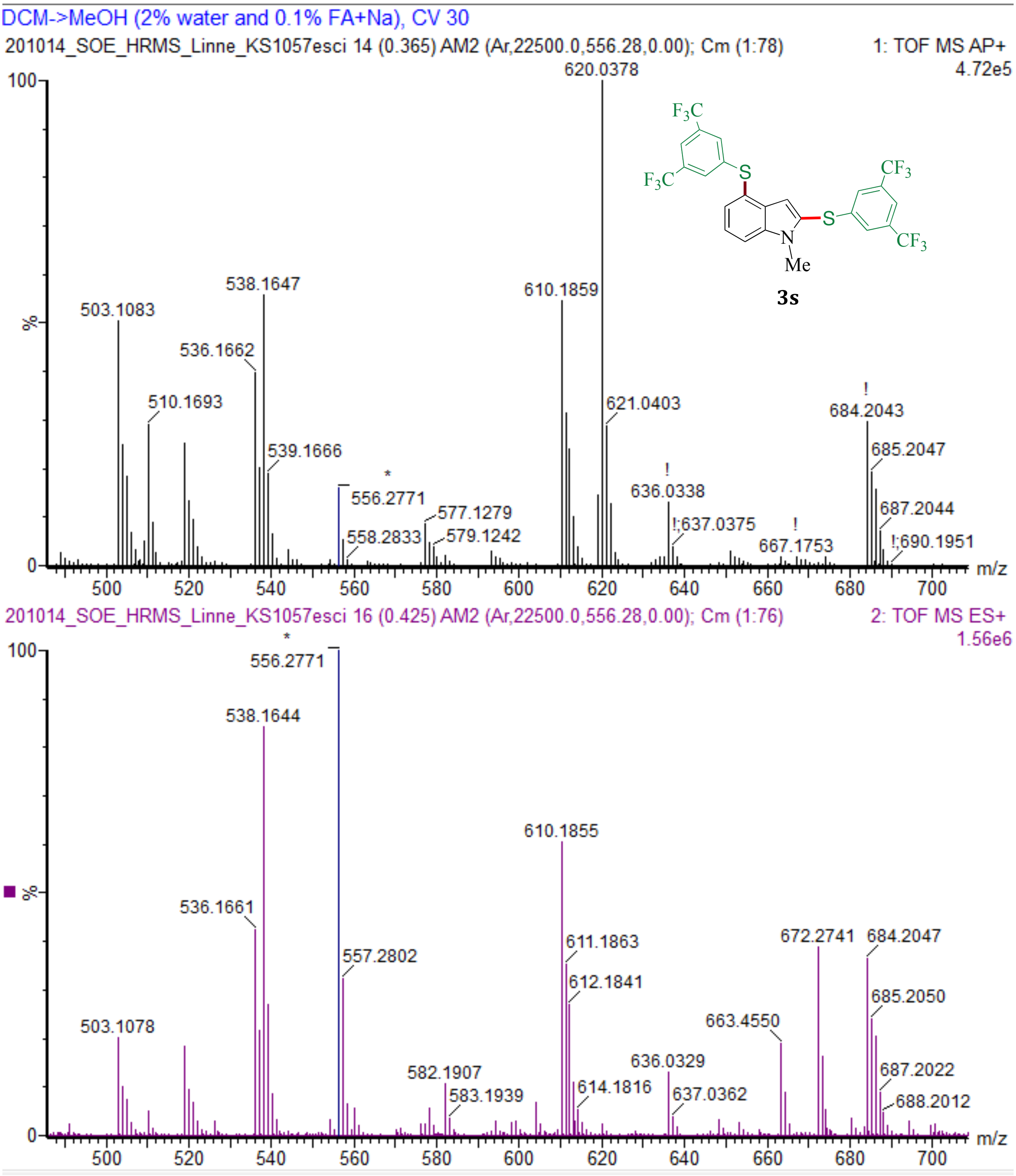

^1H NMR spectrum of 4b

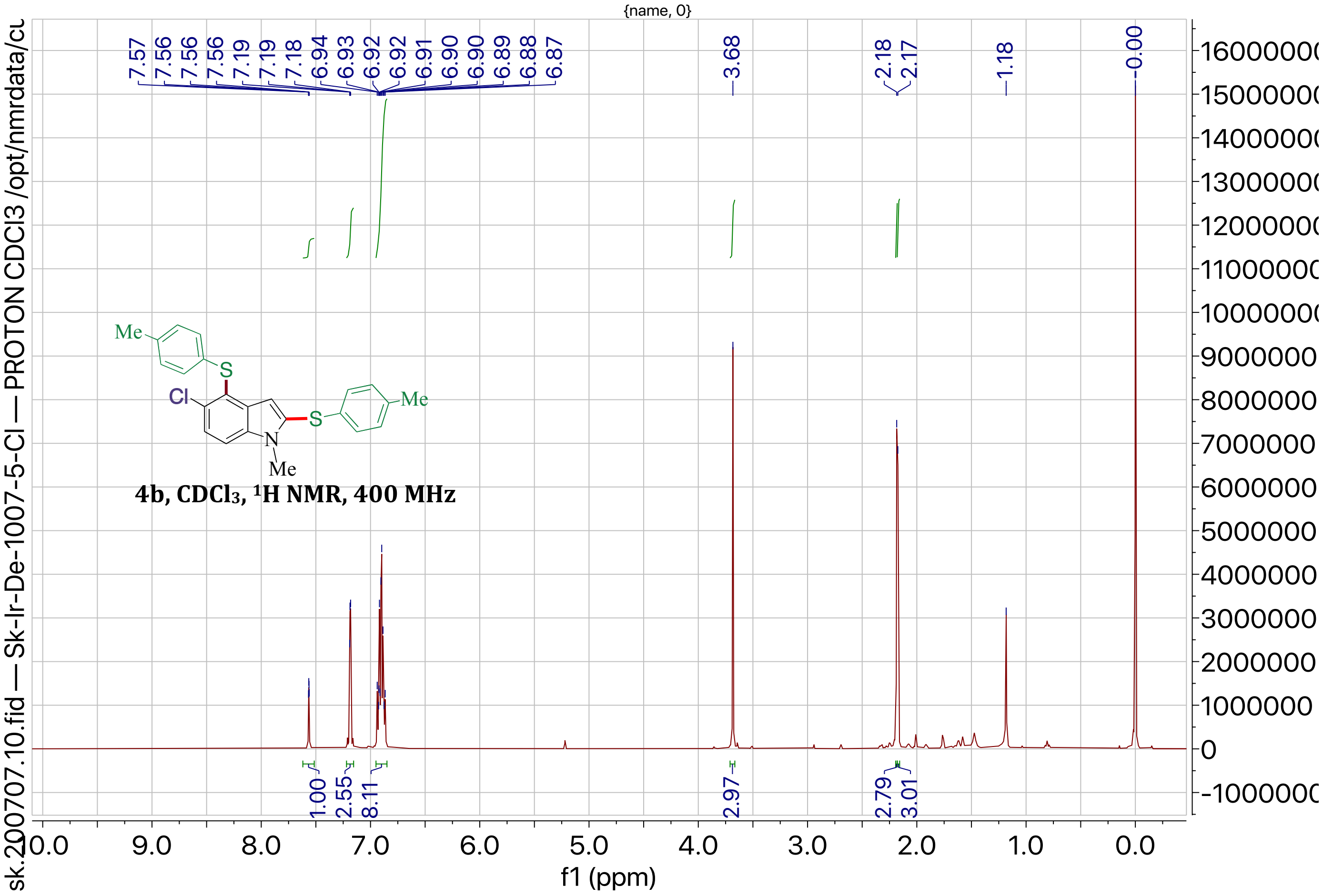

$^{13}C\{^1H\}$ NMR spectrum of 4b

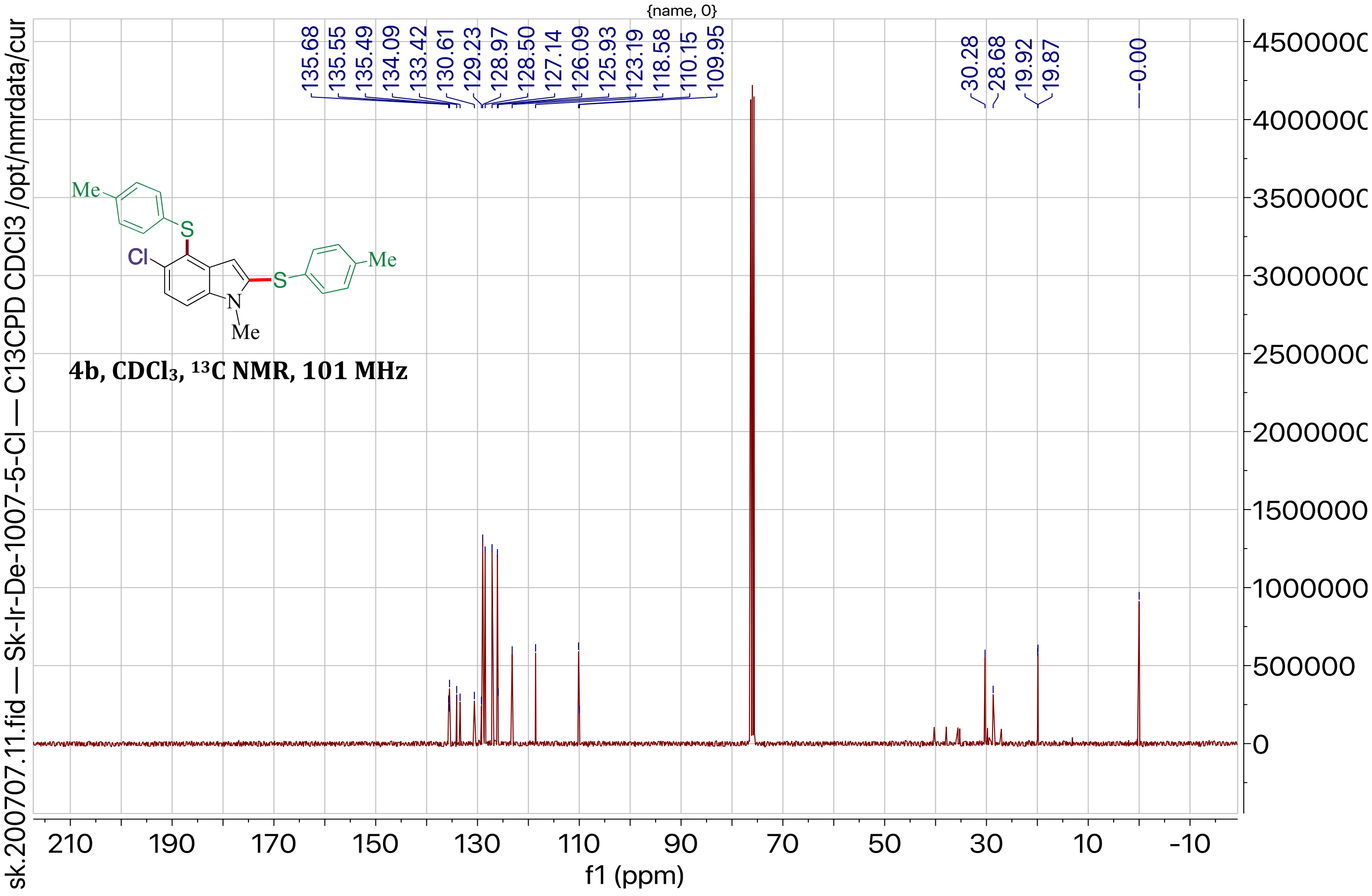

HRMS spectrum of 4b

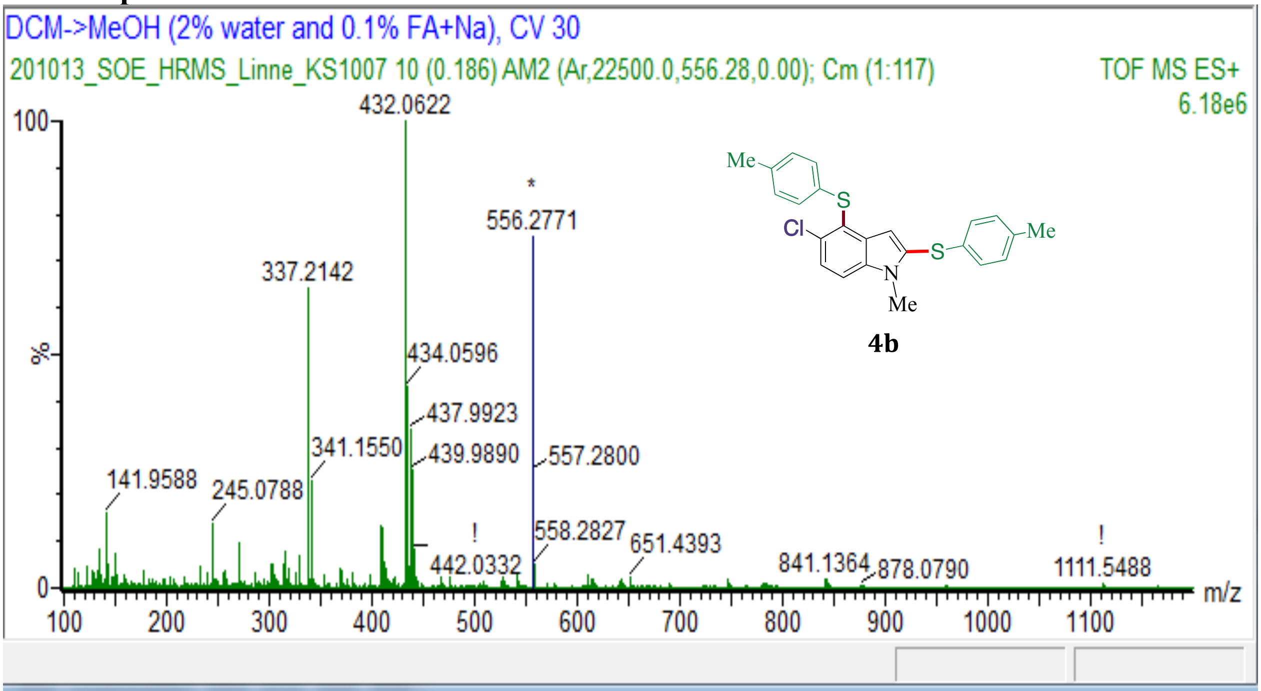

Single Mass Analysis

Tolerance = 2.0 mDa / DBE: min = -0.5, max = 100.0

Element prediction: Off

Number of isotope peaks used for i-FIT = 3

Monoisotopic Mass, Even Electron Ions

596 formula(e) evaluated with 3 results within limits (all results (up to 1000) for each mass)

Elements Used:

C: 0-50 H: 0-50 N: 0-2 O: 0-3 Na: 0-1 S: 0-2

Cl: 1-2

Mass	Calc. Mass	mDa	PPM	DBE	Formula	i-FIT	Fit Conf %	C	H	N	O	Na	S	Cl
432.0622	432.0623	-0.1	-0.2	13.5	C23 H20 N Na S2 Cl	60...	99.97	23	20	1		1	2	1
	432.0626	-0.4	-0.9	7.5	C19 H24 N O2 S2 Cl2	61...	0.00	19	24	1	2		2	2
	432.0614	0.8	1.9	21.5	C28 H15 N S Cl	68...	0.03	28	15	1			1	1

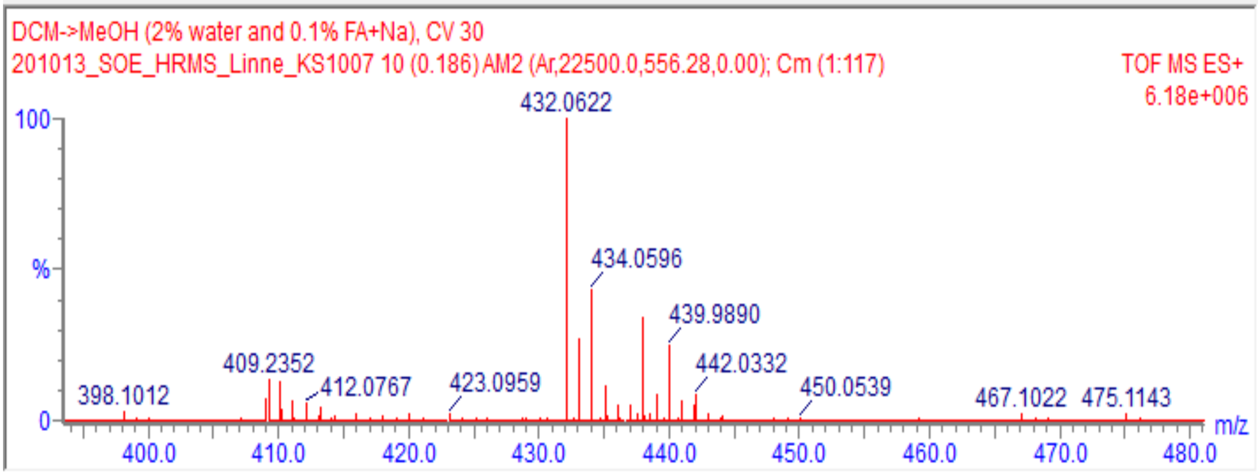

^{1}H NMR spectrum of 4c

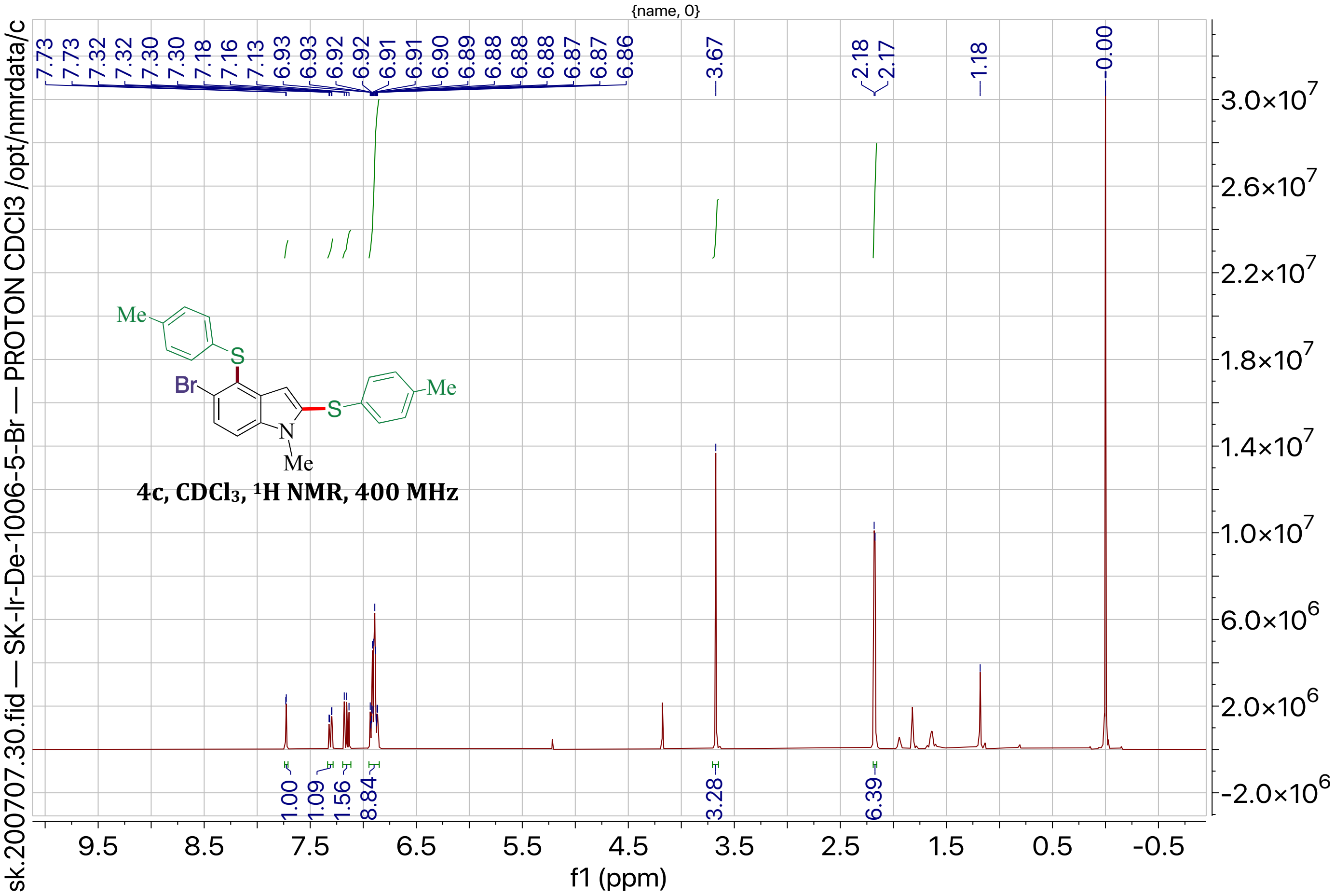

^{13}C{^{1}H} NMR spectrum of 4c

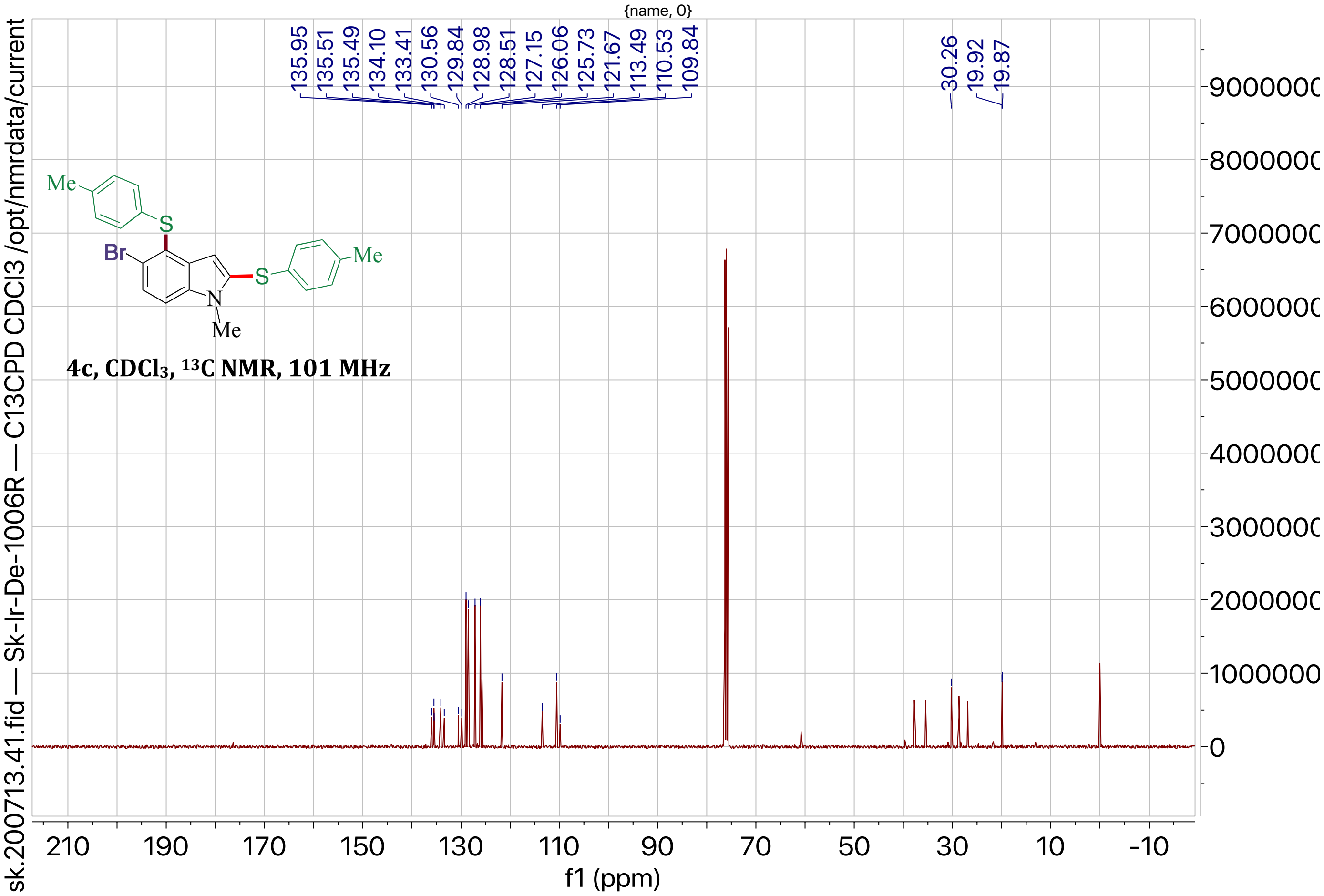

HRMS spectrum of 4c

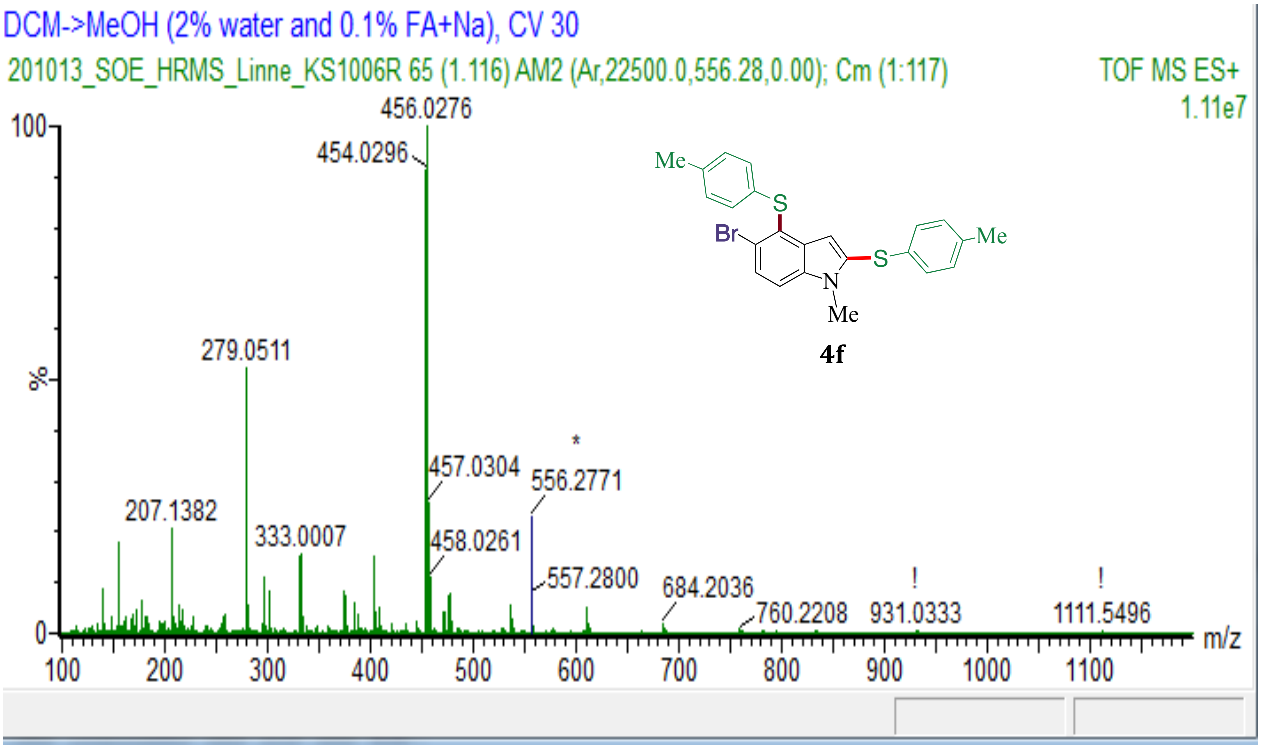

Single Mass Analysis

Tolerance = 2.0 mDa / DBE: min = -0.5, max = 100.0

Element prediction: Off

Number of isotope peaks used for i-FIT = 3

Monoisotopic Mass, Even Electron Ions

613 formula(e) evaluated with 3 results within limits (all results (up to 1000) for each mass)

Elements Used:

C: 0-50 H: 0-50 N: 0-2 O: 0-3 Na: 0-1 S: 0-2

Br: 0-1

Mass	Calc. Mass	mDa	PPM	DBE	Formula	i...	Fit Conf %	C	H	N	O	Na	S	Br
454.0296	454.0299	-0.3	-0.7	13.5	C23 H21 N S2 Br	60...	100.00	23	21	1			2	1
	454.0293	0.3	0.7	34.5	C35 H4 N O	62...	0.00	35	4	1	1			

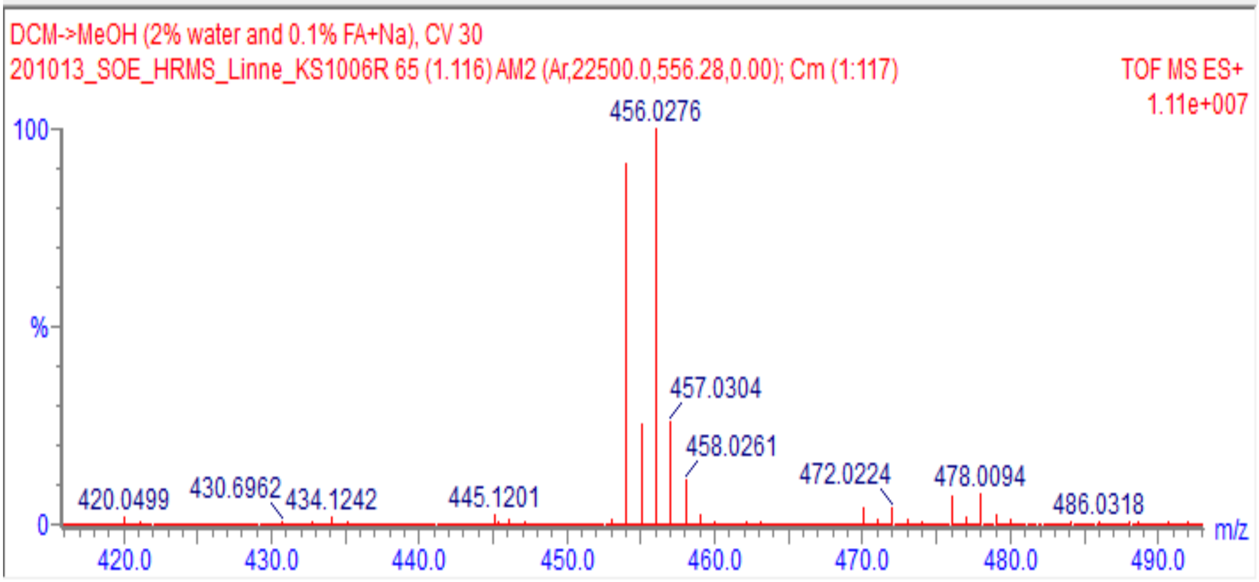

^{1}H NMR spectrum of 4d

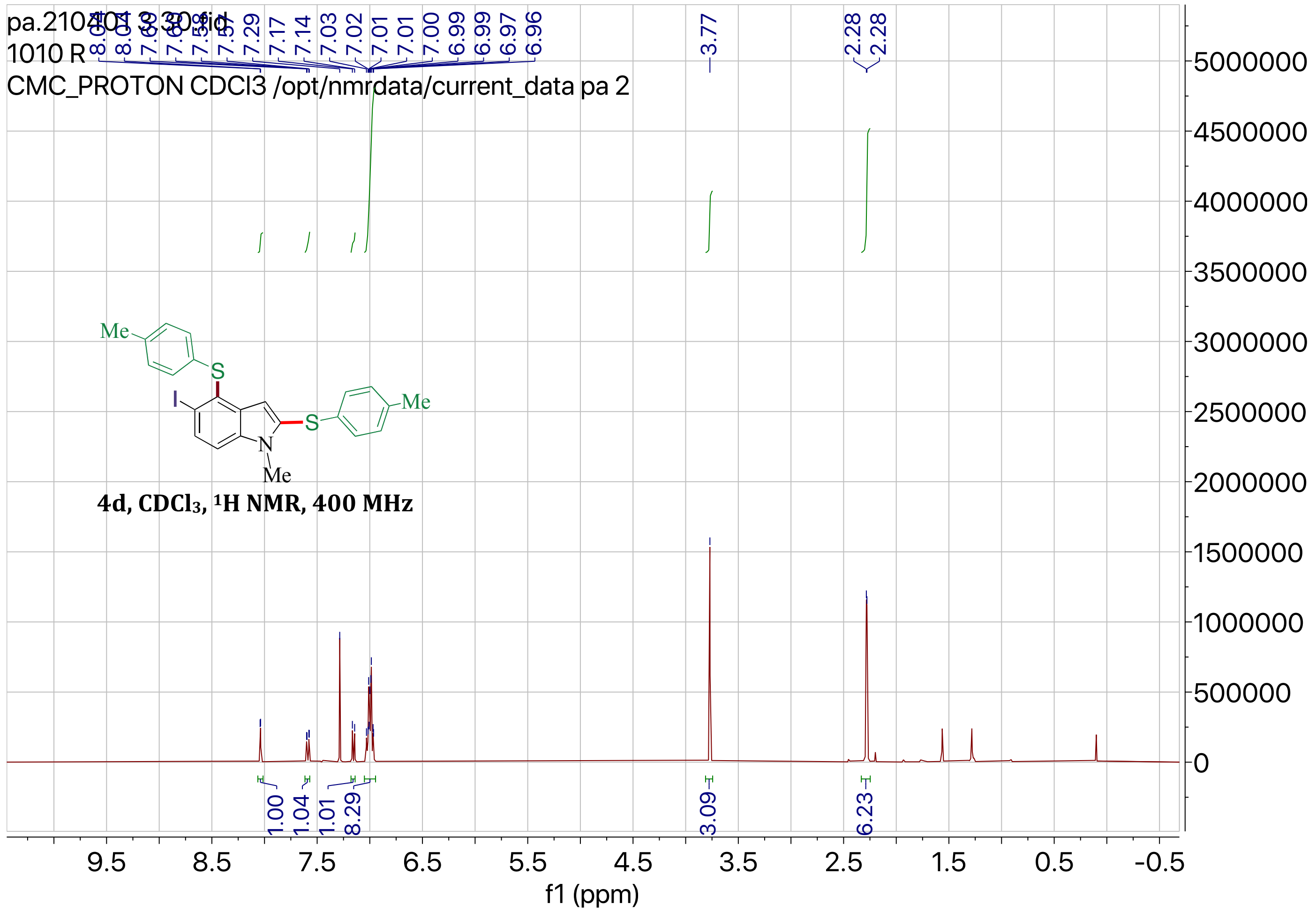

^{13}C{^{1}H} NMR spectrum of 4d

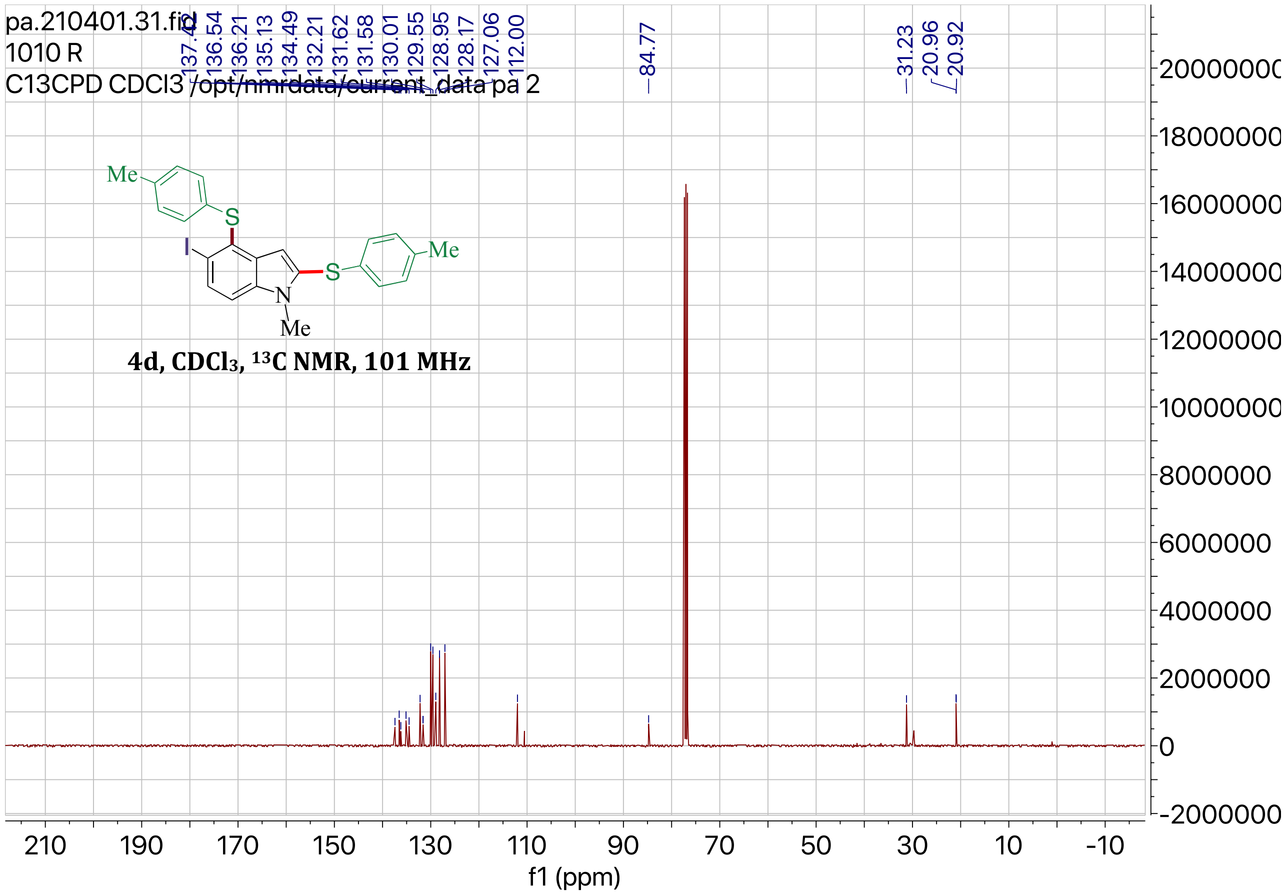

HRMS spectrum of 4d

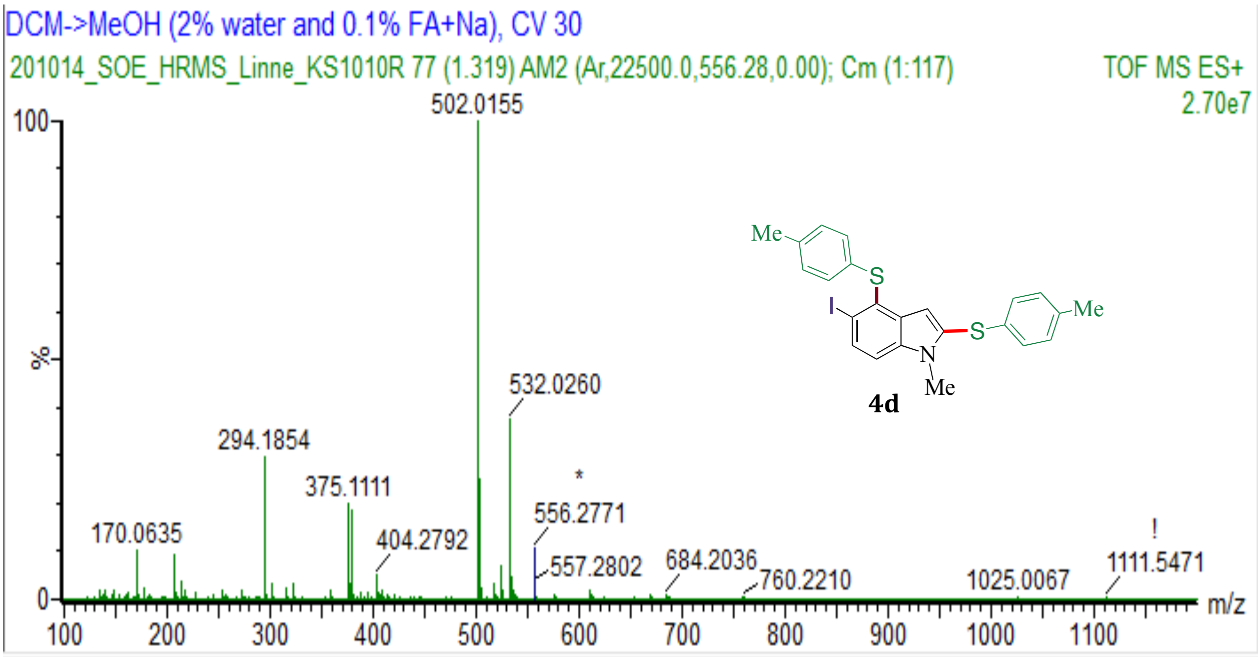

Single Mass Analysis

Tolerance = 3.0 mDa / DBE: min = -0.5, max = 100.0

Element prediction: Off

Number of isotope peaks used for i-FIT = 3

Monoisotopic Mass, Even Electron Ions

461 formula(e) evaluated with 3 results within limits (all results (up to 1000) for each mass)

Elements Used:

C: 0-50 H: 0-50 N: 0-2 O: 0-1 Na: 0-1 S: 0-2

I: 0-2

Mass	Calc. Mass	mDa	PPM	DBE	Formula	i..	Fit Conf %	C	H	N	O	Na	S	I
502.0155	502.0160	-0.5	-1.0	13.5	C23 H21 N S2 I	5	...31.62	23	21	1			2	1
	502.0136	1.9	3.8	10.5	C21 H22 N Na S2 I	5	...68.37	21	22	1		1	2	1
	502.0126	2.9	5.8	18.5	C26 H17 N S I	5	...0.01	26	17	1			1	1

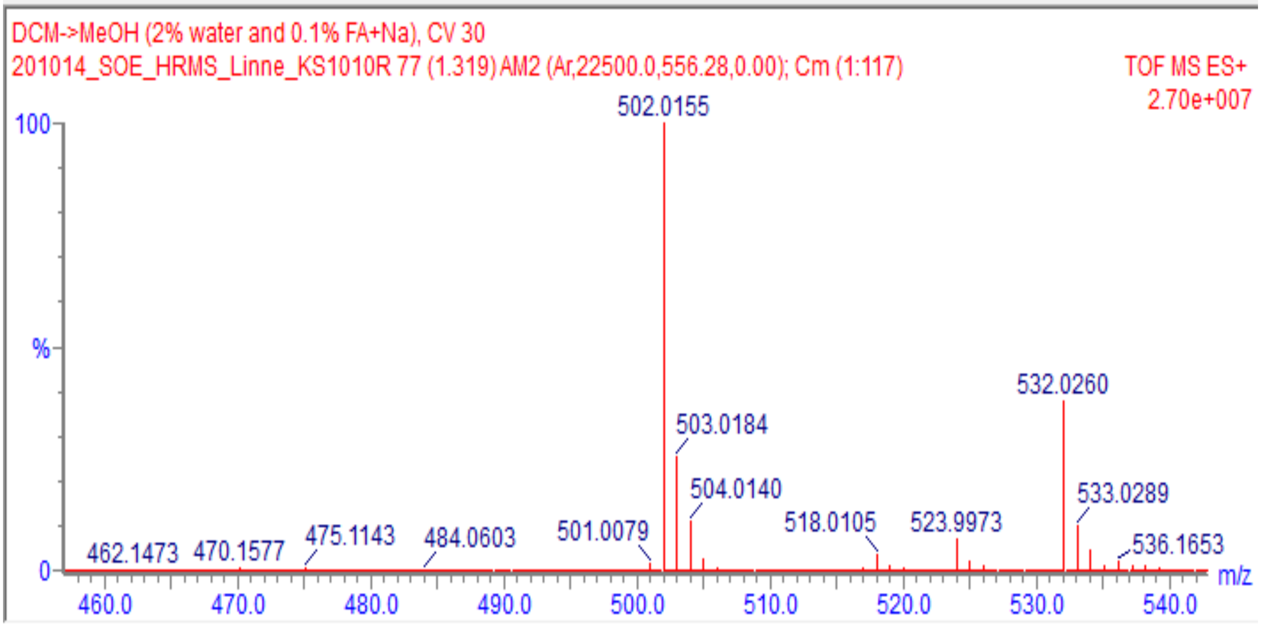

^{1}H NMR spectrum of 4e

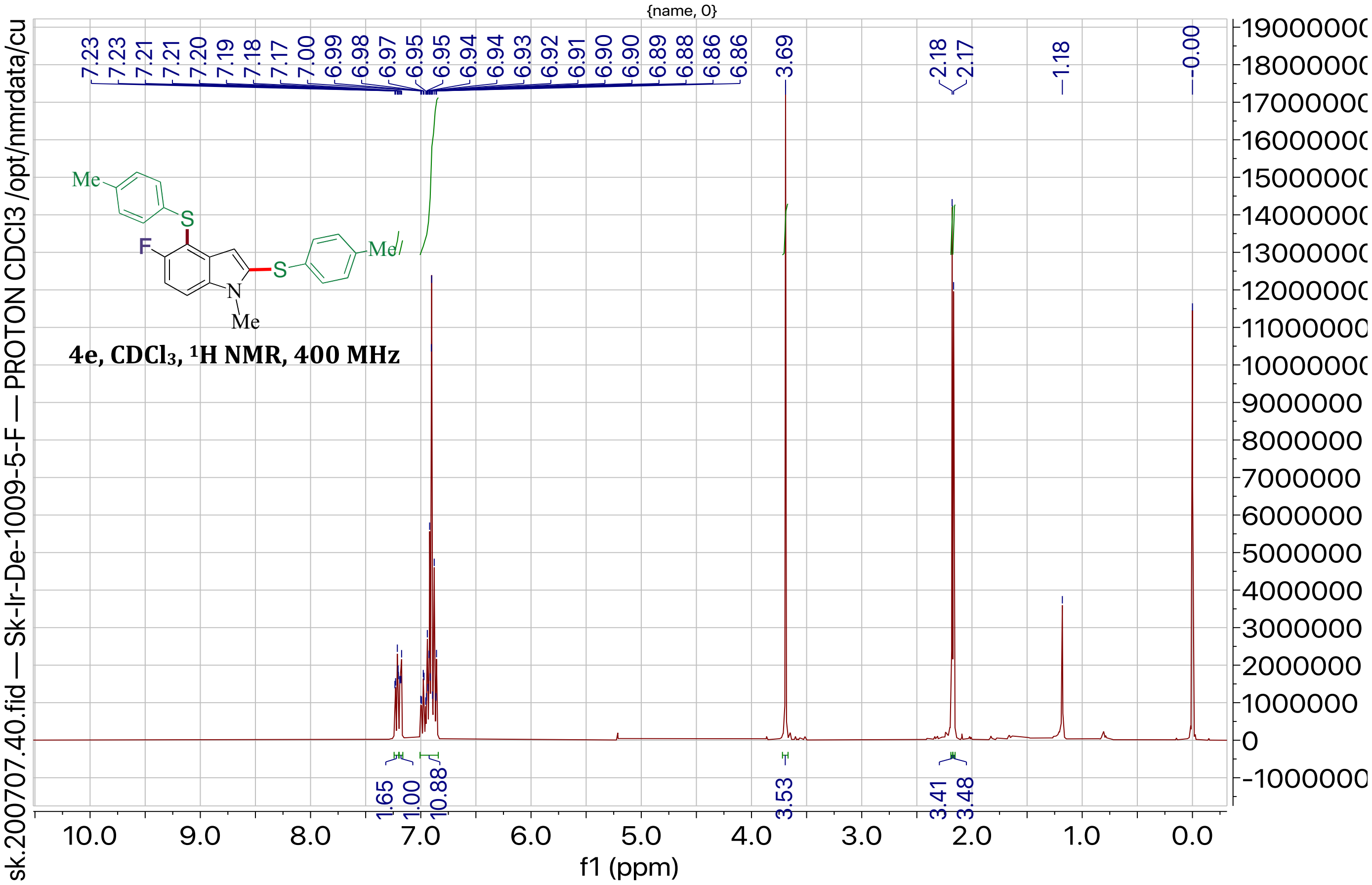

^{19}F NMR spectrum of 3s

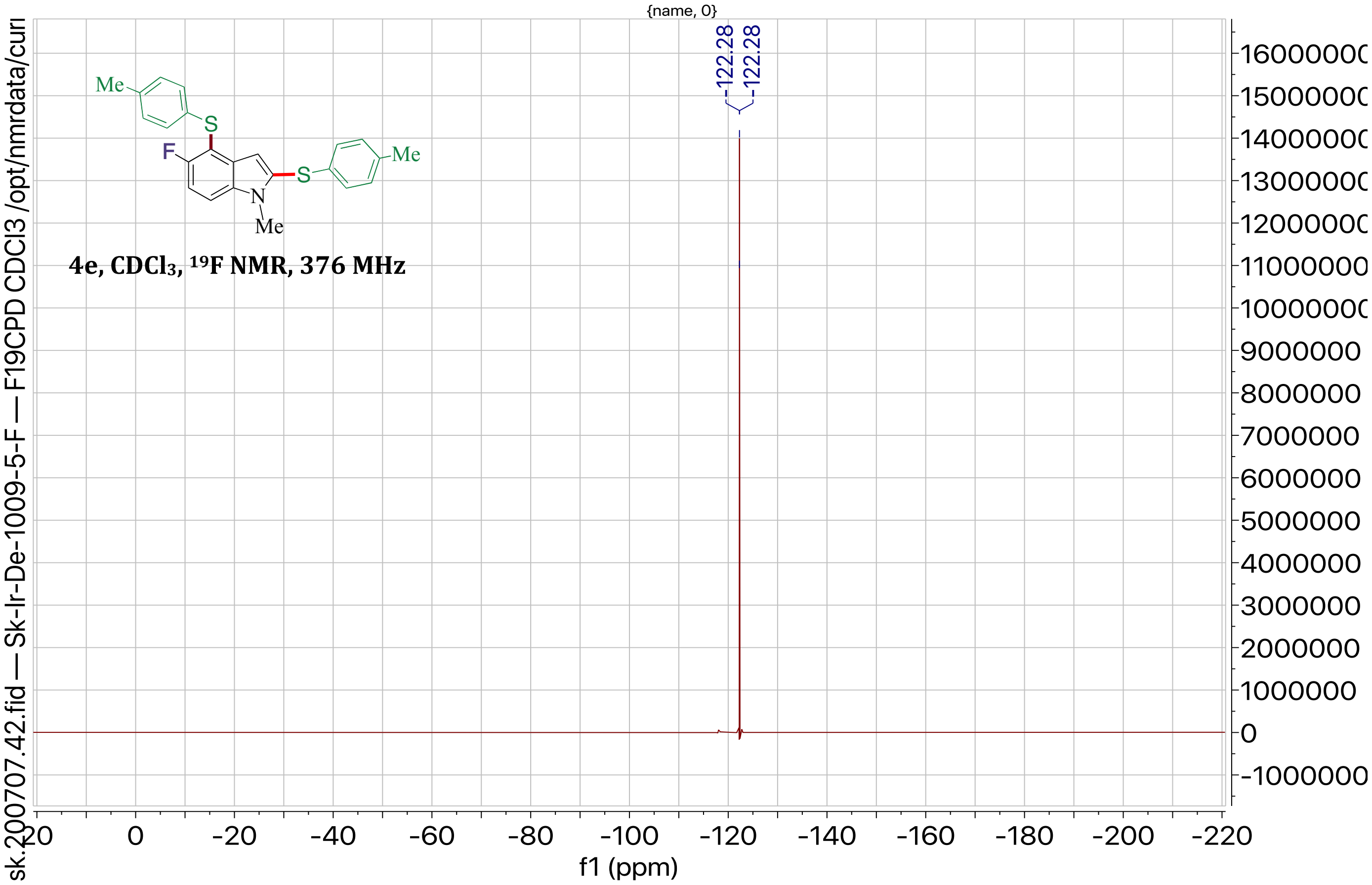

$^{13}C\{^{1}H\}$ NMR spectrum of 4e

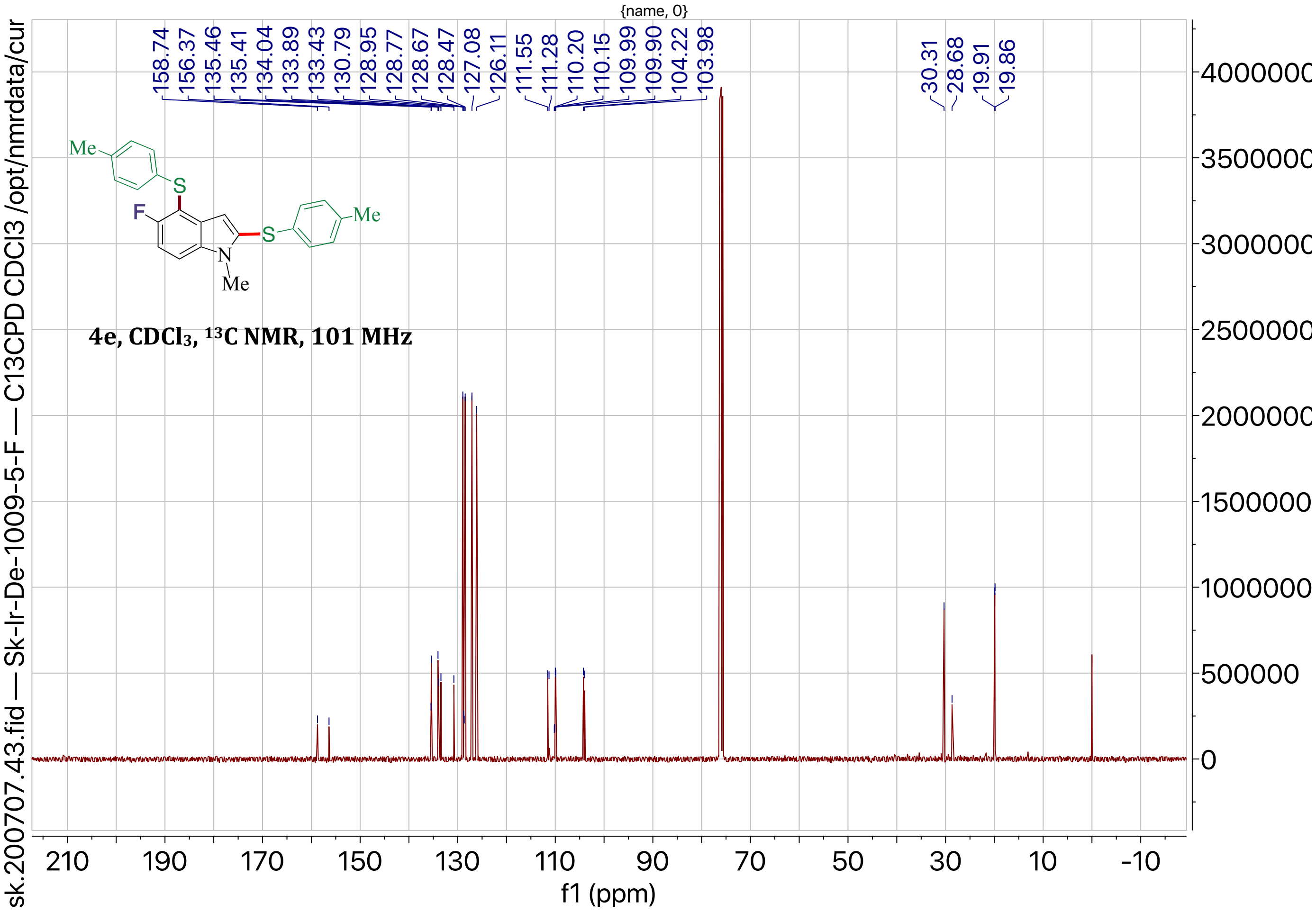

HRMS spectrum of 4e

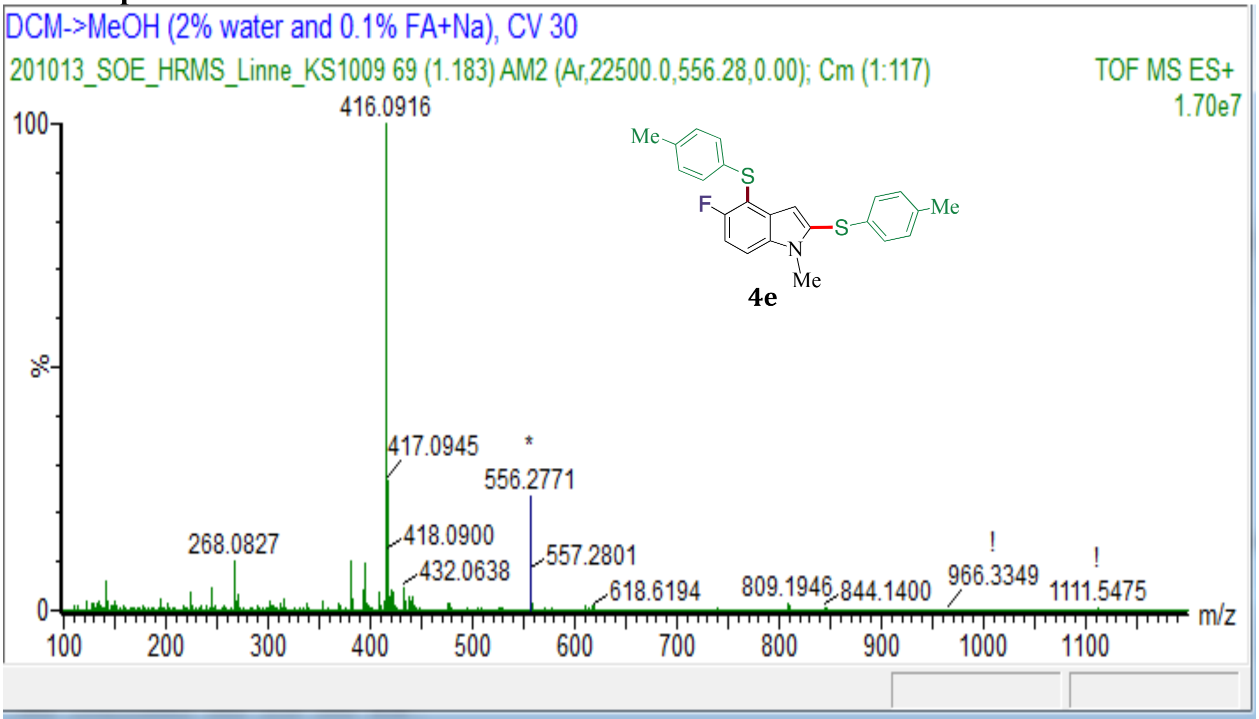

Single Mass Analysis

Tolerance = 2.0 mDa / DBE: min = -0.5, max = 100.0

Element prediction: Off

Number of isotope peaks used for i-FIT = 3

Monoisotopic Mass, Even Electron Ions

616 formula(e) evaluated with 2 results within limits (all results (up to 1000) for each mass)

Elements Used:

C: 0-50 H: 0-50 N: 0-2 O: 0-3 F: 0-1 Na: 0-1

S: 0-2

Mass	Calc. Mass	mDa	PPM	DBE	Formula	i..	Fit Conf %	C	H	N	O	F	Na	S
416.0916	416.0919	-0.3	-0.7	13.5	C23 H20 N F Na S2	6.0...	100.00	23	20	1		1	1	2
	416.0909	0.7	1.7	21.5	C28 H15 N F S	6.1...	0.00	28	15	1		1		1

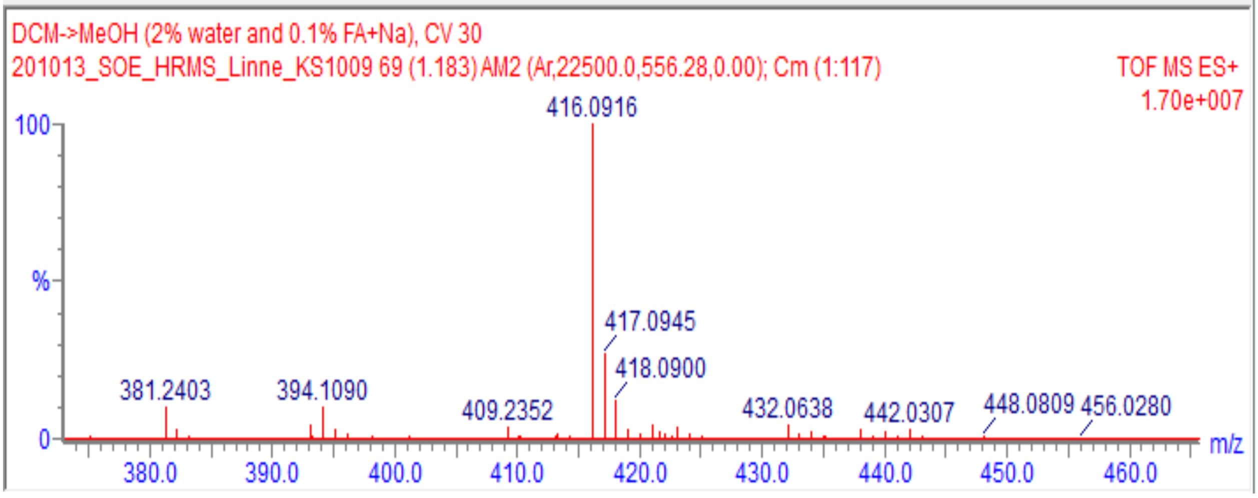

^{1}H NMR spectrum of 4f

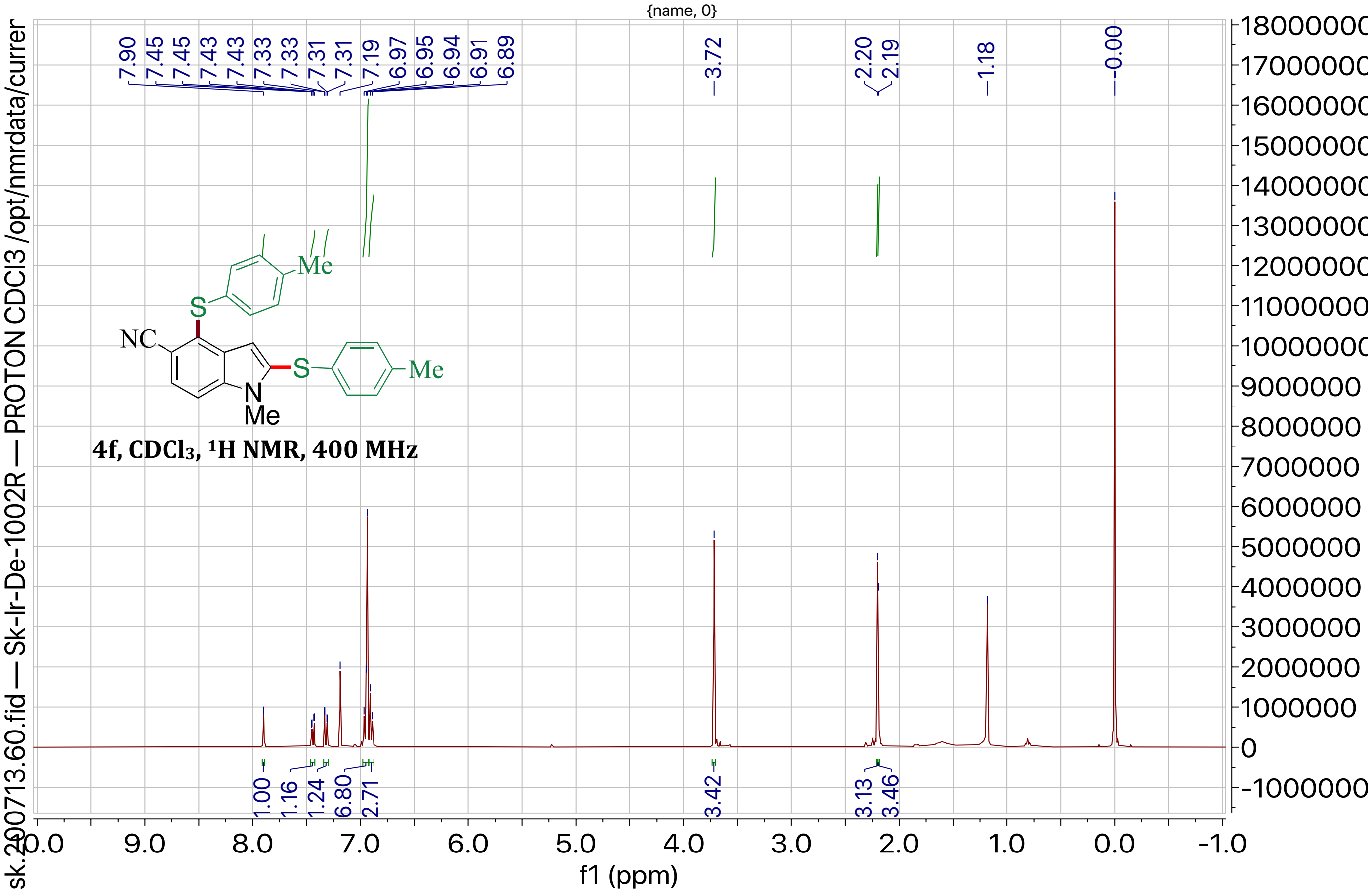

^{13}C{^{1}H} NMR spectrum of 4f

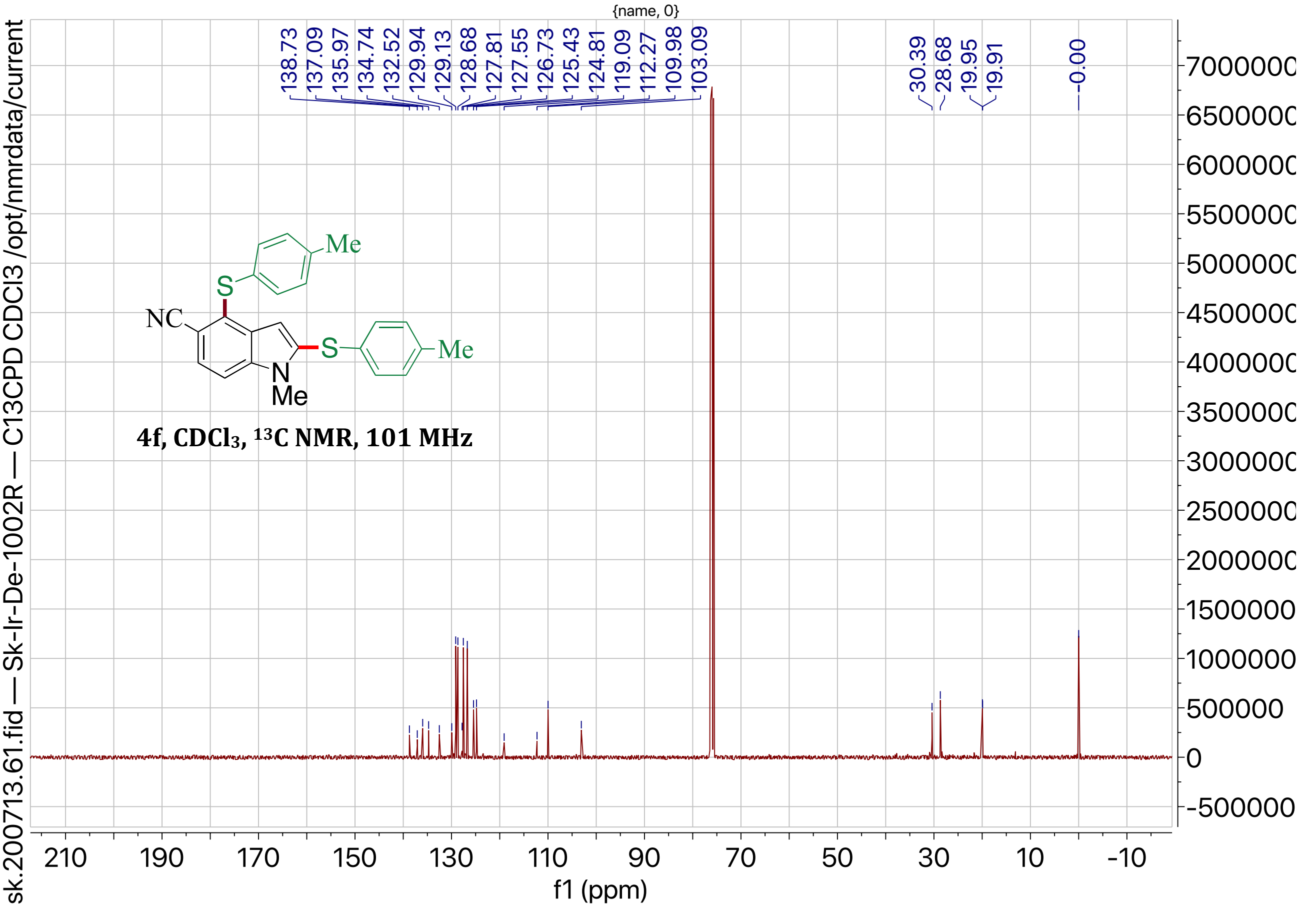

HRMS spectrum of 4f

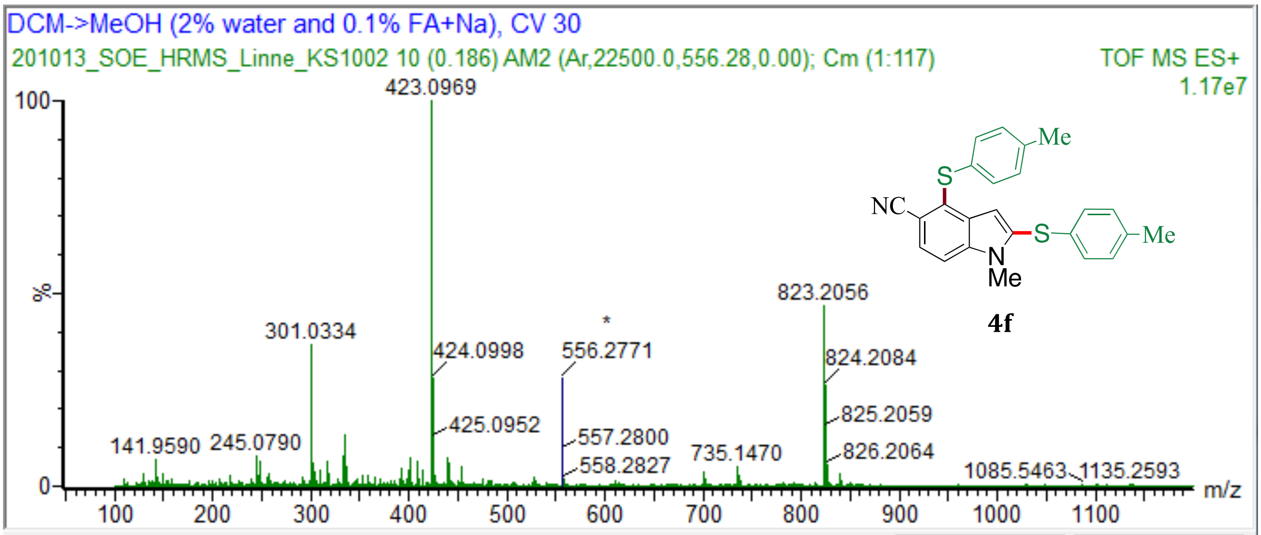

Single Mass Analysis
Tolerance = 2.0 mDa / DBE: min = -0.5, max = 100.0
Element prediction: Off
Number of isotope peaks used for i-FIT = 3
Monoisotopic Mass, Even Electron Ions
312 formula(e) evaluated with 2 results within limits (all results (up to 1000) for each mass)
Elements Used:
C: 0-50 H: 0-50 N: 0-2 O: 0-3 Na: 0-1 S: 0-2

Mass	Calc. Mass	mDa	PPM	DBE	Formula	i...	Fit Conf %	C	H	N	O	Na	S
423.0969	423.0966	0.3	0.7	15.5	C24 H20 N2 Na S2	60...	100.00	24	20	2		1	2
	423.0956	1.3	3.1	23.5	C29 H15 N2 S	61...	0.00	29	15	2			1

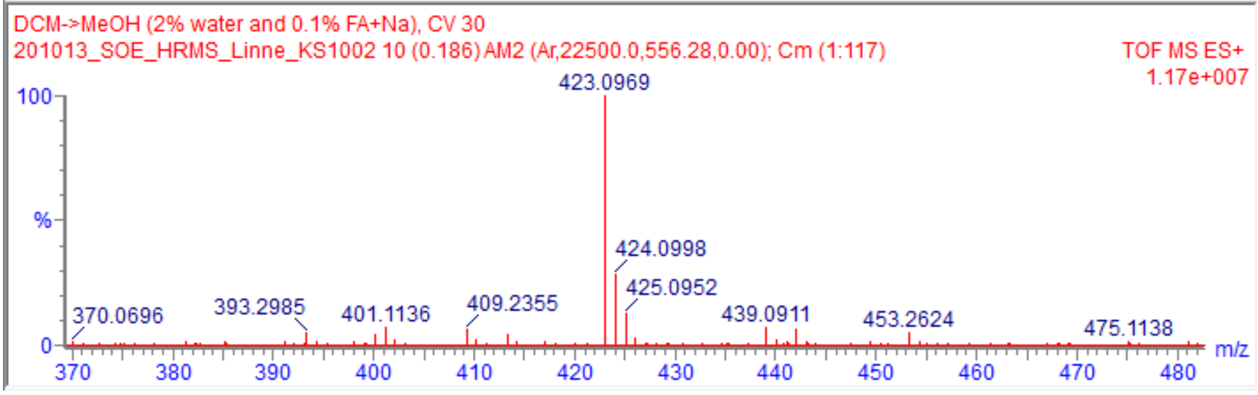

^{1}H NMR spectrum of 4g

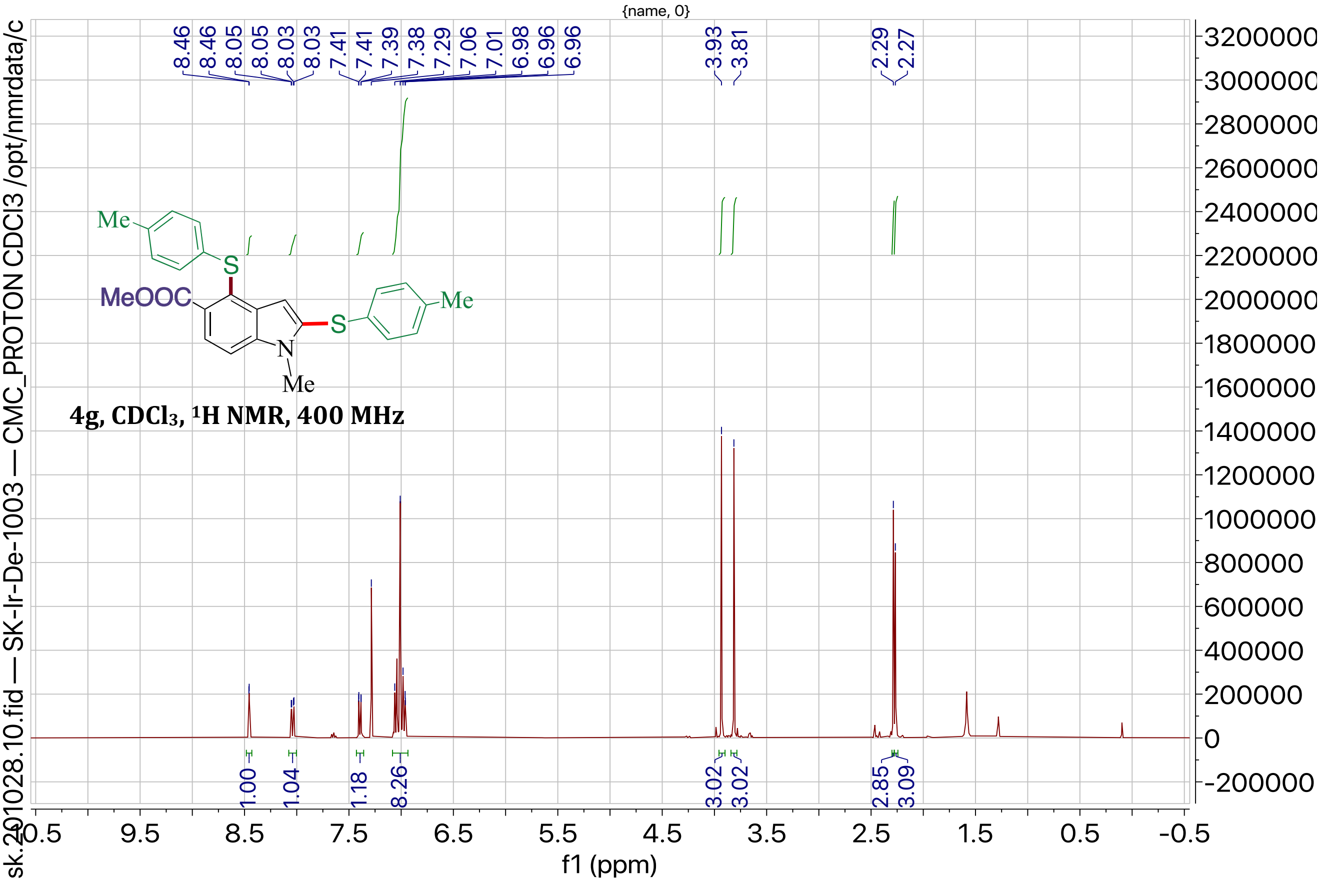

^{3}C{^{1}H} NMR spectrum of 4g

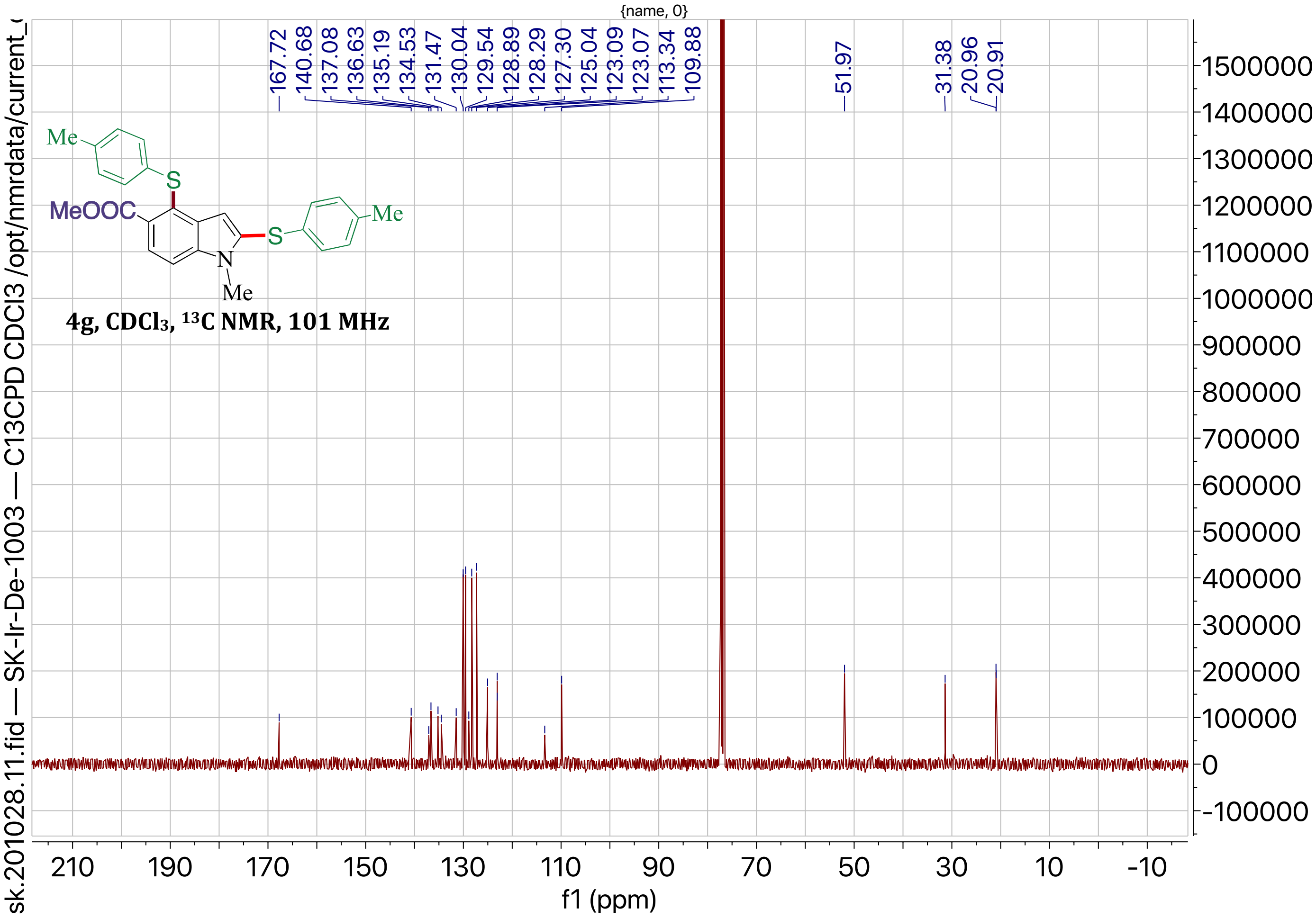

HRMS spectrum of 4g

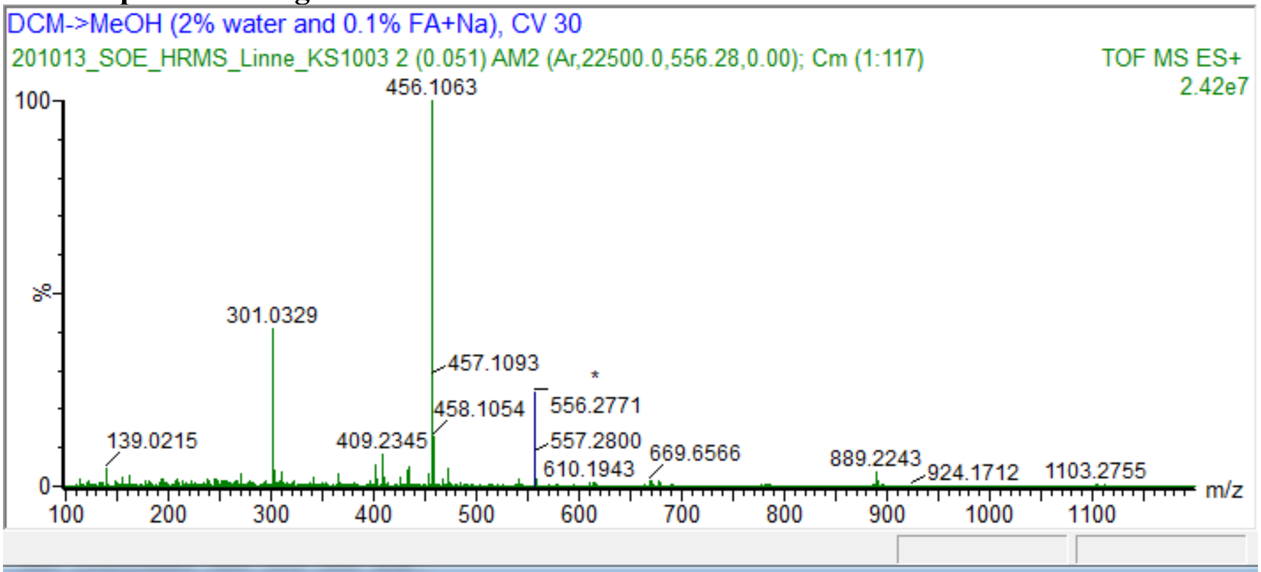

Single Mass Analysis

Tolerance = 2.0 mDa / DBE: min = -0.5, max = 100.0

Element prediction: Off

Number of isotope peaks used for i-FIT = 3

Monoisotopic Mass, Even Electron Ions

312 formula(e) evaluated with 2 results within limits (all results (up to 1000) for each mass)

Elements Used:

C: 0-50 H: 0-50 N: 0-2 O: 0-3 Na: 0-1 S: 0-2

Mass	Calc. Mass	mDa	PPM	DBE	Formula	i.	Fit Conf %	C	H	N	O	Na	S
456.1063	456.1058	0.5	1.1	22.5	C30 H18 N O2 S	61...	0.00	30	18	1	2		1
	456.1068	-0.5	-1.1	14.5	C25 H23 N O2 Na S2	60...	100.00	25	23	1	2	1	2

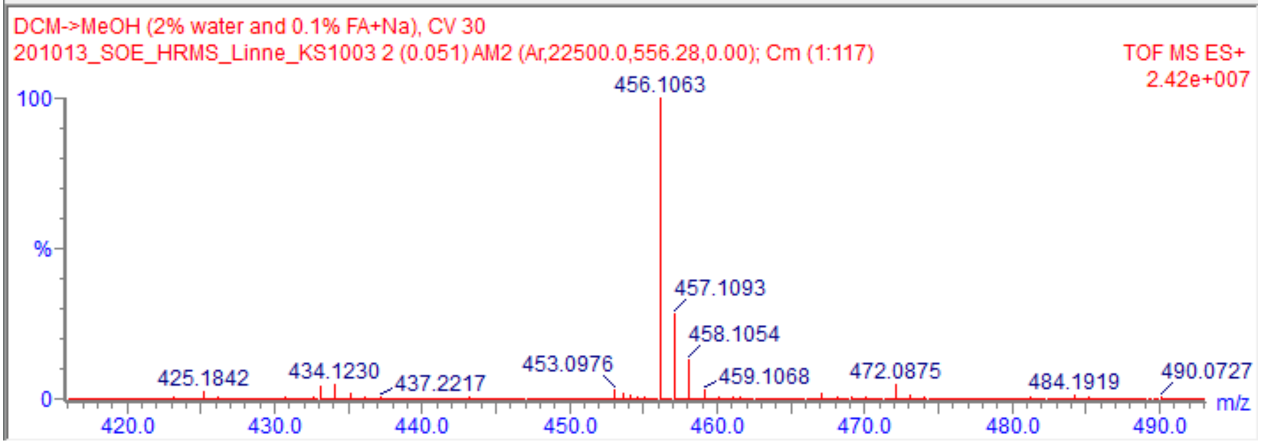

^{1}H NMR spectrum of 4h

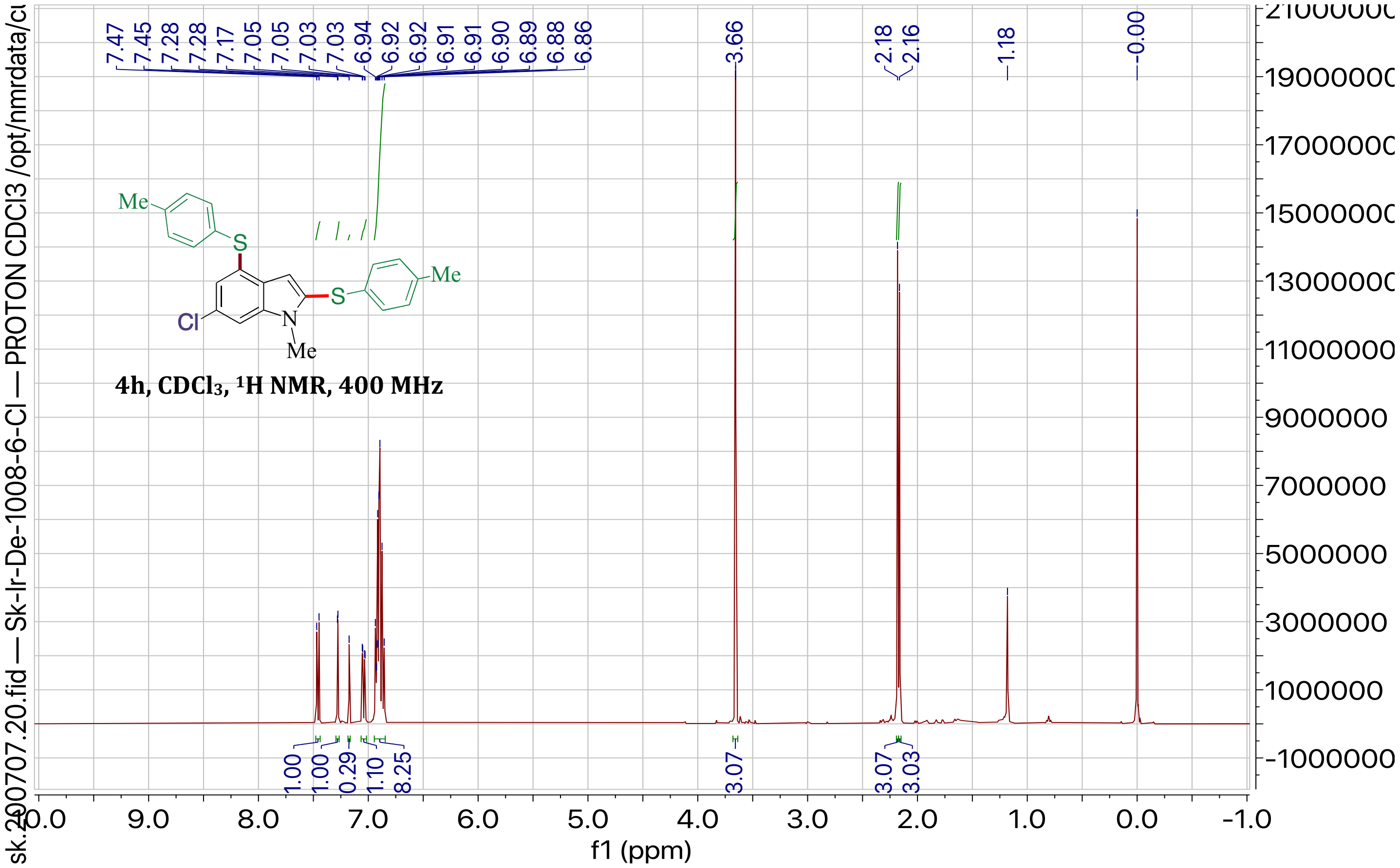

^{13}C{^{1}H} NMR spectrum of 4h

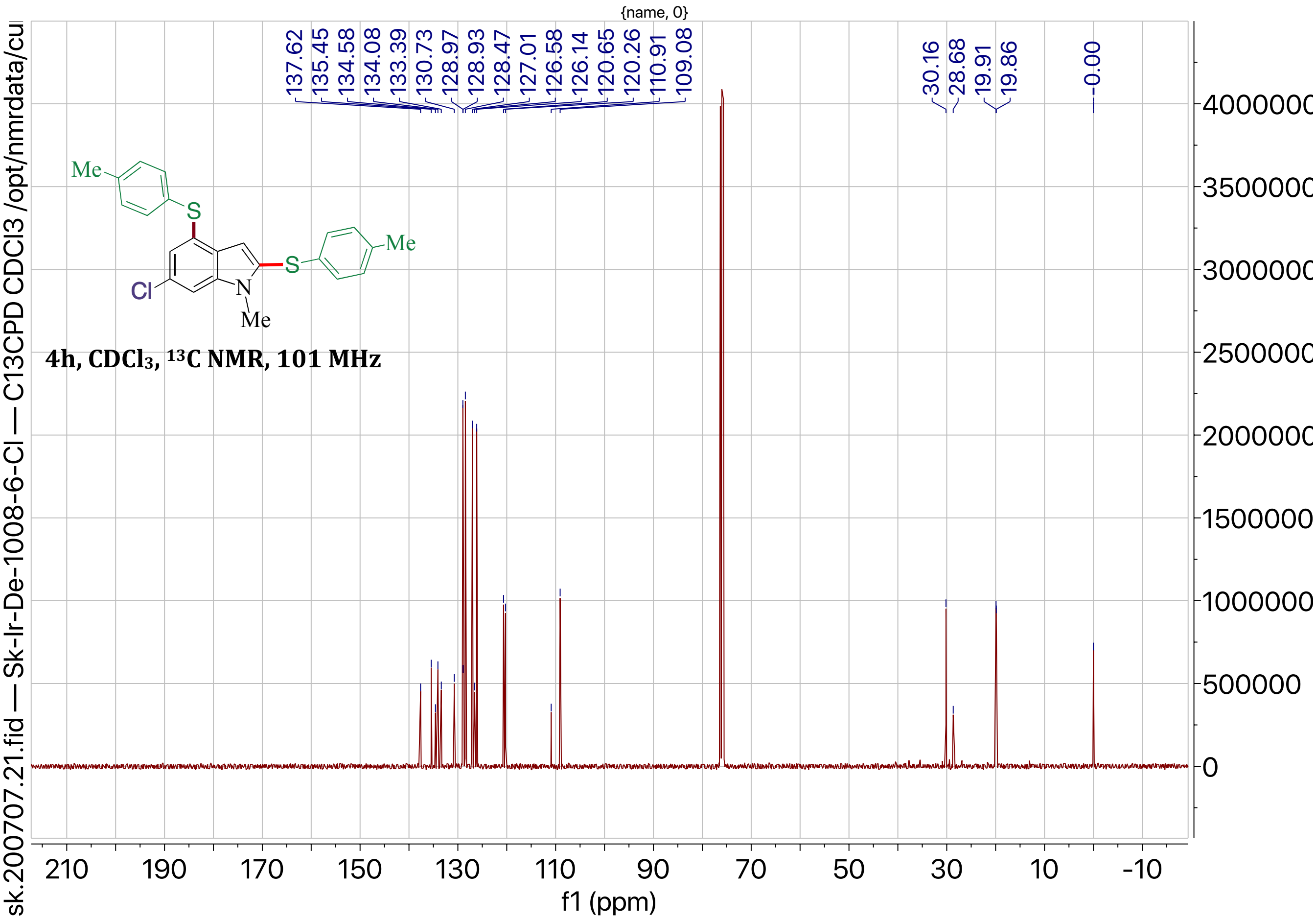

HRMS spectrum of 4h

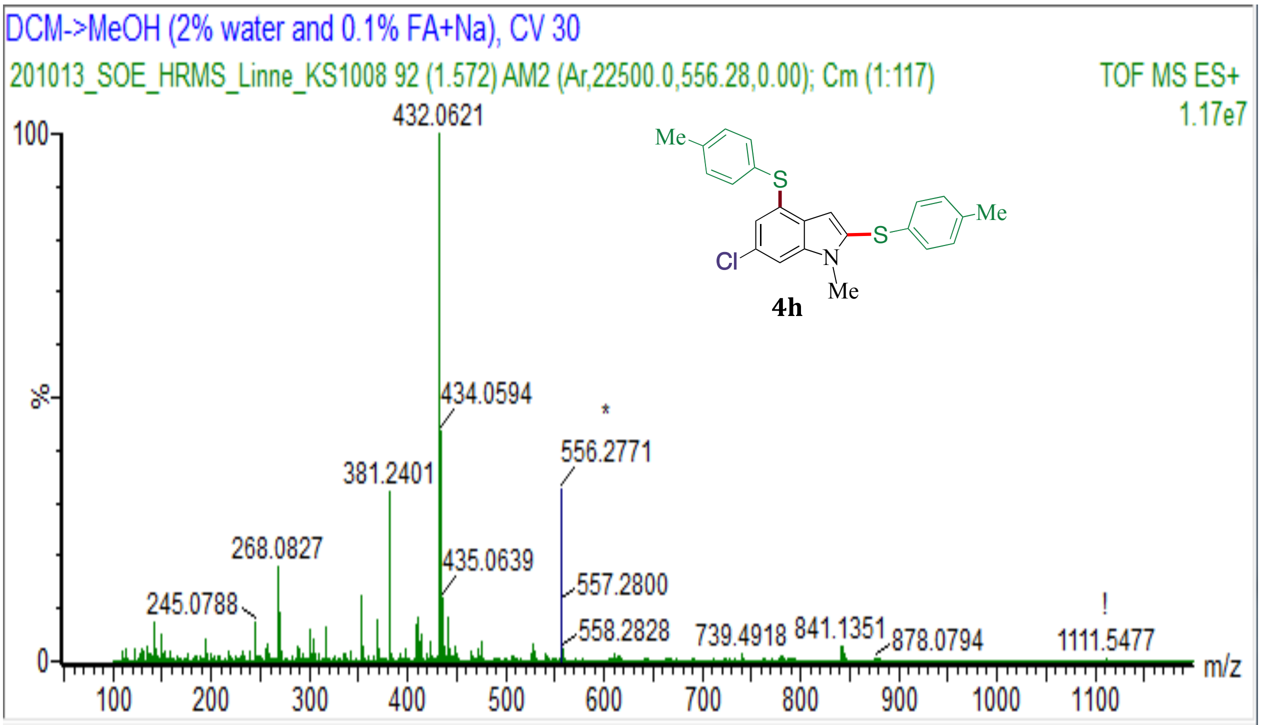

Single Mass Analysis

Tolerance = 2.0 mDa / DBE: min = -0.5, max = 100.0

Element prediction: Off

Number of isotope peaks used for i-FIT = 3

Monoisotopic Mass, Even Electron Ions

305 formula(e) evaluated with 2 results within limits (all results (up to 1000) for each mass)

Elements Used:

C: 0-50 H: 0-50 N: 0-2 O: 0-3 Na: 0-1 S: 0-2

Cl: 1-1

Mass	Calc. Mass	mDa	PPM	DBE	Formula	i...	Fit Conf %	C	H	N	O	Na	S	Cl
432.0621	432.0623	-0.2	-0.5	13.5	C23 H20 N Na S2 Cl	5...	99.98	23	20	1		1	2	1
	432.0614	0.7	1.6	21.5	C28 H15 N S Cl	8...	0.02	28	15	1			1	1

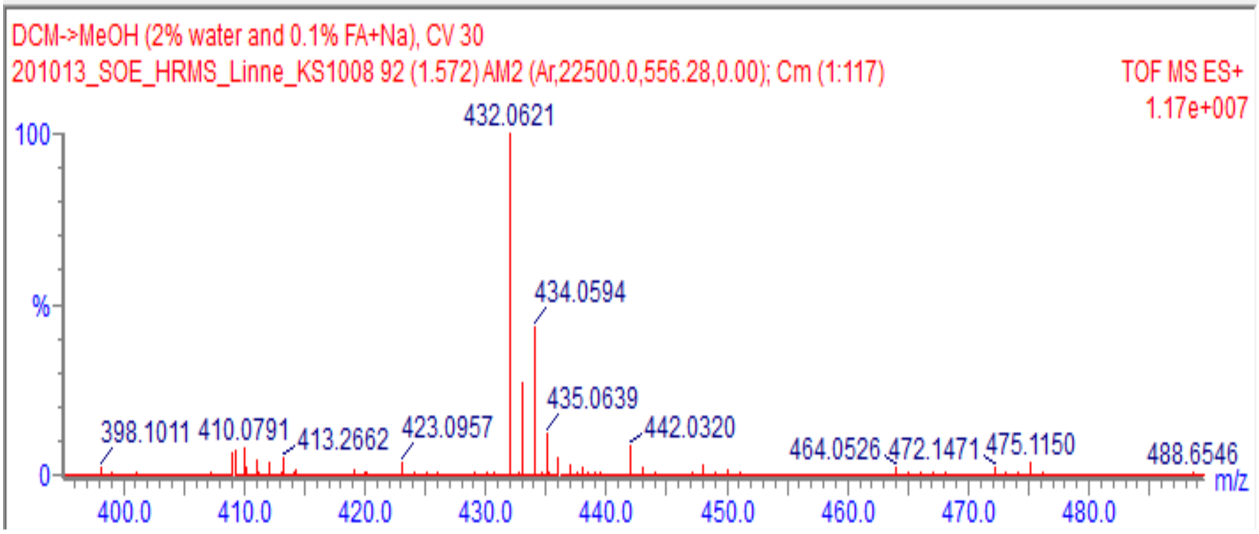

^1H NMR spectrum of 4i

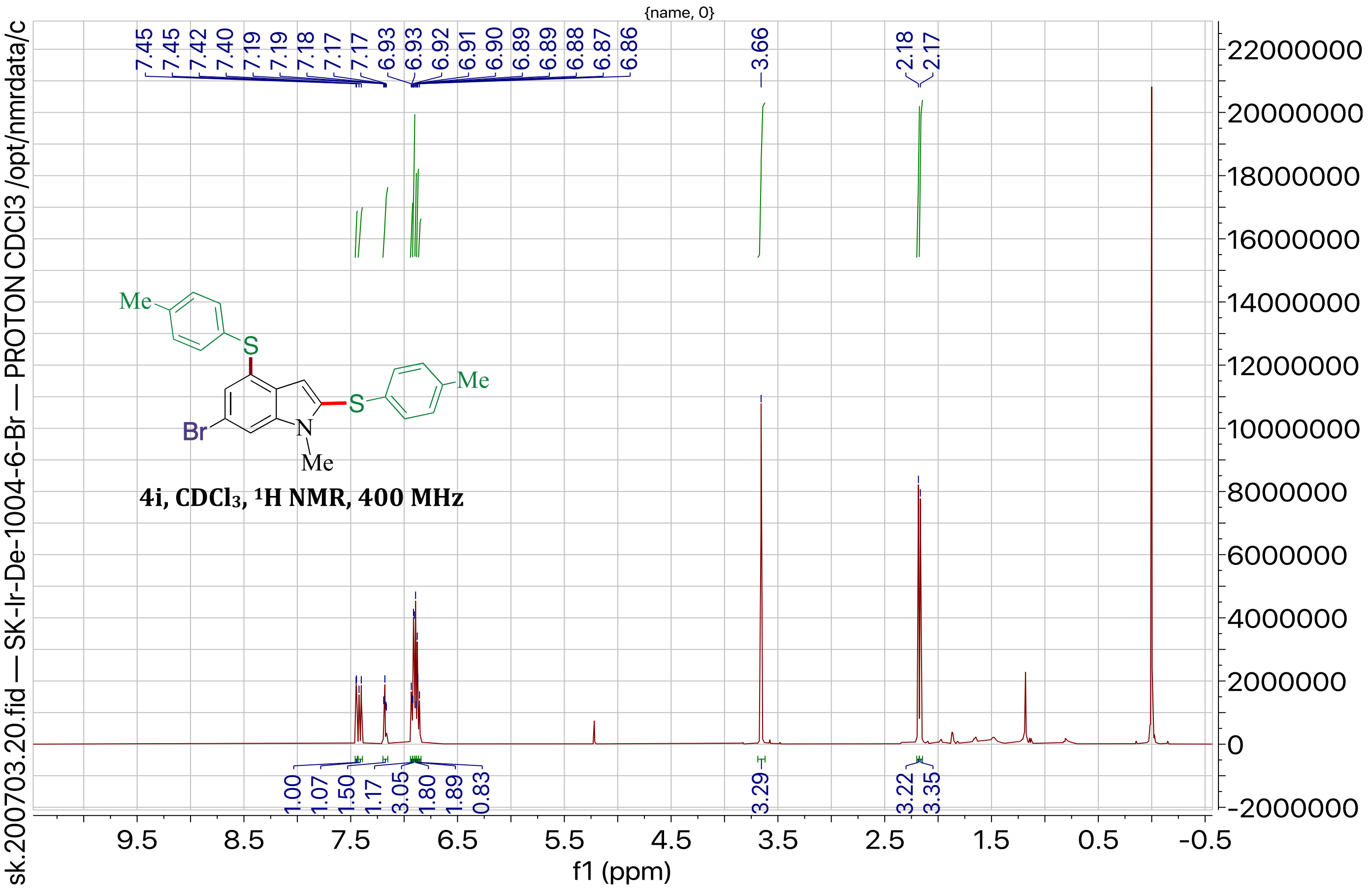

$^{13}C\{^1H\}$ NMR spectrum of 4i

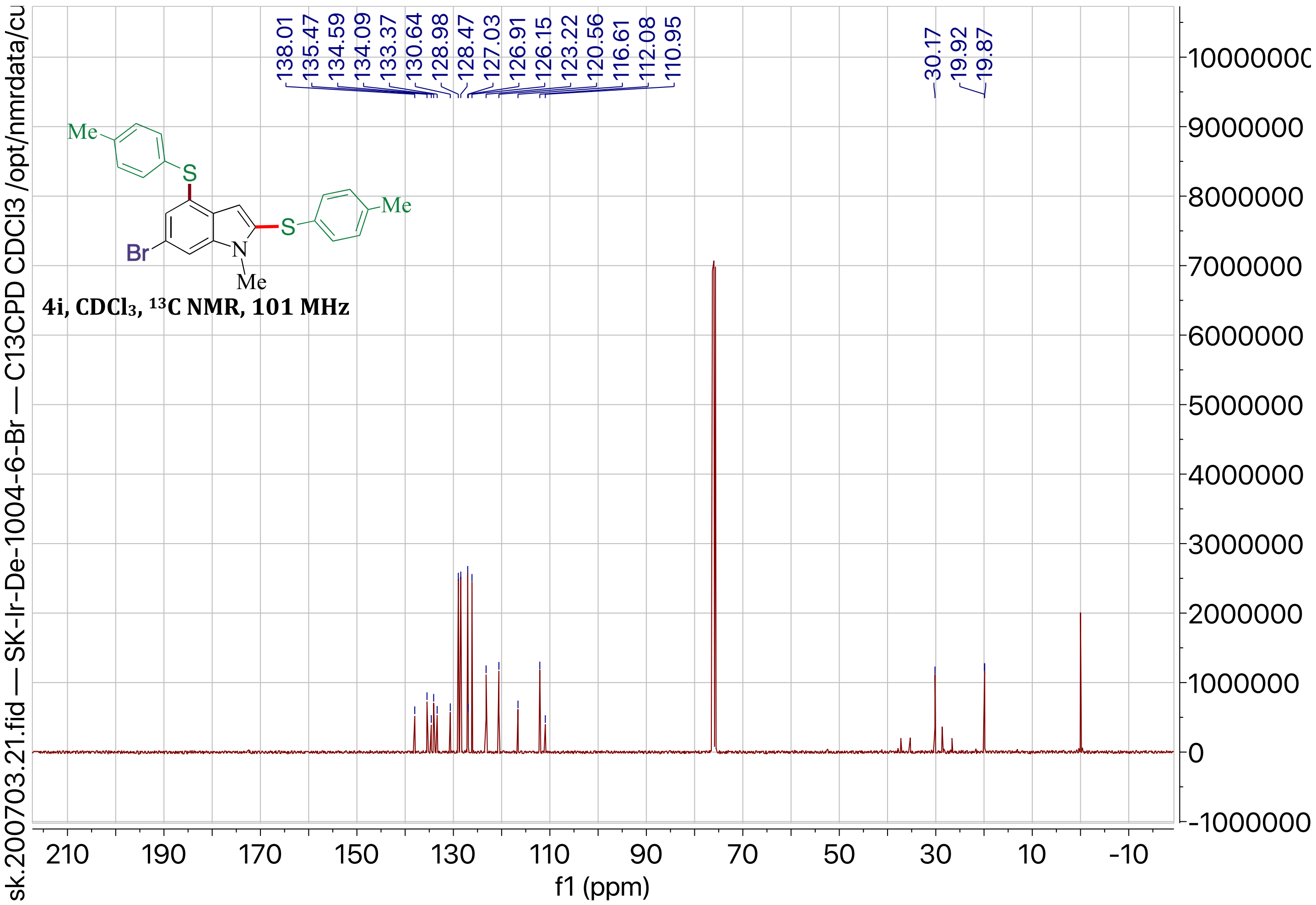

HRMS spectrum of 4i

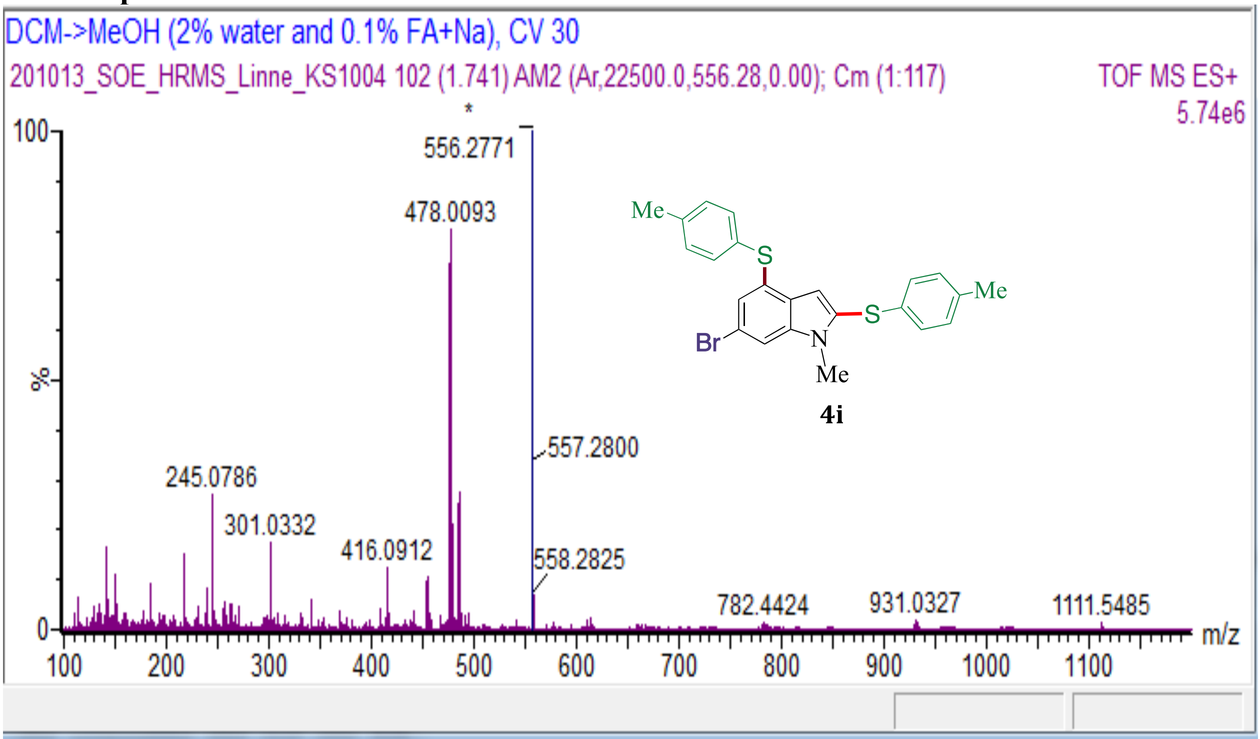

Single Mass Analysis
Tolerance = 2.0 mDa / DBE: min = -0.5, max = 100.0
Element prediction: Off
Number of isotope peaks used for i-FIT = 3
Monoisotopic Mass, Even Electron Ions
617 formula(e) evaluated with 3 results within limits (all results (up to 1000) for each mass)
Elements Used:
C: 0-50 H: 0-50 N: 0-2 O: 0-3 Na: 0-1 S: 0-2
Br: 0-1

Mass	Calc. Mass	mDa	PPM	DBE	Formula	i...	Fit Conf %	C	H	N	O	Na	S	Br
476.0113	476.0112	0.1	0.2	34.5	C35 H3 N O Na	5.2...	0.00	35	3	1	1	1		
	476.0109	0.4	0.8	21.5	C28 H15 N S Br	5.1...	0.00	28	15	1			1	1
	476.0118	-0.5	-1.1	13.5	C23 H20 N Na S2 Br	5.0...	100.00	23	20	1		1	2	1

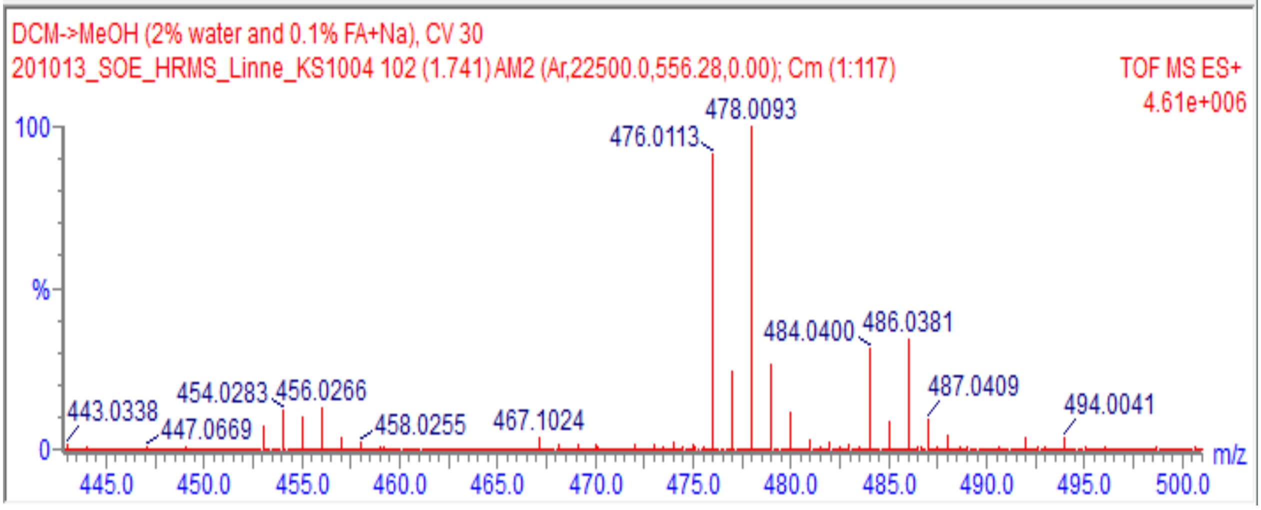

^{1}H NMR spectrum of 4j

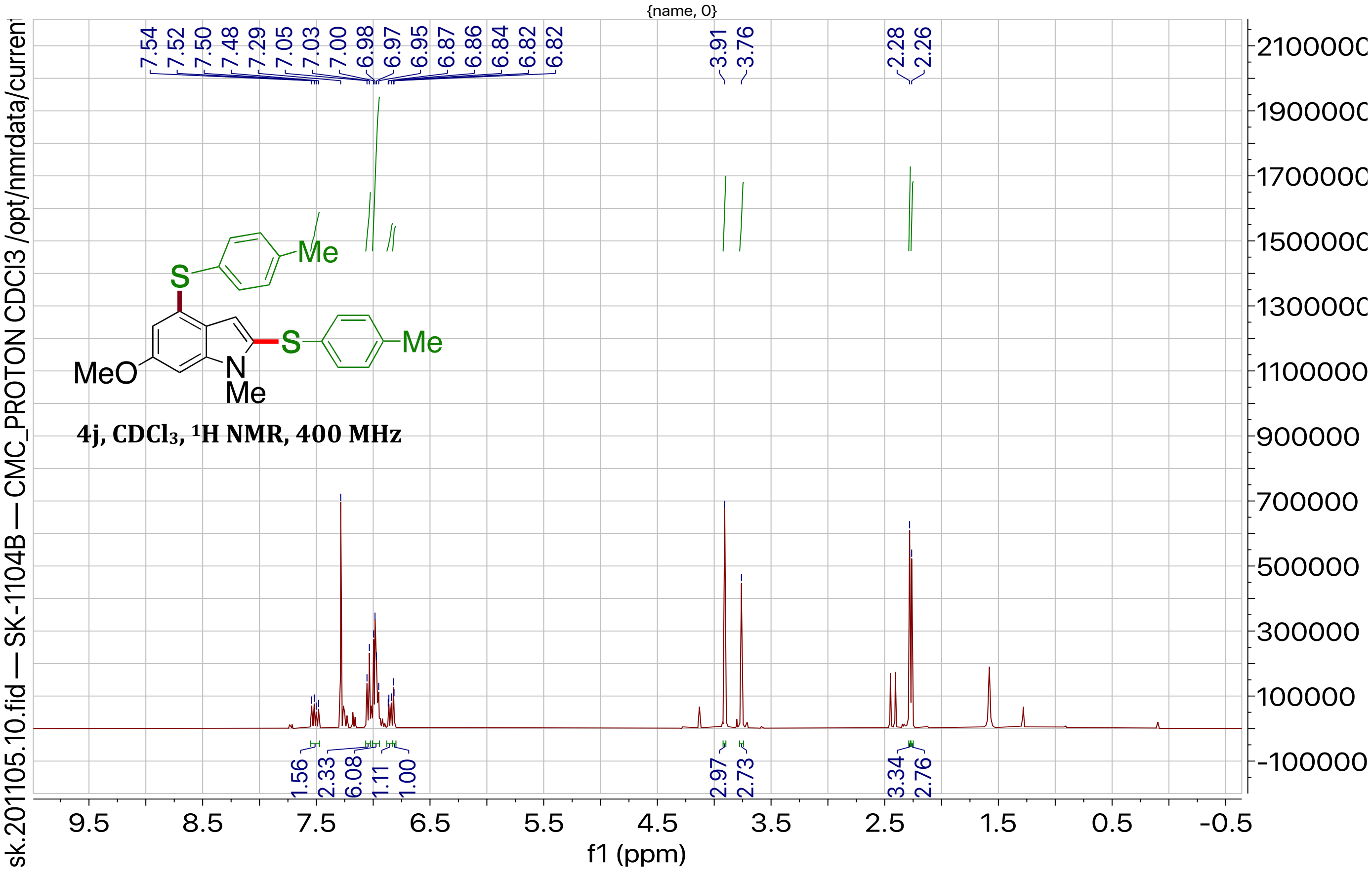

$^{13}C\{^{1}H\}$ NMR spectrum of 4j

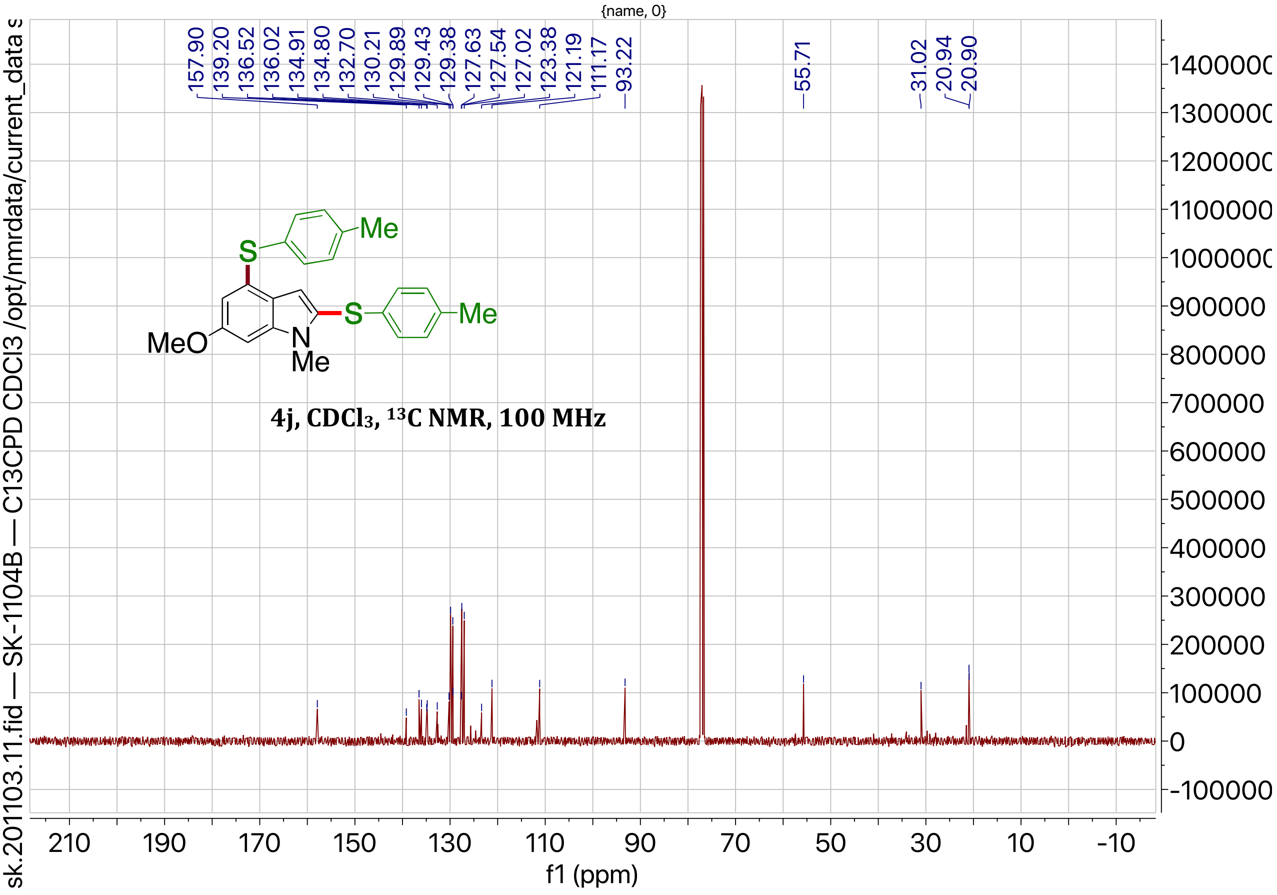

^{1}H NMR spectrum of 4k

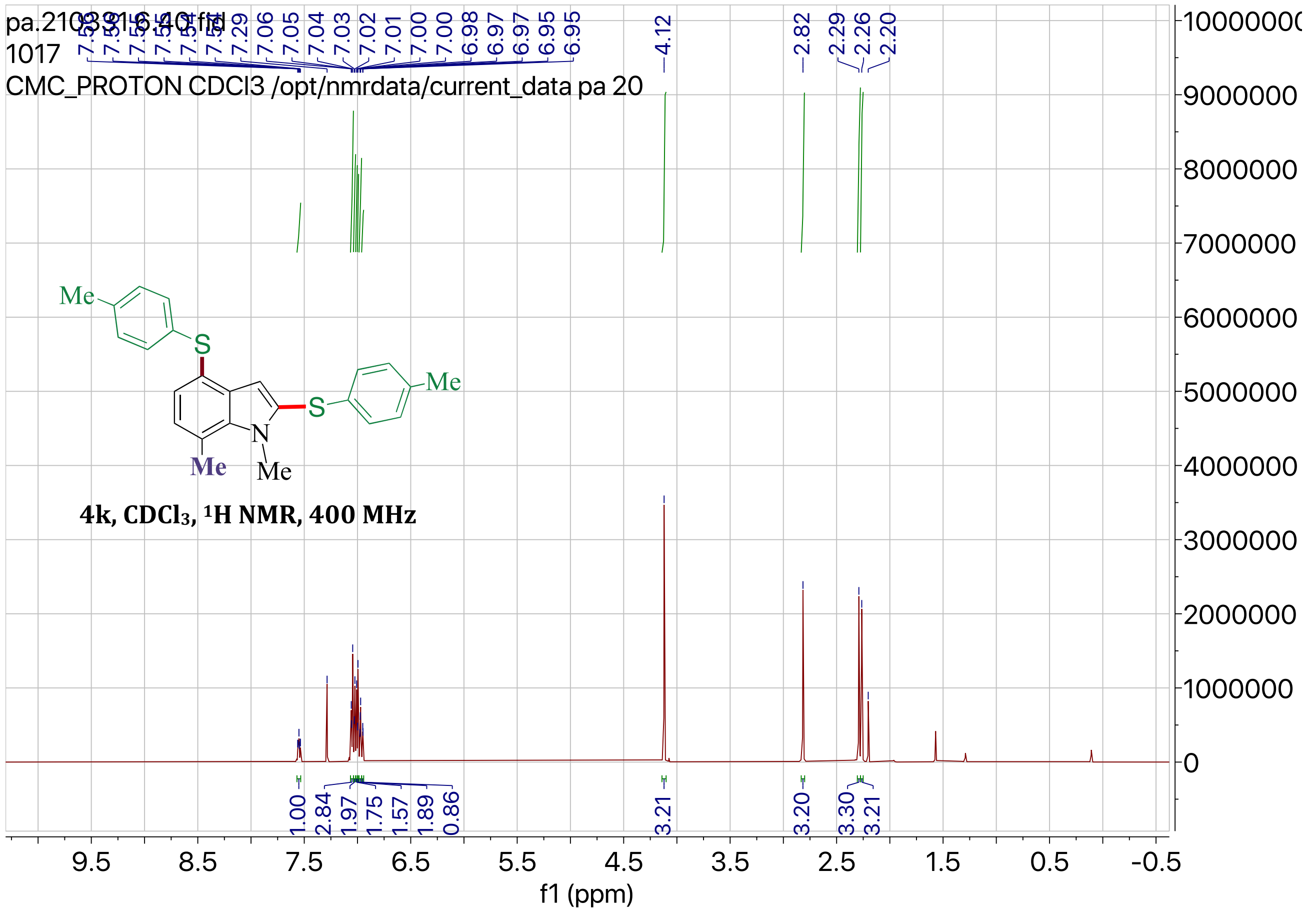

$^{13}C\{^{1}H\}$ NMR spectrum of 4k

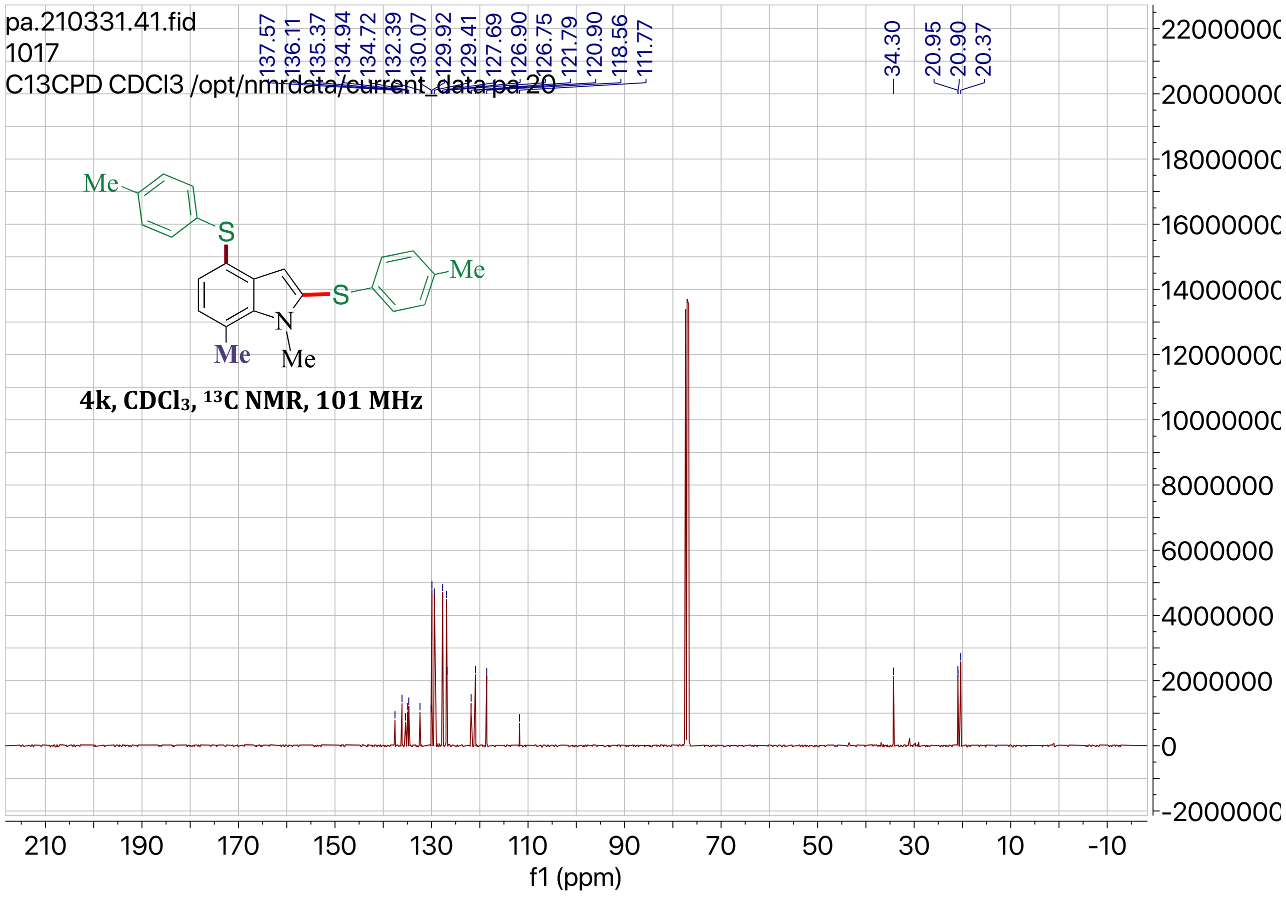

HRMS spectrum of 4k

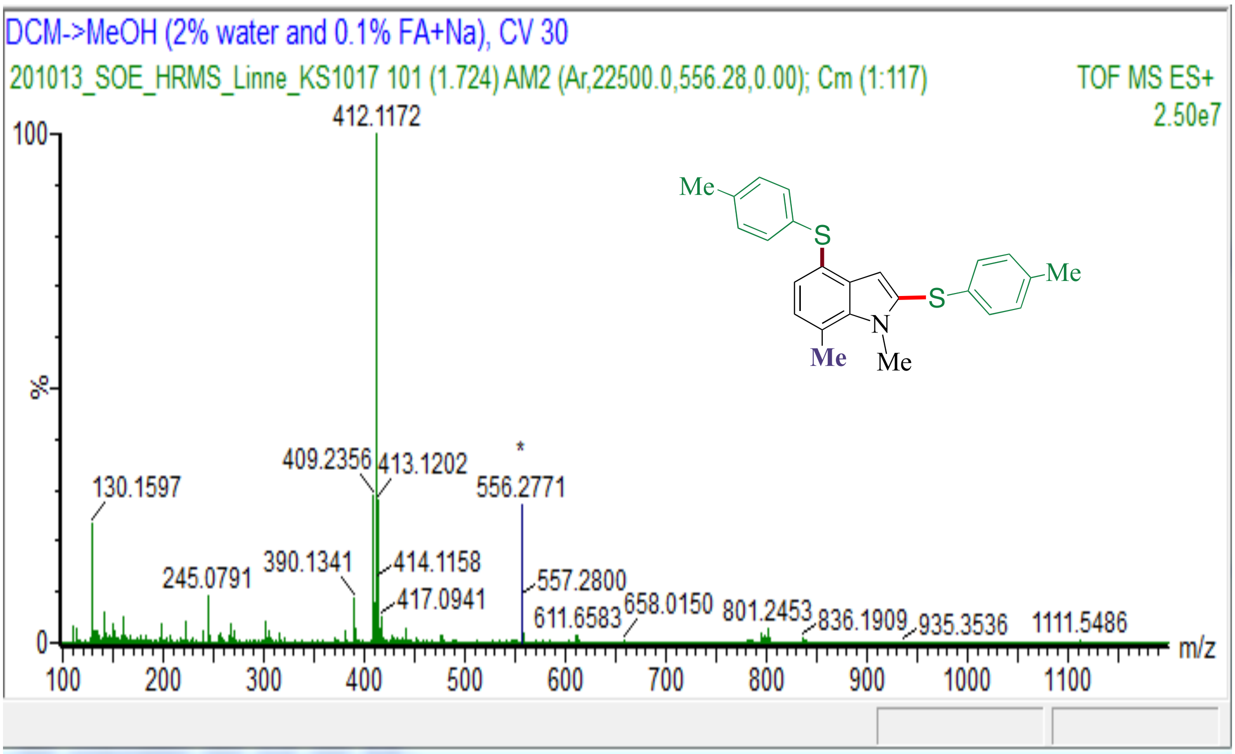

Single Mass Analysis

Tolerance = 2.0 mDa / DBE: min = -0.5, max = 100.0

Element prediction: Off

Number of isotope peaks used for i-FIT = 3

Monoisotopic Mass, Even Electron Ions

309 formula(e) evaluated with 2 results within limits (all results (up to 1000) for each mass)

Elements Used:

C: 0-50 H: 0-50 N: 0-2 O: 0-3 Na: 0-1 S: 0-2

Mass	Calc. Mass	mDa	PPM	DBE	Formula	i-FIT	i-FIT Norm	Fit Conf %	C	H
412.1172	412.1170	0.2	0.5	13.5	C24 H23 N Na S2	745.2	0.000	100.00	24	23
	412.1160	1.2	2.9	21.5	C29 H18 N S	760.4	15.263	0.00	29	18

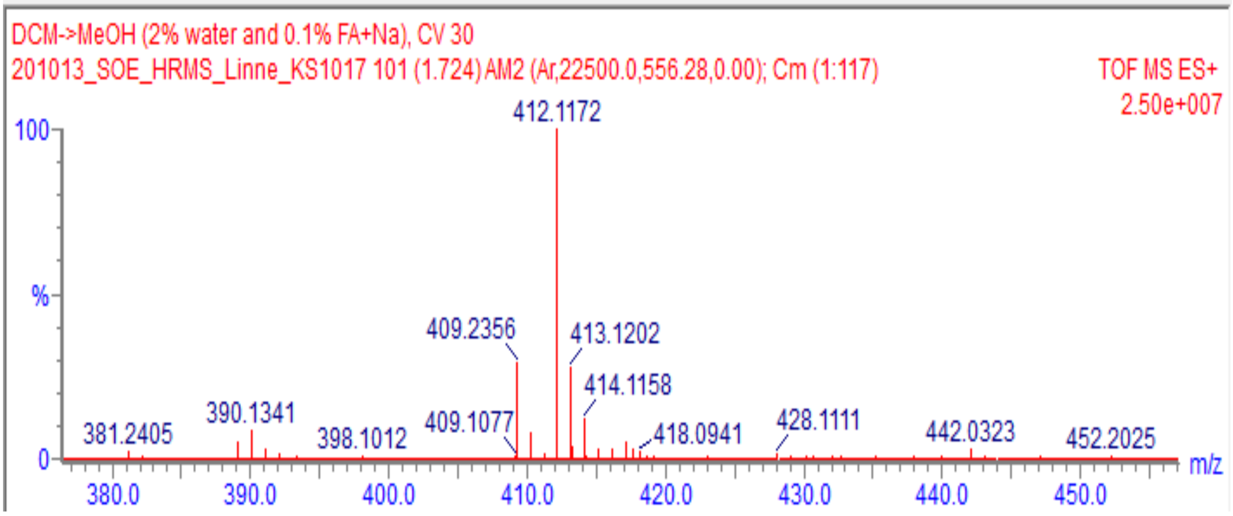

References

1. X. Chen, G. Zheng, Y. Li, G. Song, and X. Li*, Org. Lett*. 2017, **19**, 6184-6187.

2. S. K. Guchhait, M. Kashyap, and H. Kamble, *J. Org. Chem*. 2011, **76**, 4753-4758.

3. H. Ghafuri and M. H. Hashemi, *J. Sulphur. Chem*. 2009, **30**, 578-580.
